# Targeting 2D Nanostructures in Phase-Separated Materials through Molecular Design

**DOI:** 10.1021/acs.macromol.4c02691

**Published:** 2025-03-06

**Authors:** Martin H.C. van Son, Bart W.L. van den Bersselaar, Bas F.M. de Waal, Ghislaine Vantomme, E.W. Meijer

**Affiliations:** Laboratory of Macromolecular and Organic Chemistry and Institute for Complex Molecular Systems, Eindhoven University of Technology, P.O. Box 513, 5600MB Eindhoven, The Netherlands

## Abstract

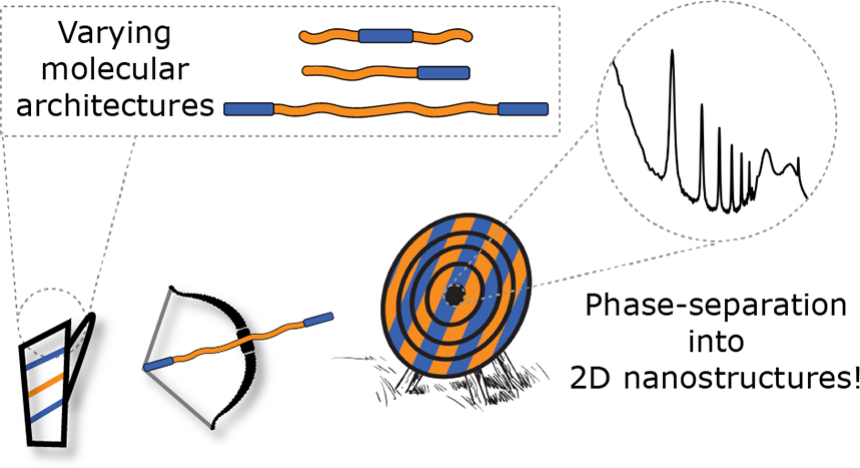

Oligomeric materials that spontaneously order into 2D morphologies are of interest for a broad range of applications. In the absence of molar mass dispersity, these materials are perfectly defined at the molecular level and have been shown to form sub-10 nm nanostructures. Consequently, such nanostructured oligomers exhibit intriguing properties for e.g., photophysical applications depending on their constituents. However, ab initio prediction of the obtained morphologies remains challenging. Therefore, we herein report a systematic approach to investigate the influence of molecular architecture as well as the influence of the pendant chain attached to the core on spontaneously phase-separated nanostructures. We synthesized 20 molecules containing discrete oligodimethylsiloxane (oDMS) and four different crystalline units, varying their molecular architecture and pendant chains. Lamellar morphologies were obtained most reliably using telechelic and head–tail architectures with symmetric peripheral crystalline blocks. Contrarily, these architectures in conjunction with asymmetric cores as well as core-centered architectures resulted primarily in columnar morphologies. This systematic investigation of the design parameters for 2D nanostructures aids the development of next-generation materials, e.g., nanoscale optoelectronics.

## Introduction

In the development of new materials for nanotechnologies, it is essential to have control over their nanostructure, its periodicity and domain size.^1^ In this context, oligomeric materials are of increasing interest, as their characteristics combine the precision of directional interactions in small molecules with the mechanical properties and functionality of polymers.^2^ As a result, oligomeric materials have found applications in various fields such as biotechnology, self-healing materials and optoelectronics.^3−5^ When immiscible building blocks are linked in oligomeric materials, spontaneous phase separation into well-defined morphologies with sub 5 nm periodicity is observed.^6,7^ Such nanostructures are ubiquitous in the fields of liquid crystals and block copolymers (BCPs), and their formation is predicted using established theories. For example, the formation of nanostructures in surfactants is rationalized by Israelachvili’s packing parameter for amphiphiles, which dictates their morphology based on shape anisotropy and space filling arguments.^8^ Similarly, maximizing interaction energy while minimizing excluded volume has been described as key design principles for thermotropic liquid crystals.^9,10^ Additionally, the driving force for phase separation in BCPs is known to be a combination of the immiscibility (χ), degree of polymerization (*N*) and the volume fraction of the two blocks (φ).^11,12^

**Figure 1:**
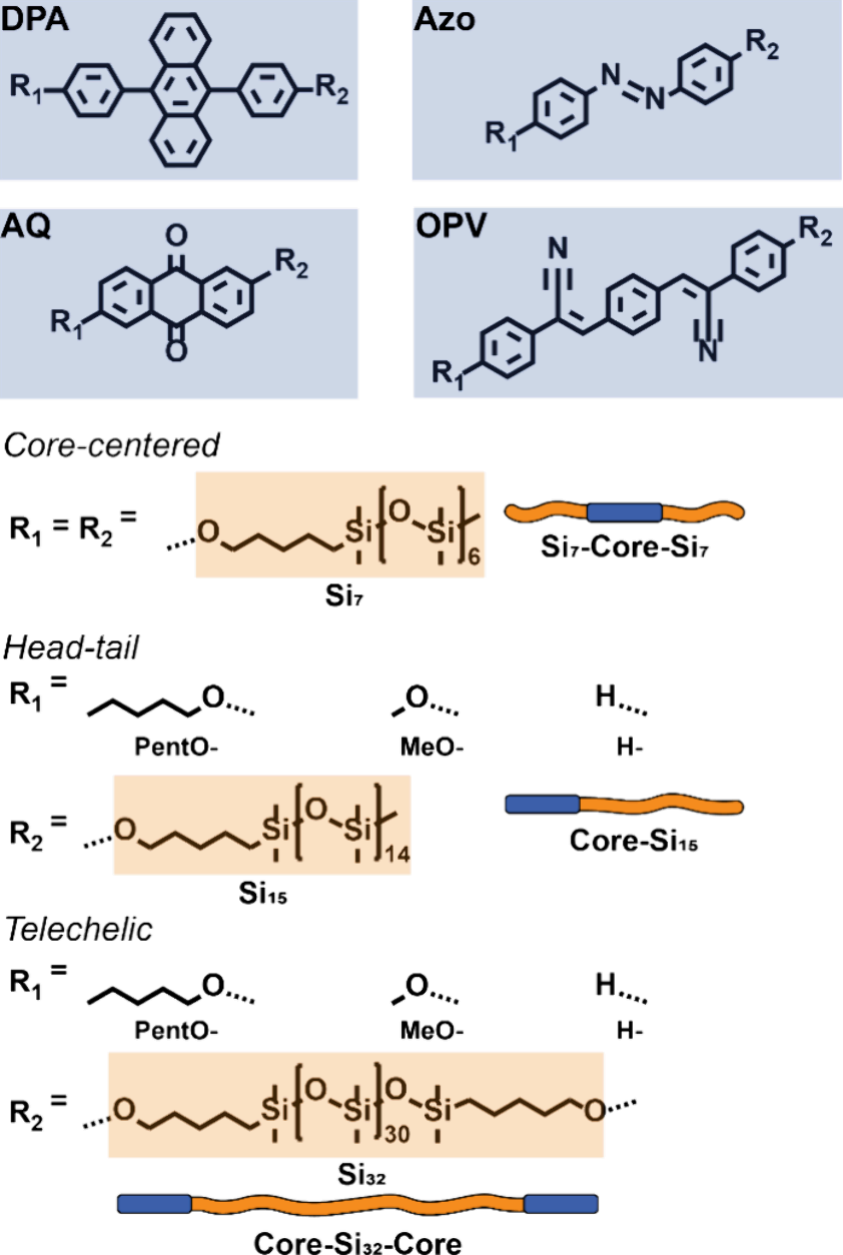
Chemical structures and schematic representations of the molecules used in this study. The four different cores (blue) DPA, Azo, AQ, and OPV are attached either on both sides with Si_7_ (core-centered), attached on a single side with Si_15_ (head–tail), or attached on the periphery of Si_32_ (telechelic). Head–tail and telechelic DPA and Azo were decorated with a pentoxy (**PentO-**) terminus (R1), while AQ and OPV were also substituted with a methoxy (**MeO-**) or hydride (**H-**) group.

To study such nanostructures in oligomeric materials, our group has developed a protocol to synthesize monodisperse (*Đ* < 1.00001) oligodimethylsiloxane (*o*DMS).^13^ Due to the high immiscibility of *o*DMS with various crystalline units, this design has proven successful in making “high χ-low *N*” materials with well-defined morphologies.^14,15^ We have shown that pathway control between 1D and 2D nanostructures can be obtained in so-called block molecules by carefully adjusting their composition and temperature during sample processing.^16^ Besides, we have found that especially lamellar nanostructures proved useful for various applications such as circularly polarized light emission and tunable conductivity.^17,18^ Recently, the origin of the spontaneous formation of these 2D morphologies was explained using Crystal Lattice Analysis, aiding the a priori prediction of nanostructures in novel materials.^19^ Herein, we aim to further elucidate the influence of molecular design and chain-end effects on the obtained nanostructures in *o*DMS-based block molecules. Hereto, we have selected a library of block molecules comprising four core molecules (**DPA**, **Azo**, **AQ**, **OPV**, Figure 1). These aromatic cores were selected based on their similarities as conjugated crystalline materials, yet varying crystalline packing.^20−23^ Thereby, we aim to unveil general design rules to target 2D nanostructures in block molecules comprising aromatic crystalline cores and discrete *o*DMS. The cores were decorated with *o*DMS chains to result in either core-functionalized, head–tail or telechelic block molecules, following the nomenclature of recent literature.^24^ Moreover, the chain-end effects were studied in the **AQ** and **OPV** cores by varying the terminal functionalization (**PentO-**, **MeO-**, and **H-**). To ensure a fair comparison between the block molecules, the volume fraction of the non-*o*DMS block (φ_non-oDMS_) was kept constant (14, 15, or 16 silicon atoms per core molecule for center-functionalized, head–tail or telechelic, respectively). All materials were investigated by differential scanning calorimetry (DSC), polarized optical microscopy (POM) and (Variable Temperature) Medium/Wide Angle X-ray Scattering (VT-MAXS/VT-WAXS) to obtain detailed information about their thermal and morphological properties. Using this approach, we established design strategies for block molecules to assemble into 2D morphologies.

## Results and Discussion

### Synthesis

All molecules were synthesized by first preparing the symmetric and asymmetric cores (Figure 1, Supporting Information Paragraph 2). The synthesis of **DPA** (Scheme S1), **AQ** (Scheme S2), and **Azo** (Scheme S3) derivatives started with a Williamson etherification on the mono- or dihydroxylated core using potassium carbonate as a base, unless stated otherwise. For the core-centered architectures, olefin terminated side chains were attached on both sides of the crystalline unit. Pentoxy-terminated cores for head–tail and telechelic architectures were prepared by monoalkylation of dihydroxylated cores with 1-bromopentane and subsequent reaction with 5-bromo-1-pentene. To obtain the **MeO-AQ** core (**7**), 2,6-dihydroxyanthraquinone was coupled with 5-bromo-1-pentene, and subsequently with dimethyl sulfate. The **H-AQ** core (**8**) was obtained through a reaction of 2-hydroxyanthraquinone with 5-bromo-1-pentene using potassium iodide as a catalyst. **OPV** (Scheme S4) cores were prepared starting from a Williamson etherification using K_2_CO_3_ on 4-hydroxyphenylacetonitrile with either 5-bromo-1-pentene or 1-bromopentane. Subsequently, the olefin decorated precursor (**12**) was reacted in a Knoevenagel condensation with terephthaldehyde to yield the symmetric core necessary for the core-centered material. Asymmetric **PentO-OPV** (**16**), comprising both a saturated and unsaturated linker, was required for the head–tail and telechelic architectures and was prepared using a Knoevenagel condensation on hemiacetal protected benzaldehyde. Deprotection under acidic conditions followed by a Knoevenagel condensation with the olefin terminated precursor yielded the **PentO-OPV** core (**16**). Similarly, **MeO-OPV** (**18**) and **H-OPV** (**20**) were prepared by reaction of 4-methoxyphenylacetonitrile and phenylacetonitrile with the aforementioned protected benzaldehyde. Discrete *o*DMS mono- and dihydrides were synthesized via a stepwise procedure, previously described by our group.^25^ The appropriate *o*DMS-(di)hydrides were attached to the corresponding olefins through a platinum catalyzed hydrosilylation to obtain the target molecules in moderate to excellent yield (24–80%). Core-centered materials were waxy to liquid materials at room temperature, indicating different types of packing from the solid materials formed by the head–tail and telechelic architectures. All compounds were characterized by ^1^H NMR, ^13^C NMR and mass spectrometry (MALDI-TOF-MS). Thereafter, recycling GPC in THF was used to ensure high purity of the materials before the characterization of their properties with DSC, POM and MAXS/WAXS (Figures S1–S22, Tables S1–S5).

### Influence of Molecular Design

To gain a deeper understanding of the requirements for formation of 2D nanostructures, the thermal and morphological properties of all molecules were studied in-depth. Here, the results for the core-centered materials are discussed first, followed by the head–tail and telechelic architectures with symmetric cores (Figure 1, **PentO-**). Finally, the differences between **PentO-**, **MeO-, H-AQ**, and **-OPV** functionalized molecules are clarified. Herein, only **AQ** and **OPV** were chosen to study the chain-end effects. The **DPA** core was excluded as its desymmetrization was expected to have a negligible influence on the assembly due to the strong interaction energies of overlapping aromatic systems. Additionally, the **Azo** core was excluded to prevent erroneous interpretation of the results due to cis–trans isomerization.^26^ All molecules were designed such that the formation of a columnar hexagonal (Col_h_) phase would be predominant when arguments based on block copolymer theory were followed (φ_non-oDMS_ = 0.21–0.31).

**Figure 2:**
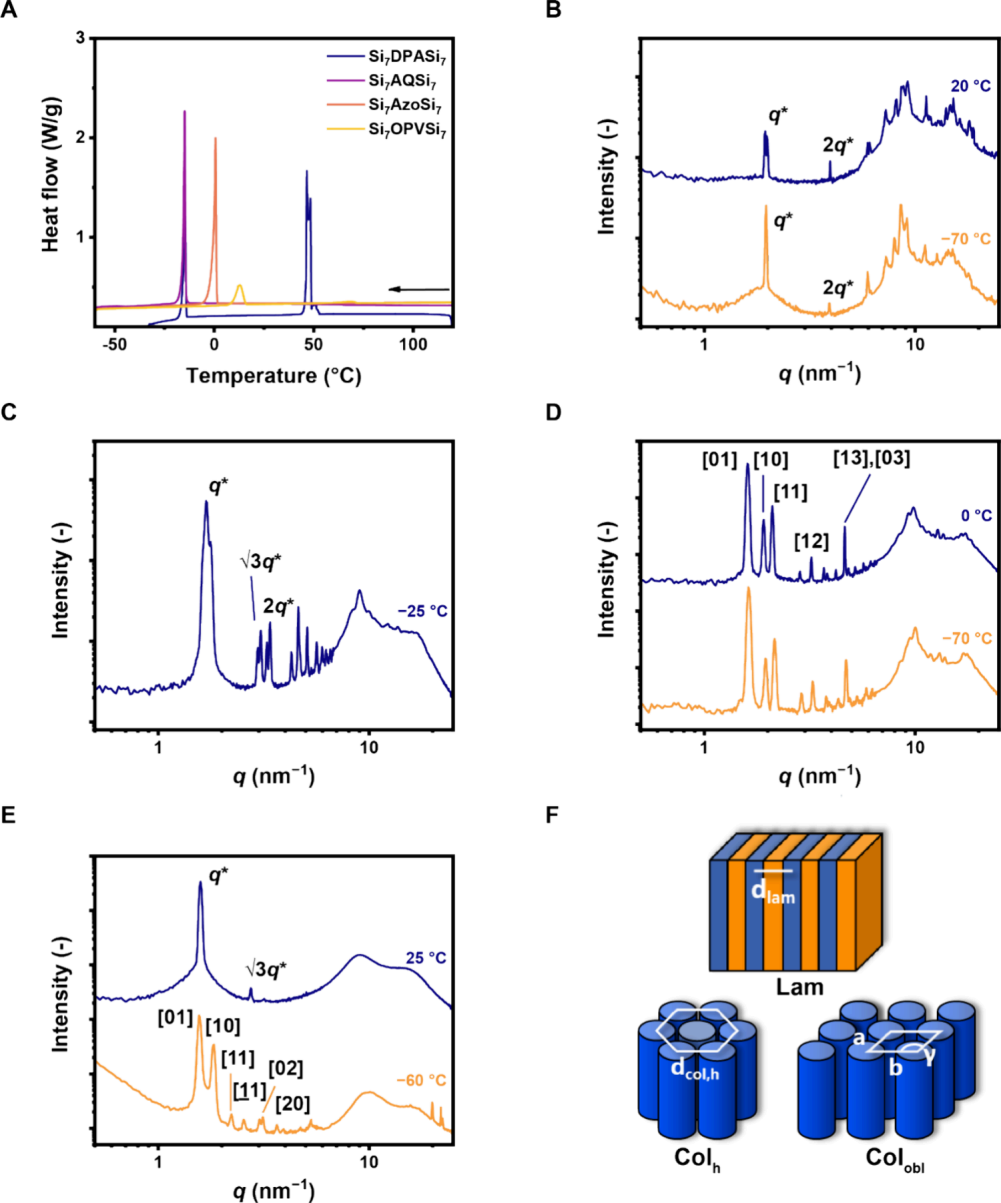
(A) DSC traces of all core-centered molecules showing the exothermic transitions. Exothermic transitions of the second cooling run with 10 K min^–1^ are depicted. (B) MAXS/WAXS spectra of **Si**_**7**_**-DPA-Si**_**7**_. The first peak *q* at 20 °C consists of two peaks in proximity q* and *q*′. The integer-spaced Bragg’s reflection peaks (q*, 2q*) indicate Lam ordering. (C) MAXS/WAXS spectra of **Si**_**7**_**-AQ-Si**_**7**_ showing Col_h_ order. (D) MAXS/WAXS spectra of **Si**_**7**_**-Azo-Si**_**7**_ showing a Col_obl_ pattern at both 0 and −70 °C. (E) MAXS/WAXS spectra of **Si**_**7**_**-OPV-Si**_**7**_ showing a Col_h_ packing at 25 °C and a Col_obl_ packing at −60 °C. (F) Schematic representations of the most common morphologies in this study; Lam, Col_h_ and Col_obl_.

First, the thermal properties of all core-centered molecules were characterized by DSC (Figure 2A, Table 1). **Si**_**7**_**-DPA-Si**_**7**_ (Figure S1), **Si**_**7**_**-Azo-Si**_**7**_ (Figure S11), and **Si**_**7**_**–OPV-Si**_**7**_ (Figure S14) showed two endothermic thermal transitions (*T*_Endo_), while **Si**_**7**_**-AQ-Si**_**7**_ (Figure S4) only displayed one transition. All *T*_Endo_ had corresponding exothermic thermal transitions (*T*_Exo_), except for **Si**_**7**_**-Azo-Si**_**7**_. Here, the absence of a second *T*_Exo_ was likely due to the presence of such a transition below the detection limit of our instrument (−70 °C). We correlate the presence of two endothermic transitions to the formation of mesophases, similar to typically observed for thermotropic liquid crystals, based on the difference in nanomorphologies as apparent from the MAXS/WAXS patterns of **Si**_**7**_**-DPA-Si**_**7**_ (Figure S1) and **Si**_**7**_**-OPV-Si**_**7**_ (Figure S14) at different temperatures.

**Table 1:** Morphological and Thermal Characterization of Molecules with Symmetric Cores

name^a^	φ_non-oDMS_ (−)^b^	phase^c^	*d* (nm)^d^	*T*_Endo_ (°C)^e^	Δ*H*_Endo_ (kJ mol^–1^)^f^	*T*_Exo_ (°C)^e^	Δ*H*_Exo_ (kJ mol^–1^)^f^
Si_7_-DPA-Si_7_	0.26	Lam^g^	3.1^g^	–39 ± 1	26	–44 ± 1	24
				80 ± 1	44	60 ± 1	48
DPA-Si_15_	0.25	Lam	5.8	112 ± 1	35	102 ± 1	36
DPA-Si_32_-DPA	0.24	Lam	6.1	117 ± 2	32^f^	103 ± 2	33^f^
Si_7_-AQ-Si_7_	0.31	2 × Col_h_	3.7	–3 ± 2	21	–16 ± 1	19
			3.5				
AQ-Si_15_	0.30	Lam	5.9	32 ± 1	23	20 ± 2	23
AQ-Si_32_-AQ	0.28	Lam	6.3	38 ± 2	22^f^	25 ± 1	19^f^
Si_7_-Azo-Si_7_	0.23	Col_obl_	*a* = 3.4	4 ± 2	26	–2 ± 1	24
			*b* = 4.1	–59 ± 2	2		
			γ = 74°				
Azo-Si_15_	0.22	Lam^h^	6.3	38 ± 1	17	36 ± 1	17
				13 ± 2^h^	1^h^		
Azo-Si_32_-Azo	0.21	Lam	6.7	45 ± 2	33^f^	41 ± 2	42^f^
Si_7_–OPV-Si_7_	0.26	Col_h_;	3.9	30 ± 1	11	18 ± 1	10
		Col_obl_	*a* = 3.5	83 ± 1	2^i^	82 ± 1	2^i^
			*b* = 4.0				
			γ = 82°				
OPV-Si_15_	0.25	Col_h_;	6.3	108 ± 2	14	105 ± 1	14
		Col_obl_	*a* = 5.8	132 ± 2	1^i^^,^^j^	129 ± 2	1^i^^,^^j^
			*b* = 6.5				
			γ = 83°				
OPV-Si_32_–OPV	0.24	Col_h_	5.8	107 ± 2	15^f^	104 ± 1	17^f^
		Lam	5.1	134	n.d.^i^^,^^j^	131 ± 2	1^f^^,^^i^

a Block molecules as depicted in Figure 1 with a **PentO**-group on both sides of the core.

b Volume fraction of the amorphous *o*DMS, calculated using bulk densities of PDMS (0.95 g mL^–1^),^29^ and the densities of the crystal structures of **DPA**,^30^**C**_**3**_**AQC**_**3**_,^31^**C**_**6**_**AzoC**_**6**_,^32^ and **C**_**4**_**OPVC**_**4**_.^33^ The alkyl spacers were taken as part of the core molecules in the calculation of the core molecular weight.

c Morphology of the nanostructure determined with SAXS below the temperature of the lowest exothermic peak. Lam = Lamellar, Col_h_ = Columnar hexagonal, Col_obl_ = Columnar oblique.

d Domain spacing (*d*) calculated using *d* = 2π/q*, with q* the principal scattering peak for Lam and Col_h_. Calculation of the lattice parameters of the Col_obl_ phases can be found in Tables S1–S3.

e Thermal transitions as determined by DSC from the second heating and cooling cycle using a heating and cooling rate of 10 K min^–1^. The endothermic transitions are attributed to melting of the material and the exothermic transitions are attributed to crystallization of the material unless otherwise specified.

f Enthalpic values reported per mole of core molecule.

g An additional principal scattering peak without any further reflections was found at 3.2 nm, contrary to previously reported.^34^

h Measured below the order–order transition at 13 °C.

i Classified as order–disorder (when endothermic) or disorder–order (when exothermic) transition due to their small enthalpic contribution.^35^

j Not determined as peak onset could not be resolved (Figures S16A and S20A).

The identified thermal transitions were used to probe the presence of nanoscale ordering of the molecules using POM at temperatures below the lowest *T*_Exo_. Herein, birefringent domains were found growing up to 1 mm in size. To study what type of ordering was present within these morphologies, we recorded MAXS/WAXS spectra below *T*_Exo_. First, **Si**_**7**_**-DPA-Si**_**7**_ phase-separated into a lamellar (Lam) morphology with a spacing (*d*_lam_) of 3.2 nm (Figures 2B and S1). Contrarily, columnar phases were found for the other three core-centered molecules. The diffraction pattern of **Si**_**7**_**-AQ-Si**_**7**_ revealed the presence of coexisting hexagonally packed columnar phases (2 × Col_h_), as both principal scattering peaks *q′* and q* have subsequent reflection peaks (*q*, √3*q*, √4*q*, ···) corresponding to a Col_h_ phase (Figures 2C and S4). The presence of these two similar morphologies were hypothesized to stem from subtle differences in the packing of the crystalline unit. Next, **Si**_**7**_**-Azo-Si**_**7**_ formed a columnar nanostructure, wherein the columns are packed under an angle (columnar oblique, Col_obl_, Figures 2D and S11). Here, the dimensions of the unit cell were approximated from the reflection peaks as *a* = 3.4 nm, *b* = 4.1 nm, γ = 74° (Table S1). Similarly, below the lowest *T*_Exo_ (−60 °C) a columnar oblique packing was unveiled in the diffraction pattern of **Si**_**7**_**-OPV-Si**_**7**_ (Figures 2E and S14, Table S2). A visual representation of all observed morphologies is shown in Figure 2F.

Besides the long-range order of the materials that is deducted from the medium-angle region (MAXS, *q* < 7 nm^–1^), we characterized the core–core interactions from the wide-angle region (WAXS, *q* > 7 nm^–1^). Here, π–π stacking was clearly visible with distances (*d*_π–π_) 3.1 Å for **Si**_**7**_**-AQ-Si**_**7**_ and 3.2 Å for **Si**_**7**_**-Azo-Si**_**7**_. For **Si**_**7**_**-OPV-Si**_**7**_, two π–π distances were observed at 3.1 and 2.9 Å, which is characteristic for a Col_obl_ phase.^27^ Finally, the WAXS region of **Si**_**7**_**-DPA-Si**_**7**_ showed many sharp peaks, hampering identification of specific π–π stacking peaks. Nevertheless, the plethora of peaks underline the crystallinity of the DPA core. Next, a sharp peak was observed around 10 nm^–1^ in the spectra of **Si**_**7**_**-DPA-Si**_**7**_, **Si**_**7**_**-AQ-Si**_**7**_, and **Si**_**7**_**-Azo-Si**_**7**_ corresponding well to the diameter of an *o*DMS chain (∼7 Å).^28,29^ This feature implies that the motion of the *o*DMS chain was restricted in these materials, whereas a broad halo was observed at the same position for **Si**_**7**_**–OPV-Si**_**7**_, indicative of the amorphous nature of the *o*DMS.

**Figure 3:**
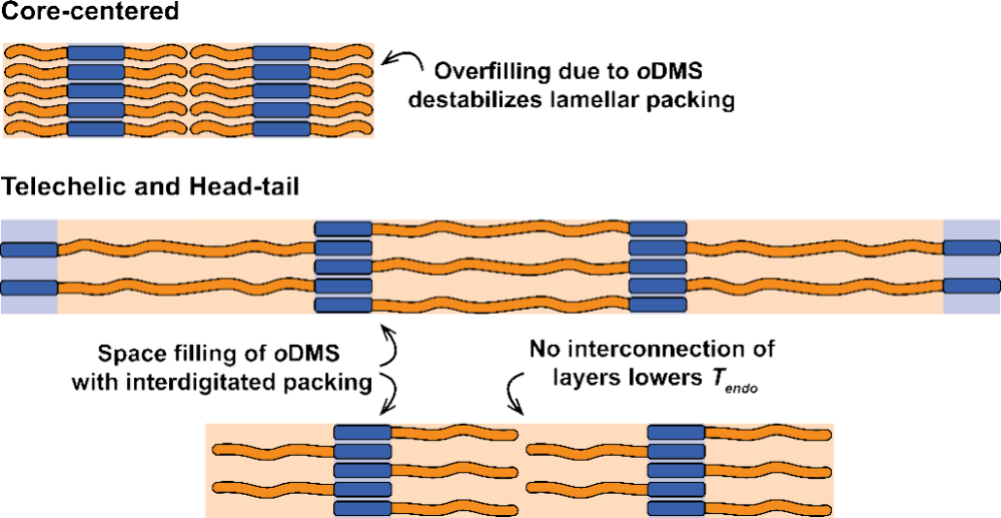
Schematic of the 2D Lam morphology of core-centered, telechelic and head–tail molecules. Space filling arguments previously reported in literature were used to explain the destabilization of 2D morphologies for the core-centered architecture.^36^

Contrary to the observations made for the core-centered molecules, both the head–tail and telechelic architectures of **DPA**, **AQ**, and **Azo** block molecules crystallized into a lamellar nanostructure. We relate this difference for **AQ** and **Azo** to the size of the *o*DMS chain compared to the aromatic core. In head–tail and telechelic architectures, each core was grafted with a single *o*DMS chain, effectively allowing for more space for the *o*DMS through the formation of an interdigitated packing where the *o*DMS of adjacent molecules point in alternating directions (Figure 3). As mentioned before, a clear scattering peak around 10 nm^–1^ was found for **Si**_**7**_**-DPA-Si**_**7**_, **Si**_**7**_**-AQ-Si**_**7**_, and **Si**_**7**_**-AZO-Si**_**7**_ hinting at partial immobilization of the *o*DMS side chain. We propose that the head–tail and telechelic molecules display an interdigitated packing with such immobilized *o*DMS chains (Figure 3), which is destabilized in a core-centered architecture due to overfilling of the oDMS side chains.^36^ For the telechelic architectures, the observed lamellar domain spacings (6.1, 6.3, and 6.7 nm for **DPA**, **AQ**, and **Azo**, respectively) correspond well to the theoretical spacing of an interdigitated packing (7.0, 6.7, and 6.7 nm for **DPA**, **AQ**, and **Azo**, respectively). Further confirmation was provided by the melting temperature *T*_Endo_, which is ∼6 °C lower in the head–tail compared to telechelic architectures while the enthalpic contributions were similar. We conclude that this difference in *T*_Endo_ originates from the lack of interconnection of the layers through the *o*DMS (Figure 3).

Interestingly, head–tail and telechelic block molecules based on **PentO-OPV** (**16**) showed phase behavior more reminiscent of thermotropic liquid crystals, as is common for such systems.^37,38^ For **PentO-OPV** molecules, predominantly columnar morphologies were found in the respective MAXS/WAXS patterns. Only upon cooling below the final *T*_Endo_ a lamellar morphology was observed in **OPV-Si**_**32**_**-OPV**. We ascribe this difference to the rod-shape anisotropy present in **OPV-Si**_**15**_, which is diminished in symmetrical **OPV-Si**_**32**_**-OPV**.^8^ Thus, combining the results of the three different architectures, the telechelic architecture should be used to most reliably target lamellar morphologies.

### Influence of Chain-End Effects

**Figure 4:**
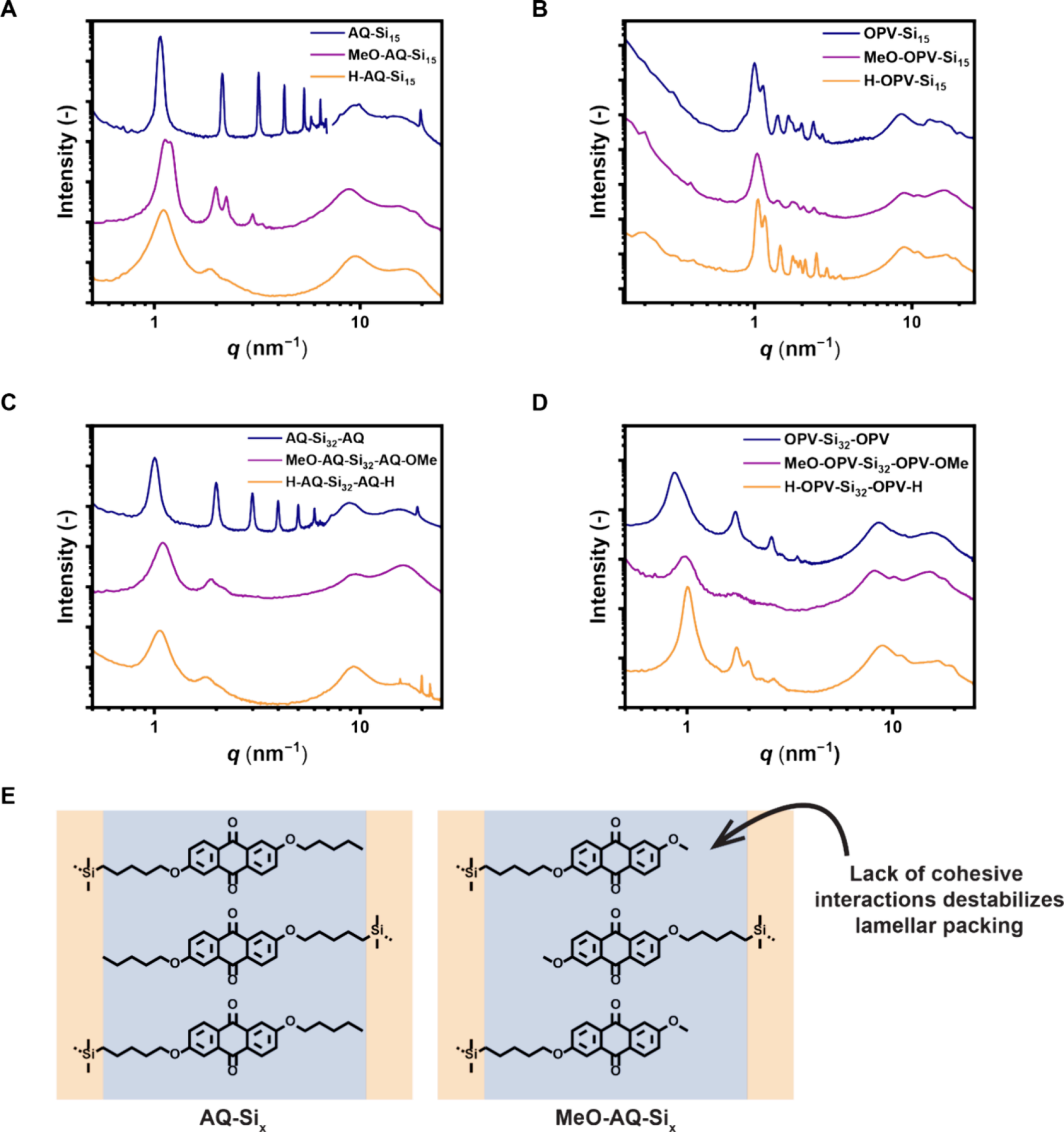
(A) MAXS/WAXS spectra of head–tail architecture AQ-based block molecules. **AQ-Si**_**15**_ (blue) phase separated into Lam stacks, whereas the latter two block molecules (purple, orange) showed Col_h_ packing. (B) MAXS/WAXS spectra of head–tail architecture OPV-based block molecules. All materials showed Col_obl_ packing. (C) MAXS/WAXS spectra of telechelic architecture AQ-based block molecules. **AQ-Si**_**32**_**-AQ** (blue) phase separated into Lam stacks, whereas the latter two block molecules (purple, orange) showed Col_h_ packing. (D) MAXS/WAXS spectra of telechelic architecture OPV-based block molecules. **OPV-Si**_**32**_**-OPV** (blue) phase separated into Lam stacks, whereas the latter two block molecules (purple, orange) showed Col_h_ packing. (E) Schematic indication of interdigitated packing of **AQ-Si**_*x*_ and **MeO-AQ-Si**_*x*_. The decrease in cohesive van der Waals interactions between alkyl chains in the latter case, as well as in **H-AQ-Si**_*x*_, destabilizes the Lam packing and causes the formation of Col_h_ domains.

The aforementioned results describe aromatic groups with pentoxy spacers on both sides. However, our group has previously shown that well-defined morphologies were observed in block molecules comprising asymmetric aromatic moieties, such as hydrazone or ureidopyrimidinone.^16,39^ Thus, to systematically study the effect of substituting the core with different pendants, the pentoxy (**PentO-**) terminus of head–tail and telechelic **AQ**- and **OPV**-block molecules was exchanged to either a methoxy-group (**MeO-**) or a hydride (**H-**). Their thermal and morphological properties were studied similarly using DSC and VT-MAXS/WAXS (Figure 4A–D, Table 2).

**Table 2:** Morphological and Thermal Characterization of the **AQ** and **OPV** Block Molecules, Depending on the End Group

name^a^	end group	phase^b^	*d*^c^ (nm)	*T*_Endo_ (°C)^d^	Δ*H*_Endo_ (kJ mol^–1^)^d^	*T*_Exo_ (°C)^d^	Δ*H*_Exo_ (kJ mol^–1^)^d^
AQ-Si_15_	**PentO**	Lam	5.9	32 ± 1	23	20 ± 2	23
	**MeO**	Col_h_	5.6	23 ± 2	16	14 ± 2	18
	**H**	Col_h_	5.7	–18 ± 3	10	–21 ± 3	9
AQ-Si_32_-AQ	**PentO**	Lam	6.3	38 ± 2	44	25 ± 1	37
	**MeO**	Col_h_	5.7	23 ± 5	16	14 ± 4	18
	**H**	Col_h_	5.9	–10 ± 1	19	–13 ± 2	19
OPV-Si_15_	**PentO**	Col_h_	5.2	107 ± 2	14	105 ± 1	14
		Col_obl_	*a* = 5.8	133 ± 2	1^e^^,^^g^	131 ± 2	1^e^^,^^g^
			*b* = 6.5				
			γ = 83°				
	**MeO**	Col_obl_	*a* = 5.7	110 ± 5	9	102 ± 1	16^f^
			*b* = 6.1			107 ± 1^f^	
			γ = 81°				
	**H**	Col_obl_	*a* = 5.5	108 ± 1	21	102 ± 1	22
			*b* = 6.1				
			γ = 82°				
OPV-Si_32_–OPV	**PentO**	Lam	7.0	107 ± 2	30	104 ± 1	35
		Lam	7.2	134	n.d.^g^	131 ± 2	2^e^
	**MeO**	Col_h_	6.5	123 ± 2	27	117 ± 2	24
	**H**	Col_h_	6.2	104 ± 3	18	95 ± 5	41

a Block molecules as depicted in Figure 1.

b Morphology of the nanostructure determined with SAXS below the temperature of the lowest exothermic peak. Lam = Lamellar, Col_h_ = Columnar hexagonal, Col_obl_ = Columnar oblique.

c Domain spacing (*d*) calculated using *d* = 2π/q*, with q* the principal scattering peak for Lam and Col_h_. Calculation of the lattice parameters of the Col_obl_ phases can be found in Tables S3–S5.

d Thermal transitions as determined by DSC from the second heating and cooling cycle using a heating and cooling rate of 10 K min^–1^. The endothermic transitions are attributed to melting of the material and the exothermic transitions are attributed to crystallization of the material unless otherwise specified.

e Order–disorder (when endothermic) or disorder–order (when exothermic) transition due to their small enthalpic contribution.^35^

f Two temperatures are given as two clear maxima could be distinguished, while a single enthalpy is given as the peaks were overlapping.

g Not determined as peak onset could not be resolved (Figures S16A and S20A).

For **AQ**, a clear reduction in the thermal transition temperatures was observed when varying the **AQ** unit from **PentO-**, to **MeO-** and finally **H-**terminated (Figures S5–S7A). On the contrary, this effect was not found in the **OPV** series. We rationalize this difference through the size of the molecules, as changing the terminus on the **AQ** core results in a larger relative difference in length compared to the **OPV** core. A pronounced difference between **AQ** and **OPV** was also observed in their respective nanostructures upon decoration with shorter pendants. The **MeO-AQ** and **H-AQ** block molecules phase separated into a Col_h_ phase instead of the Lam phase found for **AQ-Si**_**15**_ and **AQ-Si**_**32**_**-AQ**. To explain this difference, we accounted for the decreased cohesive van der Waals interactions in the alkane region when assuming an alternating, lamellar packing as schematically shown in Figure 4E.^36^ Here, the decreased filling of the void space around the **AQ** cores is expected to result in decreased stability of the interdigitated packing.

The opposite was found in the **OPV** block molecules, where columnar nanostructures were found regardless of the terminal group (Figure 4B/D). Furthermore, all telechelic **OPV** molecules preferentially phase segregated into a Col_h_ phase, while only **OPV-Si**_**32**_**-OPV** formed into a lamellar phase. This finding underlines the aforementioned importance of chain-end effects in telechelic **OPV**-block molecules as this diminished the shape anisotropy which results in liquid crystalline-like behavior. Concluding, through the combined effects of space filling effects and shape anisotropy, lamellar morphologies are obtained most reliably when symmetric cores are employed.

## Conclusions

We have studied the influence of molecular design on the spontaneous phase separation of block molecules consisting of aromatic cores functionalized with amorphous oligodimethylsiloxane (*o*DMS). Hereto, both the molecular architecture, as well as the pendant chains on two aromatic cores (**AQ** and **OPV**) were varied. This systematic study showed that lamellar morphologies are consistently obtained when symmetric cores are used in a telechelic architecture. The higher propensity to form lamellae in telechelic architectures was assigned to the interconnection of the crystalline layers as well as decreased overcrowding of adjacent side chains. It was found that functionalization of the aromatic cores with shorter pendants results in the formation of columnar morphologies, as the decreased cohesive van der Waals interactions between alkyl chains destabilize this interdigitated packing. In summary, we identified that telechelic molecular designs with symmetric blocks result most reliably in lamellar morphologies. These results were used to develop the recently reported Crystal Lattice Analysis^19^ and as such help to better understand the design requirements for 2D nanostructures in phase-separated short oligomers and as such aid the development of nanostructured materials for lithography and nanoscale optoelectronics.

## Supporting Information Available

Additional experimental details, materials, and methods as well as MAXS/WAXS patterns, DSC spectra and POM images of all compounds (PDF)

## References

[ref1] Whitesides G. M.; Grzybowski B. Self-Assembly at All Scales. Science. 2002, 295 (5564), 2418–2421. 10.1126/science.1070821. 11923529

[ref2] van Genabeek B.; Lamers B. A. G.; Hawker C. J.; Meijer E. W.; Gutekunst W. R.; Schmidt B. V. K. J. Properties and Applications of Precision Oligomer Materials; Where Organic and Polymer Chemistry Join Forces. J. Polym. Sci. 2021, 59 (5), 373–403. 10.1002/pol.20200862.

[ref3] Wyers D.; Jirapanjawat T.; Quinn J. F.; Whittaker M. R.; Greening C.; Junkers T. Narrowing down Chain Length Effects on the Antibacterial Action of Guanylated Oligomers. Polym. Chem. 2023, 14 (17), 2126–2134. 10.1039/D3PY00203A.

[ref4] Ahner J.; Pretzel D.; Enke M.; Geitner R.; Zechel S.; Popp J.; Schubert U. S.; Hager M. D. Conjugated Oligomers as Fluorescence Marker for the Determination of the Self-Healing Efficiency in Mussel-Inspired Polymers. Chem. Mater. 2018, 30 (8), 2791–2799. 10.1021/acs.chemmater.8b00623.

[ref5] Chen J.; Rizvi A.; Patterson J. P.; Hawker C. J. Discrete Libraries of Amphiphilic Poly(Ethylene Glycol) Graft Copolymers: Synthesis, Assembly, and Bioactivity. J. Am. Chem. Soc. 2022, 144 (42), 19466–19474. 10.1021/jacs.2c07859. 36240519

[ref6] Isono T.; Komaki R.; Lee C.; Kawakami N.; Ree B. J.; Watanabe K.; Yoshida K.; Mamiya H.; Yamamoto T.; Borsali R.; Tajima K.; Satoh T. Rapid Access to Discrete and Monodisperse Block Co-Oligomers from Sugar and Terpenoid toward Ultrasmall Periodic Nanostructures. Commun. Chem. 2020, 3, 135 10.1038/s42004-020-00385-y. 36703322 PMC9814839

[ref7] Yang W.; Zhang W.; Luo L.; Lyu X.; Xiao A.; Shen Z.; Fan X. H. Ordered Structures and Sub-5 Nm Line Patterns from Rod–Coil Hybrids Containing Oligo(Dimethylsiloxane). Chem. Commun. 2020, 56 (71), 10341–10344. 10.1039/D0CC04377J. 32760981

[ref8] Israelachvili J. N.; Mitchell D. J.; Ninham B. W. Theory of Self-Assembly of Hydrocarbon Amphiphiles into Micelles and Bilayers. J. Chem. Soc., Faraday Trans. 1976, 2 (72), 1525–1568. 10.1039/f29767201525.

[ref9] Tschierske C. Non-Conventional Soft Matter. Annu. Rep. Prog. Chem., Sect. C: Phys. Chem. 2001, 97, 191–267. 10.1039/b101114f.

[ref10] Tschierske C. Liquid Crystal Engineering–New Complex Mesophase Structures and Their Relations to Polymer Morphologies, Nanoscale Patterning and Crystal Engineering. Chem. Soc. Rev. 2007, 36 (12), 1930–1970. 10.1039/b615517k. 17982518

[ref11] Bates C. M.; Bates F. S. 50th Anniversary Perspective: Block Polymers—Pure Potential. Macromolecules 2017, 50 (1), 3–22. 10.1021/acs.macromol.6b02355.

[ref12] Bates F. S. Polymer-Polymer Phase Behavior. Science 1991, 4996 (251), 898–905. 10.1126/science.251.4996.898. 17847383

[ref13] Lamers B. A. G.; Waal B. F. M.; de Meijer E. W. The Iterative Synthesis of Discrete Dimethylsiloxane Oligomers: A Practical Guide. J. Polym. Sci. 2021, 59 (12), 1142–1150. 10.1002/pol.20200649.

[ref14] Van Son M. H. C.; Berghuis A. M.; De Waal B. F. M.; Wenzel F. A.; Kreger K.; Schmidt H.-W.; Rivas J. G.; Vantomme G.; Meijer E. W. Highly Ordered Supramolecular Materials of Phase-Separated Block Molecules for Long-Range Exciton Transport. Adv. Mater. 2023, 35 (25), 2300891 10.1002/adma.202300891. 37002556

[ref15] Yang W.; Liu D.; Liu Y.; Yang S.; Liu Y.; Shen Z.; Yang H.; Fan X. H.; Zhou Q. F. Large-Area Uniaxially Oriented Sub-5 Nm Line Patterns of Hybrid Liquid Crystals Constructed by Perylene Diimide and Oligo(Dimethylsiloxane). Chem.—A Eur. J. 2023, 29 (18), e202203702 10.1002/chem.202203702. 36656133

[ref16] Lamers B. A. G.; Graf R.; De Waal B. F. M.; Vantomme G.; Palmans A. R. A.; Meijer E. W. Polymorphism in the Assembly of Phase-Segregated Block Molecules: Pathway Control to 1D and 2D Nanostructures. J. Am. Chem. Soc. 2019, 141 (38), 15456–15463. 10.1021/jacs.9b08733. 31483637 PMC6876923

[ref17] Cadeddu S.; van den Bersselaar B. W. L.; de Waal B.; Cordier M.; Vanthuyne N.; Meskers S. C. J.; Vantomme G.; Crassous J. Engineering Circularly Polarized Light Emission in Nanostructured Oligodimethylsiloxane-Helicene Chiral Materials. Nanoscale 2024, 16, 21351 10.1039/D4NR03389B. 39474743

[ref18] van den Bersselaar B. W. L.; van de Ven A. P. A.; de Waal B. F. M.; Meskers S. C. J.; Eisenreich F.; Vantomme G. Stimuli-Responsive Nanostructured Viologen-Siloxane Materials for Controllable Conductivity. Adv. Mater. 2024, 36 (23), 2312791 10.1002/adma.202312791. 38413048

[ref19] Schnitzer T.; van den Bersselaar B. W. L.; Lamers B. A. G.; van Son M. H. C.; Maessen S. J. D.; de Graaf F. V.; de Waal B. F. M.; Trapp N.; Vantomme G.; Meijer E. W. Crystal Lattice Analysis for 2D Nanomorphology Prediction of Phase-Separated Materials. J. Am. Chem. Soc. 2025, 11, 31 10.1021/jacs.4c14964. PMC11744751 39757471

[ref20] Galigné J. L. Etude Cristallographique de Composés Nématogènes. II. Structure Cristalline Du 4,4’-Azodiphénétole. Acta Crystallogr. 1970, 26 (12), 1977–1984. 10.1107/S0567740870005289.

[ref21] Adams J. M.; Ramdas S. The Crystal Structure of Solution-Grown 9,10-Diphenylanthracene. A Combined Computational and X-Ray Study. Acta Crystallogr. 1979, 35 (3), 679–683. 10.1107/S0567740879004428.

[ref22] Lonsdale K.; Milledge H. J.; El Sayed K. The Crystal Structure (at Five Temperatures) and Anisotropic Thermal Expansion of Anthraquinone. Acta Crystallogr. 1966, 20 (1), 1–13. 10.1107/S0365110X6600001X.

[ref23] Xu Y.; Wang K.; Zhang Y.; Xie Z.; Zou B.; Ma Y. Fluorescence Mutation and Structural Evolution of a π-Conjugated Molecular Crystal during Phase Transition. J. Mater. Chem. C 2016, 4 (6), 1257–1262. 10.1039/C5TC03745J.

[ref24] Cooper C. B.; Bao Z. Using Periodic Dynamic Polymers to Form Supramolecular Nanostructures. Accounts Mater. Res. 2022, 3 (9), 948–959. 10.1021/accountsmr.2c00101.

[ref25] Van Genabeek B.; De Waal B. F. M.; Gosens M. M. J.; Pitet L. M.; Palmans A. R. A.; Meijer E. W. Synthesis and Self-Assembly of Discrete Dimethylsiloxane-Lactic Acid Diblock Co-Oligomers: The Dononacontamer and Its Shorter Homologues. J. Am. Chem. Soc. 2016, 138 (12), 4210–4218. 10.1021/jacs.6b00629. 26999049

[ref26] Zha R. H.; Vantomme G.; Berrocal J. A.; Gosens R.; De Waal B.; Meskers S.; Meijer E. W. Photoswitchable Nanomaterials Based on Hierarchically Organized Siloxane Oligomers. Adv. Funct. Mater. 2018, 28 (1), 1703952 10.1002/adfm.201703952.

[ref27] Godbert N.; Crispini A.; Ghedini M.; Carini M.; Chiaravalloti F.; Ferrise A. LCDiXRay: A User-Friendly Program for Powder Diffraction Indexing of Columnar Liquid Crystals. J. Appl. Crystallogr. 2014, 47 (2), 668–679. 10.1107/S1600576714003240.

[ref28] Tosaka M.; Tashiro K. Crystal Polymorphism and Structure Models of Poly(Dimethylsiloxane). Polymer 2018, 153 (6), 507–520. 10.1016/j.polymer.2018.08.049.

[ref29] Kamatham N.; Ibraikulov O. A.; Durand P.; Wang J.; Boyron O.; Heinrich B.; Heiser T.; Lévêque P.; Leclerc N.; Méry S. On the Impact of Linear Siloxanated Side Chains on the Molecular Self-Assembling and Charge Transport Properties of Conjugated Polymers. Adv. Funct. Mater. 2021, 31 (6), 2007734 10.1002/adfm.202007734.

[ref30] Salzillo T.; Della Valle R. G.; Venuti E.; Brillante A.; Siegrist T.; Masino M.; Mezzadri F.; Girlando A. Two New Polymorphs of the Organic Semiconductor 9,10-Diphenylanthracene: Raman and X-Ray Analysis. J. Phys. Chem. C 2016, 120 (3), 1831–1840. 10.1021/acs.jpcc.5b11115.

[ref31] Ohta A.; Hattori K.; Kusumoto Y.; Kawase T.; Kobayashi T.; Naito H.; Kitamura C. Effects of Alkoxy Substitution on the Optical Properties of 9,10-Anthraquinone and Anthracene: 2,3,6,7-Tetrapropoxy-Substituted vs. 2,6-Dipropoxy-Substituted Derivatives. Chem. Lett. 2012, 41 (7), 674–676. 10.1246/cl.2012.674.

[ref32] Norikane Y.; Uchida E.; Tanaka S.; Fujiwara K.; Koyama E.; Azumi R.; Akiyama H.; Kihara H.; Yoshida M. Photoinduced Crystal-to-Liquid Phase Transitions of Azobenzene Derivatives and Their Application in Photolithography Processes through a Solid-Liquid Patterning. Org. Lett. 2014, 16 (19), 5012–5015. 10.1021/ol502223u. 25216186

[ref33] Varghese S.; Yoon S. J.; Casado S.; Fischer R. C.; Wannemacher R.; Park S. Y.; Gierschner J. Orthogonal Resonator Modes and Low Lasing Threshold in Highly Emissive Distyrylbenzene-Based Molecular Crystals. Adv. Opt. Mater. 2014, 2 (6), 542–548. 10.1002/adom.201300512.

[ref34] van Son M. H. C.; Berghuis A. M.; Eisenreich F.; de Waal B.; Vantomme G.; Rivas J. G.; Meijer E. W. Highly Ordered 2D-Assemblies of Phase-Segregated Block Molecules for Upconverted Linearly Polarized Emission. Adv. Mater. 2020, 32 (48), 2004775 10.1002/adma.202004775. 33118197

[ref35] Bruce D. W.; Deschenaux R.; Donnio B.; Guillon D. 12.05–Metallomesogens. Compr. Organomet. Chem. 2007, III, 195–293. 10.1016/B0-08-045047-4/00170-9.

[ref36] Goodby J. W.; Mandle R. J.; Davis E. J.; Zhong T.; Cowling S. J. What Makes a Liquid Crystal? The Effect of Free Volume on Soft Matter. Liq. Cryst. 2015, 42 (5–6), 593–622. 10.1080/02678292.2015.1030348.

[ref37] Cimrová V.; Remmers M.; Neher D.; Wegner G. Polarized Light Emission from LEDs Prepared by the Langmuir-Blodgett Technique. Adv. Mater. 1996, 8 (2), 146–149. 10.1002/adma.19960080209.

[ref38] Bao Z.; Chen Y.; Cai R.; Yu L. Conjugated Liquid Crystalline Polymers—Soluble and Fusible Poly(Phenylenevinylene) by the Heck Coupling Reaction. Macromolecules 1993, 26 (20), 5281–5286. 10.1021/ma00072a002.

[ref39] Zha R. H.; de Waal B.; Lutz M.; Teunissen A. J. P.; Meijer E. W. End Groups of Functionalized Siloxane Oligomers Direct Block Copolymeric or Liquid Crystalline Self-Assembly Behavior. J. Am. Chem. Soc. 2016, 138, 5693 10.1021/jacs.6b02172. 27054381 PMC4858755

