# Supplementary material for: Targeting 2D Nanostructures in Phase-Separated Materials through Molecular Design
Source: Macromolecules. 2025 Mar 6;58(6):2917–24. doi: 10.1021/acs.macromol.4c02691 (PMC11948466; doi:10.1021/acs.macromol.4c02691)
Supplement: Supplementary file 1 — ma4c02691_si_001.pdf [file ma4c02691_si_001.pdf]

Supporting Information

Targeting 2D nanostructures in phase-separated materials through molecular design

Martin H.C. van Son,[†,a] Bart W.L. van den Bersselaar,[†,a] Bas F. M. de Waal,[a] Ghislaine Vantomme[a] and E.W. Meijer[,a]*

[†]These authors contributed equally to this work.

[a]Laboratory of Macromolecular and Organic Chemistry and Institute for Complex Molecular Systems, Eindhoven University of Technology P.O. Box 513, 5600MB Eindhoven, The Netherlands

[*]Corresponding author: e.w.meijer@tue.nl

Table of Contents

1. Materials and Methods 2
2. Synthetic procedures 4
  2.1 DPA derivatives 4
  2.2 AQ Derivatives 7
  2.3 Azo derivatives 14
  2.4 OPV derivatives 18
3. Block molecule bulk characterization 27
  3.1 DPA derivatives 27
  3.2 AQ derivatives 30
  3.3 Azo derivatives 38
  3.4 OPV derivatives 42
4. NMR Spectra 53
  4.1 DPA derivatives 53
  4.2 AQ derivatives 57
  4.3 Azo derivatives 71
  4.4 OPV derivatives 77
5. References 93

1. Materials and Methods

All chemicals were purchased from commercial sources and used without further purification. The discrete length oligodimethylsiloxanes (*o*DMS) monohydride with a length of 7 (CH_3Si_7H) or 15 ($CH_3Si_{15}H$) silicon atoms, and *o*DMS dihydride with a length of 32 silicon atoms ($HSi_{32}H$) were synthesized according to literature procedure.[1] Dry solvents were obtained with an MBRAUN solvent purification system (MB-SPS). Oven-dried round bottom flasks (120 °C) or flame-dried Schlenk flasks were used for all reactions carried out under argon atmosphere. Reactions were followed by thin-layer chromatography (TLC) using 60-F254 silica gel plates from Merck and visualized by UV light at 254 nm. Automated column chromatography was conducted on a Grace system using Büchi FlashPure Silica Cartridges.

Recycling Gel Permeation Chromatography (recGPC) was performed in stabilized tetrahydrofuran solution with a Shodex-GPC H-2002.5 column (20 mm internal diameter × 500 mm length, MW up to 20.000 calibrated to PS standards). A Shimadzu SPD-10AV VP was used as UV-Vis detector in conjunction with DAX 9.0 software from Van Mierlo Software Consultancy.

Nuclear magnetic resonance (NMR) spectra were recorded on Bruker 400 MHz Ultrashield spectrometers (400 MHz for ^1H NMR, 100 MHz for ^{13}C NMR). Deuterated chloroform is used in each case with its shifts used as internal standard (7.26 ppm for ^1H NMR; 77.16 ppm for ^{13}C NMR). Chemical shifts (δ) are expressed in ppm values relative to tetramethylsilane. Peak multiplicity is abbreviated as s: singlet; d: doublet; t: triplet; q: quartet; p: pentet; m: multiplet.

Matrix assisted laser desorption/ionization time of flight mass spectroscopy (MALDI-TOF-MS) spectra were acquired using a Bruker Autoflex Speed MALDI-TOF using γ-cyano-4-hydroxycinnamic acid (CHCA) or trans-2-[3-(4-tert-butylphenyl)-2-methyl-2-propenylidene]malononitrile (DCTB) as matrices. All samples were dissolved in chloroform.

Differential scanning calorimetry (DSC) data were collected on a DSC Q2000 from TA instruments, calibrated with an indium standard. The samples (4-8 mg) were weighed directly into aluminium pans and hermetically sealed. The samples were initially heated to 180 °C and then subjected to two cooling/heating cycles from −70 °C to 180 °C with a rate of 10 K min^{-1}. The data that is presented, represents the second heating and/or cooling cycle, with the third cycle similar to the second. The thermal transition temperatures were assigned at the maximum of the transition.

Polarized Optical Microscopy (POM) samples were sandwiched between glass substrates and imaged using Nikon ECLIPSE Ci-POL microscope with 5x, or 20x magnification. The samples were heated and cooled using a Linkam LTS 420 heating stage mounted in the POM set-up. All samples were heated to their isotropic phase (10-20 °C above

their melting point, isothermal for 5-10 minutes) and subsequently cooled with 5 °C min^{-1} to their melting point. The samples were further cooled with 1 K min^{-1} to form birefringent domains. Temperatures below room temperature were reached through controlled cooling with liquid nitrogen.

Bulk Medium/Wide angle X-ray scattering (MAXS/WAXS) was performed on a Ganesha instrument from SAXSLAB. The flight tube and sample holder are all under vacuum in a single housing, with a high brilliance Microfocus Cu Source (Xenocs Genix3D). The source produces X-rays with a wavelength (λ) of 0.154 nm and flux of 1×108 ph s^{-1}. Scattered X-rays were captured on a 2-dimensional Pilatus 300K detector with 487×619 pixel resolution. The sample-to-detector distance was 0.084 m (WAXS mode) or 0.48 m (MAXS mode). The measurement time was 300 seconds (WAXS mode) or 1200 seconds (MAXS mode). The instrument was calibrated with diffraction patterns from silver behenate. Bulk X-ray scattering was performed on samples in a 1 mm glass capillary. All room temperature measurements were performed after overnight cooling from their respective isotropic melt. Domain spacings (d) are calculated using $d = 2\pi/q^*$, with q^* the principal scattering peak.

Volume fractions were estimated as the ratio between the *o*DMS volume and the core volume. For this, the bulk density of PDMS was taken as 0.95 g ml^{-1} which was converted to volume with the respective molecular weights of the three lengths *o*DMS ($M_{w,CH3Si7H}$ = 519.1 g mol^{-1} gives $V_{CH3Si7H}$ ≈ 546 ml mol^{-1}; $M_{w,CH3Si15H}$ = 1112.4 g mol^{-1} gives $V_{CH3Si15H}$ ≈ 1170 ml mol^{-1}; $M_{w,HSi32H}$ = 2359 g mol^{-1} gives V_{HSi32H} ≈ 2483 ml mol^{-1}).[2] The volume of the cores were calculated using the densities of the crystal structures of compounds similar to used cores. The crystal densities were estimated from the crystal structures of unfunctionalized 9,10-diphenylanthracene,[3] C_3AQC_3,[4] C_6AzoC_6,[5] and C_4OPVC_4[6] by dividing the unit cell volume in Å^3 with the number of molecules in the unit cell followed by conversion to ml mol^{-1}. For the calculations for the volume of the cores, their molecular weights were taken including alkyl spacer on both sides. The calculated volume fractions thus give an upper limit of the core volume fractions.

2. Synthetic procedures

2.1 DPA derivatives

Si_7-DPA-Si_7 was synthesized according to literature procedure.[7]

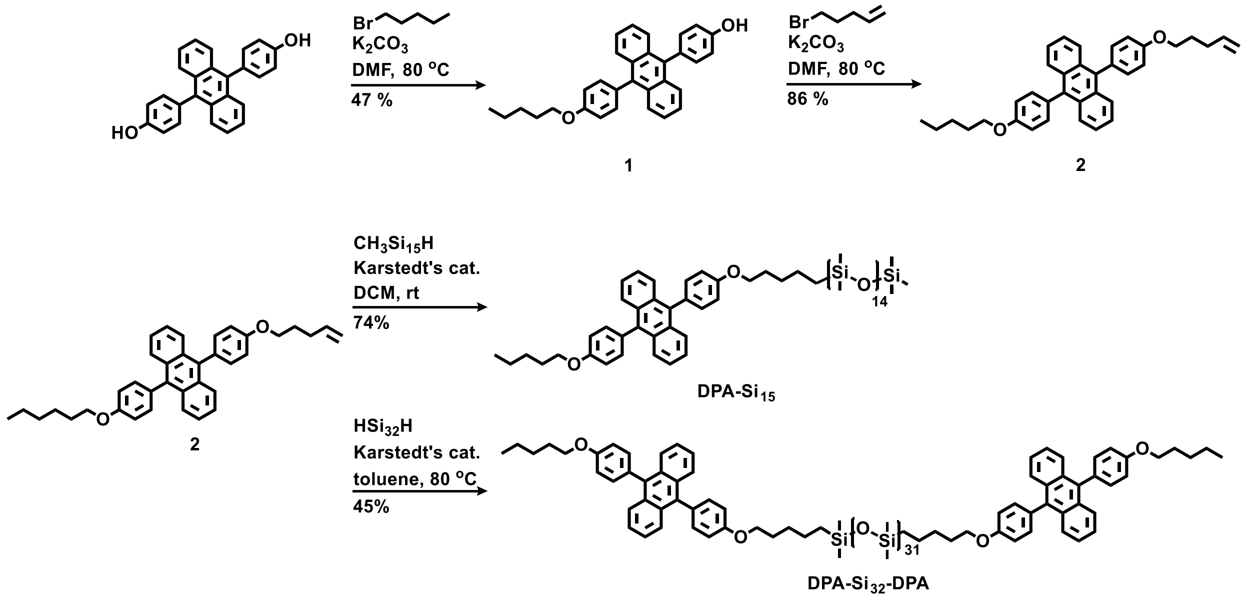

***Scheme 1**: Synthetic pathway to DPA derivatives.*

4-(10-(4-(pentyloxy)phenyl)anthracen-9-yl)phenol (1)

An oven-dried round bottom flask was charged with 4,4'-(9,10-anthracenediyl)bis-phenol (811.6 mg, 2.24 mmol), 1-bromopentane (166.7 mg, 1.12 mmol) and K_2CO_3 (454.0 mg, 3.28 mmol) in dry DMF (50 mL). This mixture was stirred at 80 °C under argon atmosphere for 18 hours. Consequently, the mixture was cooled to room temperature and precipitated in cold 1M HCl (500 mL). The precipitate was filtered twice with a Büchner funnel over the same filter paper. The precipitate was purified using column chromatography (Solid loaded, SNAP-Kil 40g, eluent: Heptane, 100%) to yield the target compound as slightly yellow crystals (224.8 mg, 0.526 mmol, 47%).

^{1}H NMR (400 MHz, $CDCl_3$) δ 7.75 (m, 4H, Ar), 7.41 – 7.29 (m, 8H, Ar), 7.13 (d, J = 8.4 Hz, 2H, ArO), 7.11 – 7.04 (m, 2H, ArO), 5.04 (s, 1H, DPAO$\underline{H}$), 4.12 (t, J = 6.5 Hz, 2H, DPAOC$\underline{H}_2$), 1.91 (p, J = 6.7 Hz, 2H, DPAOCH$_2$C$\underline{H}_2$), 1.64 – 1.42 (m, 4H, C$\underline{H}_2$C$\underline{H}_2$CH$_3$), 0.99 (t, J = 7.1 Hz, 3H, C$\underline{H}_3$). ^{13}C NMR was not recorded due to poor solubility.

9-(4-(pent-4-en-1-yloxy)phenyl)-10-(4-(pentyloxy)phenyl)anthracene (2)

An oven-dried round bottom flask was charged with **1** (140.0 mg, 0.320 mmol), 5-bromo-1-pentene (271.0 mg, 1.82 mmol) and K_2CO_3 (144.0 mg, 1.04 mmol) in dry DMF (50 mL). This mixture was stirred at 80 °C under argon atmosphere for 18 hours. Consequently, the mixture

was cooled to room temperature and precipitated in cold DI water (500 mL) to yield the target compound as white crystals (Yield = 140 mg, 0.275 mmol, 86%).

^{1}H NMR (400 MHz, $CDCl_3$) δ 7.79 – 7.73 (m, 4H, Ar), 7.41 – 7.30 (m, 8H, Ar), 7.17 – 7.11 (m, 4H, ArO), 5.94 (m, 1H, $C\underline{H}=CH_2$), 5.21 – 5.02 (m, 2H, $CH=C\underline{H}_2$), 4.13 (q, *J* = 6.9 Hz, 4H, $DPAOC\underline{H}_2$), 2.35 (q, *J* = 7.7, 7.2 Hz, 2H, $C\underline{H}_2CH=CH_2$), 2.01 (m, 2H, $DPAOCH_2C\underline{H}_2$), 1.96 – 1.86 (m, 2H, $DPAOCH_2C\underline{H}_2$), 1.60 – 1.40 (m, 4H, $C\underline{H}_2C\underline{H}_2CH_3$), 1.00 (m, 3H, $C\underline{H}_3$). ^{13}C NMR was not recorded due to poor solubility.

DPA-Si_{15}

A flame dried Schlenk flask was charged with **2** (29.4 mg, 0.06 mmol), $CH_3Si_{15}H$ (133.6 mg, 0.12 mmol) and dry DCM (1.5 mL). Subsequently, 1 drop Karstedt's catalyst was added and the mixture was stirred under argon at room temperature. After 5 hours of reaction, another drop of catalyst was added. The mixture was left to stir over the weekend after which the mixture was purified by column chromatography (Solid load, Büchi FlashPure 40g, eluent: Heptane/$CHCl_3$ 0/100 – 100/0) to yield the target compound as a waxy solid (Yield: 70.0 mg, 0.044 mmol, 74%).

^{1}H NMR (400 MHz, $CDCl_3$) δ 7.76 (m, 4H, Ar), 7.38 (d, *J* = 8.5 Hz, 4H, Ar), 7.33 (m, 4H, Ar), 7.13 (d, *J* = 8.5 Hz, 4H, Ar), 4.12 (t, *J* = 6.6 Hz, 4H, 2x $DPAOC\underline{H}_2$), 1.95 – 1.87 (m, 4H, 2x $DPAOCH_2C\underline{H}_2$), 1.64 – 1.42 (m, 8H, H_2O, 2x $C\underline{H}_2C\underline{H}_2$), 1.00 (t, *J* = 7.1 Hz, 3H, $C\underline{H}_3$), 0.68 – 0.61 (m, 2H, $C\underline{H}_2Si$), 0.14 – 0.07 (m, 90H, *o*DMS). ^{13}C NMR (100 MHz, $CDCl_3$) δ 158.76, 136.97, 132.51, 131.08, 131.07, 130.43, 127.23, 124.98, 114.53, 77.48, 77.16, 76.85, 68.27, 32.05, 30.04, 29.36, 29.29, 29.19, 28.50, 23.33, 22.85, 22.71, 18.43, 14.27, 14.24, 1.94, 1.58, 1.37, 1.30, 1.26, 1.23, 1.21, 0.84, 0.39, 0.15. MALDI-ToF-MS M_w calculated for $C_{67}H_{130}O_{16}Si_{15}$: 1610.60 g/mol, m/z found = 1612.56 $[M+H]^+$.

DPA-Si_{32}-DPA

A flame dried Schlenk flask was charged with **2** (69.2 mg, 0.14 mmol), $HSi_{32}H$ (110.1 mg, 0.05 mmol) and dry toluene (2.5 mL). The mixture was heated to 100 °C and subsequently, 1 drop Karstedt's catalyst was added and the mixture was stirred under argon at room temperature for 18 hours. The mixture was cooled to room temperature, after which the mixture was purified by column chromatography (Solid load, Büchi FlashPure 40g, eluent: Heptane/$CHCl_3$ 0/100 – 100/0) to yield the target compound as a yellowish wax (70.0 mg, 0.022 mmol, 45%).

^{1}H NMR (400 MHz, $CDCl_3$) δ 7.78 – 7.72 (m, 8H, Ar), 7.40 – 7.35 (m, 8H, Ar), 7.35 – 7.29 (m, 8H, Ar), 7.16 – 7.10 (m, 8H, Ar), 4.11 (m, 8H, 4x $DPAOC\underline{H}_2$), 1.95 – 1.86 (m, 8H, 4x $DPAOCH_2C\underline{H}_2$), 1.64 – 1.41 (m, 16H, H_2O, 4x $C\underline{H}_2C\underline{H}_2$), 0.99 (t, *J* = 7.2 Hz, 6H, 2x $C\underline{H}_3$), 0.67 – 0.60 (m, 4H, 2x $C\underline{H}_2Si$), 0.15 – 0.02 (m, 192H, *o*DMS). ^{13}C NMR (100 MHz, $CDCl_3$) δ 158.76,

136.97, 132.51, 131.08, 131.07, 130.43, 127.23, 124.98, 114.53, 77.48, 77.16, 76.84, 68.27, 32.05, 30.05, 29.36, 29.29, 29.19, 28.50, 23.33, 22.86, 22.71, 18.43, 14.27, 14.24, 1.56, 1.36, 1.25, 1.23, 1.19, 0.82, 0.38, 0.15. MALDI-ToF-MS M_w calculated for $C_{136}H_{266}O_{35}Si_{32}$: 3355.20g/mol, m/z found = 3359.12 $[M+H]^+$.

2.2 AQ Derivatives

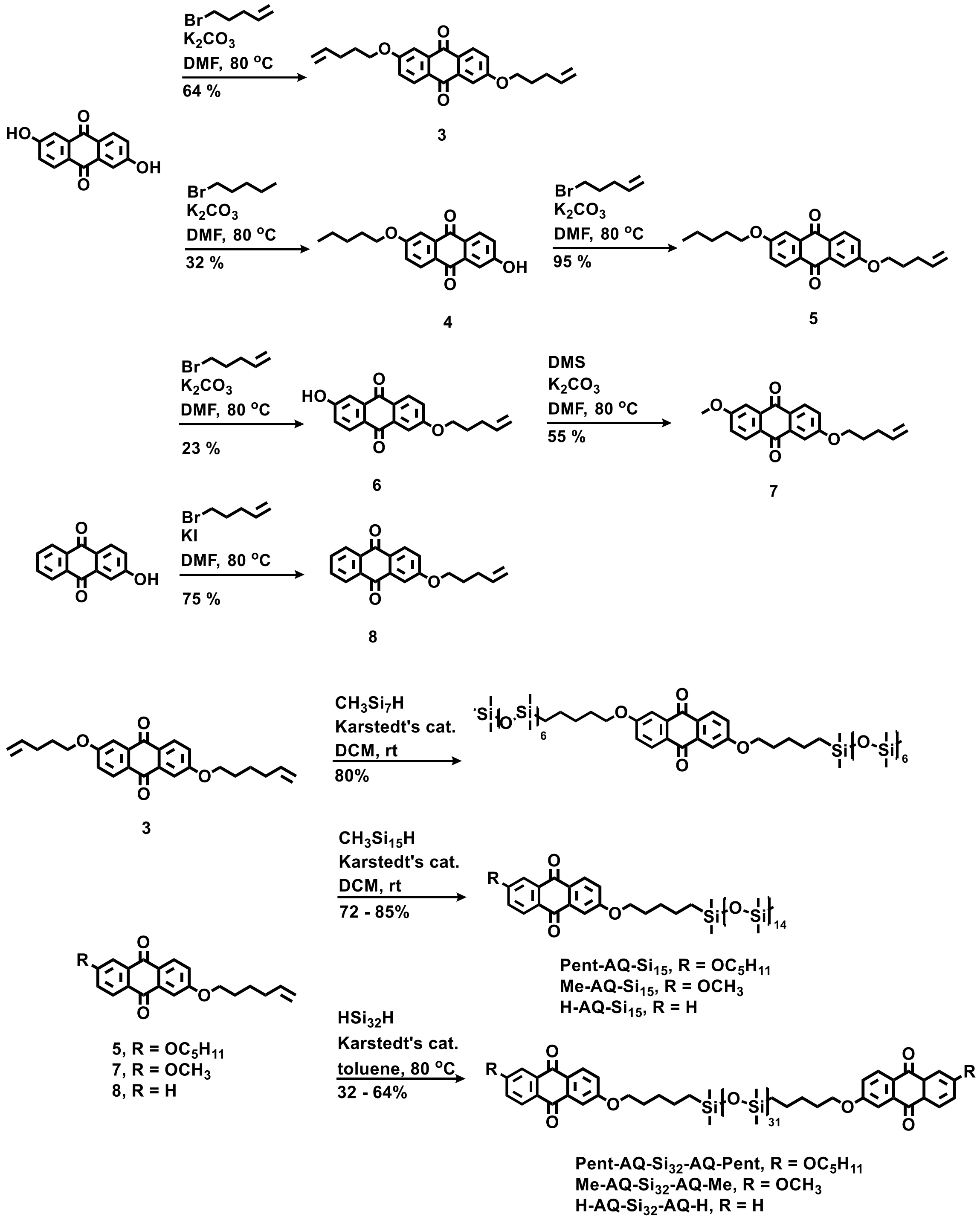

Scheme 2: *Synthetic pathway to AQ derivatives.*

2-(pent-4-en-1-yloxy)-6-(pent-4-en-1-yloxy)anthracene-9,10-dione (3)

An oven-dried round bottom flask was charged with 2,6-dihydroxyanthraquinone (123.4 mg, 0.51 mmol), 5-bromo-1-pentene (213.4 mg, 1.43 mmol) and K_2CO_3 (216.6 mg, 1.57 mmol) in dry DMF (6 mL). This mixture was stirred at 80 °C under argon atmosphere for 20 hours. Consequently, the mixture was cooled to room temperature and precipitated in cold DI water.

The precipitate was purified using column chromatography (Solid load, SNAP-Kil 40g, eluent: Heptane, 100%) to yield the target compound as slightly yellow fluffy crystals (123.0 mg, 0.326 mmol, 64%).

^{1}H NMR (400 MHz, $CDCl_3$) δ 8.23 (d, *J* = 8.7 Hz, 2H, Ar), 7.71 (d, *J* = 2.6 Hz, 2H, Ar), 7.22 (m, 2H, Ar), 5.86 (m, 2H, 2x $C\underline{H}=CH_2$), 5.17 – 4.95 (m, 4H, 2x $CH=C\underline{H}_2$), 4.16 (t, *J* = 6.4 Hz, 4H, 2x $AQOC\underline{H}_2$), 2.28 (q, *J* = 7.2 Hz, 4H, 2x $AQOCH_2C\underline{H}_2$), 1.96 (p, *J* = 6.7 Hz, 4H, 2x $AQOCH_2CH_2C\underline{H}_2$). ^{13}C NMR (100 MHz, $CDCl_3$) δ 182.30, 163.96, 137.40, 135.87, 129.68, 127.07, 120.91, 115.58, 110.57, 77.33, 77.01, 76.69, 67.93, 29.97, 28.18.

2-hydroxy-6-(pentyloxy)anthracene-9,10-dione (4)

An oven-dried round bottom flask was charged with 2,6-dihydroxyanthraquinone (1.640 g, 6.83 mmol), 1-bromopentane (0.591 g, 3.91 mmol) and K_2CO_3 (1.00 g, 7.24 mmol) in dry DMF (200 mL). This mixture was stirred at 100 °C under argon atmosphere for 18 hours. Consequently, the mixture was cooled to room temperature and precipitated in cold 1M HCl (500 mL). The precipitate was purified using column chromatography (Solid load, SNAP-Kil 40g, eluent: DCM, 100%) to yield the target compound as yellow crystals (388.0 mg, 1.251 mmol, 32%).

^{1}H NMR (400 MHz, $CDCl_3$) δ 8.22 (t, *J* = 8.4 Hz, 2H, AQ), 7.79 (d, *J* = 2.6 Hz, 1H, AQ), 7.71 (d, *J* = 2.7 Hz, 1H, AQ), 7.22 (m, 2H, AQ), 6.75 (s, 1H, $O\underline{H}$), 4.15 (t, *J* = 6.6 Hz, 2H, $AQOC\underline{H}_2$), 1.94 – 1.75 (m, 2H, $AQOCH_2C\underline{H}_2$), 1.55 – 1.35 (m, 4H, $AQOCH_2CH_2C\underline{H}_2C\underline{H}_2CH_3$), 0.95 (t, *J* = 7.1 Hz, 3H, $AQOCH_2CH_2CH_2CH_2C\underline{H}_3$). ^{13}C NMR was not recorded.

2-(pent-4-en-1-yloxy)-6-(pentyloxy)anthracene-9,10-dione (5)

An oven-dried round bottom flask was charged with **4** (388.0 mg, 1.25 mmol), 5-bromo-1-pentene (377.0 mg, 2.53 mmol) and K_2CO_3 (265.0 mg, 1.92 mmol) in dry DMF (50 mL). This mixture was stirred at 80 °C under argon atmosphere for 20 hours. Consequently, the mixture was cooled to room temperature and precipitated in cold 1M HCl (250 mL). The precipitate was purified using column chromatography (Solid load, SNAP-Kil 40g, eluent: Heptane, 100%) to yield the target compound as crashed out yellow crystals (450.0 mg, 1.19 mmol, 95%).

^{1}H NMR (400 MHz, $CDCl_3$) δ 8.22 (m, 2H, AQ), 7.70 (d, *J* = 2.6 Hz, 2H, AQ), 7.22 (m, 2H, AQ), 5.86 (m, 1H, $C\underline{H}=CH_2$), 5.14 – 4.98 (m, 2H, $CH=C\underline{H}_2$), 4.15 (q, *J* = 6.6 Hz, 4H, 2x $AQOC\underline{H}_2$), 2.34 – 2.20 (m, 2H, $C\underline{H}_2CH=CH_2$), 1.96 (p, *J* = 6.7 Hz, 2H, $AQOCH_2C\underline{H}_2$), 1.85 (p, *J* = 6.8 Hz, 2H, $C\underline{H}_2CH_2CH=CH_2$), 1.52 – 1.32 (m, 4H, $C\underline{H}_2C\underline{H}_2CH_3$), 0.95 (t, *J* = 7.0 Hz, 3H, $CH_2CH_2C\underline{H}_3$). ^{13}C NMR (100 MHz, $CDCl_3$) δ 182.32, 164.08, 163.95, 137.40, 135.88, 135.86, 129.67, 127.09, 126.99, 120.93, 120.90, 115.58, 110.57, 68.82, 67.93, 29.97, 28.73, 28.19, 28.10, 22.41, 14.00.

2-hydroxy-6-(pent-4-en-1-yloxy)anthracene-9,10-dione (6)

An oven-dried round bottom flask was charged with 2,6-dihydroxyanthraquinone (619.0 mg, 2.58 mmol), 5-bromo-1-pentene (197.0 mg, 0.51 mmol) and K_2CO_3 (414.0 mg, 3.0 mmol) in dry DMF (80 mL). This mixture was stirred at 80 °C under argon atmosphere for 18 hours. Consequently, the mixture was cooled to room temperature, filtered to remove the K_2CO_3 and washed with DCM, MeOH and Acetone. The filtrate was reduced *in vacuo* and dropwise added to 1M HCl (300 mL). The material was filtered and the residue was purified using column chromatography (solid load, SNAP-Kil 40g, eluent: Heptane/DCM 100/0 – 100/0, switch to *iso*-propanol/DCM 10/90) to yield the material as a yellow solid (Yield: 180 mg, 0.116 mmol, 23%).

^{1}H NMR (400 MHz, $CDCl_3$) δ 8.89 (s, 1H, $O\underline{H}$), 8.19 (m, 2H, AQ), 7.77 (d, *J* = 2.6 Hz, 1H, AQ), 7.69 (d, *J* = 2.6 Hz, 1H, AQ), 7.22 (m, 2H, AQ), 5.86 (m, 1H, $C\underline{H}=CH_2$), 5.13 – 4.98 (m, 2H, $CH=C\underline{H}_2$), 4.15 (t, *J* = 6.4 Hz, 2H, $AQOC\underline{H}_2$), 2.34 – 2.21 (m, 2H, $C\underline{H}_2CH=CH_2$), 1.95 (p, *J* = 6.7 Hz, 2H, $AQOCH_2C\underline{H}_2$). ^{13}C NMR (100 MHz, $CDCl_3$) δ 182.65, 182.27, 164.03, 162.97, 162.52, 137.40, 135.98, 135.90, 130.22, 129.65, 127.00, 126.58, 121.13, 120.86, 115.58, 113.22, 110.66, 67.94, 36.80, 31.76, 29.97, 28.17.

2-methoxy-6-(pent-4-en-1-yloxy)anthracene-9,10-dione (7)

An oven-dried round bottom flask was charged with **6** (180.0 mg, 0.584 mmol), dimethyl sulfide (0.2 mL, 2.34 mmol), K2CO3 (148.9 mg, 1.08 mmol) and acetone (40 mL). This mixture was stirred at 65 °C under argon atmosphere for 18 hours. Subsequently, the mixture was cooled to room temperature, filtered to remove K2CO3 and the material was purified using column chromatography (Solid load, SNAP-Kil 24g, eluent: Heptane/EtAc 100/0 – 80/20) to yield the product as crystals in the test tubes (103.9 mg, 0.321 mmol, 55%)

^{1}H NMR (400 MHz, $CDCl_3$) δ 8.17 (m, 2H, AQ), 7.64 (m, 2H, AQ), 7.17 (m, 2H, AQ), 5.85 (m, 1H, $C\underline{H}=CH_2$), 5.11 – 4.96 (m, 2H, $CH=C\underline{H}_2$), 4.12 (t, *J* = 6.4 Hz, 2H, $AQOC\underline{H}_2CH_2$), 3.95 (s, 3H, $C\underline{H}_3OAQ$), 2.33 – 2.18 (m, 2H, $C\underline{H}_2CH=CH_2$), 1.94 (m, 2H, $AQOCH_2C\underline{H}_2$). ^{13}C NMR (100 MHz, $CDCl_3$) δ 182.13, 182.07, 164.35, 163.89, 137.40, 135.80, 135.74, 129.63, 129.61, 128.31, 127.14, 126.97, 126.10, 120.85, 120.54, 115.57, 110.50, 109.92, 68.48, 67.91, 55.91, 32.27, 31.88, 29.97, 29.02, 28.17, 22.69, 18.05, 14.12.

2-(pent-4-en-1-yloxy)anthracene-9,10-dione (8)

An oven-dried round bottom flask was charged with 2-hydroxyanthraquinone (580.0 mg, 2.57 mmol), 5-bromo-1-pentene (770.0 mg, 5.13 mmol) and KI (43.0 mg, 0.26 mmol) in dry DMF (50 mL). This mixture was stirred at 80 °C under argon atmosphere for 20 hours. Consequently, the mixture was cooled to room temperature and precipitated in cold water (250 mL). The

precipitate was filtered and dried under vacuum overnight to yield the target compound as a dark green-brown solid (0.570 mg, 1.93 mmol, 75%).

^{1}H NMR (400 MHz, Chloroform-d) δ = 8.36 – 8.19 (m, 3H, AQ), 7.84 – 7.65 (m, 3H, AQ), 7.31 – 7.18 (m, 1H, AQ), 5.94 – 5.84 (m, 1H, $C\underline{H}=CH_2$), 5.10 – 5.04 (m, 2H, $CH=C\underline{H}_2$), 4.17 (t, *J* = 6.4 Hz, 2H, $AQOC\underline{H}_2$), 2.28 (q, *J* = 7.1 Hz, 2H, $C\underline{H}_2CH=CH_2$), 2.01 – 1.92 (m, 2H, $AQOCH_2C\underline{H}_2$). ^{13}C NMR (100 MHz, $CDCl_3$) δ = 182.65, 163.83, 137.37, 135.52, 135.12 – 133.22, 129.71, 121.40, 115.59, 110.50, 67.94, 29.97, 28.16.

Si_7-AQ-Si_7

A flame dried Schlenk flask was charged with **3** (55.3 mg, 0.15 mmol), CH_3Si_7H (231.8 mg, 0.45 mmol) and dry DCM (0.4 mL). Subsequently, 1 drop Karstedt's catalyst was added and the mixture was stirred under argon at room temperature. The mixture was left to stir until full conversion was observed by TLC, after which the mixture was purified by column chromatography (Solid load, Büchi FlashPure 40g, eluent: Heptane/$CHCl_3$ 0/100 – 100/0) to yield the target compound as yellowish liquid (168.0 mg, 0.120 mmol, 80%).

^{1}H NMR (400 MHz, $CDCl_3$) δ 8.22 (d, *J* = 8.6 Hz, 2H, AQ), 7.70 (d, *J* = 2.6 Hz, 2H, AQ), 7.21 (dd, *J* = 8.6, 2.7 Hz, 2H, AQ), 4.14 (t, *J* = 6.5 Hz, 4H, 2x $AQOC\underline{H}_2$), 1.85 (p, *J* = 6.8 Hz, 4H, 2x $AQOCH_2C\underline{H}_2$), 1.62 – 1.37 (m, 8H, $AQOCH_2CH_2C\underline{H}_2C\underline{H}_2$), 0.64 – 0.53 (m, 4H, $C\underline{H}_2SiO$), 0.14 – 0.01 (m, 90H, *o*DMS). ^{13}C NMR (100 MHz, $CDCl_3$) δ 182.45, 164.21, 136.02, 129.79, 127.16, 121.05, 110.68, 77.48, 77.16, 76.84, 68.97, 29.80, 28.97, 23.22, 18.36, 1.94, 1.34, 1.30, 1.24, 1.22, 0.35. MALDI-ToF-MS M_w calculated for $C_{54}H_{116}O_{16}Si_{14}$: 1412.50 g/mol, m/z found = 1437.48 $[M+Na]^+$.

Pent-AQ-Si_{15}

A flame dried Schlenk flask was charged with **5** (51.3 mg, 0.140 mmol), $CH_3Si_{15}H$ (225.6 mg, 0.200 mmol) and dry DCM (1 mL). Subsequently, 2 drops Karstedt's catalyst were added and the mixture was stirred under argon at room temperature. The mixture was left to stir for 20 hours, after which the mixture was purified by column chromatography (Solid load, Büchi FlashPure 40g, eluent: Heptane/$CHCl_3$ 0/100 – 60/40) to yield the target compound as yellowish wax (145.0 mg, 0.100 mmol, 72%).

^{1}H NMR (400 MHz, $CDCl_3$) δ 8.22 (d, *J* = 8.6 Hz, 2H, AQ), 7.70 (d, *J* = 2.6 Hz, 2H, AQ), 7.21 (m, 2H, AQ), 4.14 (m, 4H, 4x $AQOC\underline{H}_2$), 1.92 – 1.76 (m, 4H, $AQOCH_2C\underline{H}_2$), 1.58 – 1.35 (m, 8H, $AQOCH_2CH_2C\underline{H}_2C\underline{H}_2$), 0.95 (t, *J* = 7.1 Hz, 3H, $C\underline{H}_3CH_2CH2$), 0.66 – 0.54 (m, 2H, $C\underline{H}_2SiO$), 0.08 (m, 90H, *o*DMS). ^{13}C NMR (100 MHz, $CDCl_3$) δ 182.47, 182.45, 164.20, 136.00, 129.79, 127.14, 121.05, 110.67, 68.95, 29.80, 28.96, 28.87, 28.24, 23.21, 22.56, 18.34, 14.15, 1.93,

1.34, 1.29, 1.23, 1.20, 0.34. MALDI-ToF-MS M_w calculated for $C_{55}H_{120}O_{18}Si_{15}$: 1488.50 g/mol, m/z found = 1513.44 $[M+Na]^+$.

Pent-AQ-Si$_{32}$-AQ-Pent

A flame dried Schlenk flask was charged with **5** (49.5 mg, 0.130 mmol), $HSi_{32}H$ (125.0 mg, 0.050 mmol) and dry toluene (1 mL). Subsequently, 2 drops Karstedt's catalyst were added and the mixture was stirred under argon at 80 °C. The mixture was left to stir for 5 hours, after which the mixture was cooled to room temperature and purified by column chromatography (Solid load, Büchi FlashPure 40g, eluent: Heptane/$CHCl_3$ 0/100 – 100/0) to yield the target compound as a soft yellowish wax (52.0 mg, 0.016 mmol, 32%).

^{1}H NMR (400 MHz, $CDCl_3$) δ 8.24 (d, J = 8.6 Hz, 4H, AQ), 7.72 (d, J = 2.6 Hz, 4H, AQ), 7.23 (m, 4H, AQ), 4.16 (m, 8H, 4x AQOC$\underline{H}_2$), 1.95 – 1.77 (m, 8H, 4x AQOCH$_2$C$\underline{H}_2$), 1.54 – 1.38 (m, 16H, 2x AQOCH$_2$CH$_2$C$\underline{H}_2$C$\underline{H}_2$), 0.97 (t, J = 7.1 Hz, 6H, 2x C$\underline{H}_3$CH$_2$CH$_2$), 0.67 – 0.50 (m, 4H, 2x C$\underline{H}_2$SiO), 0.09 (s, 190H, *o*DMS). ^{13}C NMR (100 MHz, $CDCl_3$) δ 13C NMR (100 MHz, CDCl3) δ 182.39, 164.09, 135.86, 129.68, 126.98, 120.93, 110.57, 77.33, 77.01, 76.70, 68.82, 29.65, 28.81, 28.72, 28.10, 23.07, 22.41, 18.20, 14.00, 1.41, 1.19, 1.08, 1.06, 0.67, 0.20, -0.00. MALDI-ToF-MS M_w calculated for $C_{112}H_{246}O_{39}Si_{32}$: 3110.0 g/mol, m/z found = 3137.94 $[M+Na]^+$.

Me-AQ-Si$_{15}$

A flame dried Schlenk flask was charged with **7** (15.0 mg, 0.0465 mmol), $CH_3Si_{15}H$ (126.5 mg, 0.114 mmol) and dry DCM (0.8 mL). Subsequently, 1 drop Karstedt's catalyst was added and the mixture was stirred under argon at room temperature. The mixture was left to stir for 20 hours, after which the mixture was purified by column chromatography (Solid load, Büchi FlashPure 40g, eluent: Heptane/$CHCl_3$ 100/0 – 50/50) to yield the target compound as yellowish wax (50.0 mg, 0.0349 mmol, 75%).

^{1}H NMR (400 MHz, $CDCl_3$) δ 8.22 (m, 2H, AQ), 7.71 (m, 2H, AQ), 7.22 (m, 2H, AQ), 4.14 (t, J = 6.6 Hz, 2H, AQOC$\underline{H}_2$), 3.98 (s, 3H, C$\underline{H}_3$OAQ), 1.90 – 1.80 (m, 2H, AQOCH$_2$C$\underline{H}_2$), 1.61 – 1.37 (m, 4H, AQOCH$_2$CH$_2$C$\underline{H}_2$C$\underline{H}_2$), 0.63 – 0.54 (m, 2H, C$\underline{H}_2$SiO), 0.13 – 0.01 (m, 90H, *o*DMS). ^{13}C NMR (100 MHz, $CDCl_3$) δ 207.03, 182.42, 182.36, 164.55, 164.22, 136.04, 135.96, 129.81, 127.38, 127.10, 121.09, 120.75, 110.68, 110.10, 77.48, 77.16, 76.84, 68.96, 56.08, 31.07, 29.79, 28.95, 23.21, 18.34, 1.93, 1.33, 1.28, 1.23, 1.19, 0.34. MALDI-ToF-MS M_w calculated for $C_{51}H_{112}O_{18}Si_{15}$: 1432.40 g/mol, m/z found = 1457.39 $[M+Na]^+$.

Me-AQ-Si$_{32}$-AQ-Me

A flame dried Schlenk flask was charged with **7** (44.0 mg, 0.1365 mmol), $HSi_{32}H$ (113.0 mg, 0.00479 mmol) and dry DCM (0.5 mL). Subsequently, 20 µL Karstedt's catalyst was added and the mixture was stirred under argon at room temperature. The mixture was left to stir for 20 hours, after which the mixture was purified by column chromatography (Solid load, Büchi FlashPure 40g, eluent: Heptane/$CHCl_3$ 100/0 – 50/50) to yield the target compound as yellowish wax (98.0 mg, 0.0307 mmol, 64%).

^{1}H NMR (400 MHz, $CDCl_3$) δ 8.23 (m, 4H, AQ), 7.71 (m, 4H, AQ), 7.22 (m, 4H, AQ), 4.14 (m, 4H, 2x AQOC$\underline{H}_2$), 3.98 (s, 6H, 2x C$\underline{H}_3$OAQ), 1.91 – 1.78 (m, 4H, 2x AQOCH$_2$C$\underline{H}_2$), 1.60 – 1.36 (m, 8H, AQOCH$_2$CH$_2$C$\underline{H}_2$C$\underline{H}_2$), 0.65 – 0.52 (m, 4H, C$\underline{H}_2$SiO), 0.07 (s, 190H, *o*DMS). ^{13}C NMR (100 MHz, $CDCl_3$) δ 182.10, 182.04, 164.22, 163.89, 135.71, 135.63, 129.48, 127.04, 126.76, 120.76, 120.42, 110.35, 109.77, 77.13, 76.82, 76.50, 68.63, 55.75, 29.45, 28.61, 22.87, 18.00, 0.99, 0.88, 0.86, 0.84. MALDI-ToF-MS M_w calculated for $C_{104}H_{230}O_{39}Si_{32}$: 2998.90 g/mol, m/z found = 3026.80 $[M+Na]^+$.

H-AQ-Si$_{15}$

A flame dried Schlenk flask was charged with **8** (51.4 mg, 0.176 mmol), $CH_3Si_{15}H$ (217.4 mg, 0.185 mmol) and dry DCM (1 mL). Subsequently, 1 drop Karstedt's catalyst was added and the mixture was stirred under argon at room temperature. The mixture was left to stir for 10 hours, after which the mixture was purified by column chromatography (Solid load, Büchi FlashPure 24g, eluent: Heptane/$CHCl_3$ 100/0 – 50/50) to yield the target compound as yellowish wax (211.1 mg, 0.100 mmol, 85%).

^{1}H NMR (400 MHz, $CDCl_3$) δ 8.33 – 8.22 (m, 3H, AQ), 7.81 – 7.68 (m, 3H, AQ), 7.27 – 7.22 (m, 1H, AQ), 4.14 (t, J = 6.5 Hz, 2H, AQOC$\underline{H}_2$), 1.86 (p, J = 6.7 Hz, 2H, AQOCH$_2$C$\underline{H}_2$), 1.59 – 1.36 (m, 4H, AQOCH$_2$CH$_2$C$\underline{H}_2$C$\underline{H}_2$), 0.64 – 0.55 (m, 2H, C$\underline{H}_2$SiO), 0.08 (d, J = 5.2 Hz, 90H, *o*DMS). ^{13}C NMR (100 MHz, $CDCl_3$) δ 183.46, 182.29, 164.14, 135.72, 134.25, 133.87, 133.77, 133.75, 129.87, 127.27, 127.05, 121.62, 110.64, 68.98, 29.80, 28.95, 23.22, 18.35, 1.93, 1.57, 1.34, 1.29, 1.23, 1.20, 0.83, 0.35. MALDI

H-AQ-Si$_{32}$-AQ-H

A flame dried Schlenk flask was charged with **5** (60.9 mg, 0.2083 mmol), $HSi_{32}H$ (212.4 mg, 0.090 mmol) and dry DCM (1 mL). Subsequently, 1 drop of Karstedt's catalyst were added and the mixture was stirred under argon at room temperature. The mixture was left to stir for 10 hours, after which the mixture was purified by column chromatography (Solid load, Büchi

FlashPure 40g, eluent: Heptane/$CHCl_3$ 100/0 – 50/50) to yield the target compound as a soft yellowish wax (139.0 mg, 0.0472 mmol, 52%).

^{1}H NMR (400 MHz, $CDCl_3$) δ 8.35 – 8.19 (m, 6H, AQ), 7.83 – 7.64 (m, 6H, AQ), 7.27 – 7.22 (m, 2H, AQ), 4.14 (t, *J* = 6.5 Hz, 4H, 2x AQOC$\underline{H}_2$), 1.91 – 1.80 (m, 4H, 2x AQOCH_2C$\underline{H}_2$), 1.58 – 1.36 (m, 8H, AQOCH_2CH_2C$\underline{H}_2$C$\underline{H}_2$), 0.64 – 0.53 (m, 4H, 2x C$\underline{H}_2$SiO), 0.16 – 0.02 (m, 192H, *o*DMS). ^{13}C NMR (100 MHz, $CDCl_3$) δ 183.46, 182.29, 164.14, 135.72, 134.25, 133.87, 133.77, 133.74, 129.87, 127.27, 127.05, 121.62, 110.64, 68.98, 29.80, 28.95, 23.21, 18.35, 2.58, 1.23, 0.82, 0.14. MALDI-ToF-MS M_w calculated for $C_{102}H_{226}O_{37}Si_{32}$: 2938.80 g/mol, m/z found = 2965.80 $[M+Na]^+$.

2.3 Azo derivatives

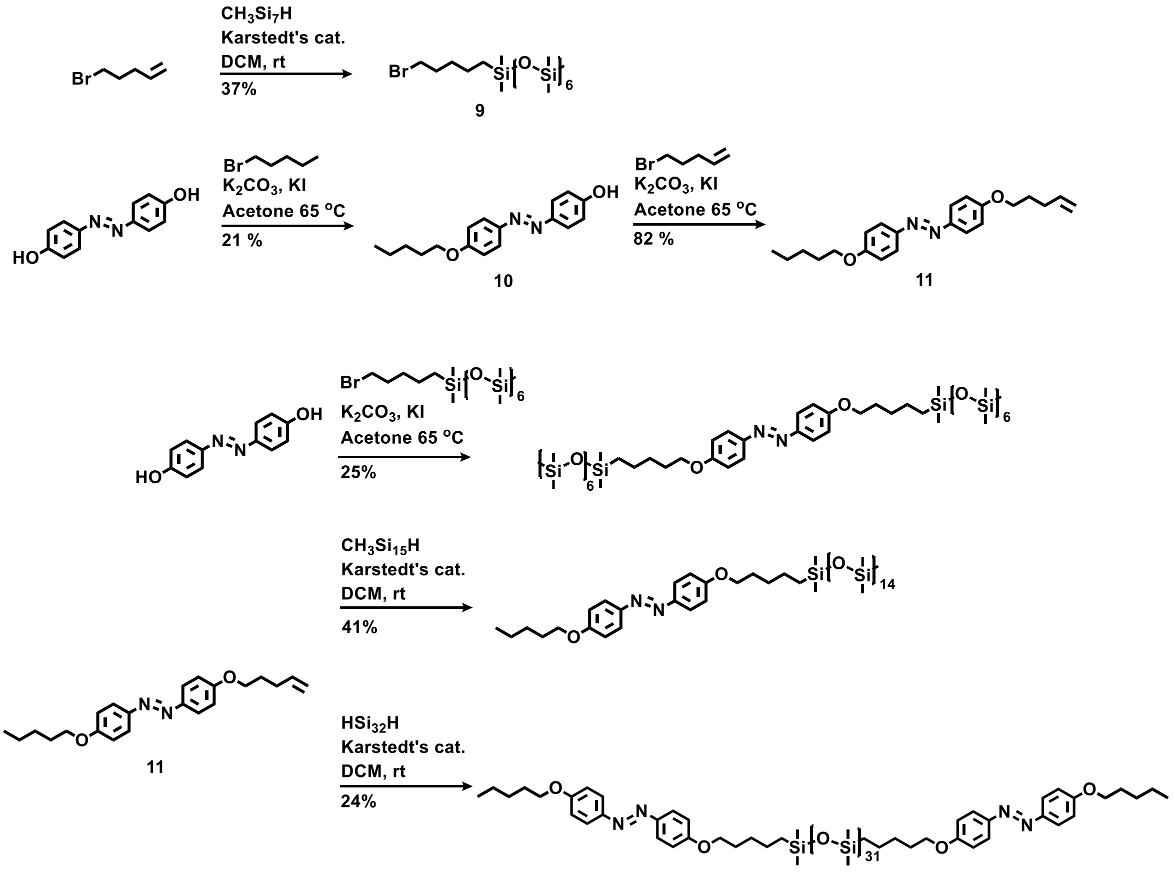

***Scheme 3**: Synthetic pathway towards Azo derivatives*

1-(5-bromopentyl)-1,1,3,3,5,5,7,7,9,9,11,11,13,13,13-pentadecamethylheptasiloxane (9)

An oven-dried round bottom flask was charged with CH_3Si_7H (1.0152 g, 1.956 mmol) and 1-bromo-5-pentene (384.5 mg, 2.542 mmol). To this, two drops of Karsteds catalyst were added. The mixture was stirred for 2 hours for full conversion and purified using column chromatography (SNAP-KiL, 40g, liquid injection from heptane, Hept/EtAc, 50/50, elution at 3 CV) to yield the target compounds as a colorless oil (488.5 mg, 0.731 mmol, 37%).

^1H NMR (400 MHz, $CDCl_3$) δ 3.40 (t, *J* = 6.9 Hz, 2H, $BrC\underline{H}_2$), 1.94 – 1.79 (m, 2H, $BrCH_2C\underline{H}_2$), 1.50 – 1.34 (m, 4H, $BrCH_2CH_2C\underline{H}_2C\underline{H}_2$), 0.58 – 0.52 (m, 2H, $SiC\underline{H}_2$), 0.10 – 0.03 (m, 48H, *o*DMS). ^{13}C NMR (100 MHz, $CDCl_3$) δ 33.93, 32.61, 31.85, 22.50, 18.09, 1.80, 1.19, 1.16, 1.09, 1.08, 0.17.

4-((4-(pentyloxy)phenyl)diazenyl)phenol (10)

An oven-dried round bottom flask was charged with dihydroxy azobenzene (304.6 mg, 1.42 mmol) K_2CO_3 (592.4 mg, 4.28 mmol, 3 eq) and KI (26.8 mg, 0.16 mmol) in acetone (30 mL). This suspension was stirred at room temperature and 1-bromopentane (0.09 mL, 0.71 mmol) was added dropwise. Consequently, the mixture was heated to reflux and stirred overnight.

The mixture was cooled to room temperature and filtered to remove the salts. The filter was rinsed with acetone and $CHCl_3$ and the organic phase was concentrated to remain with approx. 150 mL organic solvent. This was extracted with 1M HCl (50 mL) and the aqueous phase was extracted with $CHCl_3$ (2x50 mL). The organic phase was dried over $NaSO_4$, upon which the $NaSO_4$ turned deep red. The filter was rinsed thoroughly with additional $CHCl_3$ to remove all color. The organic fraction was dried *in vacuo* and the compound was purified using column chromatography (SNAP-Kil 40g, solid loaded from celite, eluent: DCM:MeOH, 99/1 – 95/5) to yield the target compound as a yellow solid (Yield = 41.6 mg, 0.146 mmol, 21%).

^{1}H NMR (400 MHz, $CDCl_3$) δ: 7.92 – 7.76 (m, 4H, $(C\underline{H})_2CN{=}NC(C\underline{H})_2$), 6.99 (d, J = 8.8 Hz, 2H, $HOC(C\underline{H})_2(CH)_2CN{=}N$), 6.93 (d, J = 8.8 Hz, 2H, $CH_2OC(C\underline{H})_2(CH)_2CN{=}N$), 4.95 (s, 1H, $\underline{H}OAr$), 4.03 (t, J = 6.6 Hz, 2H, $CH_2CH_2C\underline{H}_2OAr$), 1.82 (p, J = 6.8 Hz, 2H, $CH_2C\underline{H}_2CH_2OAr$), 1.43 (m, 4H, $CH_3C\underline{H}_2C\underline{H}_2CH_2CH_2OAr$), 0.95 (t, J = 7.0 Hz, 3H, $C\underline{H}_3CH_2CH_2CH_2CH_2OAr$). ^{13}C NMR (100 MHz, $CDCl_3$) δ 161.17, 157.63, 147.31, 146.91, 137.68, 124.56, 124.38, 115.74, 115.36, 114.70, 77.34, 77.02, 76.70, 67.49, 30.08, 28.37.

1-(4-(pent-4-en-1-yloxy)phenyl)-2-(4-(pentyloxy)phenyl)diazene (11)

An oven-dried round bottom flask was charged with **9** (33.0 mg, 0.116 mmol), K_2CO_3 (71.6 mg, 0. 518 mmol) and KI (2.1 mg, 0.12 mmol) in acetone (5 mL) and the mixture was heated to 60 °C under argon. Subsequently, 5-bromo-1-pentene (43.0 mg, 0.288 mmol) was added dropwise and the mixture is stirred overnight under reflux. The contents of the round bottom flask were filtered to remove the salts and the filter was flushed with acetone and $CHCl_3$. The organic was extracted with 0.5M HCl (50 mL) and the aqueous phase was extracted with $CHCl_3$ (2x50 mL). The organic phase was dried over $MgSO_4$ which turned orange so it was rinsed with $CHCl_3$ (3x 10 mL). The organic phase was dried *in vacuo*, yielding an orange solid. The crude was purified using column chromatography (Büchi FlashPure 40g, liquid injection from heptane, eluent: Heptane:EtAc 100/0 - 80/20) to yield the target compound as an orange solid (Yield = 36.1 mg, 0.102 mmol, 88%).

^{1}H NMR (400 MHz, $CDCl_3$) δ: 7.25 – 7.18 (m, 2H, $OC(CH)_2(C\underline{H})_2CN{=}N$), 6.93 – 6.85 (m, 2H, $OC(C\underline{H})_2(CH)_2CN{=}N$), 5.85 (ddt, J = 16.9, 10.1, 6.6 Hz, 1H, $CH_2{=}C\underline{H}CH_2CH_2$), 5.15 – 4.92 (m, 2H, $C\underline{H}_2{=}CHCH_2CH_2$), 4.04 (q, J = 6.7 Hz, 4H, 2x $C\underline{H}_2OAr$), 2.27 (q, J = 7.2 Hz, 2H, $CH_2{=}CHC\underline{H}_2$), 1.92 (p, J = 6.8 Hz, 2H, $CH_2{=}CHCH_2C\underline{H}_2$), 1.87 – 1.78 (m, 2H, $CH_3CH_2C\underline{H}_2$), 1.43 (m, 2H, $CH_3C\underline{H}_2CH_2$), 0.95 (t, J = 7.0 Hz, 3H, $C\underline{H}_3CH_2CH_2$). ^{13}C NMR was not recorded due to limited amount of material.

Si_7-Azo-Si_7

An oven-dried round bottom flask was charged with dihydroxy azobenzene (59.0 mg, 0.276 mmol), K_2CO_3 (121.6 mg, 0.870 mmol) and KI (6.8 mg, 0.040 mmol) in acetone (10 mL) and the mixture was heated to 60 °C under argon. Subsequently, $BrC_5H_{10}Si_7$ (462.7 mg, 0.694 mmol) was added dropwise and the mixture was stirred overnight under reflux. The content of the round bottom flask was then transferred to a separatory funnel and 100 mL water and 100 mL DCM were added. The organic phase was collected and the water phase was washed with DCM (4x 20 mL). The combined organic fractions were washed with brine (100 mL) and dried over $MgSO_4$. The mixture was concentrated *in vacuo* and purified using column chromatography (SNAP Si-KIL 40g, liquid inject from heptane, eluent Heptane/DCM 50/50 - 0/100) to yield the target compound as an orange viscous liquid (Yield: 91.1 mg, 0.070 mmol, 25%).

^1H NMR (400 MHz, $CDCl_3$) δ: 7.89 – 7.82 (m, 4H, $OC(CH)_2(C\underline{H})_2CN=N$), 7.01 – 6.95 (m, 4H, $OC(C\underline{H})_2(CH)_2CN=N$), 4.02 (t, J = 6.6 Hz, 4H, 2x $CH_2C\underline{H}_2OAr$), 1.82 (p, J = 6.7 Hz, 4H, 2x $C\underline{H}_2CH_2OAr$), 1.52 – 1.46 (m, 4H, 2x $C\underline{H}_2CH_2CH_2OAr$), 1.42 (t, J = 8.3 Hz, 4H, 2x $C\underline{H}_2CH_2CH_2CH_2OAr$), 0.62 – 0.55 (m, 4H, 2x Si-$C\underline{H}_2$), 0.13 – 0.03 (m, 90H, *o*DMS). ^{13}C NMR (100 MHz, $CDCl_3$) δ: 160.08, 145.86, 123.20, 113.57, 76.25, 75.93, 75.61, 67.22, 28.64, 27.90, 22.01, 17.13, 0.71, 0.11, -0.00, -0.88, -1.08. MALDI-ToF-MS M_w calculated for $C_{52}H_{118}N_2O_{14}Si_{14}$: 1386.54 g/mol, m/z found = 1387.53 $[M+H]^+$.

Azo-Si_{15}

An oven-dried round bottom flask was charged with **11** (52.3 mg, 0.0616 mmol), $CH_3Si_{15}H$ (86.4 mg, 0.0778 mmol) and dry DCM (3 mL). Subsequently, 2 drops Karstedt's cat were added and the mixture was stirred under argon at room temperature overnight . The mixture was purified using column chromatography (Büchi FlashPure 40g, liquid inject from heptane, eluent Heptane:EtAc, 100/0 - 80/20) to yield the product as an orange solid (Yield = 36.6 mg, 0.025 mmol, 41%).

^1H NMR (400 MHz, $CDCl_3$) δ 7.86 (d, J = 8.9 Hz, 4H, 2x $OC(CH)_2(C\underline{H})_2CN=N$), 6.98 (m, 4H, 2x $OC(C\underline{H})_2(CH)_2CN=N$), 4.03 (m, 4H, $C\underline{H}_2OAr$), 1.82 (p, J = 6.6 Hz, 4H, $C\underline{H}_2CH_2OAr$), 1.43 (m, 4H, $C\underline{H}_2CH_2CH_2OAr$), 0.95 (t, J = 7.0 Hz, 3H, $C\underline{H}_3CH_2CH_2CH_2CH_2OAr$), 0.65 – 0.52 (m, 2H, $SiC\underline{H}_2$), 0.08 (m, 122H, *o*DMS). ^{13}C NMR (100 MHz, $CDCl_3$) δ: 182.79, 164.48, 130.25, 123.98, 120.74, 113.00, 104.76, 32.06, 28.19, 27.84, 27.18, 25.13, 21.60, 13.08, 1.44, -1.06. MALDI-ToF-MS M_w calculated for $C_{53}H_{122}N_2O_{16}Si_{15}$: 1462.53 g/mol, m/z found: 1465.05 g/mol $[M+H]^+$ and 1487.05 g/mol $[M+Na]^+$

Azo-Si_{32}-Azo

An oven-dried round bottom flask was charged with **11** (28.6 mg, 0.081 mmol), $HSi_{32}H$ (97.0 mg, 0.041 mmol) and dry DCM (2 mL). Subsequently, 5 drops of Karstedt's catalyst were added and the mixture was stirred at room temperature under argon until full conversion of the hydride. Hereafter, the material was purified using column chromatography (SNAP-Kil 24g, liquid injection from heptane, eluent Heptane:DCM 100/0 – 50/50) to yield the material as an orange viscous liquid. (30.4 mg, 0.010 mmol, 24%).

^{1}H NMR (400 MHz, $CDCl_3$) δ: 7.88 – 7.83 (m, 8H, 2x 4 Azo inside), 6.99 (d, *J* = 1.9 Hz, 4H, 2x 2 Azo pentane side), 6.97 (d, *J* = 1.8 Hz, 4H, 2x 2 AZO siloxane side), 4.03 (td, *J* = 6.6, 2.8 Hz, 8H, 4x C<u>H</u>$_2$OAr), 1.82 (m, 8H, 4x C<u>H</u>$_2$CH$_2$OAr), 1.52 – 1.36 (m, inference of H_2O peak, 16H, 4x C<u>H</u>$_2$C<u>H</u>$_2$CH$_2$CH$_2$OAr), 0.61 – 0.55 (m, 4H, 2x SiC<u>H</u>$_2$), 0.07 (m, 192H, oDMS). ^{13}C NMR (100 MHz, $CDCl_3$) δ 161.31, 147.09, 124.43, 114.80, 68.56, 29.87, 29.22, 29.07, 28.33, 23.23, 22.62, 18.29, 14.17, 1.33, 1.18, 0.34, 0.14. MALDI-ToF-MS M_w calculated for $C_{108}H_{250}N_4O_{35}Si_{32}$: 3059.05 g/mol, m/z found: 3060.08 $[M+H]^+$, 3081.94 $[M+Na]^+$, 3096.39 $[M+K]^+$.

2.4 OPV derivatives

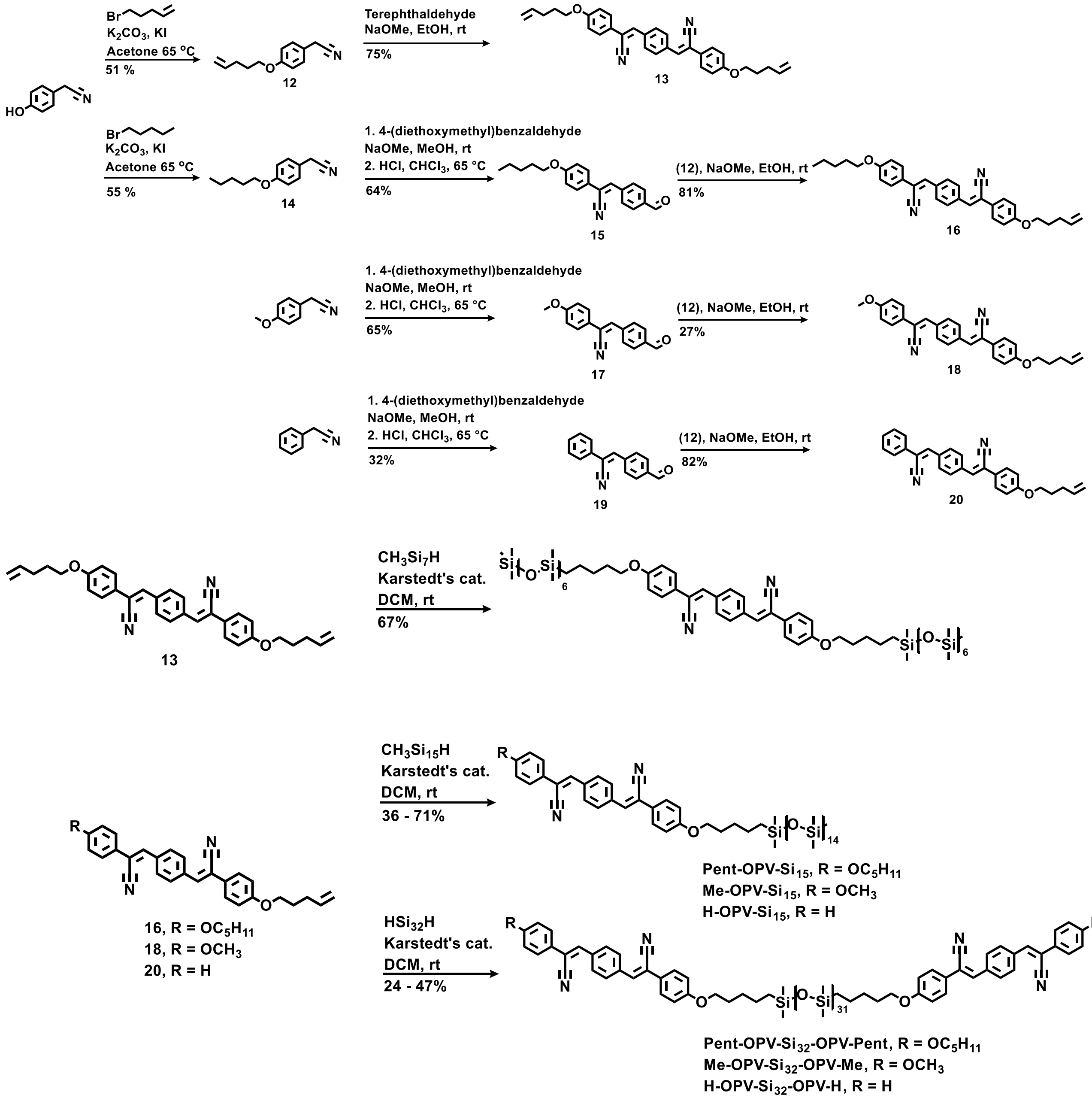

***Scheme 4**: Synthetic pathway towards OPV derivatives*

2-(4-(pent-4-en-1-yloxy)phenyl)acetonitrile (12)

An oven-dried round bottom flask was charged with 4-hydroxyphenylacetonitrile (508.8 mg, 3.82 mmol), K_2CO_3 (1.0592 g, 7.67 mmol) and KI (73.7 mg, 0.44 mmol) in acetone (30 mL) and the mixture was heated to 60 °C under argon. Subsequently, 5-bromo-1-pentene (0.8 mL, 6.77 mmol) was added dropwise and the mixture was stirred overnight under reflux. The content of the round bottom flask was then transferred to a separatory funnel and 100 mL water and 100 mL DCM were added. The organic phase was collected and the water phase was washed with DCM (4x 20 mL). The combined organic fractions were washed with brine

(100 mL) and dried over $MgSO_4$. The mixture was concentrated *in vacuo* and purified using column chromatography (SNAP Si-Kil 40g, liquid injection from heptane, eluent Heptane:DCM, 50/50 - 0/100) to yield the target compound as a transparent liquid (425 mg, 2.11 mmol, 55%).

^1H NMR (400 MHz, $CDCl_3$) δ 7.24 – 7.19 (m, 2H, Ar), 6.91 – 6.86 (m, 2H, Ar), 5.85 (m, 1H, $CH_2{=}C\underline{H}CH_2$), 5.10 – 4.97 (m, 2H, $C\underline{H}_2{=}CHCH_2$), 3.96 (t, *J* = 6.4 Hz, 2H, $C\underline{H}_2OAr$), 3.67 (s, 2H $NCC\underline{H}Ar$), 2.28 – 2.19 (m, 2H, $CH_2{=}CHC\underline{H}_2$), 1.89 (m, 2H, $C\underline{H}_2CH_2OAr$). ^{13}C NMR (100 MHz, $CDCl_3$) δ: 158.84, 137.70, 129.06, 121.62, 118.22, 115.29, 115.10, 67.28, 30.06, 28.34, 22.84.

3,3'-(1,4-phenylene)bis(2-(4-(pent-4-en-1-yloxy)phenyl)acrylonitrile) (13)

An oven-dried round bottom flask was charged with **12** (824.0 mg, 4.097 mmol) in EtOH (60 mL). Whilst stirring the mixture, 0.5 M NaOMe in MeOH was added (75 mL). Subsequently, terephthaldehyde (1.100 g, 8.194 mmol) was added and the mixture was stirred overnight, after which the sample turned into a yellow turbid solution and ^1H NMR showed full aldehyde conversion. Hereafter, the mixture was quenched with EtOH and filtered through a Büchner funnel. The funnel was rinsed with EtOH and MeOH and the residue was collected and dried in a vacuum oven. To remove remaining sodium salts, the mixture was extracted with $CHCl_3$/water (50 mL/50 mL, 3 x). The organic fraction was dried over $MgSO_4$ and concentrated *in vacuo* to yield the target compound as a bright yellow solid (1.00 gram, 2.00 mmol, 75%).

^1H NMR (400 MHz, $CDCl_3$) δ: 7.95 (s, 4H, central Ar), 7.63 (d, *J* = 8.9 Hz, 4H, Ar signals on cyanide side), 7.43 (s, 2H, $CNC{=}C\underline{H}$-Ar), 6.97 (d, *J* = 8.9 Hz, 4H, Ar signals on OCH_2 side), 5.87 (m, 2H, CH_2-$C\underline{H}{=}CH_2$), 5.13 – 4.95 (m, 4H, CH_2-$CH{=}C\underline{H}_2$), 4.03 (t, *J* = 6.4 Hz, 4H, $CH_2C\underline{H}_2O$-Ar), 2.33 – 2.19 (m, 4H, $C\underline{H}_2$-$CH{=}CH_2$), 1.98 – 1.84 (m, 4H, $C\underline{H}_2CH_2O$-Ar). ^{13}C NMR (100 MHz, $CDCl_3$) δ 160.03, 129.12, 127.35, 123.89, 115.58, 115.06, 29.94, 28.78, 28.24, 25.67.

2-(4-(pentyloxy)phenyl)acetonitrile (14)

An oven-dried round bottom flask was charged with 4-hydroxyphenylacetonitrile (494.0 mg, 3.71 mmol), K_2CO_3 (1.0612, 7.67 mmol) and KI (64.7 mg, 0.39 mmol) in acetone (15 mL) and the mixture was heated to 60 °C under argon. Subsequently, 1-bromopentane (0.6 mL, 4.83 mmol). The content of the round bottom flask was then transferred to a separatory funnel and 100 mL water and 100 mL DCM were added. The organic phase was collected and the water phase was washed with DCM (4x 20 mL). The combined organic fractions were washed with brine (100 mL) and dried over $MgSO_4$. The mixture was concentrated *in vacuo* and purified using column chromatography (SNAP Si-Kil 40g, liquid injection from heptane, eluent Heptane:DCM 50/50 - 0/100) to yield the target compound (383.0 mg, 1.8 mmol, 51%).

^{1}H NMR (400 MHz, $CDCl_3$) δ 7.24 – 7.18 (m, 2H, Ar), 6.93 – 6.86 (m, 2H, Ar), 3.95 (t, *J* = 6.4 Hz, 2H, $C\underline{H}_2OAr$), 3.68 (s, 2H, $ArC\underline{H}_2CN$), 1.84 - 1.73 (m, 2H, $C\underline{H}_2CH_2OAr$), 1.49 - 1.34 (m, 4H, $CH_3C\underline{H}_2C\underline{H}_2CH_2CH_2O$), 0.93 (t, *J* = 7.1 Hz, 3H, $C\underline{H}_3CH_2$). ^{13}C NMR (100 MHz, $CDCl_3$) δ: 158.94, 129.04, 121.51, 118.24, 115.10, 68.14, 31.89, 28.90, 28.18, 22.84, 22.70, 22.45, 14.01.

3-(4-formylphenyl)-2-(4-(pentyloxy)phenyl)acrylonitrile (15)

An oven-dried round bottom flask was charged with diethoxymethylbenzaldehyde (305.9 mg, 1.47 mmol), **14** (386.0 mg, 1.90 mmol) and MeOH (17 mL). To this, 1M NaOMe in MeOH (3 mL) was added and the mixture was stirred at room temperature under argon. Conversion was monitored using ^{1}H NMR to check for disappearance of the aldehyde peak. After complete conversion, the mixture was diluted in MeOH and the precipitate was filtered off. The precipitate was dissolved in $CHCl_3$ and concentrated HCl was added. The mixture was stirred at 65 °C for 1 hour until full conversion. Hereafter, the mixture was purified using column chromatography (SNAP-Kil 40g, liquid injection from heptane, eluent Heptane:DCM 50/50 - 0/100), to yield the target compound as a yellow solid (306 mg, 0.96 mmol, 65.3%).

^{1}H NMR (400 MHz, $CDCl_3$) δ 10.06 (s, 1H, $O{=}C\underline{H}Ar$), 7.98 (q, *J* = 8.4 Hz, 4H, central Ar), 7.64 (d, *J* = 8.9 Hz, 2H, Ar), 7.46 (s, 1H, $ArC\underline{H}{=}CCNAr$), 6.97 (d, *J* = 8.9 Hz, 2H, Ar), 4.01 (t, *J* = 6.6 Hz, 2H, $CH_3CH_2CH_2CH_2C\underline{H}_2OAr$), 1.87 – 1.77 (m, 2H, $CH_3CH_2CH_2C\underline{H}_2CH_2OAr$), 1.52 – 1.35 (m, 4H, $CH_3C\underline{H}_2C\underline{H}_2CH_2CH_2OAr$), 0.95 (t, *J* = 7.0 Hz, 3H, $C\underline{H}_3CH_2CH_2CH_2CH_2OAr$). ^{13}C NMR (100 MHz, $CDCl_3$) δ: 191.35, 160.66, 139.61, 137.70, 136.72, 130.11, 129.49, 127.60, 126.06, 117.60, 115.11, 114.46, 77.34, 77.02, 76.70, 68.30, 28.85, 28.16, 22.45, 14.02.

3-(4-(2-cyano-2-(4-(pent-4-en-1-yloxy)phenyl)vinyl)phenyl)-2-(4-(pentyloxy)phenyl)acrylonitrile (16)

An oven-dried round bottom flask was charged with **12** (129.8 mg, 0.64 mmol) in EtOH (20 mL) and whilst stirring 0.5 M NaOMe in MeOH (5.5 mL) was added. Subsequently, **14** (205.3 mg, 0.64 mmol) was added and the mixture was stirred at room temperature overnight. After confirmation of aldehyde conversion, the mixture was diluted with EtOH and washed through a Büchner funnel. This was rinsed with cold EtOH (20 mL) and cold MeOH (20 mL) and the residue was collected and dried in the oven. The product was obtained as a yellow solid (Yield = 250.7 mg, 0.579 mmol, 81%).

^{1}H NMR (400 MHz, $CDCl_3$) δ 7.95 (s, 4H, central Ar), 7.63 (d, *J* = 8.8 Hz, 4H, 2x Ar), 7.43 (s, 2H, 2x $ArC\underline{H}{=}CCNAr$), 6.97 (d, *J* = 8.8 Hz, 4H, 2x Ar), 5.87 (m, 1H, $CH_2{=}C\underline{H}CH_2CH_2$), 5.13 – 4.98 (m, 2H, $C\underline{H}_2{=}CHCH_2CH_2$), 4.02 (q, *J* = 6.7 Hz, 4H, $C\underline{H}_2OAr$), 2.26 (q, *J* = 7.2 Hz, 2H, $CH_2{=}CHC\underline{H}_2CH_2$), 1.97 – 1.77 (m, 4H, $CH_2{=}CHCH_2C\underline{H}_2$, $CH_3CH_2CH_2C\underline{H}_2CH_2OAr$), 1.43 (m,

4H, $CH_3C\underline{H}_2C\underline{H}_2CH_2CH_2OAr$), 0.95 (t, *J* = 7.1 Hz, 3H, $C\underline{H}_3CH_2CH_2CH_2CH_2OAr$). ^{13}C NMR (100 MHz, $CDCl_3$) δ: 158.84, 137.70, 129.06, 121.63, 118.22, 115.29, 115.11, 67.29, 30.06, 28.34, 22.84.

3-(4-formylphenyl)-2-(4-methoxyphenyl)acrylonitrile (17)

An oven-dried round bottom flask was charged with diethoxymethylbenzaldehyde (298.6 mg, 1.43 mmol), 4-methoxyphenylacetonitrile (277.9 mg, 1.88 mmol) and MeOH (10 mL). To this, 1M NaOMe in MeOH (6 mL) was added and the mixture was stirred at room temperature under argon. Conversion was monitored using ^{1}H NMR to check for disappearance of the aldehyde peak. After complete conversion, the mixture was diluted in H_2O and the precipitate was filtered off. The precipitate was dissolved in $CHCl_3$ (10 mL) and concentrated HCl was added. The mixture was stirred at 65 °C for 1 hour until full conversion. Hereafter, the mixture was purified using column chromatography (SNAP-Kil 40g, liquid injection from heptane, Heptane:DCM 50/50 - 0/100) to yield the product as a yellow solid (208.3 mg, 0.791 mmol, 55%).

^{1}H NMR (400 MHz, $CDCl_3$) δ 10.05 (d, *J* = 1.4 Hz, 1H, $O{=}C\underline{H}Ar$), 8.05 – 7.91 (m, 4H, central Ar), 7.68 – 7.61 (m, 2H, Ar), 7.47 (s, 1H, $ArC\underline{H}CCNAr$), 6.98 (m, 2H, Ar), 3.87 (s, 3H, $C\underline{H}_3OAr$). ^{13}C NMR (100 MHz, CDCl3) δ 158.85, 137.71, 129.06, 121.63, 115.30, 115.11, 67.29, 30.07, 28.35, 22.85, 22.70**.**

3-(4-((2-cyano-2-(4-(pent-4-en-1-yloxy)phenyl)vinyl)phenyl)-2-(4-methoxyphenyl)acrylonitrile (18)

An oven-dried round bottom flask was charged with **12** (105.8 mg, 0.530 mmol) in EtOH (5 mL) and whilst stirring 0.5 M NaOMe in MeOH (5 mL) was added. Subsequently, **17** (134.5 mg, 0.510 mmol) was added and the mixture was stirred at room temperature overnight. After confirmation of aldehyde conversion, the mixture was diluted with H_2O and washed through a Büchner funnel. This was rinsed with cold EtOH (20 mL) and cold MeOH (20 mL) and the residue was collected and dried in the oven. The product was obtained as a yellow solid (61.0 mg, 0.137 mmol, 27%).

^{1}H NMR (400 MHz, $CDCl_3$) δ 7.96 (s, 4H, central Ar), 7.68 – 7.61 (m, 4H, Ar), 7.43 (d, *J* = 1.9 Hz, 2H, 2x $ArC\underline{H}CCNAr$), 6.98 (m, 4H, Ar), 5.87 (m, 1H, $CH_2{=}C\underline{H}CH_2$), 5.15 – 4.95 (m, 2H, $C\underline{H}_2{=}CHCH_2$), 4.03 (t, *J* = 6.4 Hz, 2H, $C\underline{H}_2OAr$), 3.87 (s, 3H, $C\underline{H}_3OAr$), 2.32 – 2.17 (m, 2H, $CH_2{=}CHC\underline{H}_2$), 1.99 – 1.84 (m, 2H, $C\underline{H}_2CH_2OAr$). ^{13}C NMR (100 MHz, $CDCl_3$) δ 160.73, 160.24, 138.56, 138.42, 137.63, 135.42, 135.36, 129.52, 127.48, 127.45, 126.75, 126.58, 118.00, 115.40, 115.06, 114.54, 112.33, 112.26, 77.36, 77.04, 76.72, 67.41, 55.49, 30.06, 28.32.

3-(4-formylphenyl)-2-phenylacrylonitrile (19)

An oven-dried round bottom flask was charged with diethoxymethylbenzaldehyde (1.999 g, 9.60 mmol), benzylacetonitril (1.125 g, 9.60 mmol) and MeOH (20 mL). To this, 1M NaOMe in MeOH (10 mL) was added and the mixture was stirred at room temperature under argon. Conversion was monitored using ^{1}H NMR to check for disappearance of the aldehyde peak. After complete conversion, the mixture was diluted in MeOH (30 mL) and the green/yellow precipitate was filtered off. The filtrate was poured in water and centrifuged to form a yellow gel at the bottom of the falcon tube. This gel was dissolved in $CHCl_3$ (30 mL) and concentrated HCl was added (10 mL). The mixture was stirred at 65 °C for 1 hour until full conversion. Hereafter, the mixture was purified using column chromatography (SNAP-Kil 100 g, liquid injection from heptane, eluent Heptane:DCM 50/50 - 0/100), to yield the target compound as a yellow solid (720 mg, 3.09 mmol, 32%).

^{1}H NMR (400 MHz, $CDCl_3$) δ 10.07 (s, 1H, O=CHAr), 8.04 (d, *J* = 8.3 Hz, 2H, Ar), 7.98 (d, *J* = 8.5 Hz, 2H, Ar), 7.75 – 7.67 (m, 2H, Ar), 7.53 – 7.41 (m, 3H, Ar). ^{13}C NMR (100 MHz, $CDCl_3$) δ 191.43, 140.37, 139.32, 137.16, 133.96, 130.24, 130.07, 129.84, 129.53, 129.36, 126.34, 117.54, 114.96, 77.48, 77.36, 77.16, 76.84, 0.14.

3-(4-(2-cyano-2-(4-(pent-4-en-1-yloxy)phenyl)vinyl)phenyl)-2-phenylacrylonitrile (20)

An oven-dried round bottom flask was charged with **12** (0.41 g, 2.06 mmol) in EtOH (20 mL) and whilst stirring 0.5 M NaOMe in MeOH (20 mL) was added. Subsequently, **19** (0.48 g, 2.06 mmol) was added and the mixture was stirred at room temperature overnight. After confirmation of aldehyde conversion, the mixture was diluted with EtOH (10 mL) and washed through a Büchner funnel. This was rinsed with cold EtOH (10 mL) and cold MeOH (100 mL) and the residue was collected and dried in the oven. The product was obtained as a yellow solid (416.5 mg, 1.68 mmol, 82%).

^{1}H NMR (400 MHz, $CDCl_3$) δ 8.05 – 7.92 (m, 4H, central Ar), 7.75 – 7.67 (m, 2H, Ar), 7.66 – 7.60 (m, 2H, Ar), 7.55 (s, 1H, ArCHCCNAr), 7.51 – 7.39 (m, 4H, Ar), 7.00 – 6.94 (m, 2H, Ar), 5.87 (m, 1H, CH=CH_2), 5.14 – 4.96 (m, 2H, CH=CH_2), 4.03 (t, *J* = 6.4 Hz, 2H, ArOCH_2), 2.34 – 2.19 (m, 2H, CH_2CH=CH_2), 1.93 (m, 2H, ArOCH_2CH_2). ^{13}C NMR (100 MHz, $CDCl_3$) δ 160.30, 140.80, 138.31, 137.62, 135.84, 135.05, 134.27, 129.76, 129.54, 129.17, 127.49, 126.53, 126.12, 117.94, 117.84, 115.41, 115.07, 112.71, 112.66, 67.41, 30.05, 28.32.

Si_7-OPV-Si_7

A flame dried Schlenk flask was charged with **12** (69.5 mg, 0.14 mmol), CH_3Si_7H (236.4 mg, 0.46 mmol) and dry DCM (0.5 mL). subsequently, 2 drops Karstedt's catalyst were added and the mixture was stirred for 2 hours under argon. The mixture was purified using column chromatography (Büchi FlashPure 40g, liquid injection from heptane, eluent Heptane:$CHCl_3$, 100/0 – 70/30) to yield the target compound as a yellow crystal (Yield: 142.0 mg, 0.093 mmol, 67%).

^{1}H NMR (400 MHz, $CDCl_3$) δ 7.95 (s, 4H, central Ar), 7.67 – 7.57 (m, 4H, Ar), 7.43 (s, 2H, 2x ArC<u>H</u>CCNAr), 6.96 (d, *J* = 8.8 Hz, 4H, Ar), 4.00 (t, *J* = 6.6 Hz, 4H, 2x ArOC$\underline{H}_2$), 1.82 (p, *J* = 7.1, 6.7 Hz, 4H, 2x ArOCH$_2$C$\underline{H}_2$), 1.53 – 1.35 (m, 8H, 2x ArOCH$_2$CH$_2$C$\underline{H}_2$C$\underline{H}_2$), 0.63 – 0.53 (m, 4H, 2x C$\underline{H}_2$SiO), 0.10 – 0.04 (m, 91H, *o*DMS). ^{13}C NMR (100 MHz, $CDCl_3$) δ 183.35, 160.25, 129.40, 127.85, 125.25, 115.04, 109.05, 69.75, 66.61, 50.46, 46.91, 30.74, 4.82, 1.80, 1.16, 1.08, 0.14. MALDI-ToF-MS M_w calculated for $C_{64}H_{124}N_2O_{14}Si_{14}$: 1536.60 g/mol, m/z found: 1561.58 $[M+Na]^+$.

Pent-OPV-Si_{15}

An oven-dried round bottom flask was charged with **16** (52.3 mg, 0.104 mmol), $CH_3Si_{15}H$ (124.0 mg, 0.112 mmol) and dry DCM (3 mL). Subsequently, 2 drops Karstedt's catalyst were added and the mixture was stirred under argon at room temperature overnight. The mixture was purified using column chromatography (Büchi FlashPure 40g, liquid injection from heptane, eluent Heptane:$CHCl_3$, 100/0 - 0/100) to yield the target compound as a sticky, bright yellow solid (Yield: 89.6 mg, 0.056 mmol, 54%).

^{1}H NMR (400 MHz, $CDCl_3$) δ 7.95 (s, 4H, central Ar), 7.63 (d, *J* = 8.7 Hz, 4H, Ar), 7.43 (s, 2H, 2x ArC<u>H</u>CCNAr), 6.96 (m, 5H, Ar), 4.01 (m, 4H, 2x ArOC$\underline{H}_2$), 1.87 – 1.77 (m, 4H, 2x ArOCH$_2$C$\underline{H}_2$), 1.52– 1.36 (m, 8H, 2x ArOCH$_2$CH$_2$C$\underline{H}_2$C$\underline{H}_2$), 0.95 (t, *J* = 7.1 Hz, 3H, C$\underline{H}_3$), 0.61 – 0.55 (m, 2H, C$\underline{H}_2$SiO), 0.10 – 0.04 (m, 90H, *o*DMS). ^{13}C NMR (100 MHz, $CDCl_3$) δ: 160.03, 138.60, 135.55, 129.65, 127.58, 118.30, 115.18, 112.54, 68.41, 29.86, 29.09, 29.03, 28.92, 28.35, 26.26, 23.23, 22.60, 18.38, 14.17, 1.93, 1.34, 1.29, 1.20, 0.34, 0.14. MALDI-ToF-MS M_w calculated for $C_{65}H_{127}N_2O_{16}Si_{15}$: 1612.58, found: 1612.60 $[M]^+$ and 1635.59 $[M+Na]^+$

Pent-OPV-Si_{32}-OPV-Pent

An oven-dried round bottom flask was charged with **16** (100.6 mg, 0.200 mmol), $HSi_{32}H$ (229.7 mg, 0.097 mmol) and dry DCM (2 mL). Subsequently, 2 drops Karstedt's cat were added and the mixture was stirred under argon at room temperature overnight. The mixture was purified using column chromatography (Büchi FlashPure 40g, liquid injection from heptane, eluent

Heptane:$CHCl_3$, 100/0 - 80/20) to yield the target compound as a brittle yellow solid (Yield: 153.3 mg, 47%).

^{1}H NMR (400 MHz, $CDCl_3$) δ: 7.97 (s, 8H, 2x central Ar), 7.65 (d, *J* = 8.7 Hz, 8H, Ar), 7.45 (s, 4H, 4x ArC$\underline{H}$CCNAr), 6.98 (m, 8H, Ar), 4.03 (m, 8H, 4x ArOC$\underline{H}_2$), 1.85 (h, *J* = 6.5 Hz, 8H, 4x ArOCH_2C$\underline{H}_2$), 1.46 (m, 8H, 4x ArOCH_2CH_2C$\underline{H}_2$), 0.97 (t, *J* = 7.1 Hz, 6H, 2x C$\underline{H}_3$), 0.64 – 0.56 (m, 4H, 2x C$\underline{H}_2$SiO), 0.09 (s, 196H, *o*DMS). ^{13}C NMR (100 MHz, $CDCl_3$) δ: ^{13}C NMR (100 MHz, $CDCl_3$) δ 160.37, 138.71, 129.50, 127.44, 117.51, 115.04, 112.11, 77.33, 77.02, 76.70, 68.14, 47.70, 29.71, 28.87, 28.10, 23.55, 22.50, 22.46, 18.53, 14.20, 1.19, 1.09, 1.04, 0.20. MALDI-ToF-MS M_w calculated for $C_{132}H_{262}N_4O_{35}Si_{32}$: 3359.15 g/mol, m/z found: 3381.99 $[M+Na]^+$.

Me-OPV-Si$_{15}$

An oven-dried round bottom flask was charged with **18** (18.8 mg, 0.0421 mmol), $CH_3Si_{15}H$ (62.7 mg, 0.056 mmol) and dry DCM (3 mL). Subsequently, 2 drops Karstedt's catalyst were added and the mixture was stirred under argon at room temperature overnight. The mixture was purified using column chromatography (Büchi FlashPure 40g, liquid injection from heptane, eluent Heptane:$CHCl_3$, 100/0 - 0/100, elution at 100% $CHCl_3$) to yield the target compound as a sticky yellow solid (Yield: 23.4 mg, 0.015 mmol, 36%).

^{1}H NMR (400 MHz, $CDCl_3$) δ 7.96 (s, 4H, central Ar), 7.70 – 7.56 (m, 4H, Ar), 7.43 (d, *J* = 2.7 Hz, 2H, 2x ArC$\underline{H}$CCNAr), 7.02 – 6.94 (m, 4H, Ar), 4.00 (t, *J* = 6.6 Hz, 2H, ArOC$\underline{H}_2$), 3.87 (s, 3H, C$\underline{H}_3$O-OPV), 1.81 (p, *J* = 6.7 Hz, 2H, ArOCH_2C$\underline{H}_2$), 1.53 – 1.37 (m, 4H, ArOCH_2CH_2C$\underline{H}_2$C$\underline{H}_2$), 0.62 – 0.54 (m, 2H, C$\underline{H}_2$SiO), 0.08 (dt, *J* = 3.9, 1.5 Hz, 93H, oDMS). ^{13}C NMR (100 MHz, $CDCl_3$) δ 160.73, 160.35, 138.60, 138.35, 135.46, 135.35, 129.52, 127.48, 127.43, 126.78, 126.48, 117.99, 115.03, 114.54, 112.41, 112.28, 68.26, 55.49, 29.71, 28.94, 23.09, 18.21, 1.79, 1.43, 1.19, 1.15, 1.09, 1.06, 0.68, 0.20. MALDI-ToF-MS M_w calculated for $C_{61}H_{119}N_2O_{16}Si_{15}$: 1556.51, found 1579.53 $[M+Na]^+$

Me-OPV-Si$_{32}$-OPV-Me

An oven-dried round bottom flask was charged with **18** (48.6 mg, 0.109 mmol), $HSi_{32}H$ (131.1 mg, 0.056 mmol) and dry DCM (2 mL). Subsequently, 1 drop of Karstedt's catalyst was added and the mixture was stirred under argon at room temperature overnight. The mixture was purified using column chromatography (Büchi FlashPure 40g, liquid injection from heptane, eluent Heptane:EtAc, 100/0 – 50/50, elution at 50% EtAc) to yield the target compound as a bright yellow solid (Yield: 93.7 mg, 0.029 mmol, 52%).

^{1}H NMR (400 MHz, $CDCl_3$) δ: 7.95 (s, 8H, central Ar), 7.69 – 7.55 (m, 8H, Ar), 7.43 (d, *J* = 2.7 Hz, 4H, ArC$\underline{H}$CCNAr), 6.97 (t, *J* = 8.5 Hz, 8H, Ar), 4.00 (t, *J* = 6.5 Hz, 4H, 2x ArOC$\underline{H}_2$), 3.87 (s, 6H, 2x C$\underline{H}_3$O-OPV), 1.81 (p, *J* = 6.6 Hz, 4H, 2x ArOCH_2C$\underline{H}_2$), 1.52 – 1.37 (m, 8H, 2x

$ArOCH_2CH_2C\underline{H}_2C\underline{H}_2$), 0.63 – 0.52 (m, 4H, 2x $C\underline{H}_2SiO$), 0.07 (d, *J* = 2.0 Hz, 198H, *o*DMS). ^{13}C NMR (100 MHz, $CDCl_3$) δ 160.73, 160.35, 138.59, 138.34, 135.46, 135.34, 129.52, 129.50, 127.48, 127.43, 126.77, 126.48, 117.99, 115.03, 114.54, 112.40, 112.27, 77.34, 77.02, 76.70, 68.25, 55.48, 29.72, 28.95, 23.09, 18.21, 1.41, 1.19, 1.07, 1.04, 0.68, 0.20. MALDI-ToF-MS M_w calculated for $C_{124}H_{246}N_4O_{35}Si_{32}$: 3247.0 g/mol, m/z found: 3273.95 $[M+Na]^+$.

H-OPV-Si$_{15}$

An oven-dried round bottom flask was charged with **20** (53.4 mg, 0.130 mmol), $CH_3Si_{15}H$ (182.3 mg, 0.160 mmol) and dry DCM (3 mL). Subsequently, 2 drops Karstedt's catalyst were added and the mixture was stirred under argon at room temperature overnight. The mixture was purified using column chromatography (Büchi FlashPure 40g, liquid injection from heptane, eluent Heptane:$CHCl_3$, 100/0 - 0/100, elution at 100% $CHCl_3$) to yield the target compound as a sticky bright yellow solid (Yield: 140.0 mg, 0.092 mmol, 71%).

^1H NMR (400 MHz, $CDCl_3$) δ 7.98 (d, *J* = 2.7 Hz, 4H, Ar), 7.74 – 7.68 (m, 2H, Ar), 7.63 (d, *J* = 8.8 Hz, 2H, Ar), 7.55 (s, 1H, $ArC\underline{H}CCNAr$), 7.50 – 7.38 (m, 5H, Ar), 6.96 (d, *J* = 8.9 Hz, 2H, Ar), 4.00 (t, *J* = 6.7 Hz, 2H, $SiCH_2CH_2CH_2CH_2C\underline{H}_2Ar$), 1.81 (m, 2H, $SiCH_2CH_2CH_2C\underline{H}_2CH_2Ar$), 1.54 – 1.36 (m, 4H, $SiCH_2C\underline{H}_2C\underline{H}_2CH_2CH_2Ar$), 0.62 – 0.55 (m, 2H, $SiC\underline{H}_2$), 0.10 – 0.04 (m, 90H). ^{13}C NMR (100 MHz, $CDCl_3$) δ 160.20, 140.60, 138.02, 135.67, 134.82, 134.08, 130.08, 129.55, 129.33, 129.07, 128.96, 128.64, 127.26, 126.21, 125.91, 117.74, 117.64, 114.84, 112.50, 68.06, 29.51, 28.74, 22.89, 18.01, 1.59, 1.22, 0.99, 0.95, 0.88, 0.86, 0.48. MALDI-ToF-MS M_w calculated for $C_{60}H_{117}N_2O_{15}Si_{15}$: 1526.51, found 1549.51 $[M+Na]^+$

H-OPV-Si$_{32}$-OPV-H

An oven-dried round bottom flask was charged with **20** (73.7 mg, 0.1777 mmol), $HSi_{32}H$ (203.5 mg, 0.086 mmol) and dry DCM (3 mL). Subsequently, 2 drops Karstedt's catalyst were added and the mixture was stirred under argon at room temperature overnight. The mixture was purified using column chromatography (Büchi FlashPure 40g, liquid injection from heptane, eluent Heptane:$CHCl_3$, 100/0 - 0/100, elution at 100% $CHCl_3$) to yield the target compound as a yellow solid (Yield: 79.9 mg, 0.029 mmol, 24%).

^1H NMR (400 MHz, $CDCl_3$) δ 8.01 – 7.94 (m, 8H, Ar), 7.71 (d, *J* = 7.2 Hz, 4H, Ar), 7.63 (d, *J* = 8.8 Hz, 4H, Ar), 7.55 (s, 2H, $ArC\underline{H}CCNAr$), 7.50 – 7.41 (m, 8H, Ar), 6.96 (d, *J* = 8.8 Hz, 4H, Ar), 4.00 (t, *J* = 6.5 Hz, 4H, 2x $SiCH_2CH_2CH_2CH_2C\underline{H}_2Ar$), 1.82 (dt, *J* = 14.3, 7.3 Hz, 4H, 2x $SiCH_2CH_2CH_2C\underline{H}_2CH_2Ar$), 1.51 – 1.39 (m, 8H, 2x $SiCH_2C\underline{H}_2C\underline{H}_2CH_2CH_2Ar$), 0.61 – 0.55 (m, 4H, 2x $SiC\underline{H}_2$), 0.07 (s, 192H, oDMS). ^{13}C NMR (100 MHz, $CDCl_3$) δ 160.41, 140.80, 138.22, 135.86, 134.94, 134.35, 129.75, 129.53, 129.16, 127.46, 126.11, 115.04, 112.70, 77.33, 77.02,

76.70, 67.30, 29.71, 28.94, 23.09, 18.21, 1.08, 1.04, 0.20. MALDI-ToF-MS M_w calculated for $C_{122}H_{242}N_4O_{33}Si_{32}$: 3186.97, found 3209.97 $[M+Na]^+$.

3. Block molecule bulk characterization

3.1 DPA derivatives

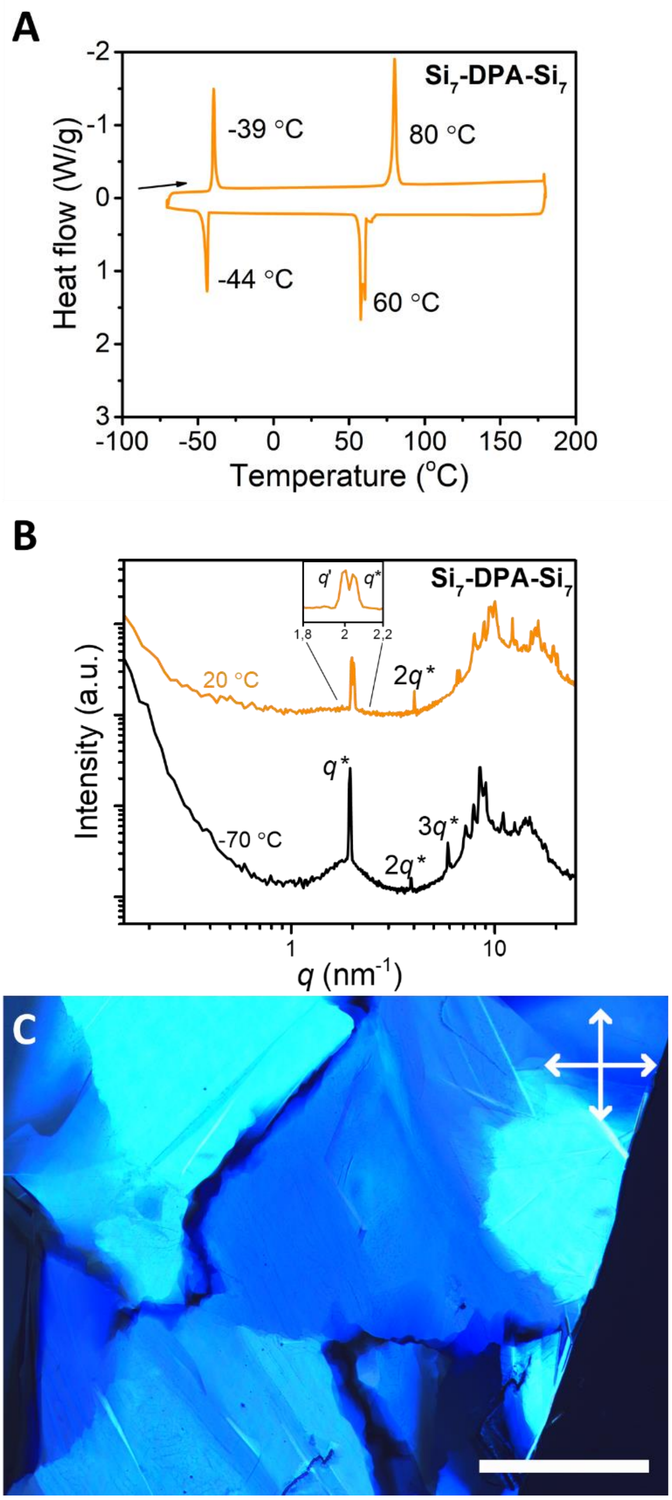

Figure S1: *Characterization of **Si7-DPA-Si7**. (A) Differential scanning calorimetry trace of the second cycle with 10 °C min^{-1} showing the melting and crystallization peaks (exothermic down). The arrow indicates heating. (B) 1D transmission scattering profile at room temperature showing lamellar order (d_{lam} = 3.1 nm) with an additional principal scattering peak at 3.2 nm, and a single lamellar order at −70 °C . (C) POM image at room temperature after slow cooling from its melt, under crossed polarizers. Scale bar indicates 200 µm.*

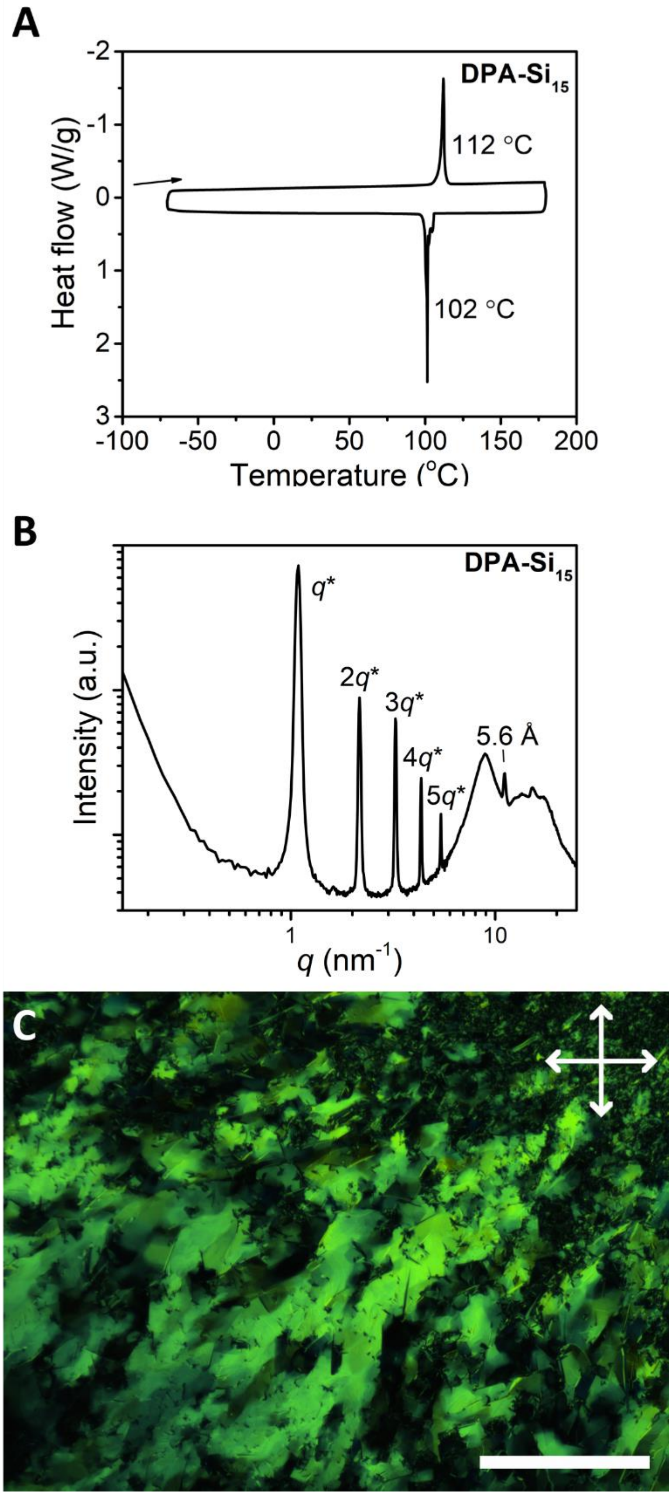

Figure S2: *Characterization of **DPA-Si15**. (A) Differential scanning calorimetry trace of the second cycle with 10 °C min^{-1} showing the melting and crystallization peaks (exothermic down). The arrow indicates heating. (B) 1D transmission scattering profile at room temperature showing lamellar order (d_{lam} = 5.8 nm). (C) POM image at room temperature after slow cooling from its melt, under crossed polarizers. Scale bar indicates 400 µm.*

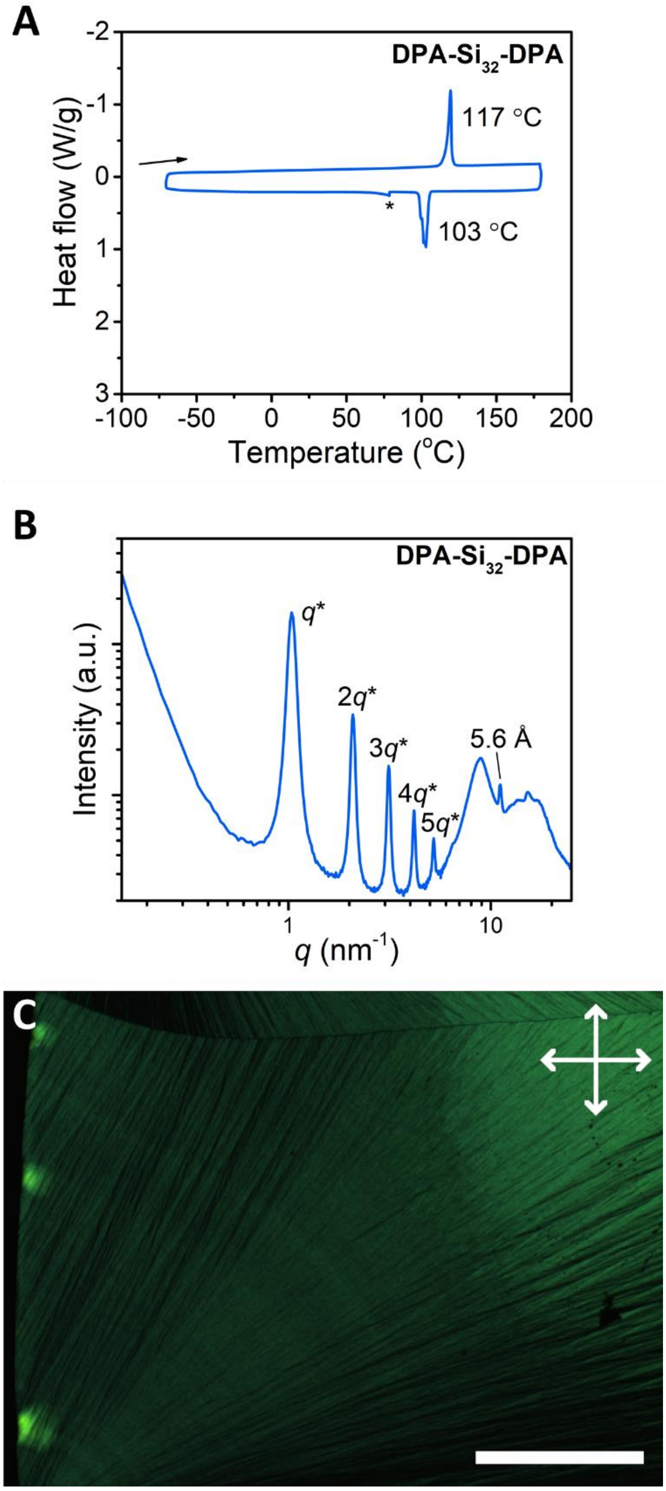

***Figure 3**: Characterization of **DPA-Si32-DPA**. (A) Differential scanning calorimetry trace of the second cycle with 10 °C min^{-1} showing the melting and crystallization peaks (exothermic down). The arrow indicates heating. Asterix indicates an instrumental error as no changes were observed below this temperature in SAXS. (B) 1D transmission scattering profile at room temperature showing lamellar order (d_{lam} = 6.1 nm). (C) POM image at room temperature after slow cooling from its melt, under crossed polarizers. Scale bar indicates 400 µm.*

3.2 AQ derivatives

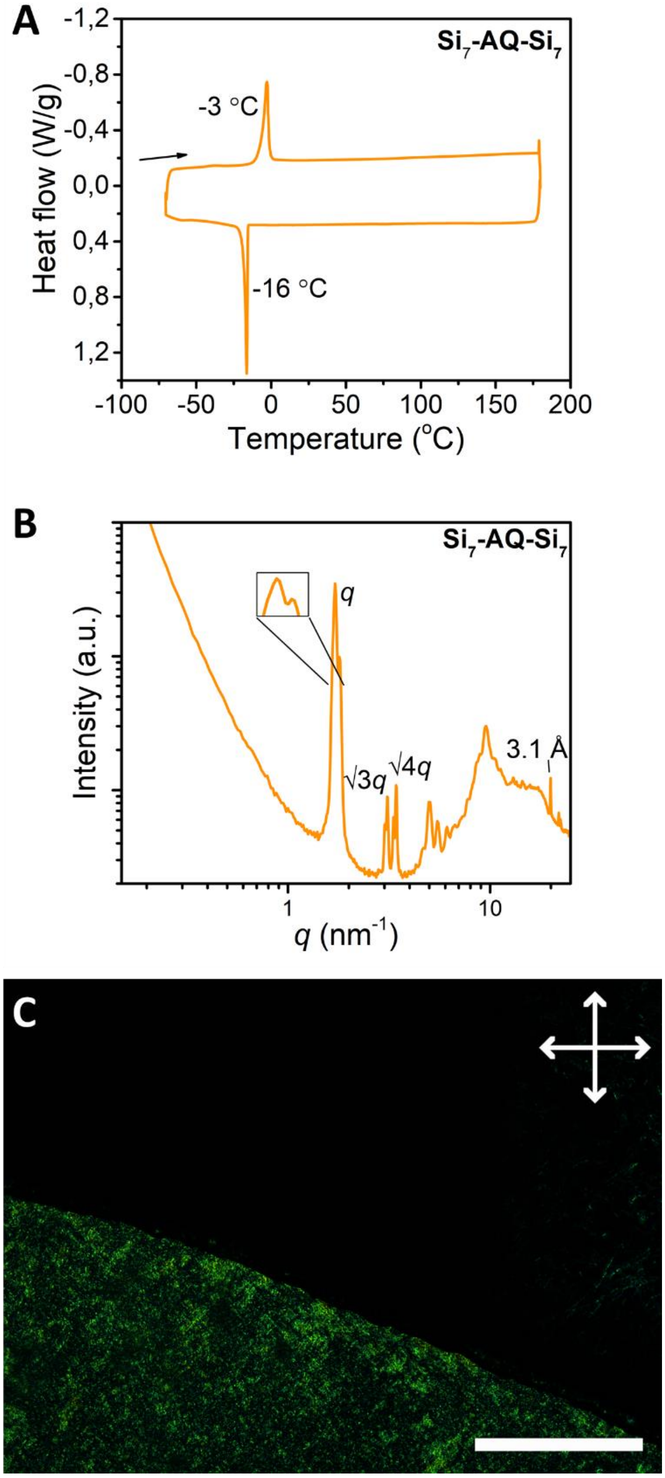

Figure S4: *Characterization of **Si_7-AQ-Si_7**. (A) Differential scanning calorimetry trace of the second cycle with 10 °C min^{-1} showing the melting and crystallization peaks (exothermic down). The arrow indicates heating. (B) 1D transmission scattering profile at room temperature showing double columnar order (d_{col} = 3.7 & 3.5 nm). (C) POM image at −15 °C after slow cooling from its melt, under crossed polarizers. Scale bar indicates 200 µm.*

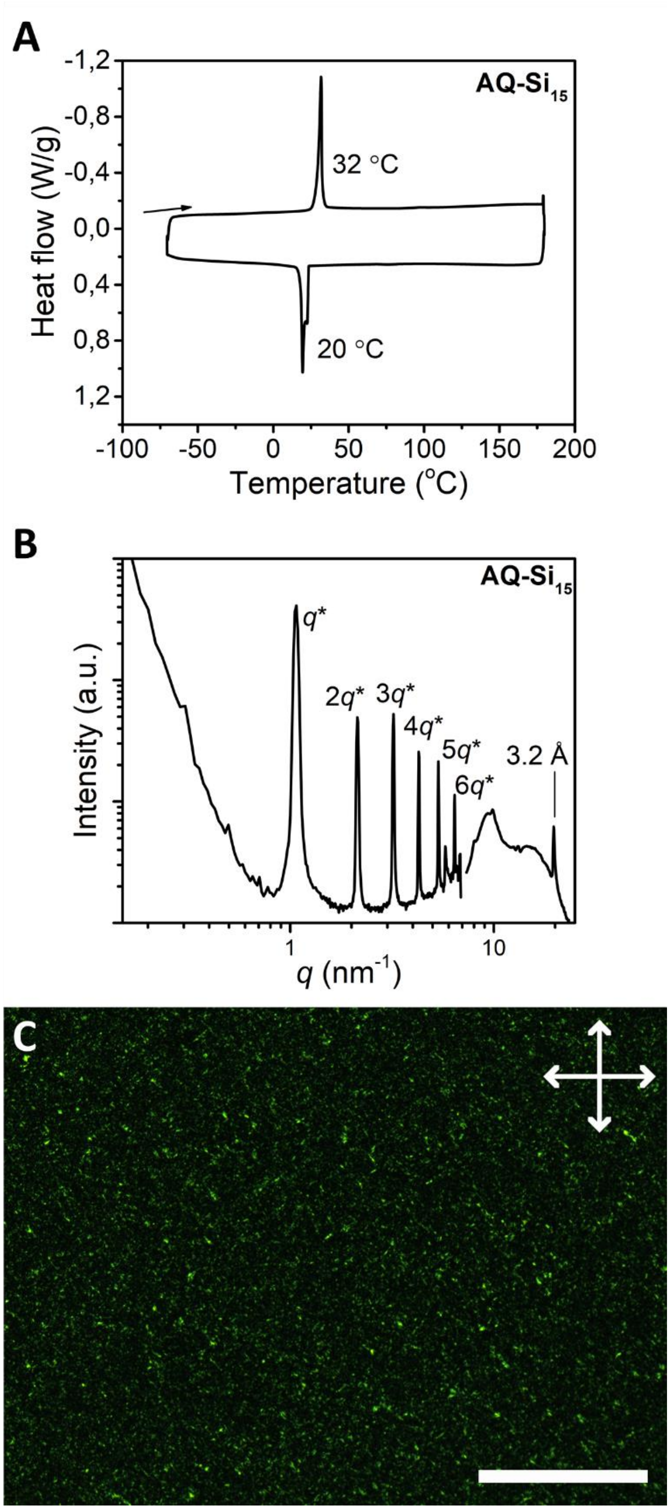

Figure S5: *Characterization of* ***AQ-Si15****. (A) Differential scanning calorimetry trace of the second cycle with 10 °C* min^{-1} *showing the melting and crystallization peaks (exothermic down). The arrow indicates heating. (B) 1D transmission scattering profile at 10 °C showing lamellar order (*d_{lam} *= 5.9 nm). (C) POM image at 15 °C after slow cooling from its melt, under crossed polarizers. Scale bar indicates 200 µm.*

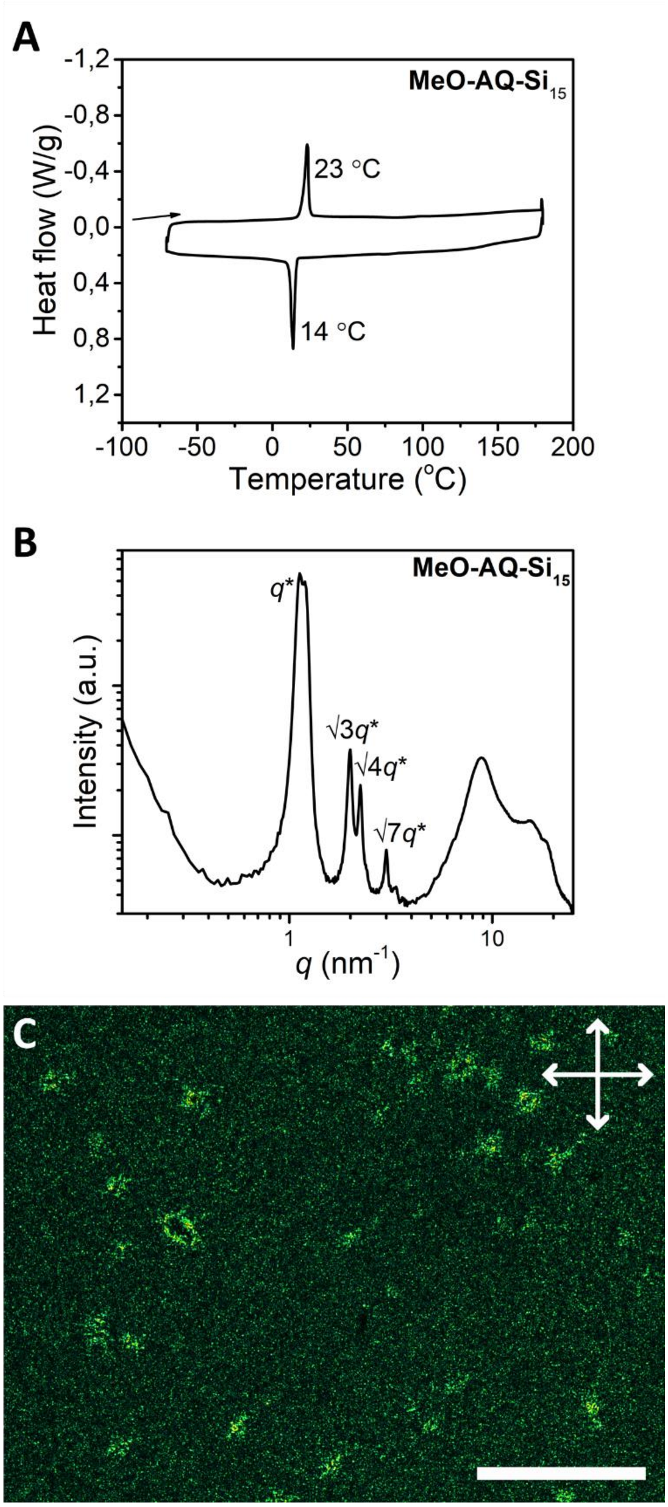

Figure S6: *Characterization of* ***MeO-AQ-Si*** $_{\mathbf{15}}$*. (A) Differential scanning calorimetry traces of the second cycle with 10 °C min*$^{-1}$ *showing the melting and crystallization peaks (exothermic down). The arrow indicates heating. (B) 1D transmission scattering profile at room temperature showing columnar order (*d_{col} *= 5.6 nm). (C) POM image at 14 °C after slow cooling from its melt, under crossed polarizers. Scale bar indicates 200 µm.*

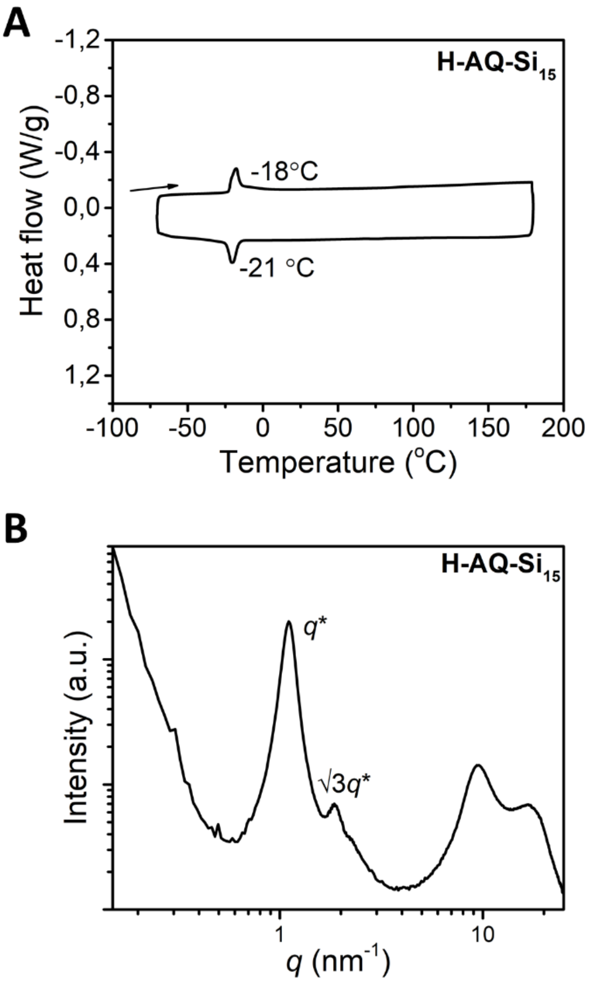

Figure S7: *Characterization of **H-AQ-Si$_{15}$**. (A) Differential scanning calorimetry traces of the second cycle with 10 °C min^{-1} showing the melting and crystallization peaks (exothermic down). The arrow indicates heating. (B) 1D transmission scattering profile at room temperature showing columnar order (d_{col} = 6.0 nm).No birefringent domains were observed below T_{exo} for **H-AQ-Si$_{15}$**.*

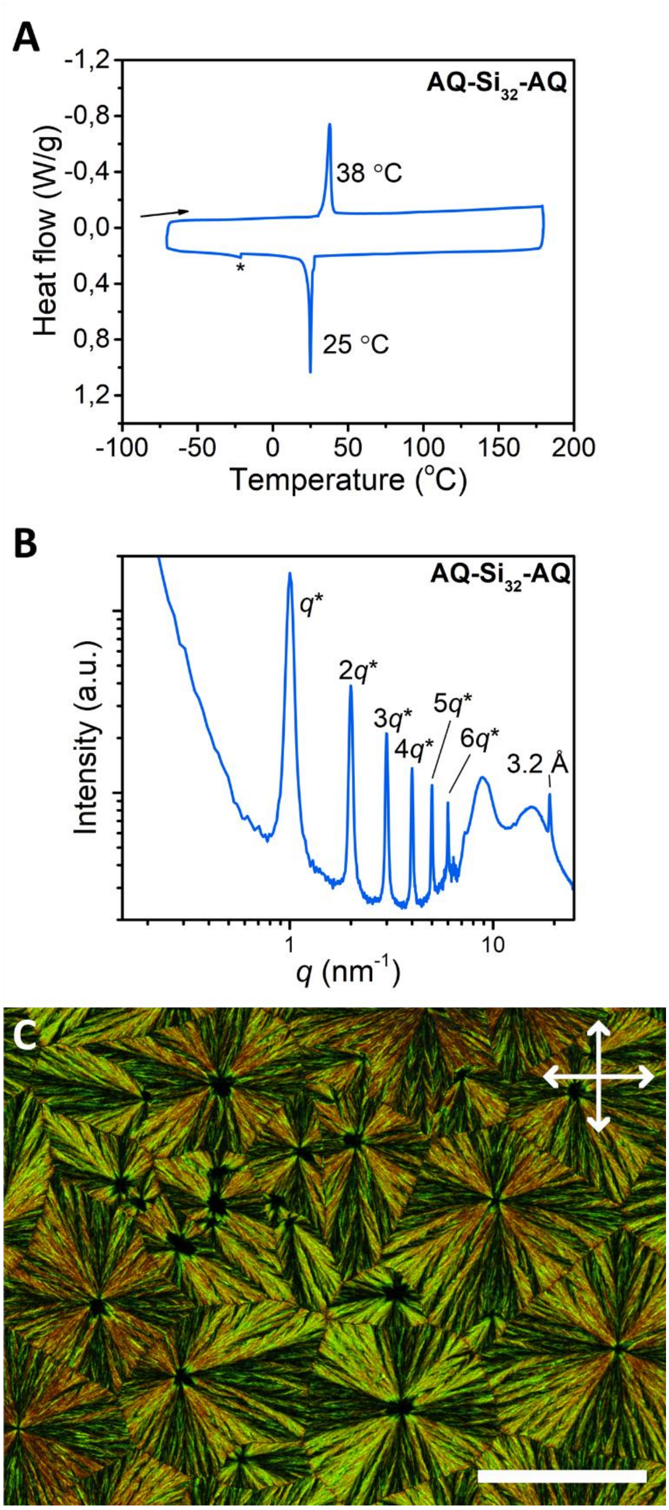

***Figure S8**: Characterization of **AQ-Si32-AQ**. (A) Differential scanning calorimetry trace of the second cycle with 10 °C min^{-1} showing the melting and crystallization peaks (exothermic down). The arrow indicates heating. Asterix indicates an instrumental error as no changes were observed below this temperature in SAXS. (B) 1D transmission scattering profile at room temperature showing lamellar order (d_{lam} = 6.3 nm). (C) POM image at room temperature after slow cooling from its melt, under crossed polarizers. Scale bar indicates 200 µm.*

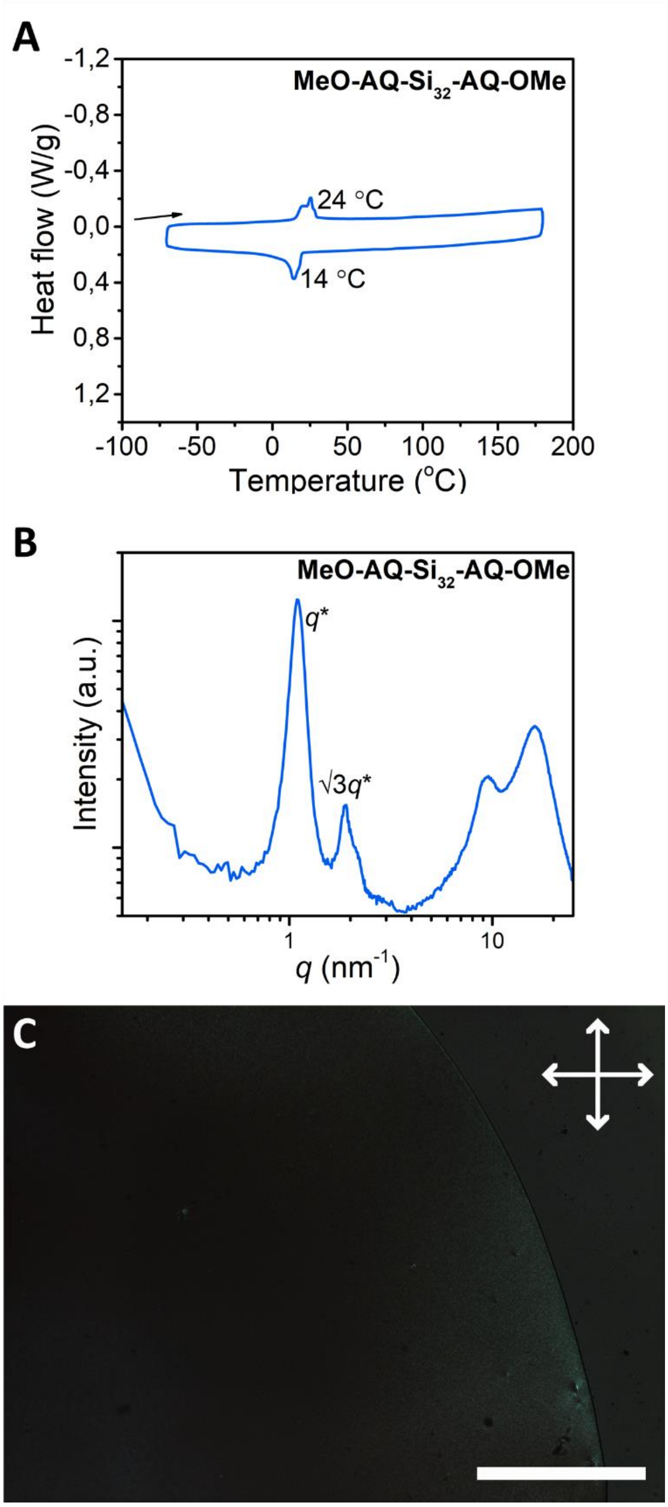

Figure S9: *Characterization of* ***MeO-AQ-Si*** $_{32}$ ***-AQ-OMe****. (A) Differential scanning calorimetry traces of the second cycle with 10 °C min*$^{-1}$ *showing the melting and crystallization peaks (exothermic down). The arrow indicates heating. (B) 1D transmission scattering profile at −25 °C showing columnar order (*d_{col} *= 5.7nm). (C) POM image at 10 °C after slow cooling from its melt, under crossed polarizers. Scale bar indicates 400 µm.*

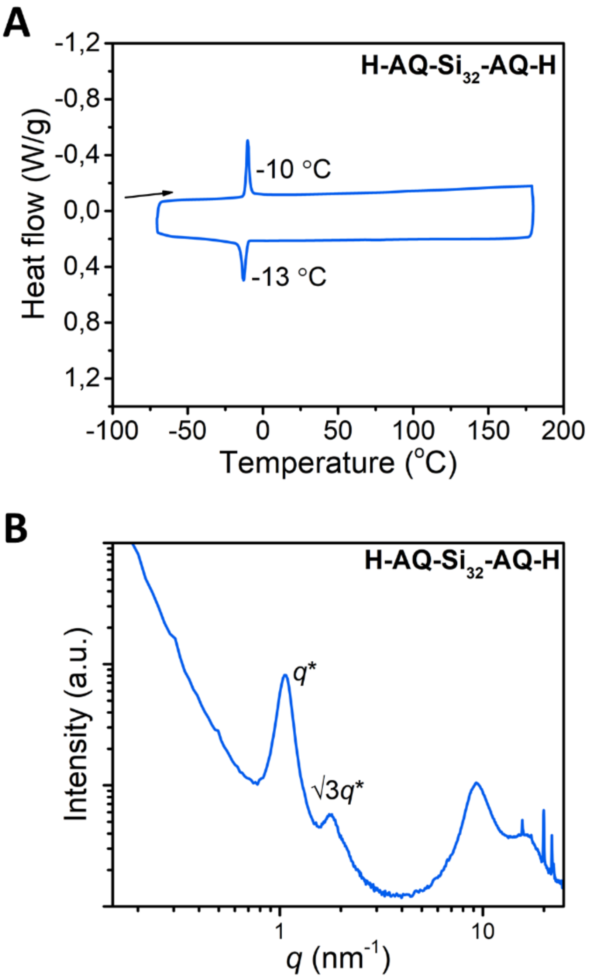

***Figure S10**: Characterization of **H-AQ-Si$_{32}$-AQ-H**. (A) Differential scanning calorimetry traces of the second cycle with 10 °C min^{-1} showing the melting and crystallization peaks (exothermic down). The arrow indicates heating. (B) 1D transmission scattering profile at −25 °C showing columnar order (d_{col} = 5.9 nm). No birefringent domains were observed below T_{exo} for **H-AQ-Si$_{32}$-AQ-H**.*

3.3 Azo derivatives

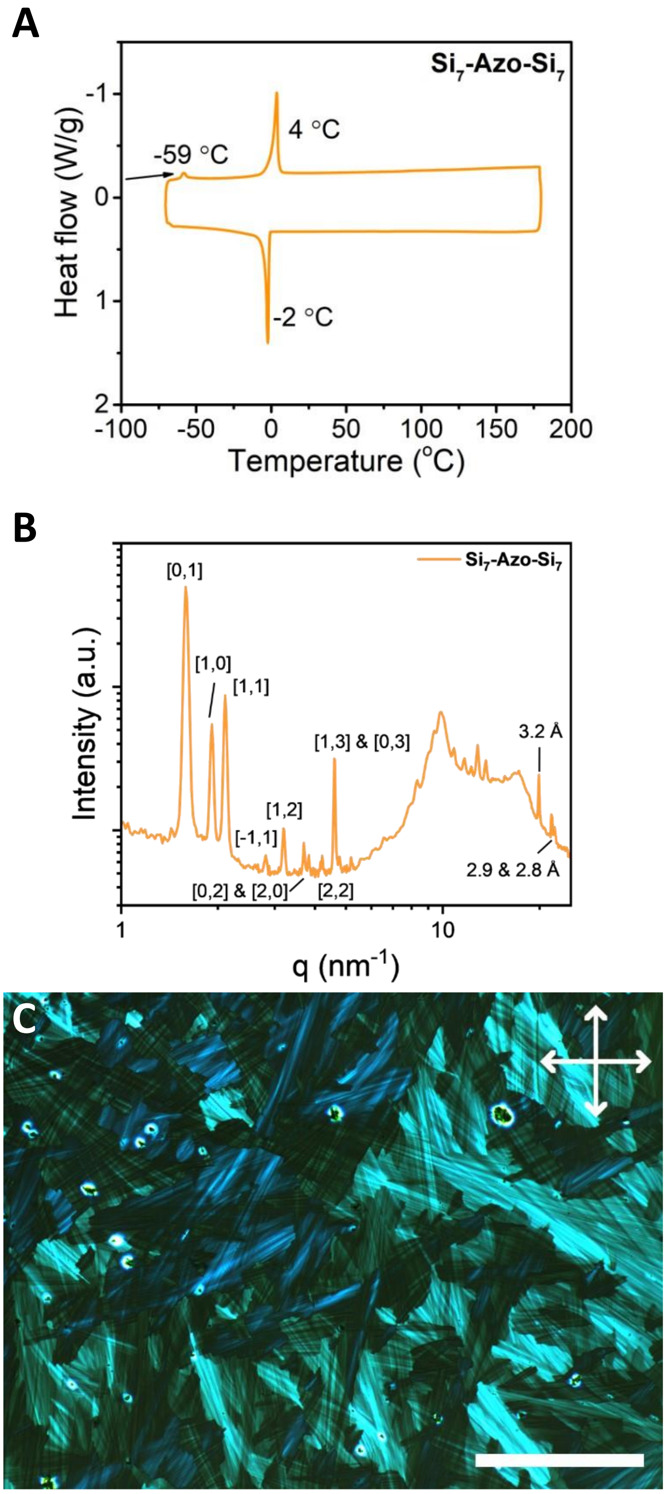

Figure S11: *Characterization of* ***Si₇-Azo-Si₇****. (A) Differential scanning calorimetry trace of the second cycle with 10 °C min^{-1} showing the melting and crystallization peaks (exothermic down). The arrow indicates heating. (B) 1D transmission scattering profile at −20 °C showing columnar oblique order ($d_{col,ob}$ = 5.6 nm). (C) POM image at −5 °C after slow cooling from its melt, under crossed polarizers. Scale bar indicates 400 µm.*

$$\frac{1}{d_{hk}^2} = \frac{1}{sin^2(\gamma)}(\frac{h^2}{a^2} + \frac{k^2}{b^2} - \frac{2hkcos(\gamma)}{ab}) \qquad \text{Eq ES1}$$

Equation ES1 with which the lattice parameters of a columnar oblique system can be determined, where *h* and *k* are the miller indices of the scattering plane, *d* is the lattice spacing, *a* and *b* are the lattice constants of the unit cell and *γ* is the angle in the unit cell.

Table S1: *Theoretical and observed values for $d_{col,obl}$ in **Si_7-Azo-Si_7**, using an angle of 74°. Calculated spacings are obtained using equation ES1. Resulting lattice constants are a = 3.4 nm and b = 4.1 nm. $d_{observed}$ is calculated from the scattering vector q through the equation d = 2π/q.*

h	***k***	$d_{calculated}$ **(nm)**	$d_{observed}$ **(nm)**
0	1	-	3.95
1	0	-	3.29
1	1	2.96	2.98
−1	1	2.24	2.22
0	2	1.98	1.95
1	2	1.95	1.95
2	1	1.69	1.70
2	0	1.64	1.62
2	2	1.48	1.49
1	3	1.36	1.36
0	3	1.32	1.36

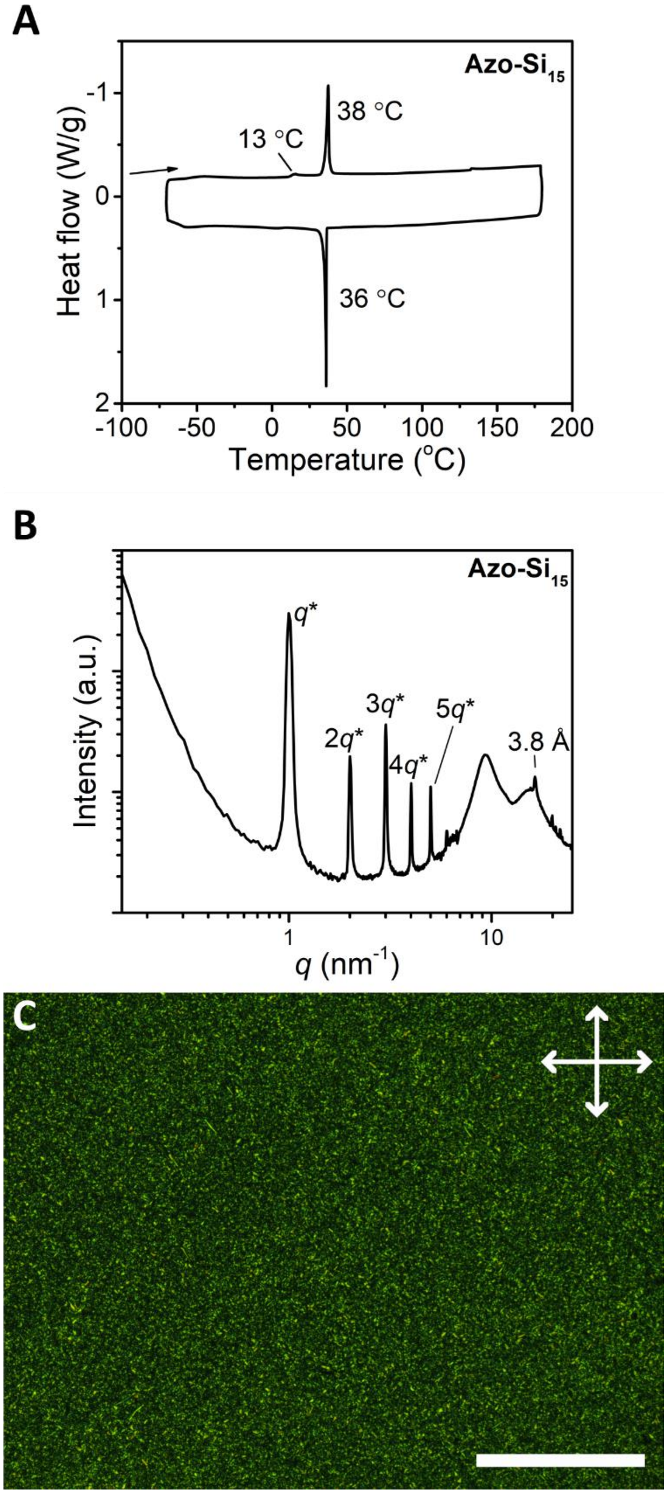

***Figure S12**: Characterization of **Azo-Si₁₅**. (A) Differential scanning calorimetry trace of the second cycle with 10 °C min^{-1} showing the melting and crystallization peaks (exothermic down). The arrow indicates heating. (B) 1D transmission scattering profile at −20 °C showing lamellar order (d_{lam} = 6.3 nm). (C) POM image at room temperature after slow cooling from its melt, under crossed polarizers. Scale bar indicates 400 µm.*

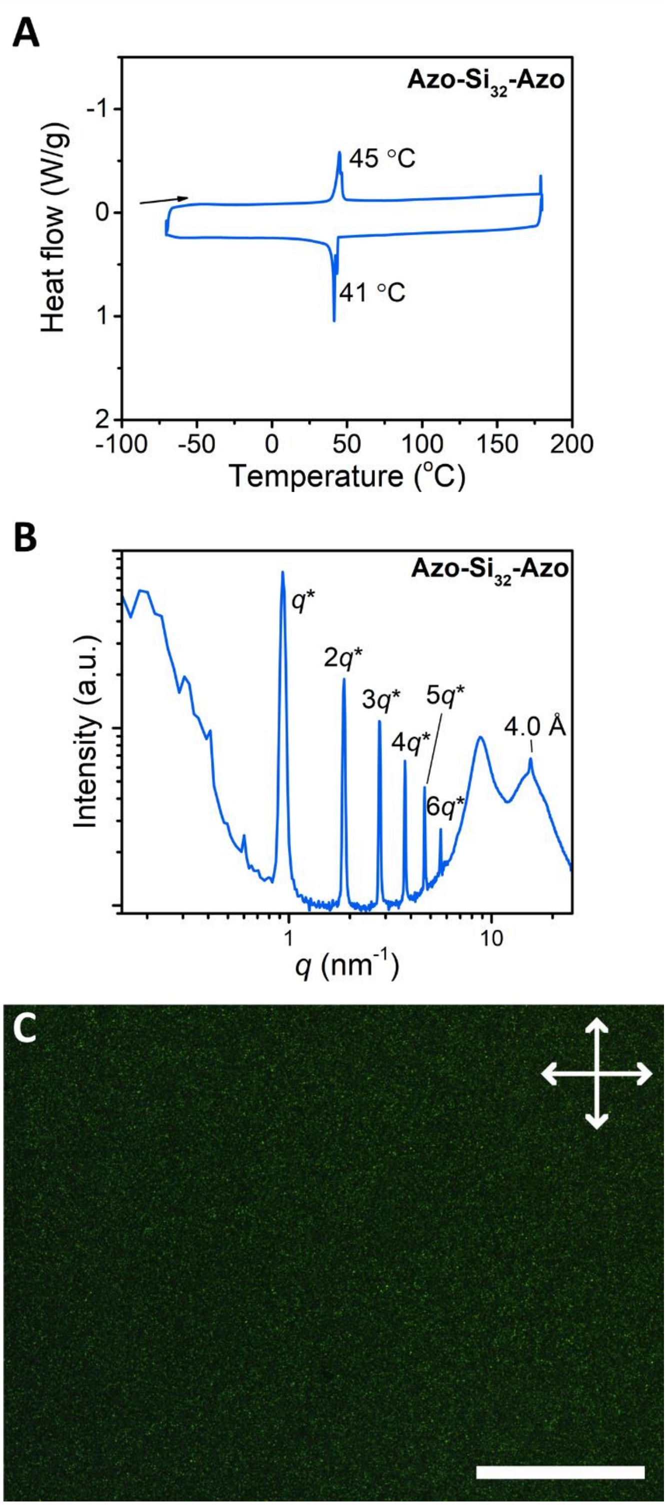

Figure S13: *Characterization of* ***Azo-Si$_{32}$-Azo****. (A) Differential scanning calorimetry trace of the second cycle with 10 °C min^{-1} showing the melting and crystallization peaks (exothermic down). The arrow indicates heating. (B) 1D transmission scattering profile at room temperature showing lamellar order (d_{lam} = 6.7 nm). (C) POM image at room temperature after slow cooling from its melt, under crossed polarizers. Scale bar indicates 200 µm.*

3.4 OPV derivatives

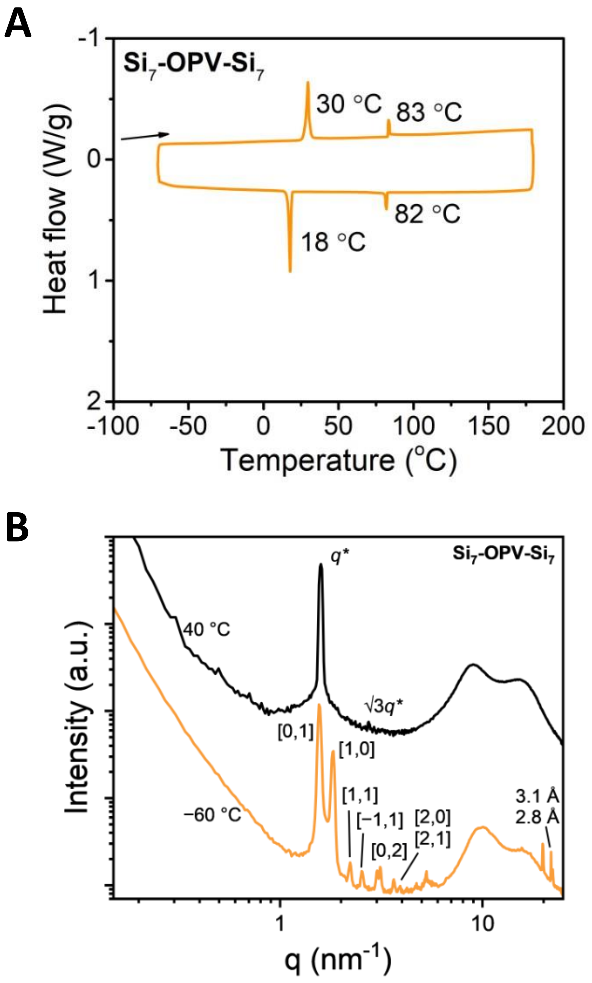

***Figure S14**: Characterization of **Si_7-OPV-Si_7**. (A) Differential scanning calorimetry trace of the second cycle with 10 °C min^{-1} showing the melting and crystallization peaks (exothermic down). The arrow indicates heating. (B) 1D transmission scattering profile showing columnar order ($d_{col,h}$ = 3.9 nm) at 40 °C, and a columnar oblique (a = 3.5 nm, b = 4.0 nm, γ = 82°,see table S2) at −60 °C.*

***Table S2**: Theoretical and observed values for $d_{col,obl}$ in **Si_7-OPV-Si_7**, using an angle of 82°. Calculated spacings are obtained using equation ES1. $d_{observed}$ is calculated from the scattering vector q through the equation d = 2π/q.*

h	*k*	$d_{calculated}$ (nm)	$d_{observed}$ (nm)
0	1	-	4.00
1	0	-	3.45
1	1	2.82	2.83
-1	1	2.45	2.47
0	2	2.00	2.07
2	0	1.73	1.72
2	1	1.67	1.60

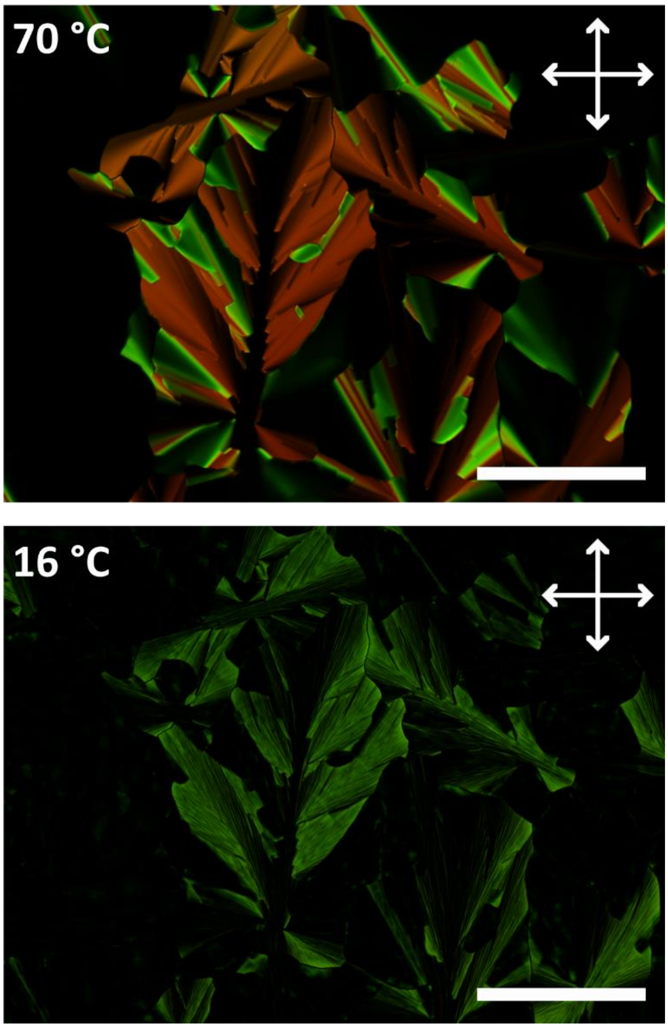

Figure S15: *POM image of* ***Si$_7$-OPV-Si$_7$*** *after slow cooling from its melt at various temperatures, under crossed polarizers. Scale bar indicates 200 µm. At 70 °C the Col$_h$ phase is observed, as well as the Col$_{obl}$ phase below the final thermal transition.*

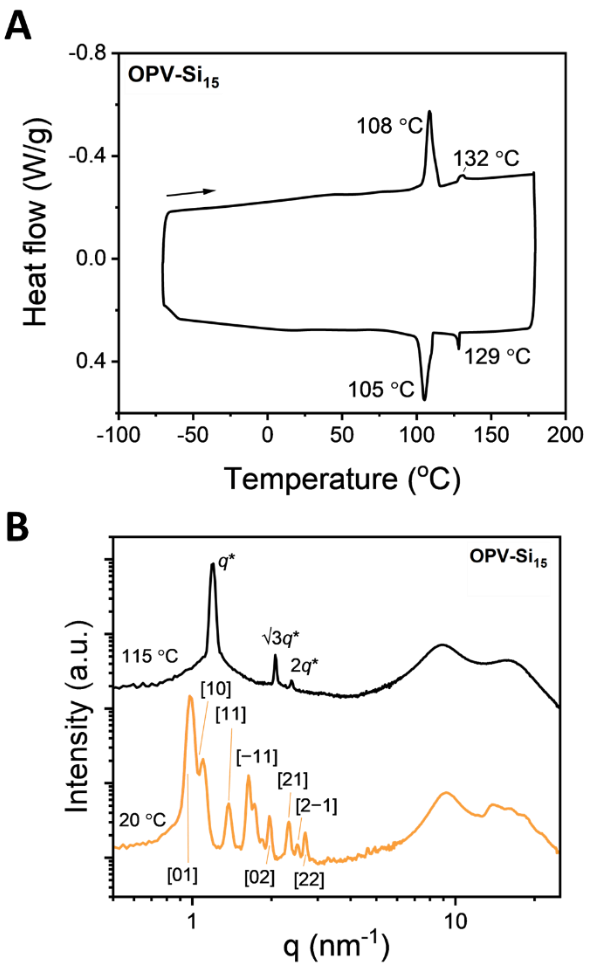

***Figure S16**: (A) Differential scanning calorimetry trace of **OPV-Si$_{15}$** of the second cycle with 10 °C min^{-1} showing the melting and crystallization peaks (exothermic down). The arrow indicates heating. (B) 1D transmission scattering profile of **OPV-Si$_{15}$** showing Col$_h$ packing at 115 °C and a Col$_{obl}$ at room temperature.*

***Table S3**: Theoretical and observed values for $d_{col,obl}$ in **OPV-Si$_{15}$**, using an angle of 83°. Calculated spacings are obtained using equation ES1. Resulting lattice constants are a = 5.8 nm and b = 6.5 nm. $d_{observed}$ is calculated from the scattering vector q through the equation d = 2π/q.*

h	***k***	**$d_{calculated}$ (nm)**	**$d_{observed}$ (nm)**
0	1	-	6.41
1	0	-	5.76
1	1	4.57	4.59
-1	1	4.05	3.93
0	2	3.21	3.21
2	1	2.76	2.70
2	-1	2.52	2.47
2	2	2.29	2.29

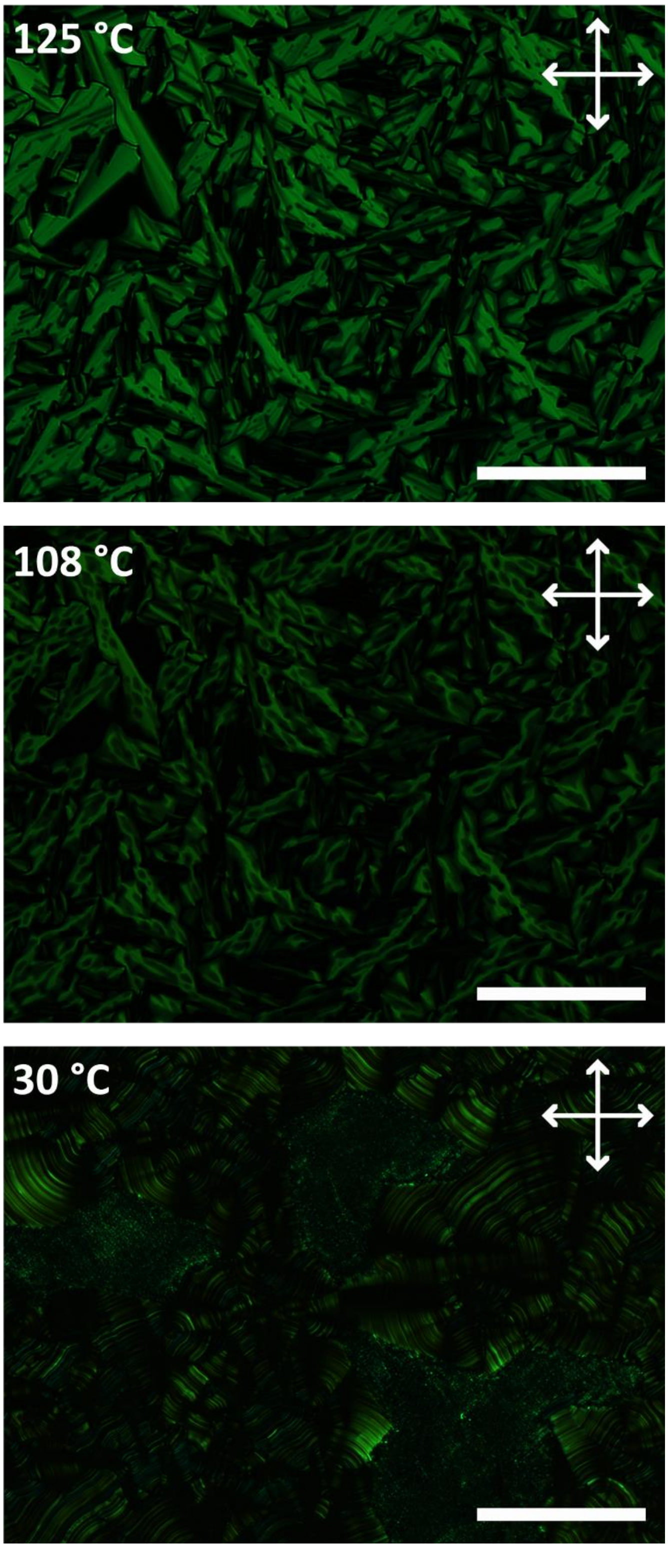

Figure S17: *POM image of* ***OPV-Si$_{15}$*** *after slow cooling from its melt at various temperatures, under crossed polarizers. Scale bar indicates 100 µm. At 125 °C the Col$_h$ phase is observed, that slowly darkens around the lowest T$_{exo}$ (~108 °C), after which birefringence is restored when the Col$_{obl}$ phase is crystallized.*

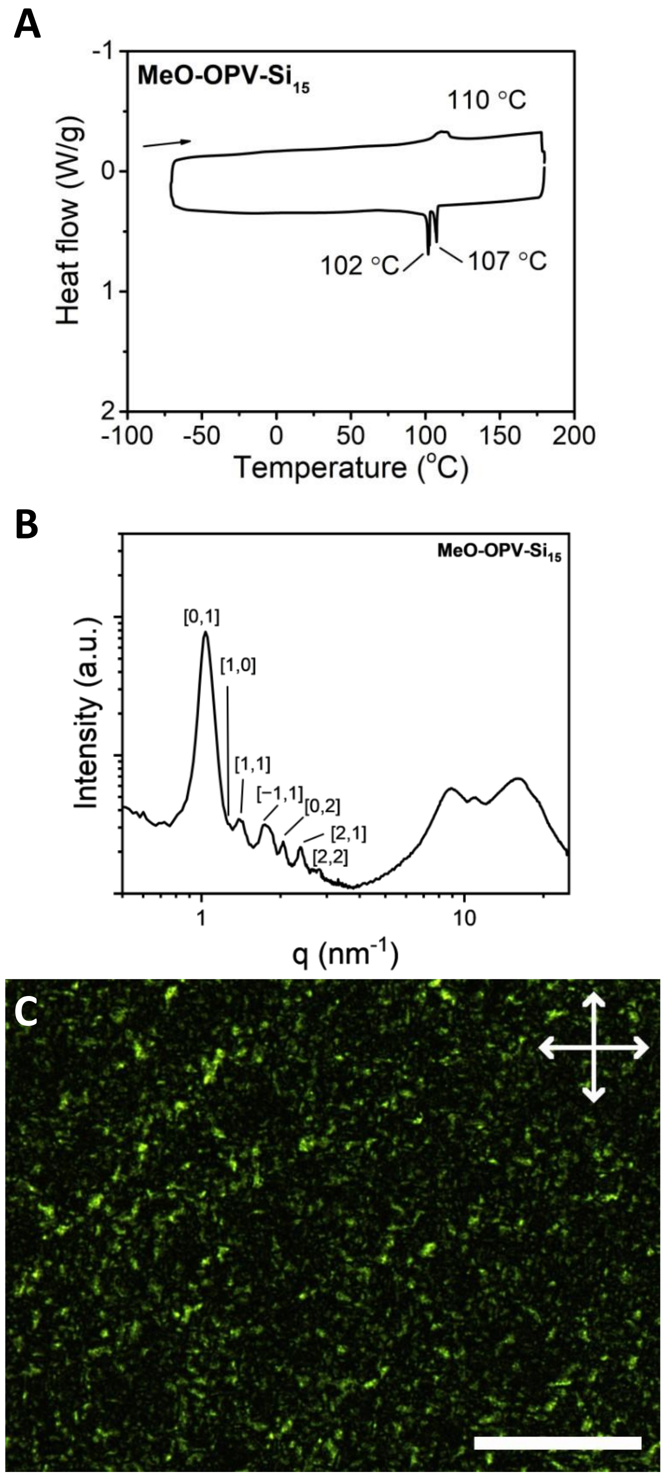

***Figure S18**: Characterization of **MeO-OPV-Si15**. (A) Differential scanning calorimetry traces of the second cycle with 10 °C min^{-1} showing the melting and crystallization peaks (exothermic down). The arrow indicates heating. (B) 1D transmission scattering profile of **MeO-OPV-Si15** showing Col$_{obl}$ packing at room temperature. (C) POM image at 90 °C after slow cooling from its melt, under crossed polarizers. Scale bar indicates 100 µm.*

***Table S4**: Theoretical and observed values for $d_{col,obl}$ in **MeO-OPV-Si$_{15}$**, using an angle of 81°. Calculated spacings are obtained using equation ES1. Resulting lattice constants are a = 5.7 nm and b = 6.1 nm. $d_{observed}$ is calculated from the scattering vector q through the equation d = 2π/q.*

h	*k*	$d_{calculated}$ (nm)	$d_{observed}$ (nm)
0	1	-	6.04
1	0	-	5.61
1	1	4.47	4.46
−1	1	3.82	3.70
0	2	3.02	3.08
2	1	2.71	2.64
2	2	2.24	2.21

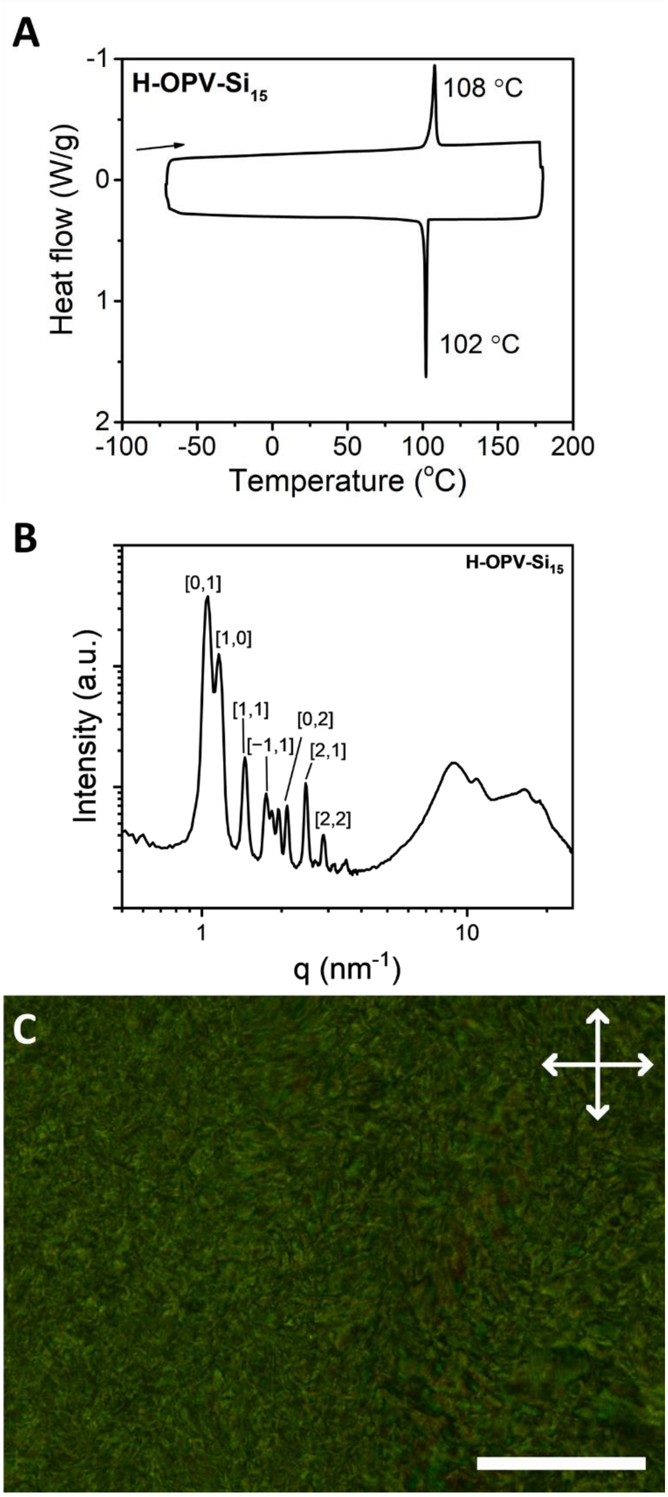

Figure S19: *Characterization of **H-OPV-Si15**. (A) Differential scanning calorimetry traces of the second cycle with 10 °C min^{-1} showing the melting and crystallization peaks (exothermic down). The arrow indicates heating. (B) 1D transmission scattering profile of H-**OPV-Si15** showing Col$_{obl}$ packing at room temperature. (C) POM image at 90 °C after slow cooling from its melt, under crossed polarizers. Scale bar indicates 100 µm.*

Table S5*: Theoretical and observed values for $d_{col,obl}$ in **H-OPV-Si$_{15}$**, using an angle of 82°. Calculated spacings are obtained using equation ES1. Resulting lattice constants are a = 5.5 nm and b = 6.1 nm. $d_{observed}$ is calculated from the scattering vector q through the equation d = 2π/q.*

h	***k***	**$d_{calculated}$ (nm)**	**$d_{observed}$ (nm)**
0	1	-	6.04
1	0	-	5.42
1	1	4.34	4.30
−1	1	3.78	3.63
0	2	3.02	2.99
2	1	2.61	2.55
2	2	2.17	2.18

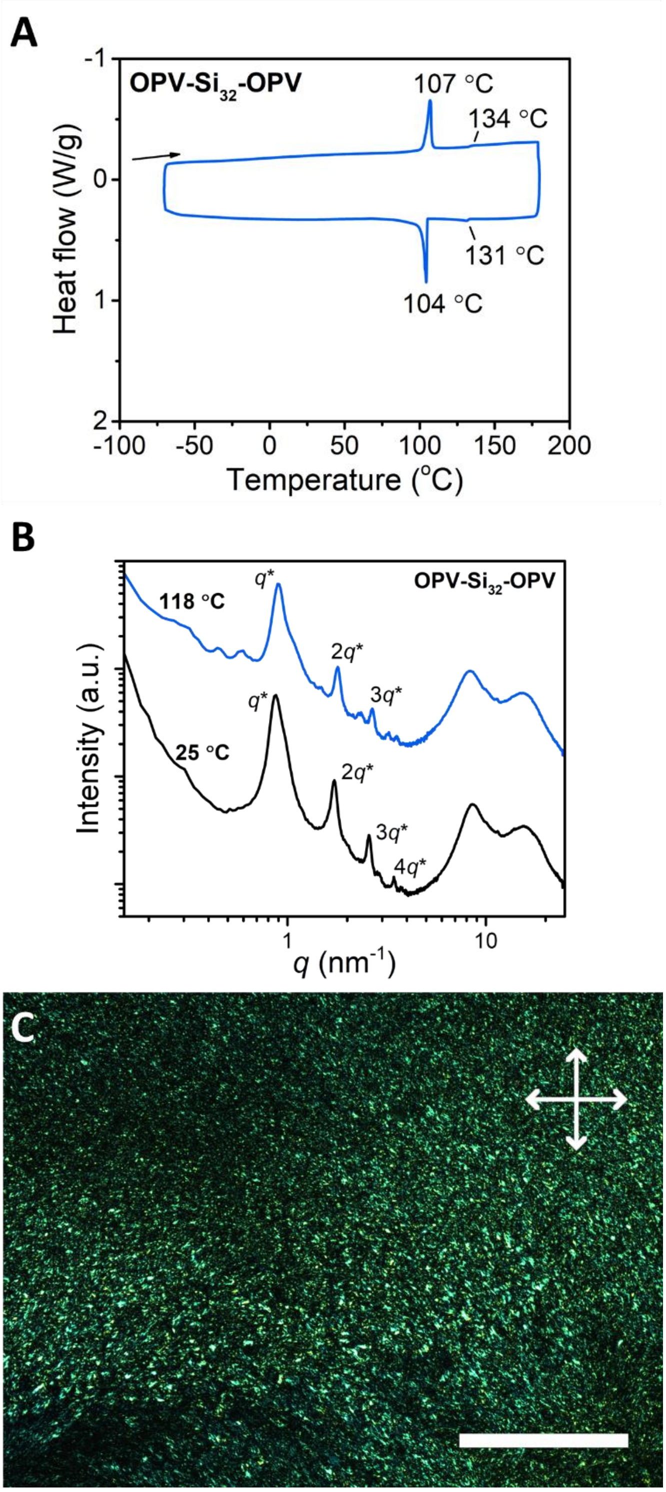

Figure S20: *Characterization of* ***OPV-Si₃₂-OPV****. (A) Differential scanning calorimetry traces of the second cycle with 10 °C min^{-1} showing the melting and crystallization peaks (exothermic down). The arrow indicates heating. (B) 1D transmission scattering profile showing lamellar order (d_{lam} = 7.0 nm) at 118 °C and a lamellar phase (d_{lam} = 7.2 nm) at room temperature.*

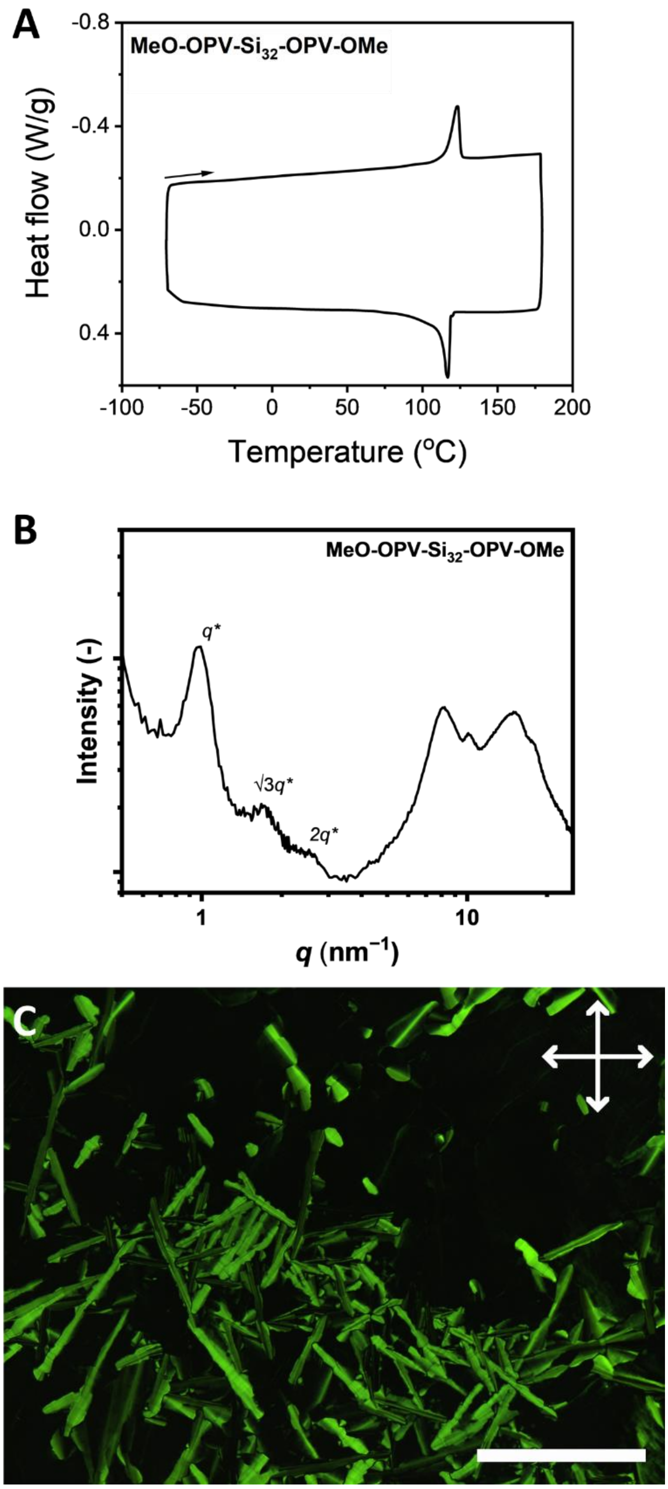

Figure 21: *Characterization of **MeO-OPV-Si₃₂-OPV-OMe**. (A) Differential scanning calorimetry traces of the second cycle with 10 °C min^{-1} showing the melting and crystallization peaks (exothermic down). The arrow indicates heating. (B) 1D transmission scattering profile at room temperature showing columnar order (d_{col} = 6.5 nm). (C) POM image at room temperature after slow cooling from its melt, under with crossed polarizers. Scale bar indicates 100 µm.*

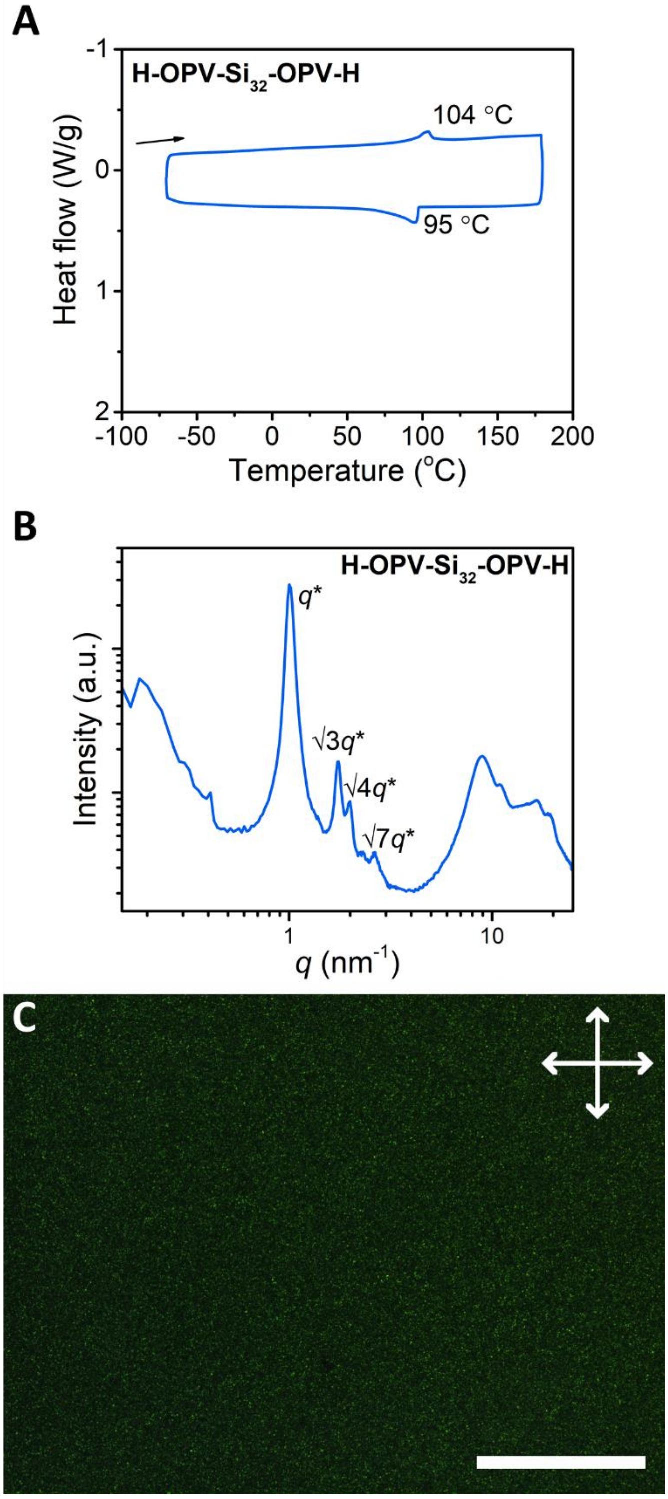

***Figure S22**: Characterization of **H-OPV-Si$_{32}$-OPV-H**. (A) Differential scanning calorimetry traces of the second cycle with 10 °C min^{-1} showing the melting and crystallization peaks (exothermic down). The arrow indicates heating. (B) 1D transmission scattering profile at room temperature showing columnar order (d_{col} = 6.2 nm). (C) POM image at room temperature after slow cooling from its melt, under with crossed polarizers. Scale bar indicates 100 µm.*

4. NMR Spectra

4.1 DPA derivatives

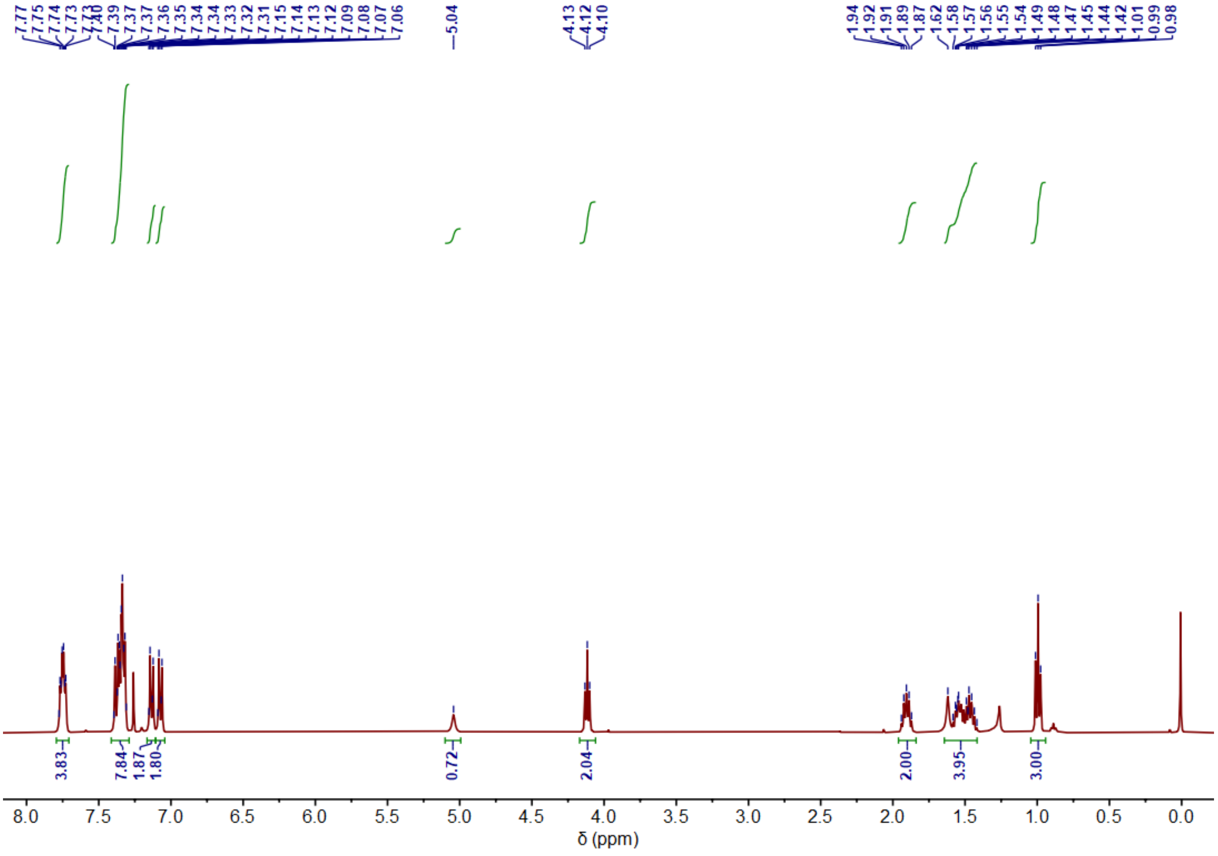

Figure S23: *^{1}H NMR spectrum (400 MHz, $CDCl_3$) of* ***4-(10-(4-(pentyloxy)phenyl)anthracen-9-yl)phenol (1)***

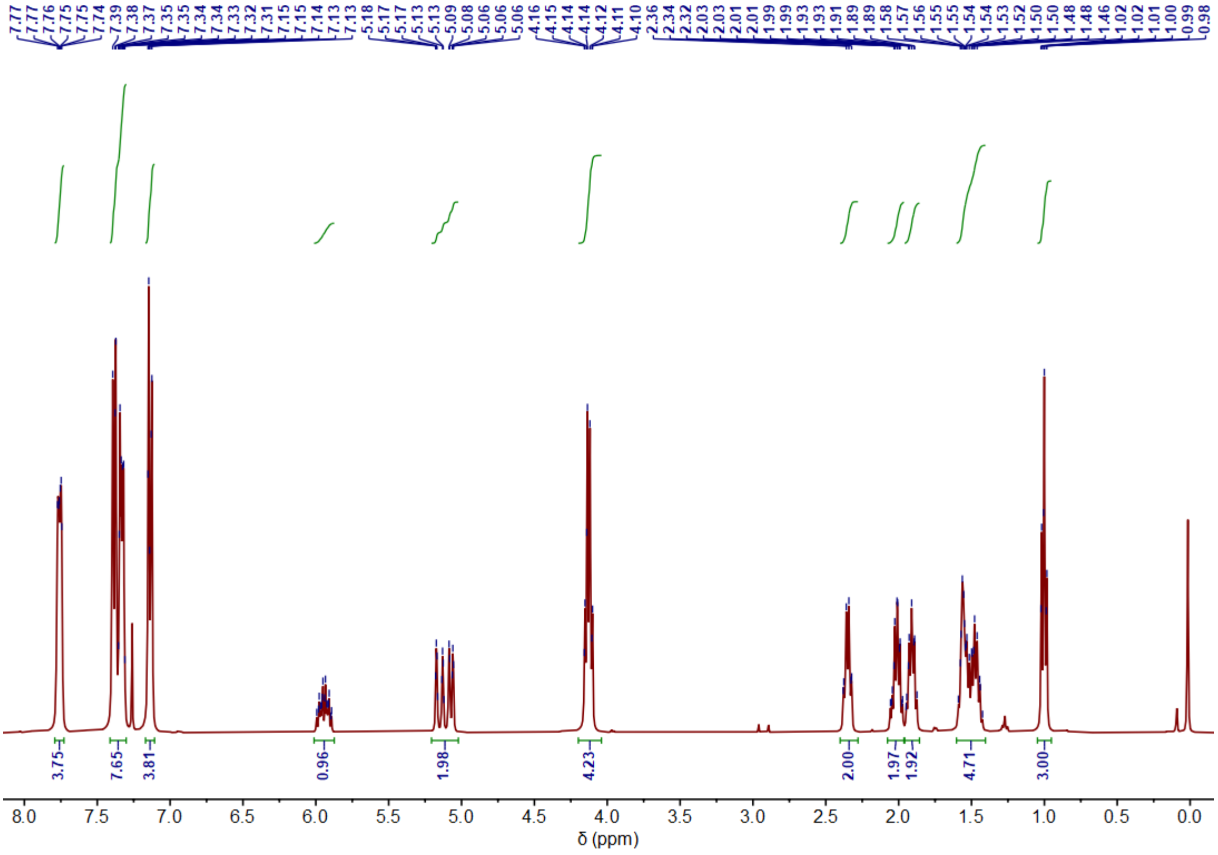

Figure S24**: ^{1}H NMR spectrum (400 MHz, $CDCl_3$) of **9-(4-(pent-4-en-1-yloxy)phenyl)-10-(4-(pentyloxy)phenyl)anthracene (2)

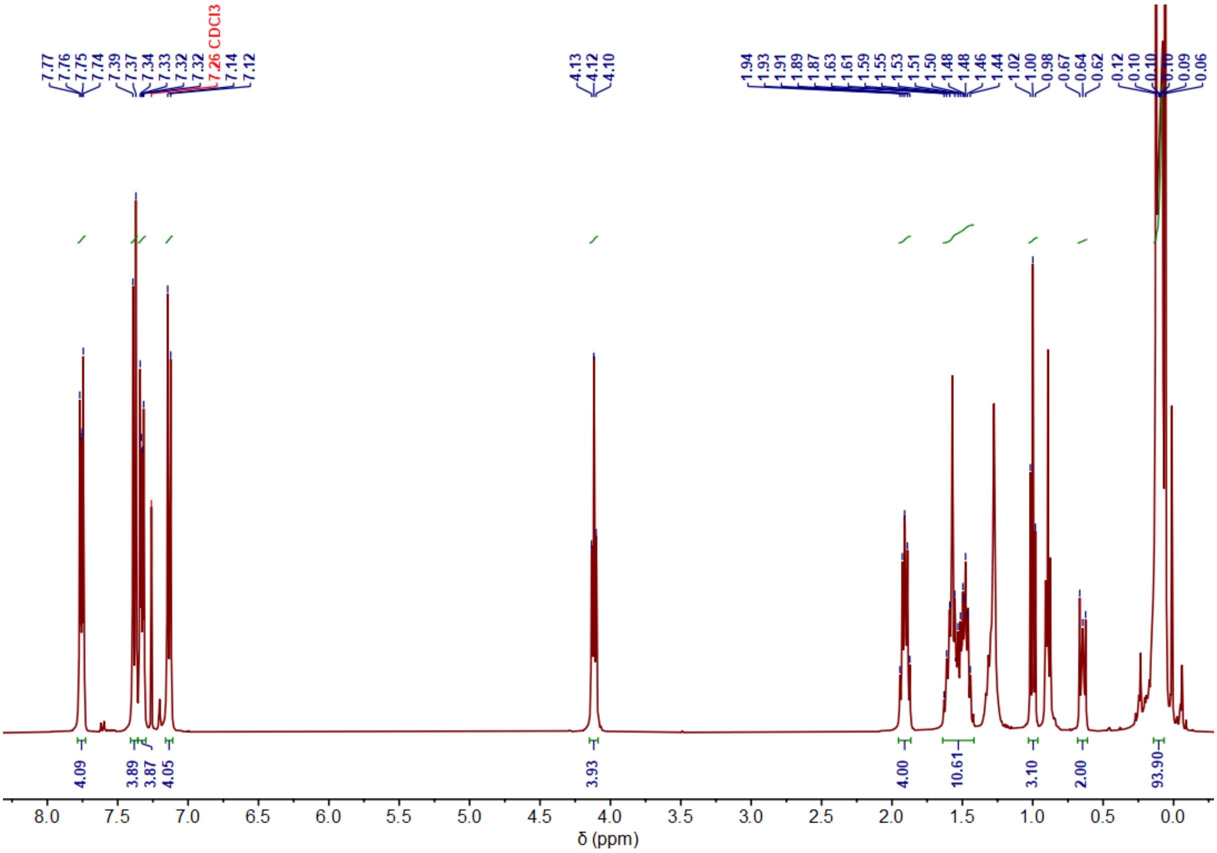

Figure S25**: ^{1}H NMR spectrum (400 MHz, $CDCl_3$) of **DPA-Si$_{15}$

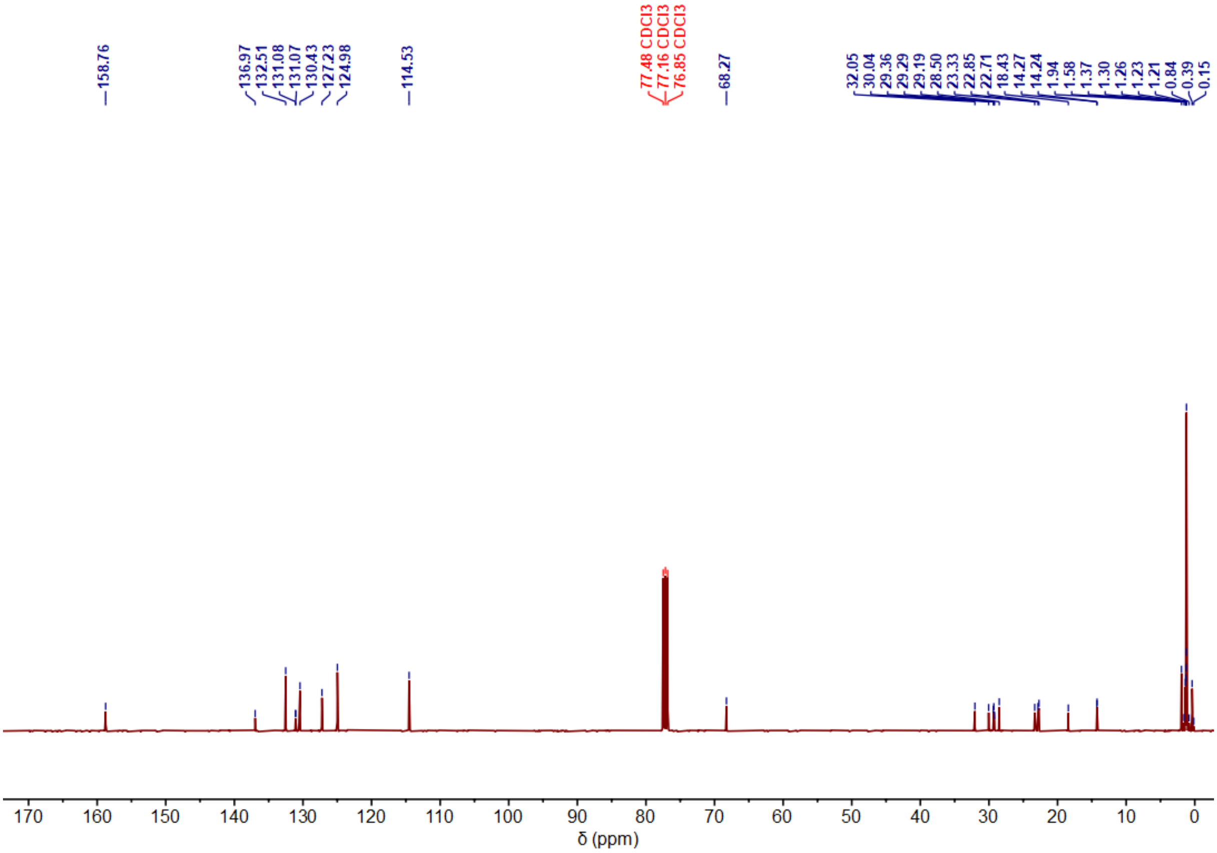

Figure S26**: ^{13}C NMR (100 MHz, $CDCl_3$) of **DPA-Si$_{15}$

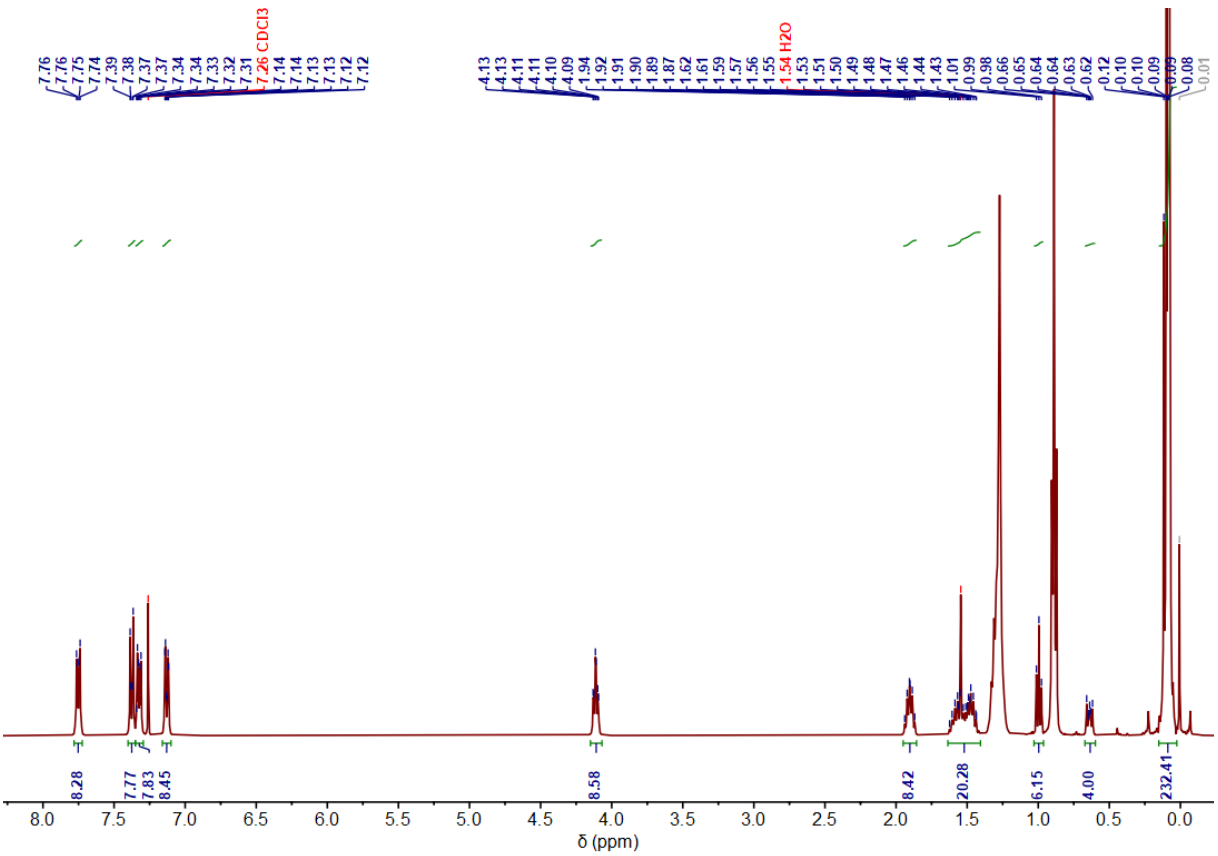

Figure S27: *^{1}H NMR spectrum (400 MHz, CDCl$_3$) of* ***DPA-Si$_{32}$-DPA***

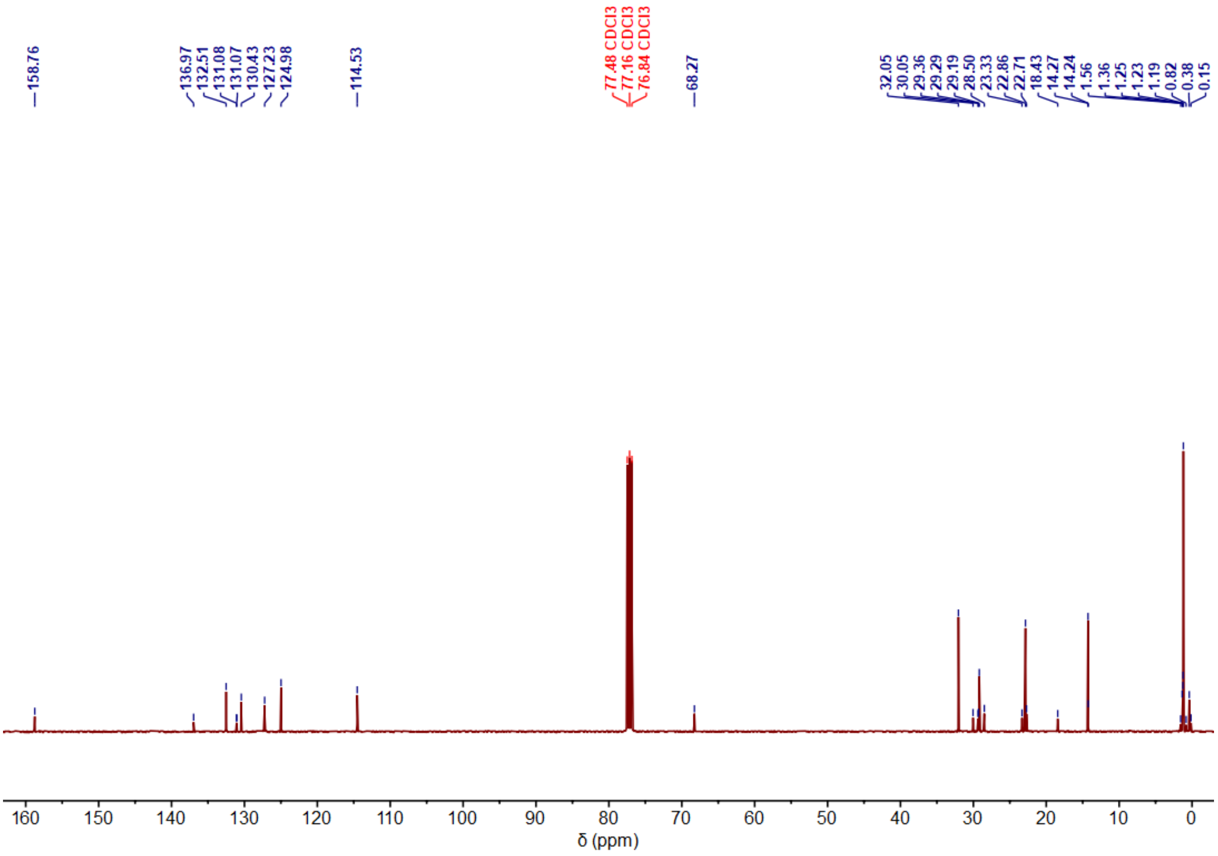

Figure S28: *^{13}C NMR (100 MHz, CDCl$_3$) of* ***DPA-Si$_{32}$-DPA***

4.2 AQ derivatives

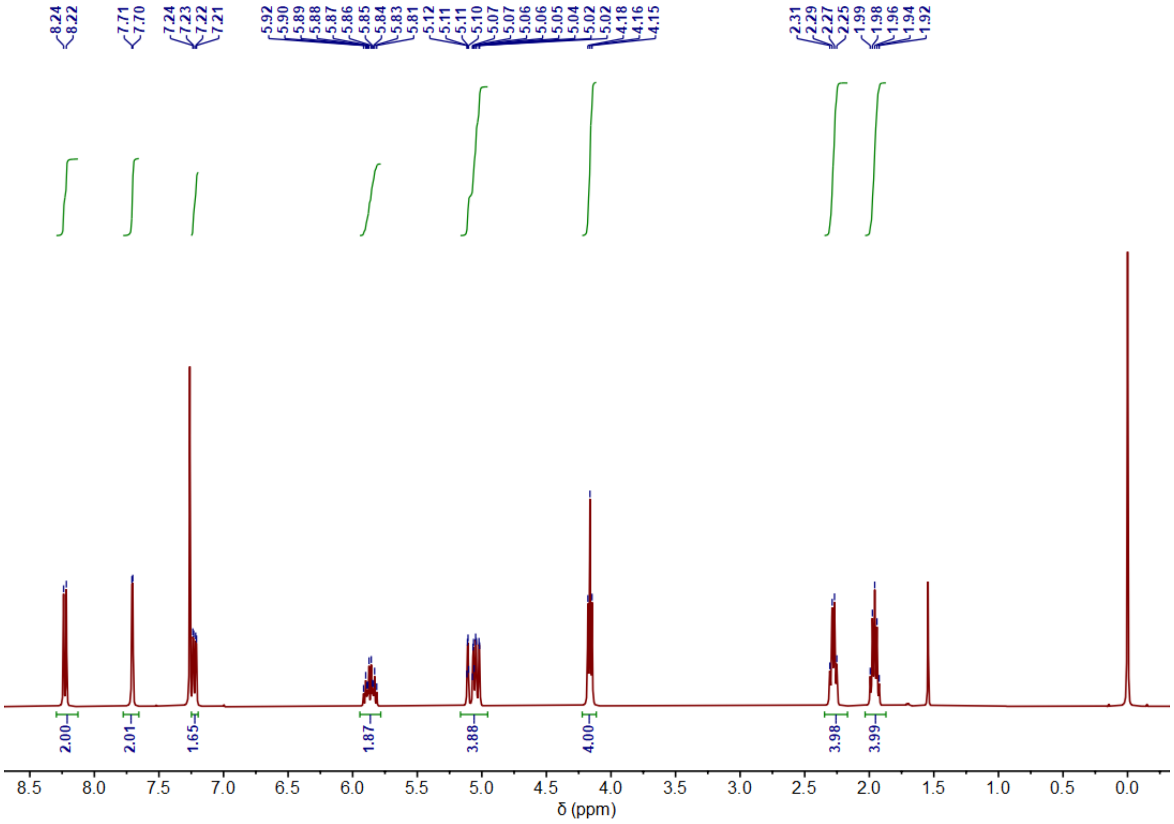

Figure S29: *^{1}H NMR (400 MHz, $CDCl_3$) of* ***2-(pent-4-en-1-yloxy)-6-(pent-4-en-1-yloxy)anthracene-9,10-dione (3)***

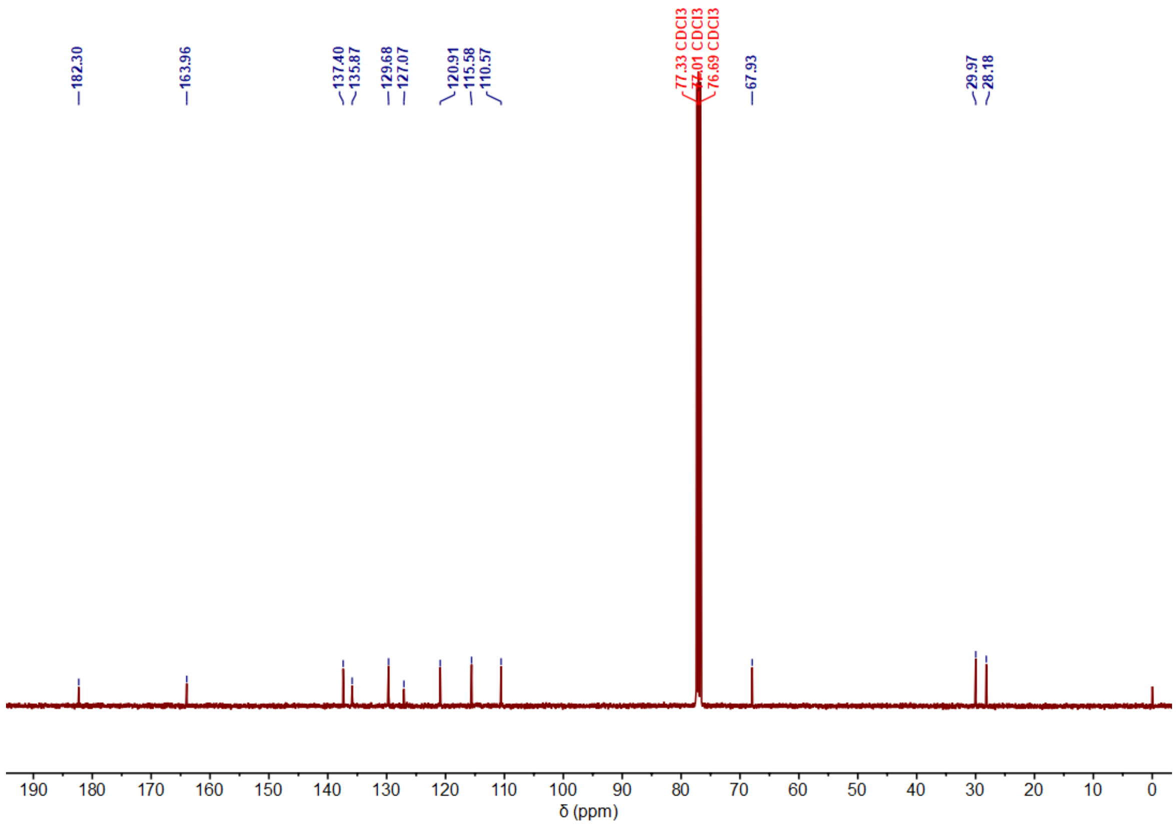

Figure S30: *^{13}C NMR (100 MHz, $CDCl_3$) of* ***2-(pent-4-en-1-yloxy)-6-(pent-4-en-1-yloxy)anthracene-9,10-dione (3)***

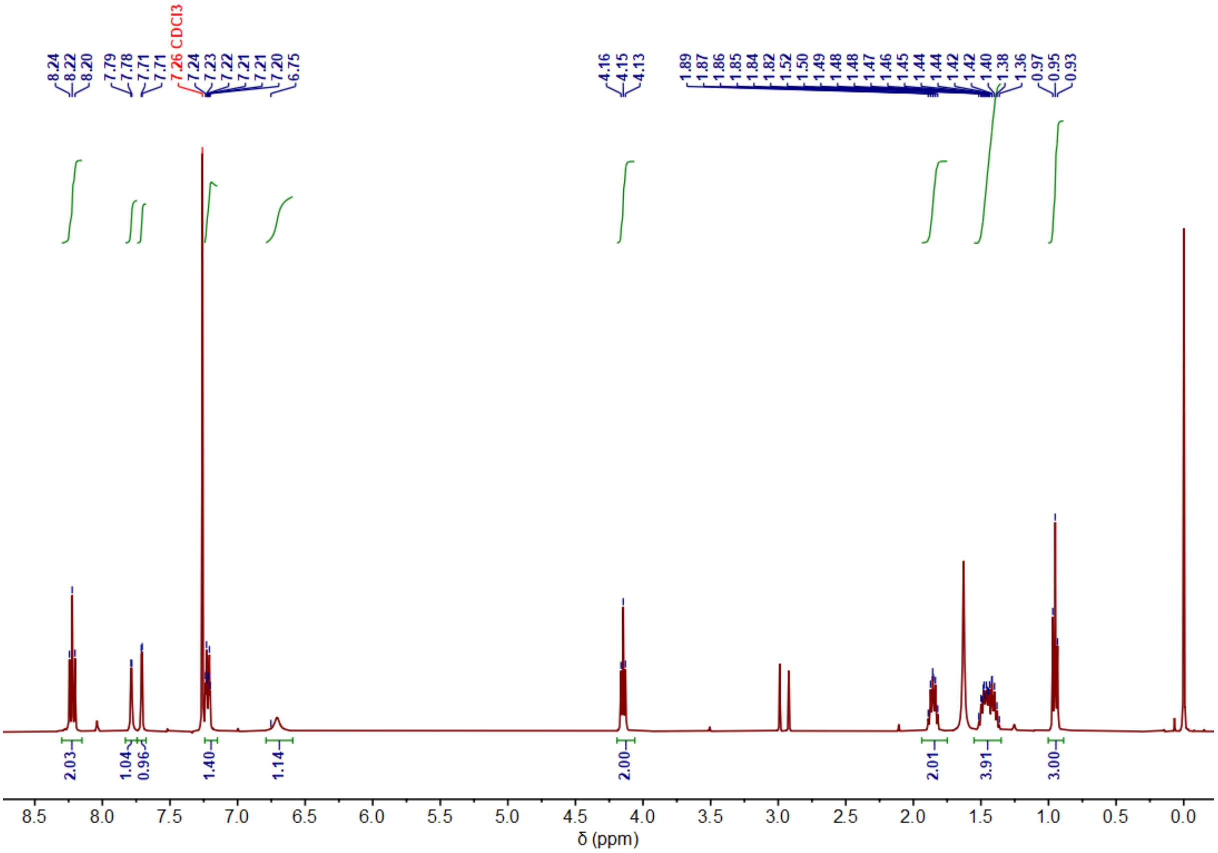

Figure S31: *^{1}H NMR (400 MHz, $CDCl_3$) of* ***2-hydroxy-6-(pentyloxy)anthracene-9,10-dione (4)***

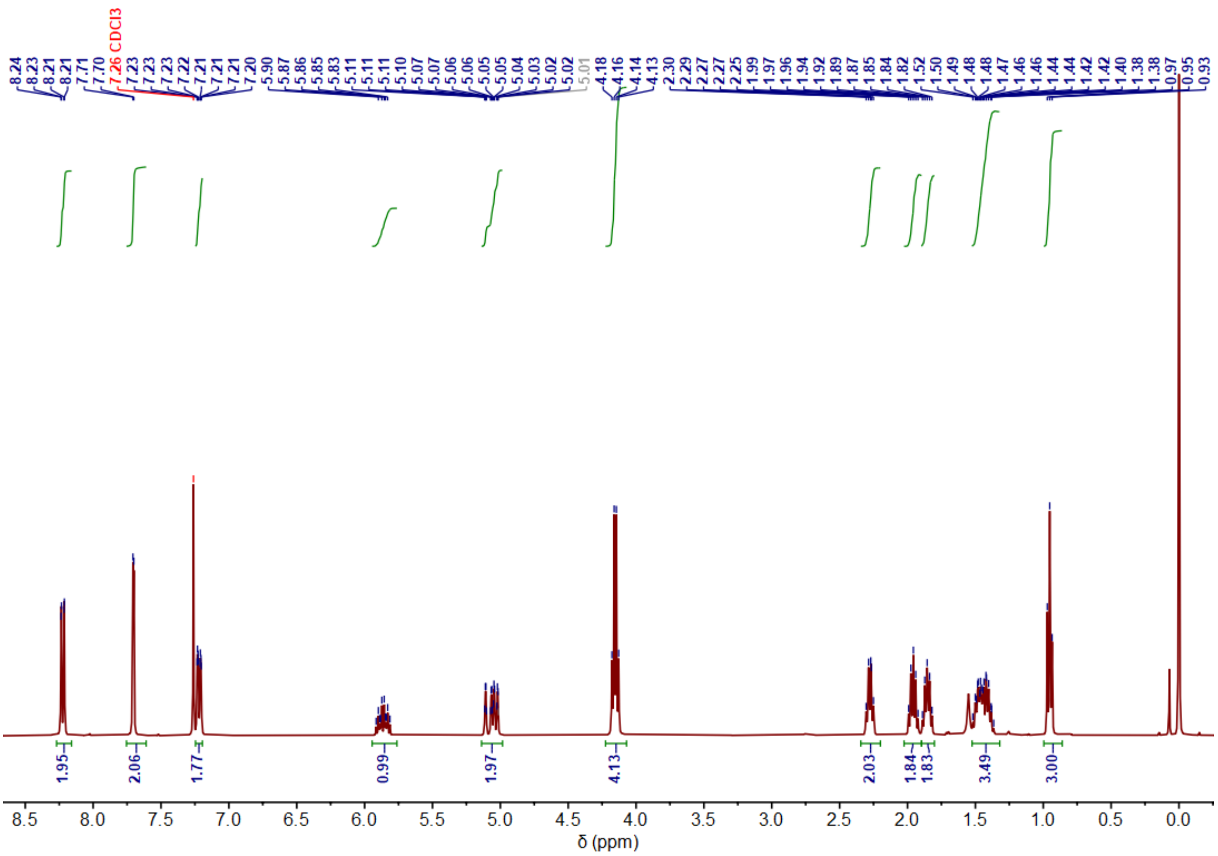

Figure S32: *^{1}H NMR (400 MHz, $CDCl_3$) of* ***2-(pent-4-en-1-yloxy)-6-(pentyloxy)anthracene-9,10-dione (5)***

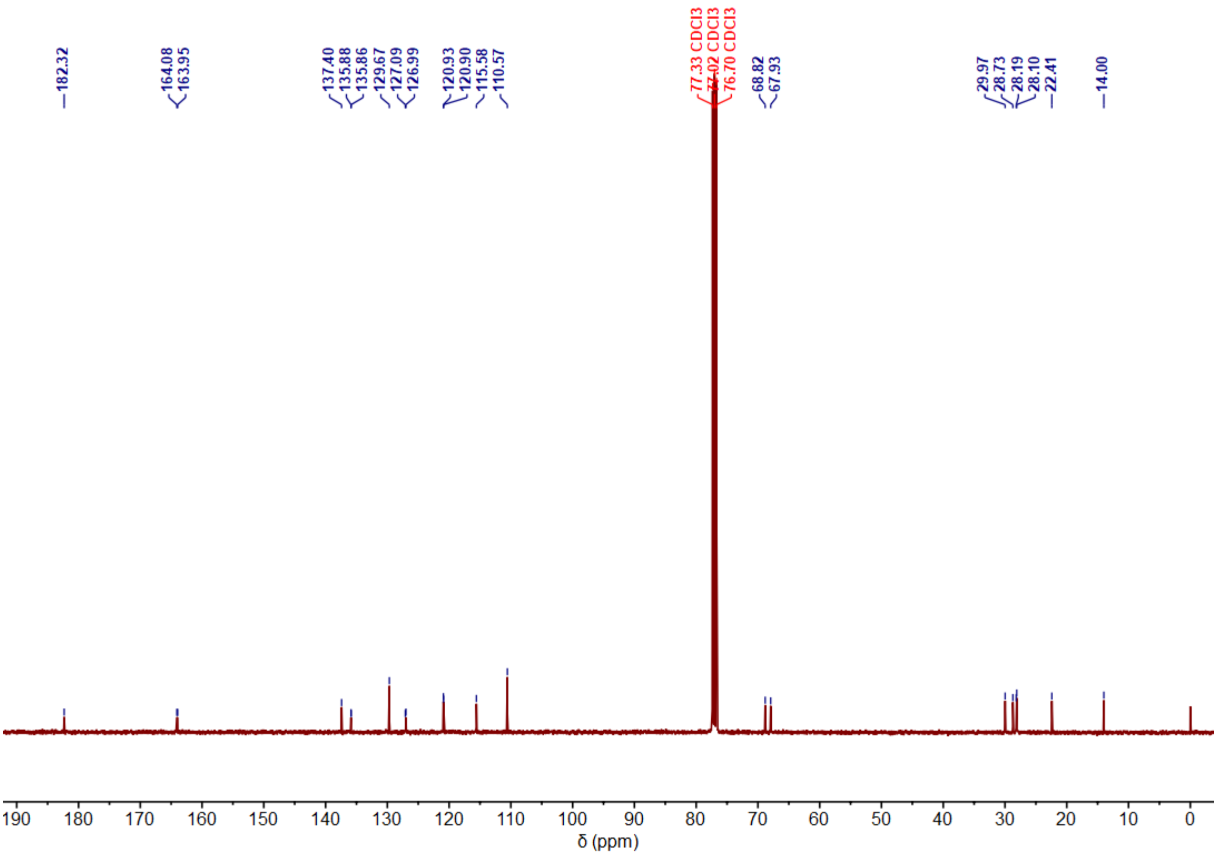

Figure S33: *^{13}C NMR (100 MHz, $CDCl_3$) of* ***2-(pent-4-en-1-yloxy)-6-(pentyloxy)anthracene-9,10-dione (5)***

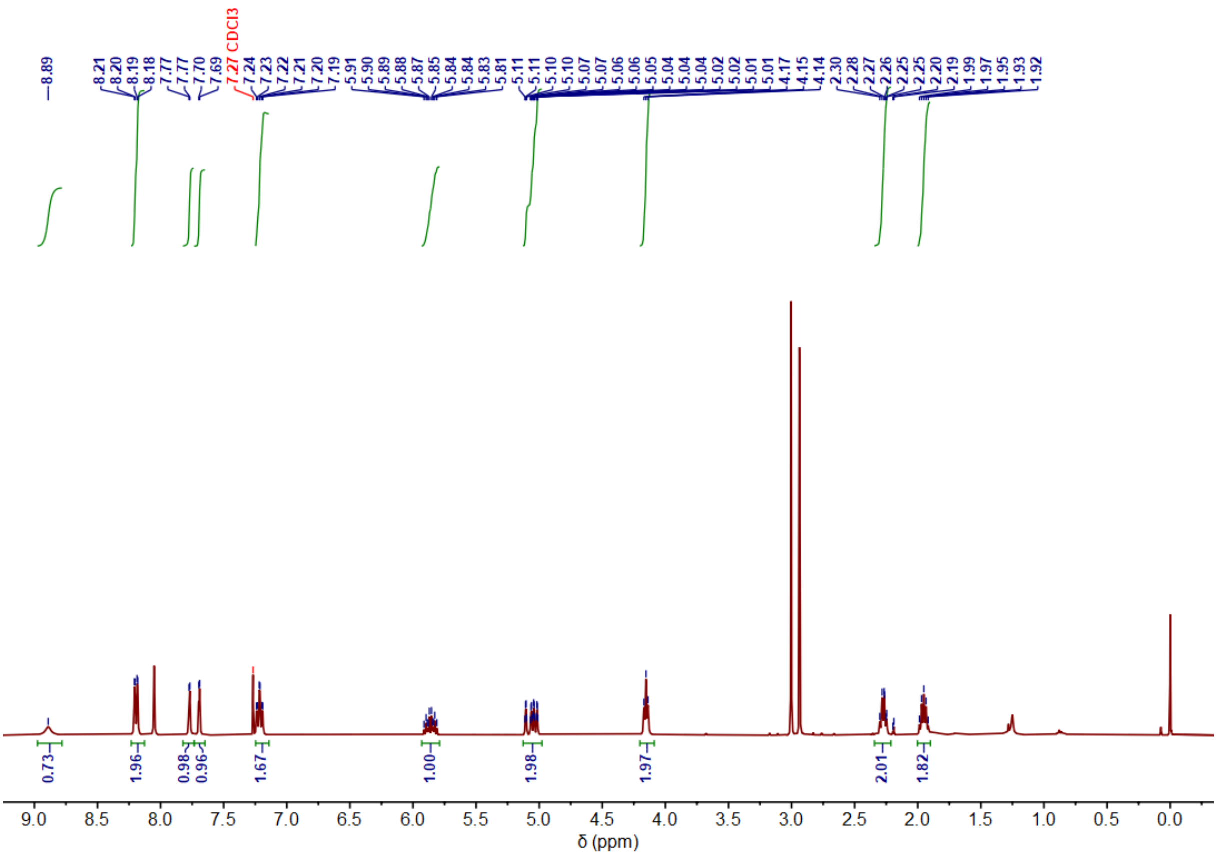

Figure S34**: ^{1}H NMR (400 MHz, $CDCl_3$) of **2-hydroxy-6-(pent-4-en-1yloxy)anthracene-9,10-dione (6)

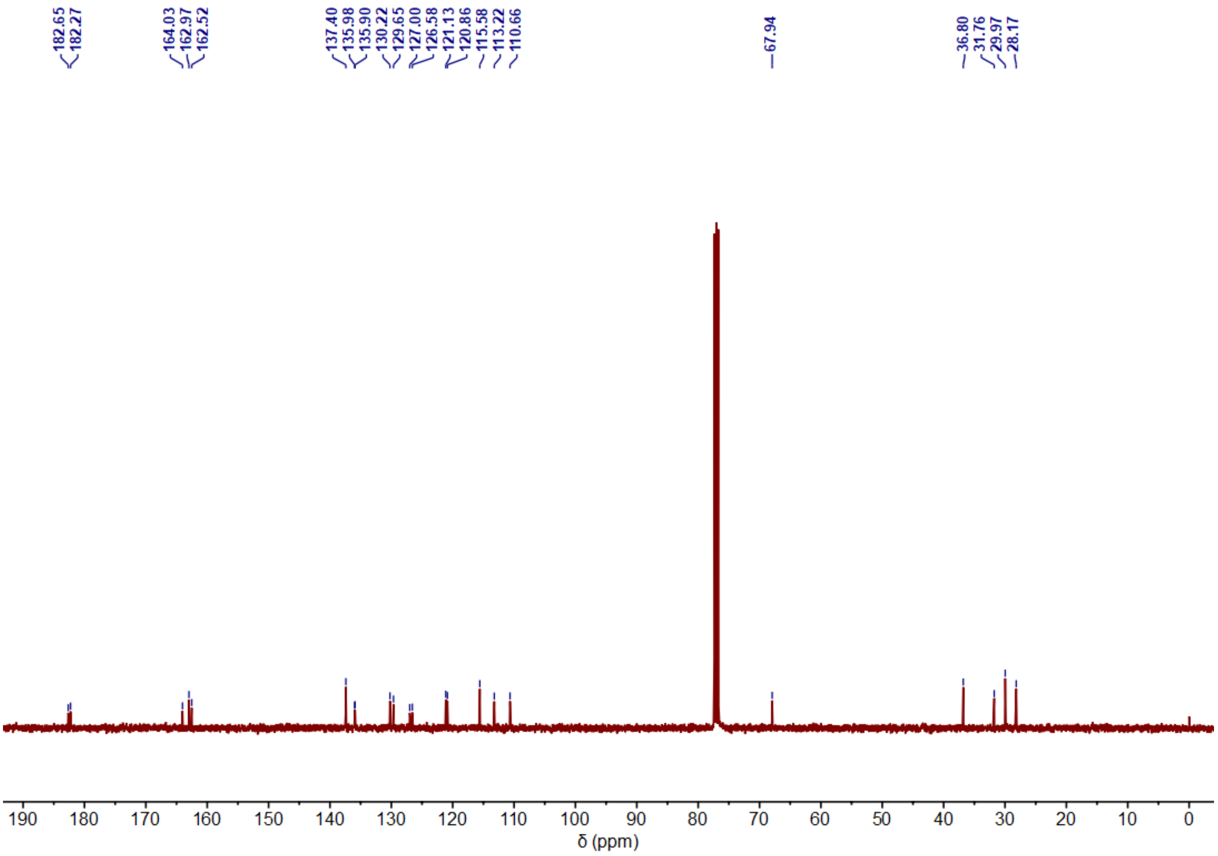

Figure S35**: ^{13}C NMR (100 MHz, $CDCl_3$) of **2-hydroxy-6-(pent-4-en-1yloxy)anthracene-9,10-dione (6)

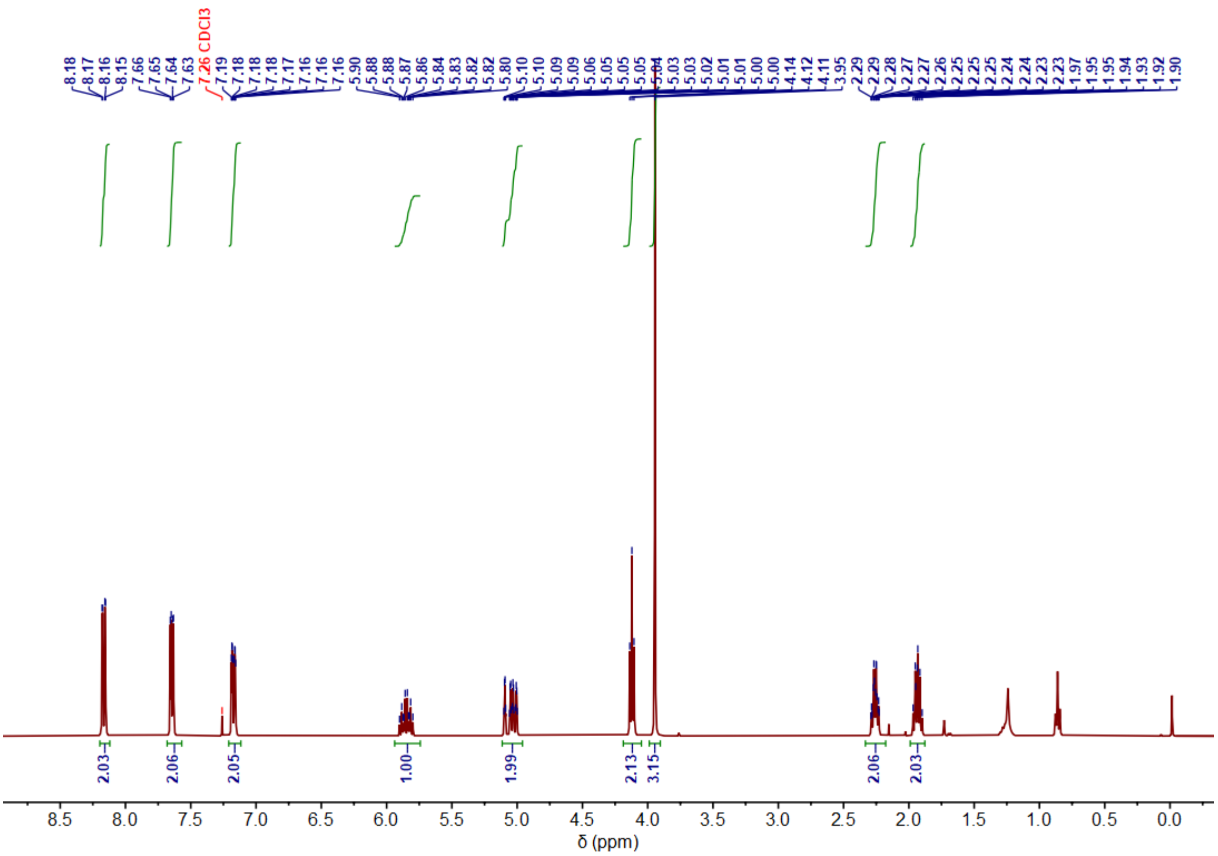

Figure S36: *^{1}H NMR (400 MHz, $CDCl_3$) of* ***2-methoxy-6-(pent-4-en-1yloxy)anthracene-9,10-dione (7)***

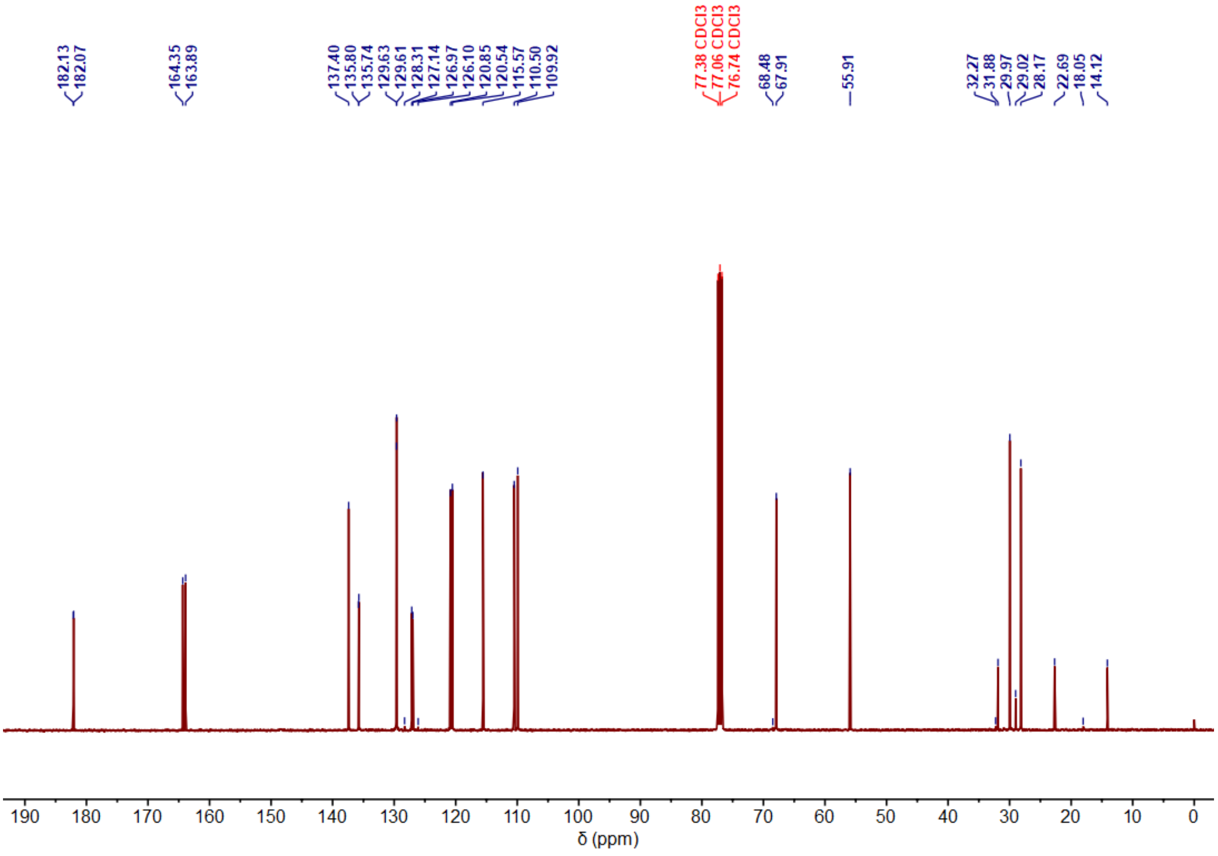

Figure S37: *^{13}C NMR (100 MHz, $CDCl_3$) of* ***2-methoxy-6-(pent-4-en-1yloxy)anthracene-9,10-dione (7)***

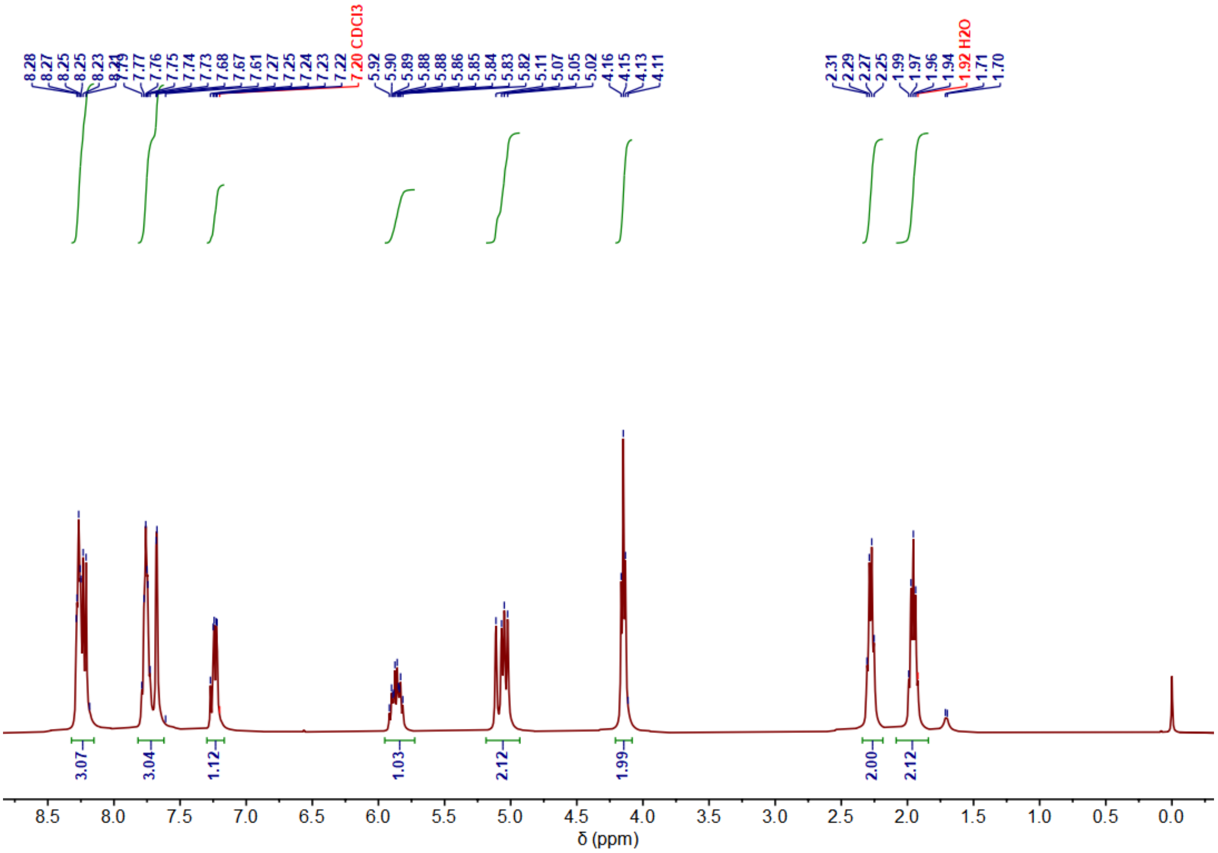

Figure S38**: ^{1}H NMR (400 MHz, $CDCl_3$) of **2-(pent-4-en-1yloxy)anthracene-9,10-dione (8)

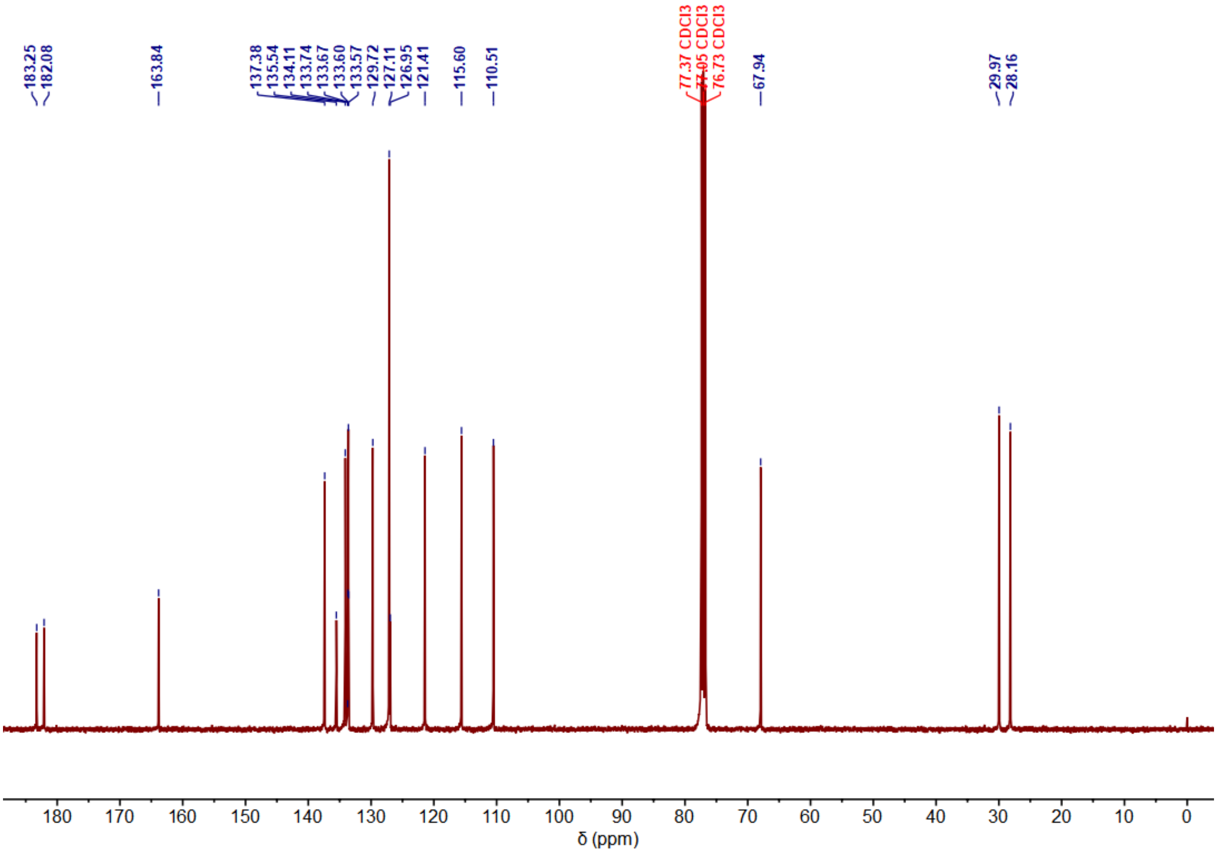

Figure S39**: ^{13}C NMR (100 MHz, $CDCl_3$) of **2-(pent-4-en-1yloxy)anthracene-9,10-dione (8)

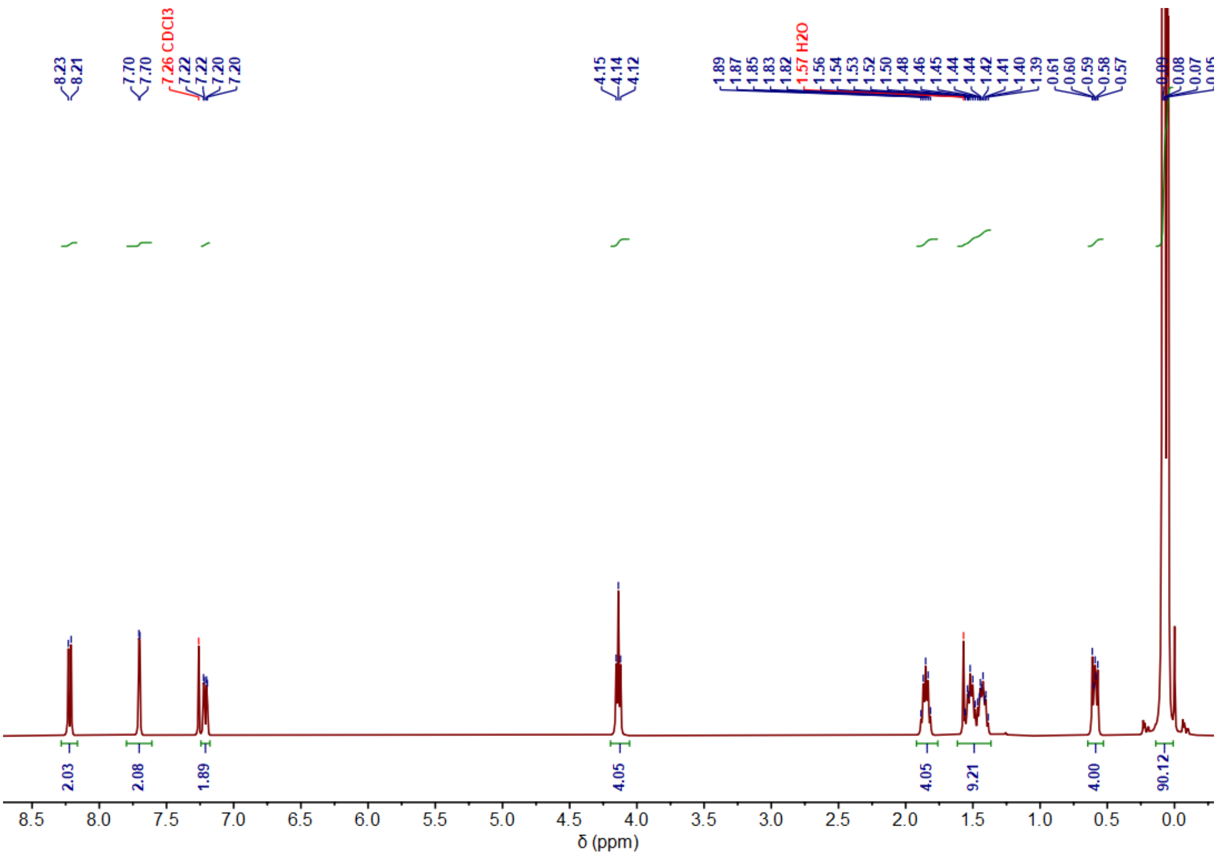

Figure S40: *^{1}H NMR (400 MHz, CDCl₃) of* ***Si₇-AQ-Si₇***

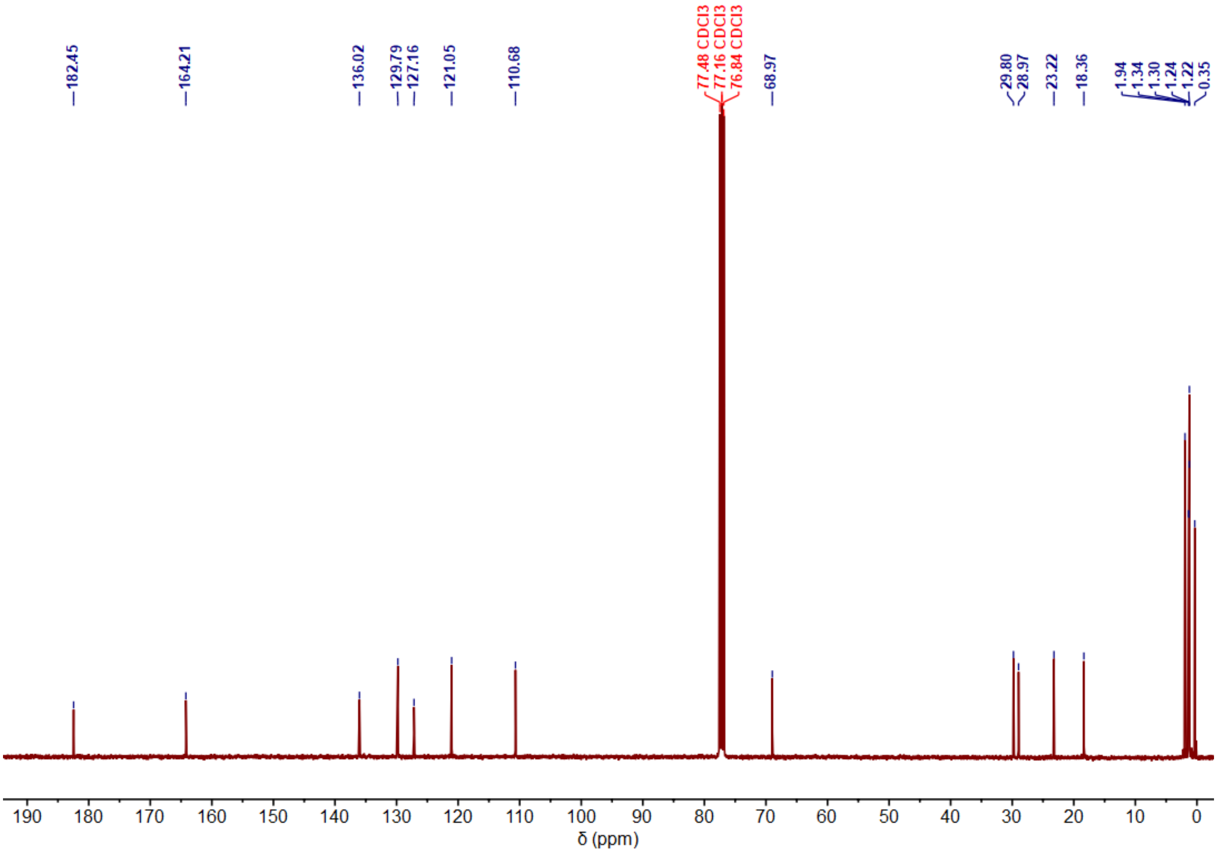

Figure S41: *^{13}C NMR (100 MHz, CDCl₃) of* ***Si₇-AQ-Si₇***

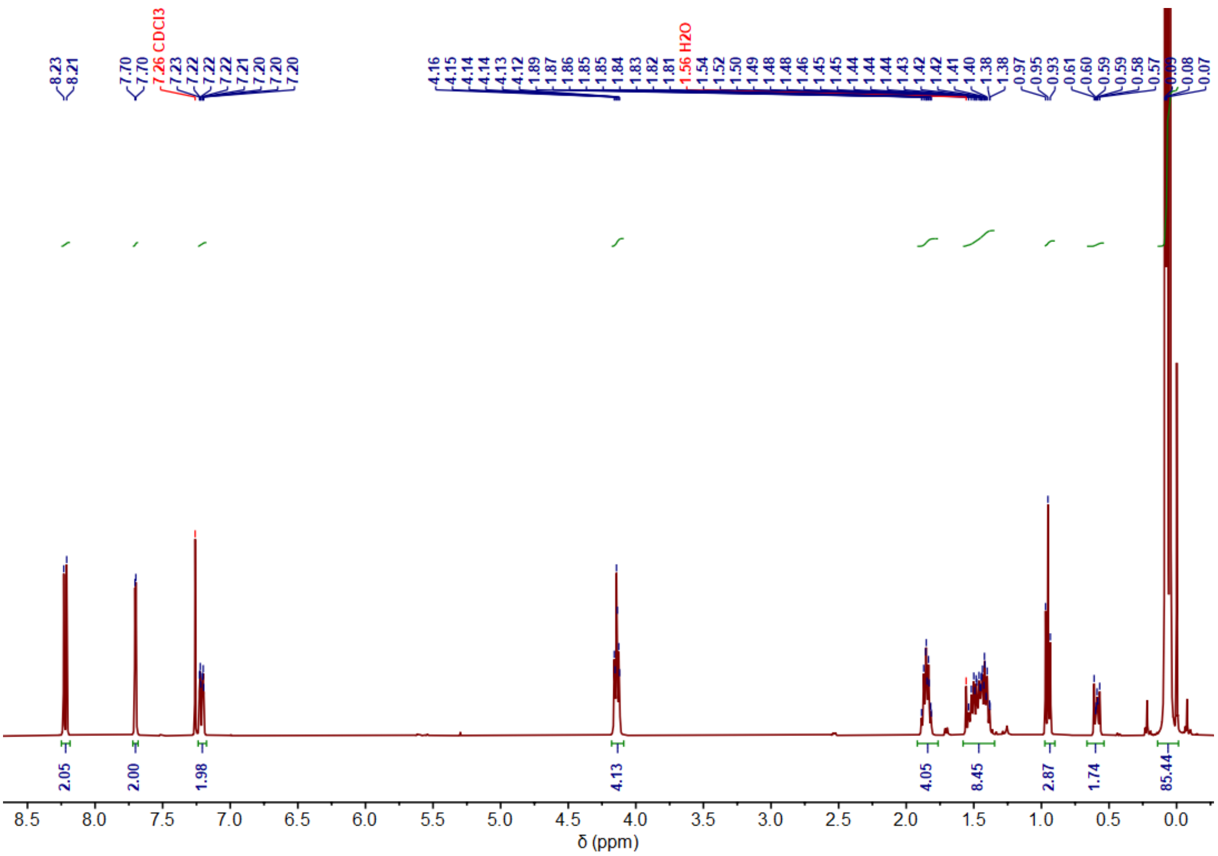

Figure S42: ^{1}H NMR (400 MHz, $CDCl_3$) of **Pent-AQ-Si15**

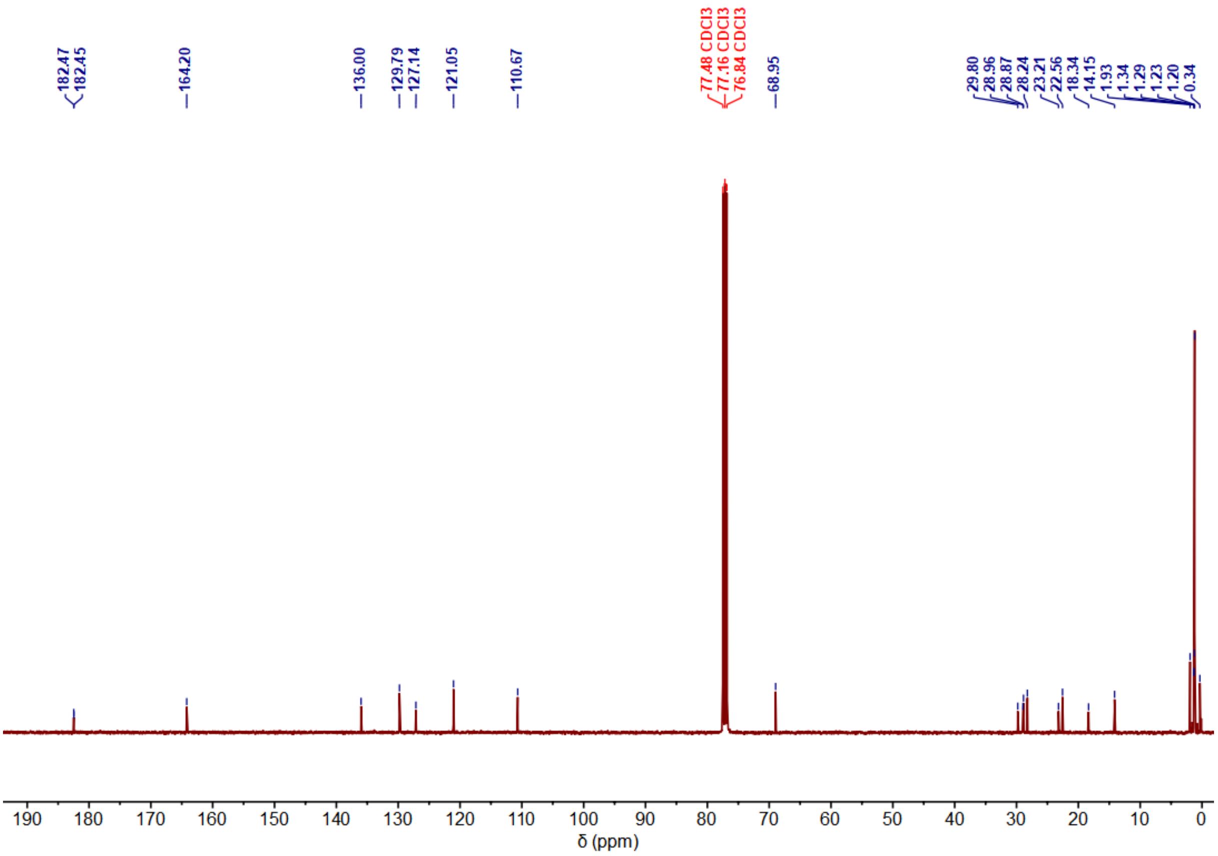

Figure S43: ^{13}C NMR (100 MHz, $CDCl_3$) of **Pent-AQ-Si15**

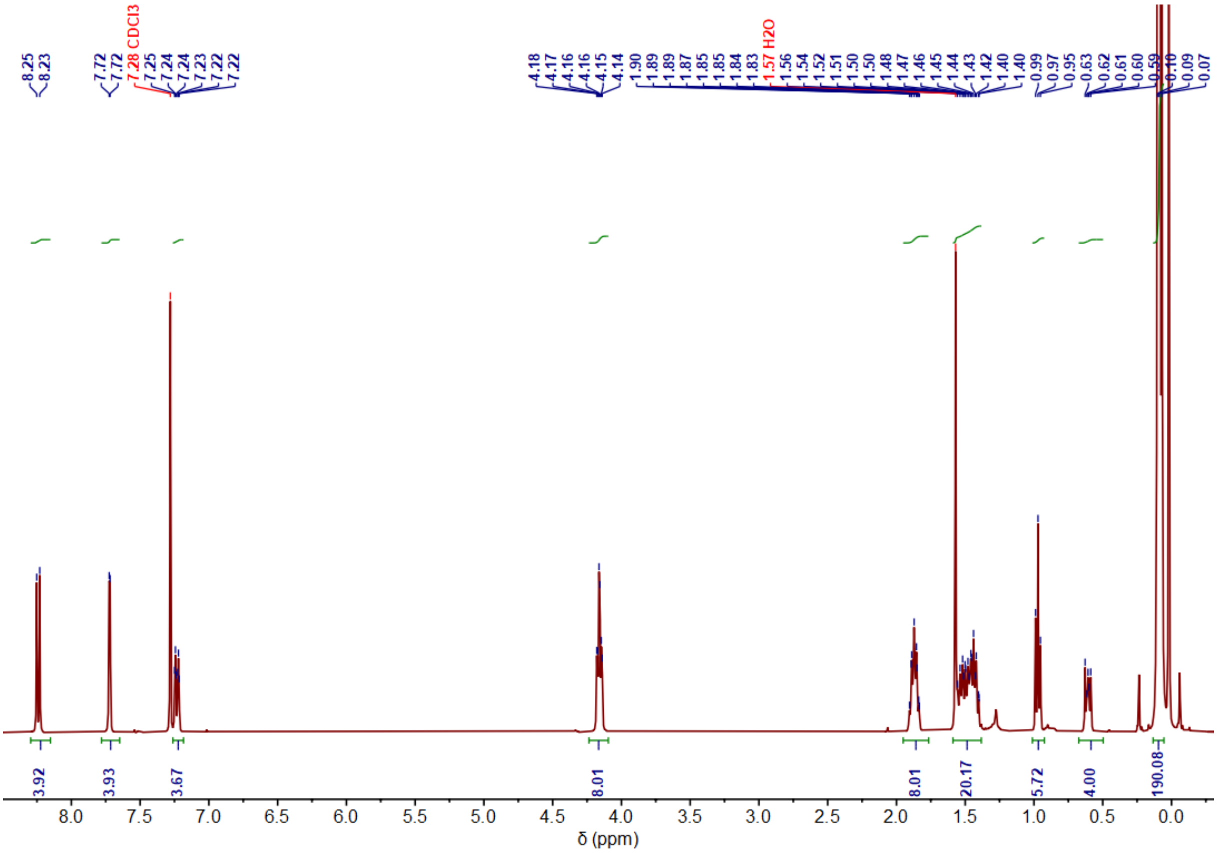

Figure S44: ^{1}H NMR (400 MHz, $CDCl_3$) of **Pent-AQ-Si32-AQ-Pent**

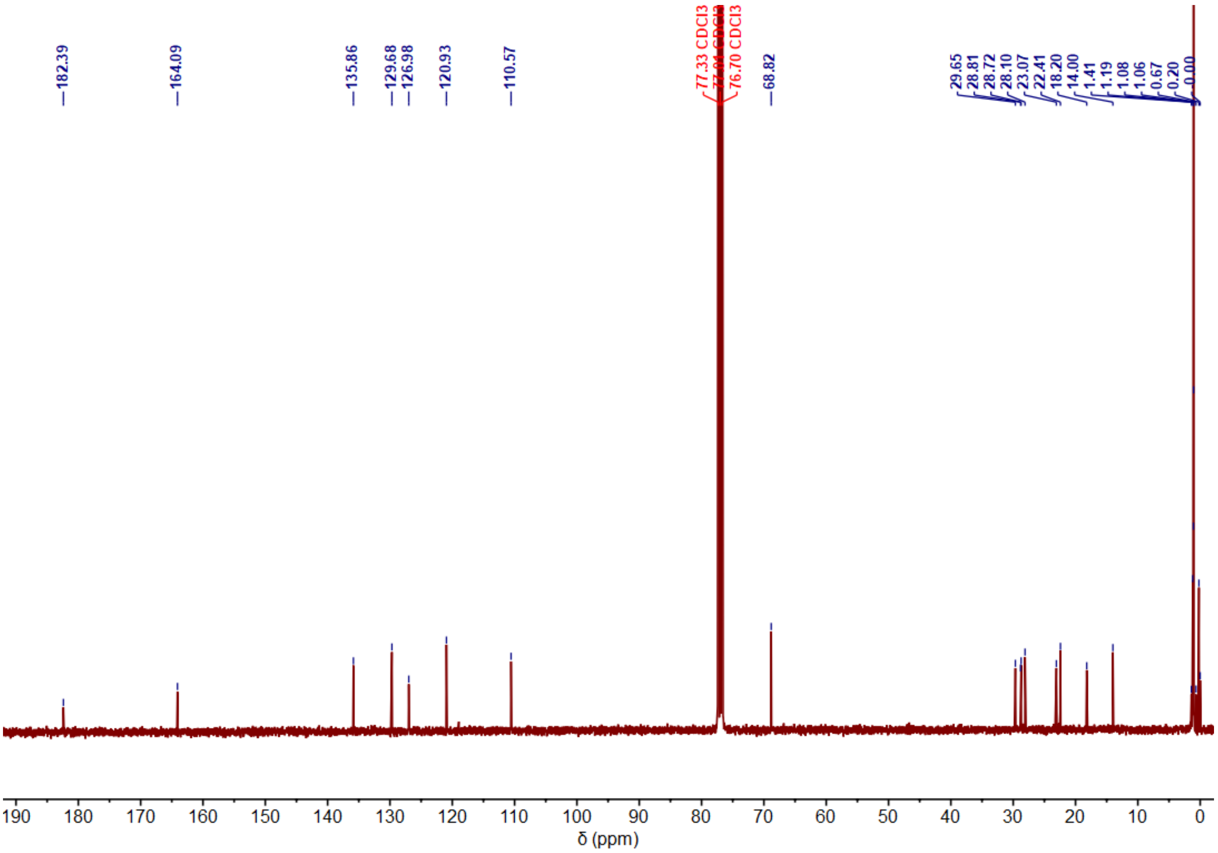

Figure S45: ^{13}C NMR (100 MHz, $CDCl_3$) of ***Pent-AQ-Si32-AQ-Pent***

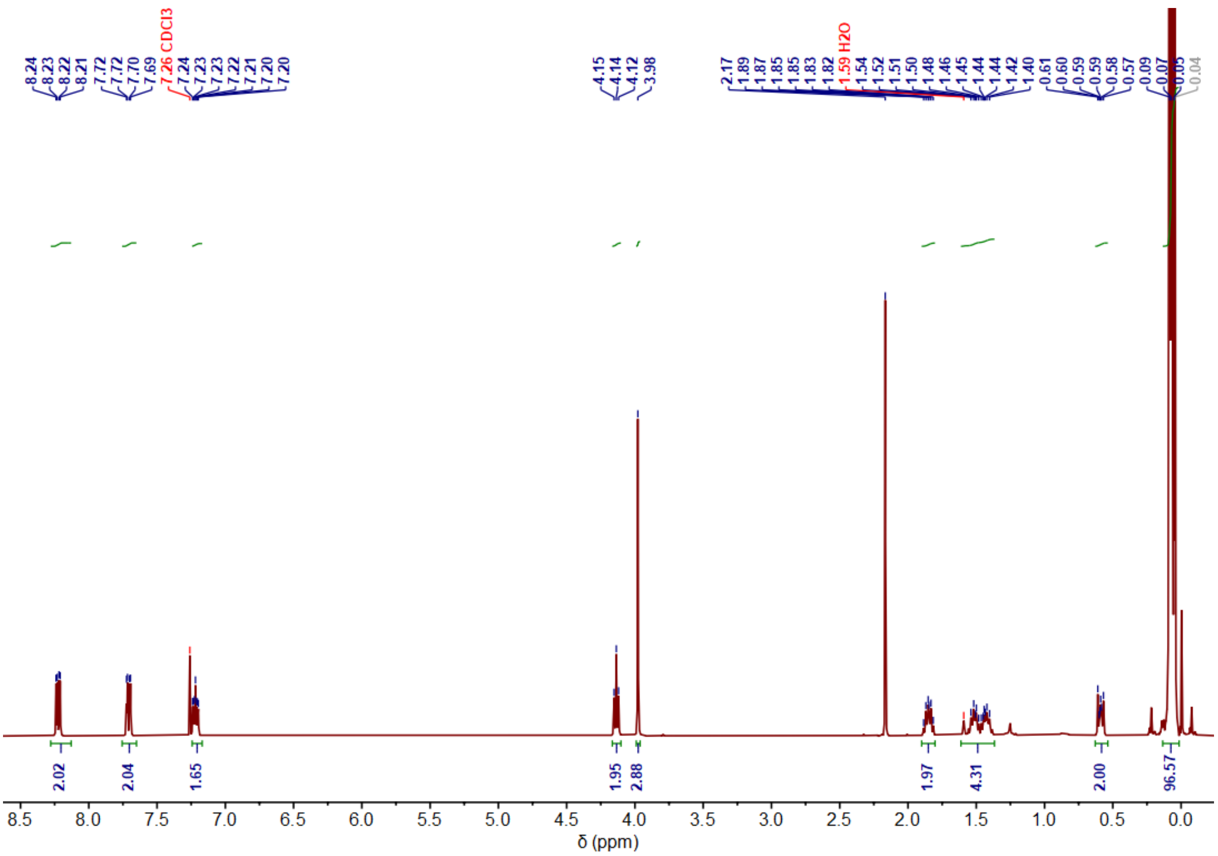

Figure S46**: ^{1}H NMR (400 MHz, $CDCl_3$) of **Me-AQ-Si$_{15}$

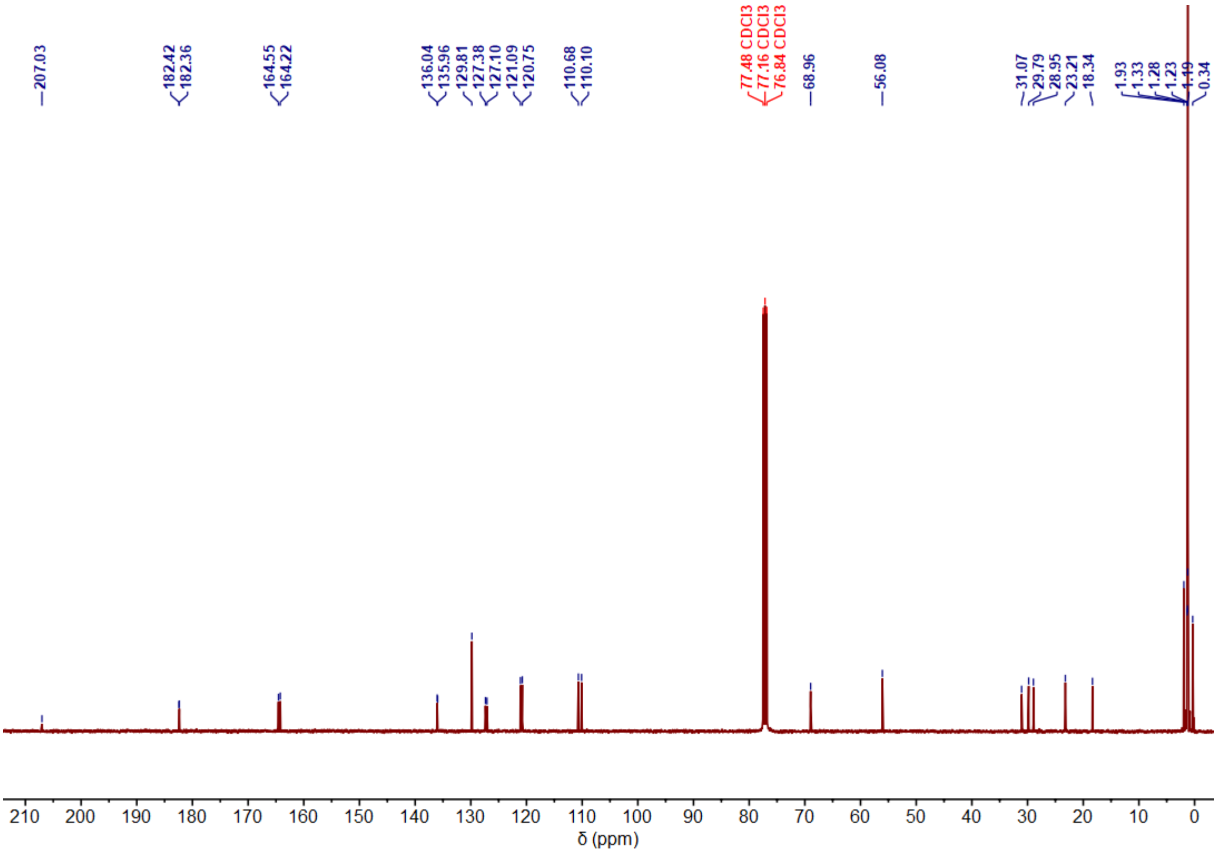

Figure S47**: ^{13}C NMR (100 MHz, $CDCl_3$) of **Me-AQ-Si$_{15}$

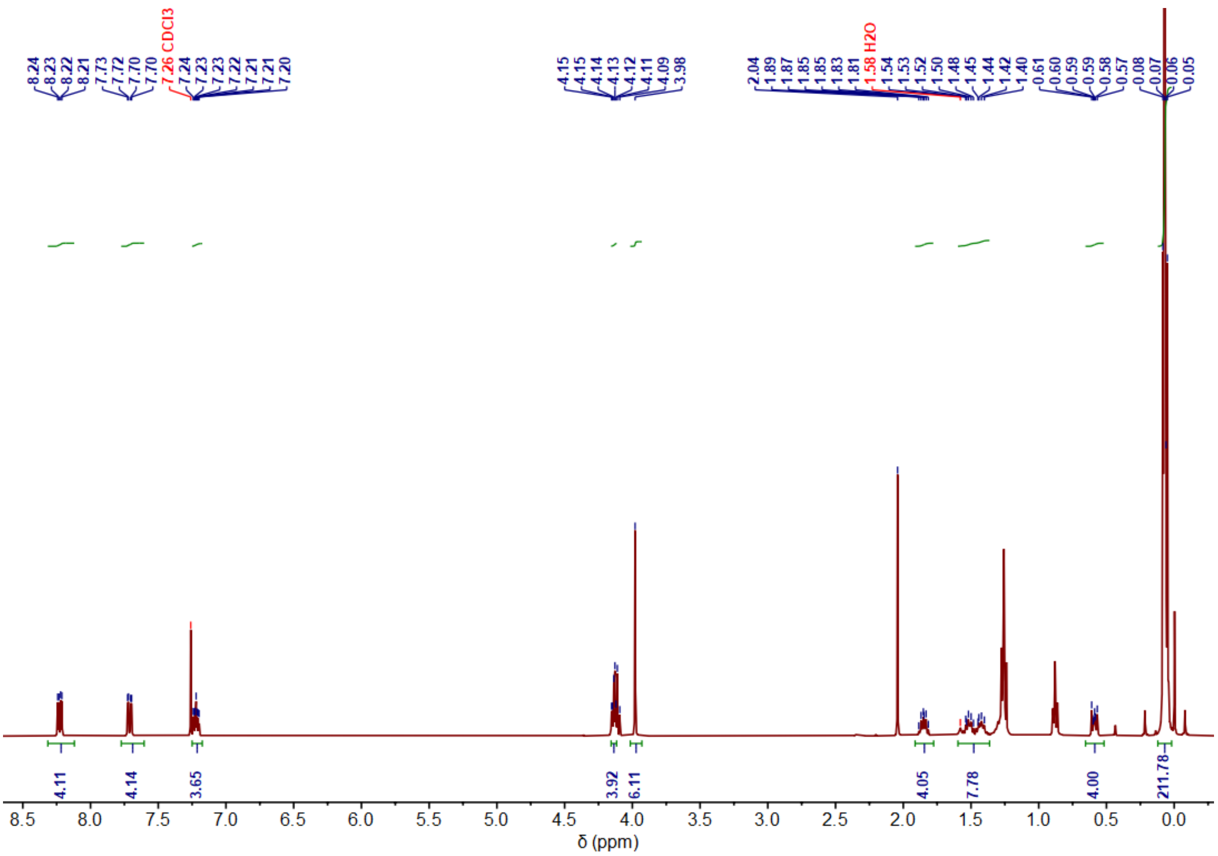

Figure S48: *^{1}H NMR (400 MHz, $CDCl_3$) of* ***Me-AQ-Si$_{32}$-AQ-Me***

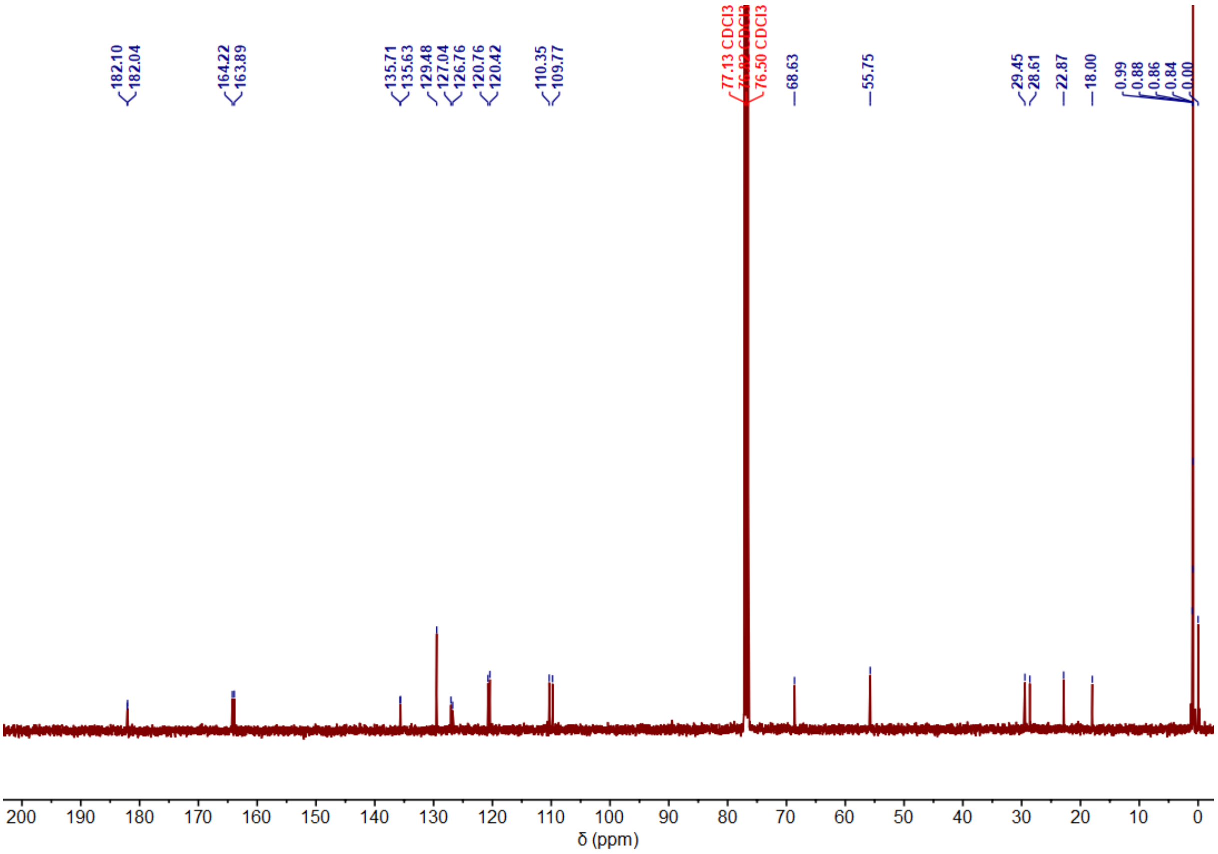

Figure S49: *^{13}C NMR (100 MHz, $CDCl_3$) of* ***Me-AQ-Si$_{32}$-AQ-Me***

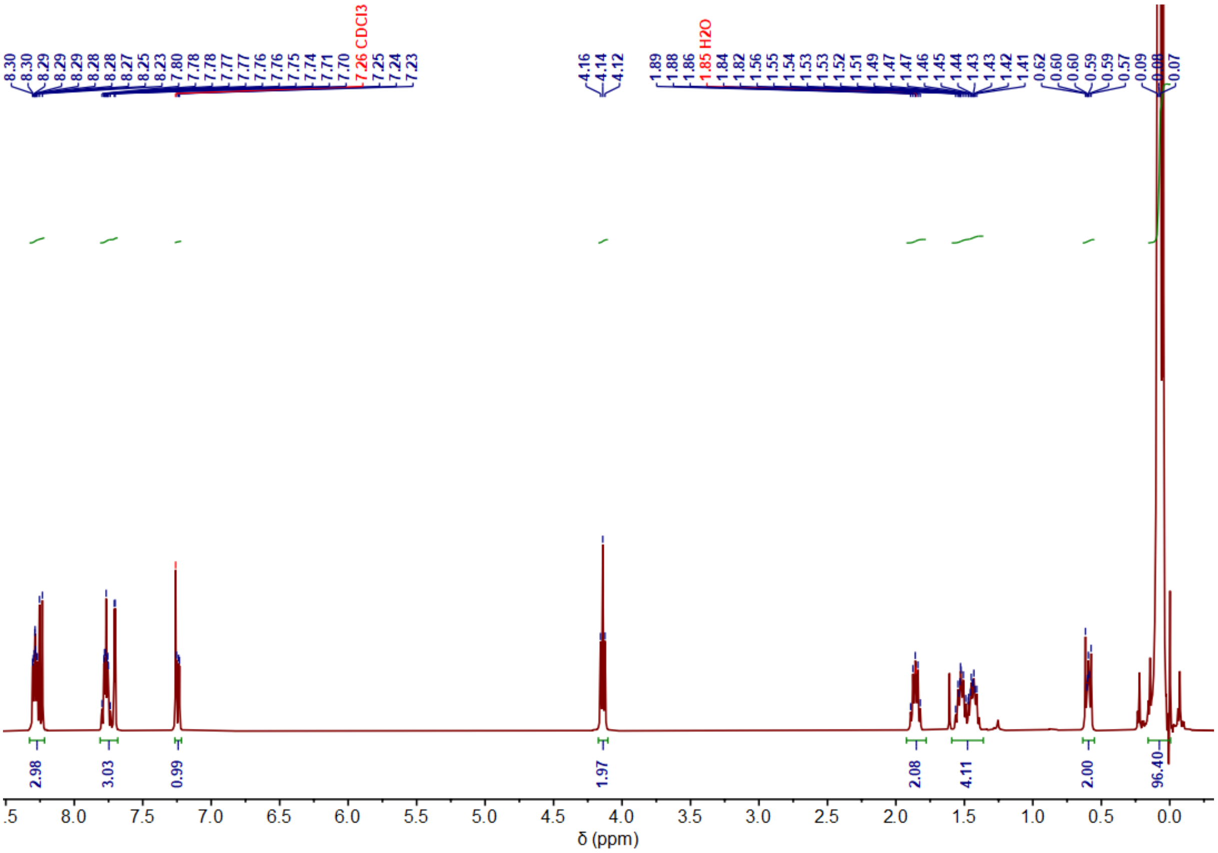

Figure S50: *¹H NMR (400 MHz, $CDCl_3$) of* ***H-AQ-Si₁₅***

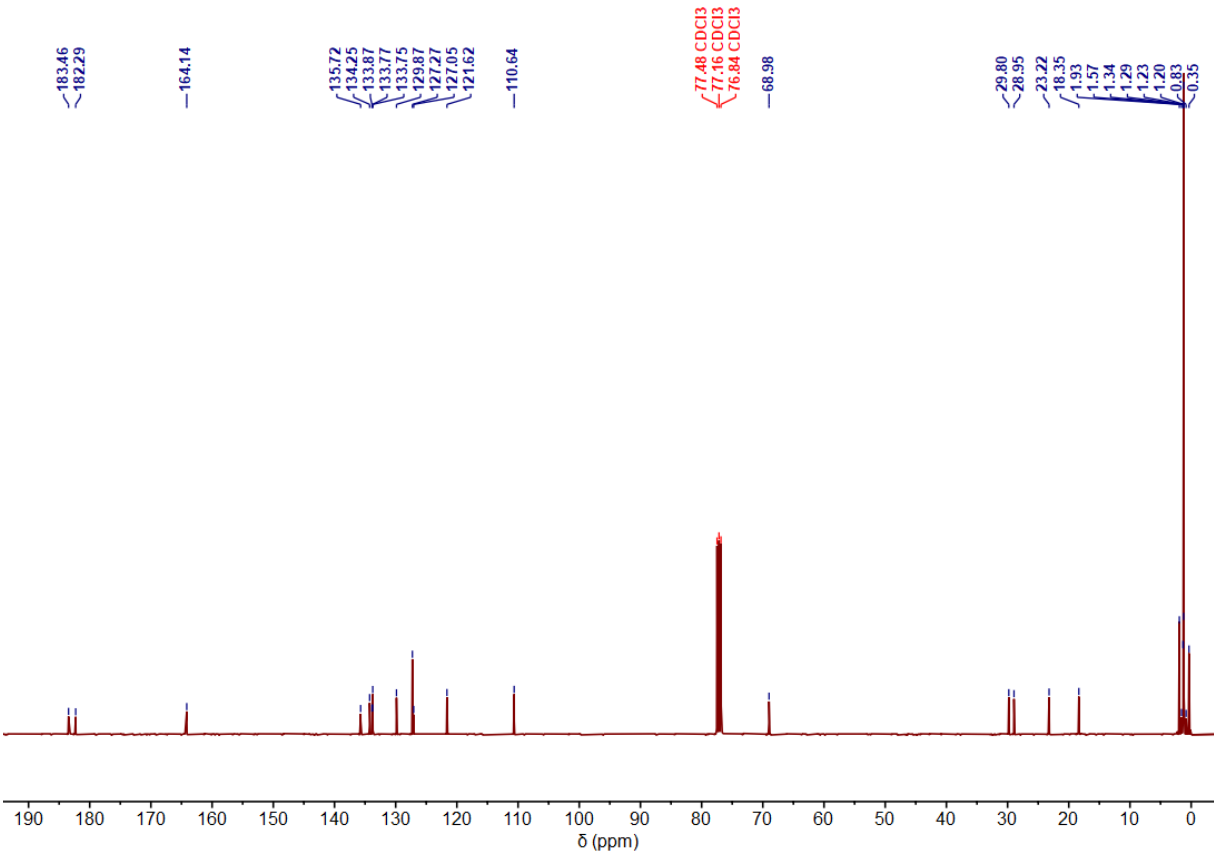

Figure S51: *¹³C NMR (100 MHz, $CDCl_3$) of* ***H-AQ-Si₁₅***

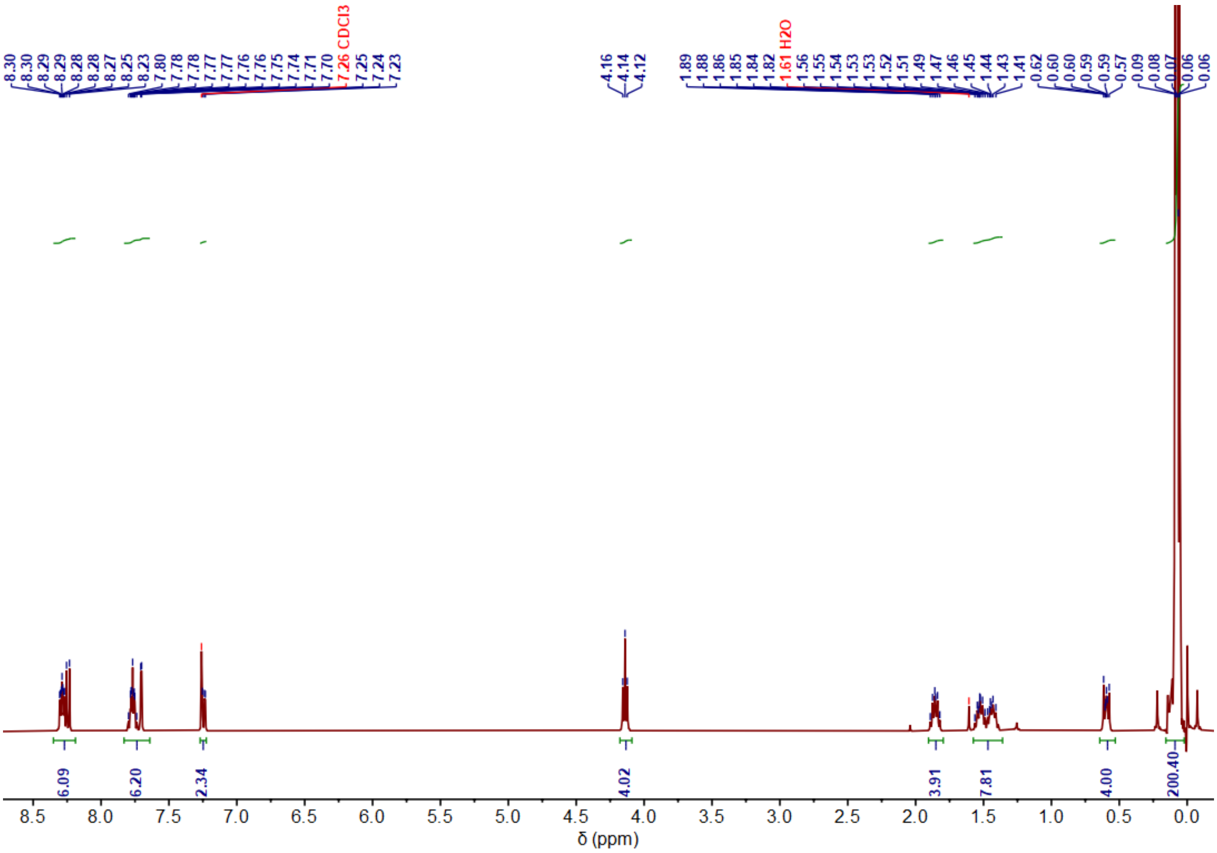

Figure S52: ^{1}H NMR (400 MHz, $CDCl_3$) of ***H-AQ-Si$_{32}$-AQ-H***

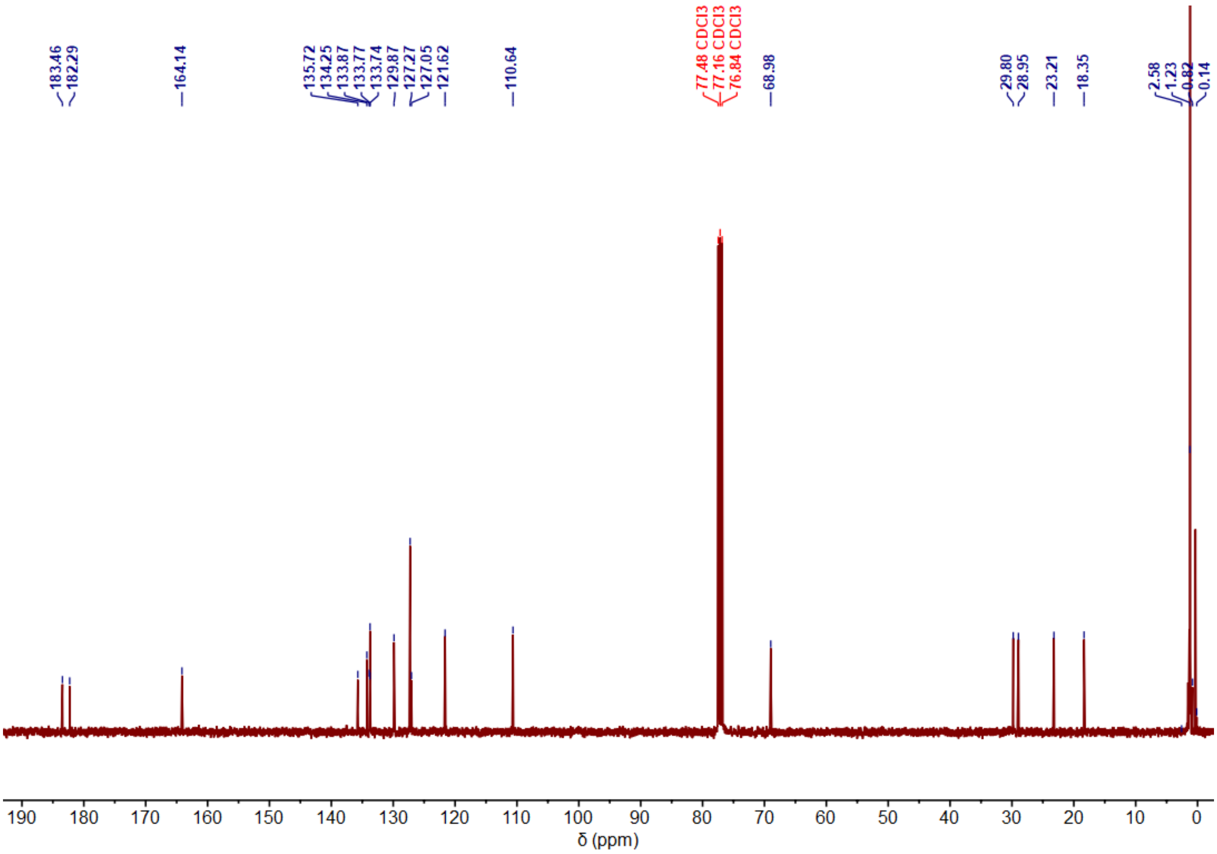

Figure S53: ^{13}C NMR (100 MHz, $CDCl_3$) of ***H-AQ-Si$_{32}$-AQ-H***

4.3 Azo derivatives

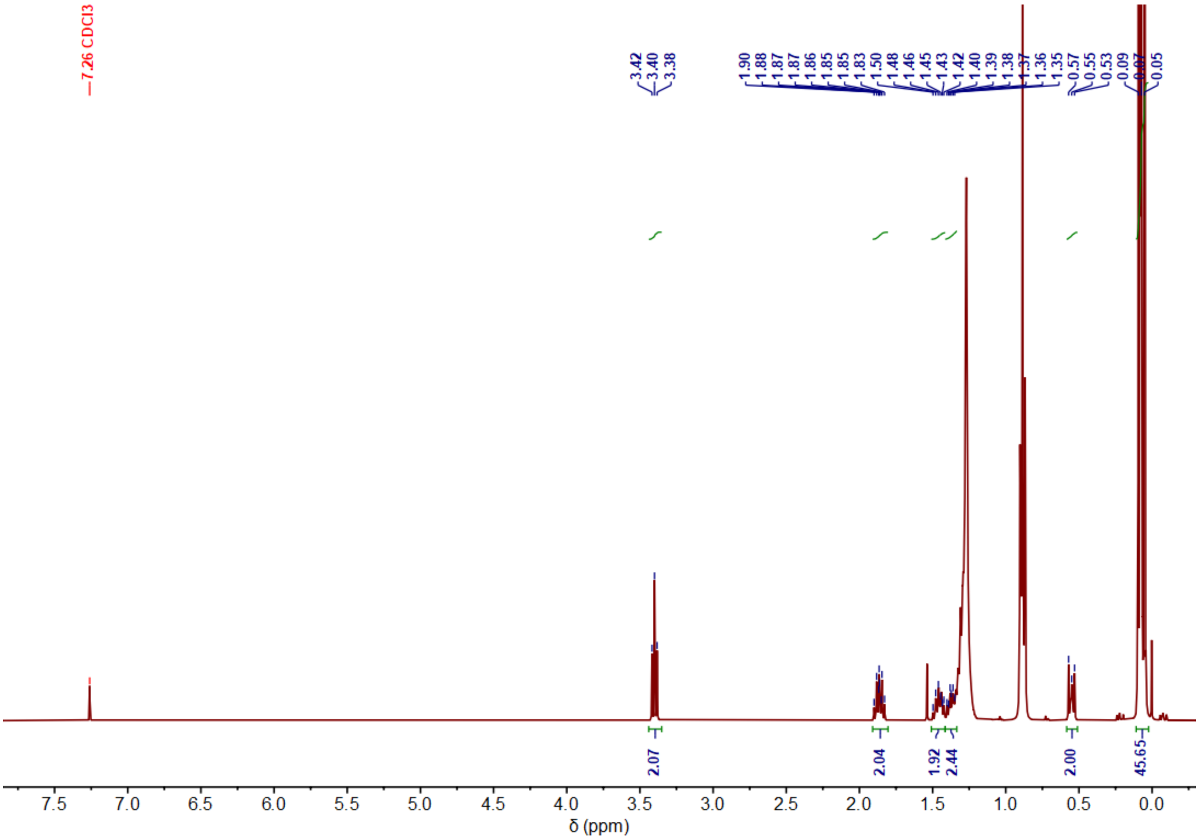

Figure S54: *^{1}H NMR (400 MHz, $CDCl_3$) of* ***Br-C$_5$-Si$_7$***

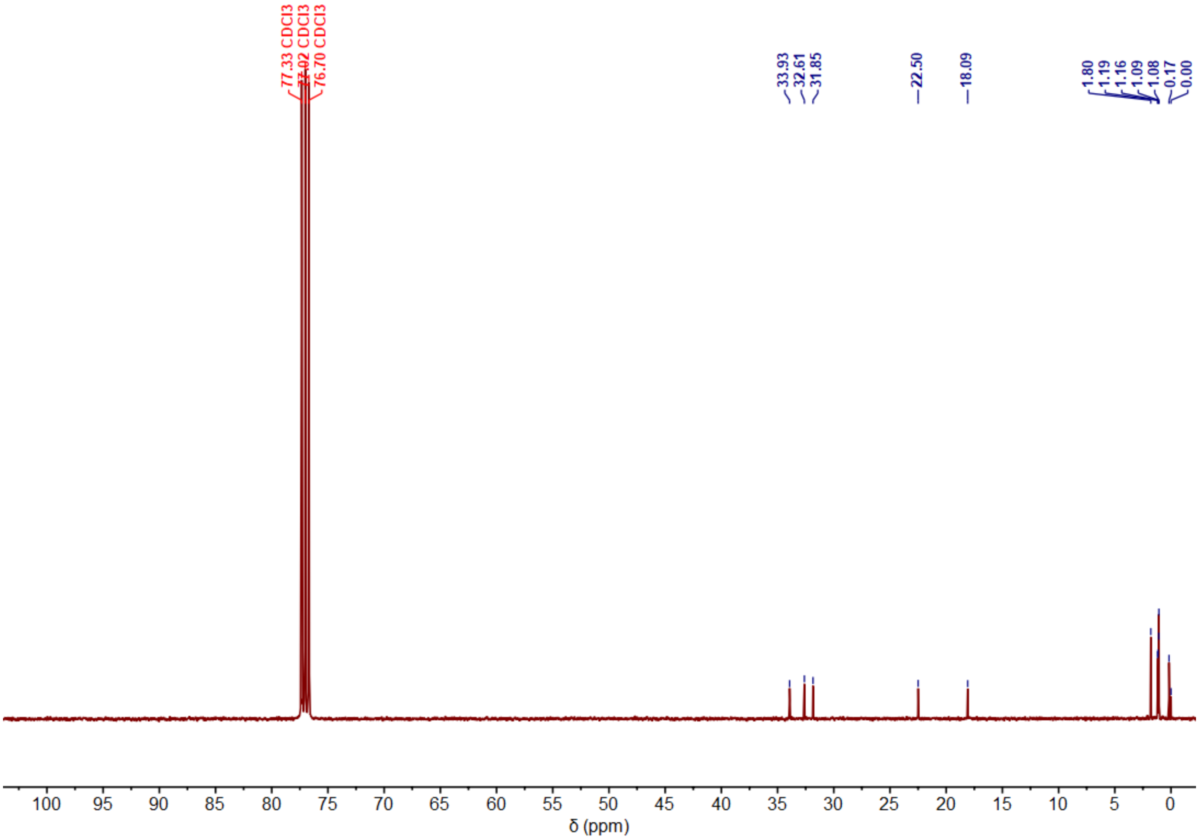

Figure S55: *^{13}C NMR (100 MHz, $CDCl_3$) of* ***Br-C$_5$-Si$_7$***

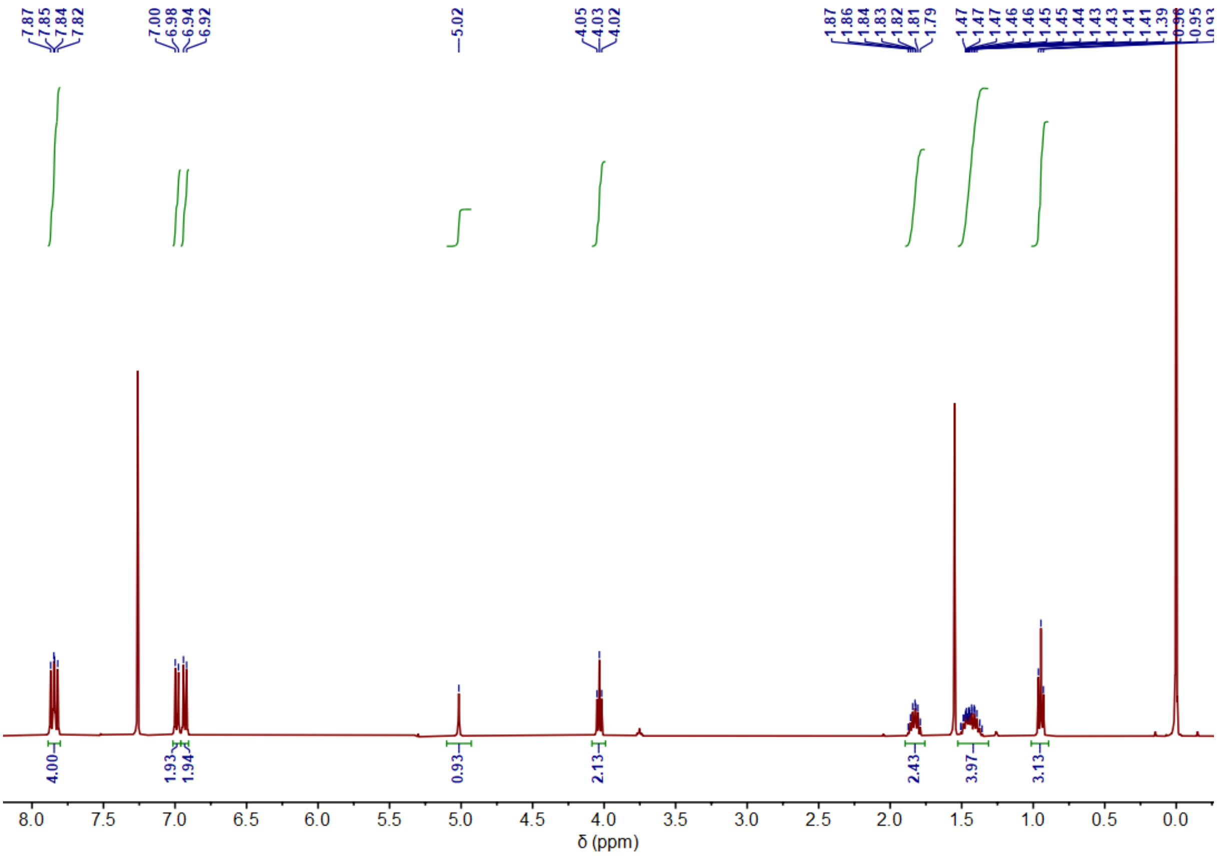

Figure S56**: ^{1}H NMR (400 MHz, $CDCl_3$) of **4-((4-(pentyloxy)phenyl)diazenyl)phenol (10)

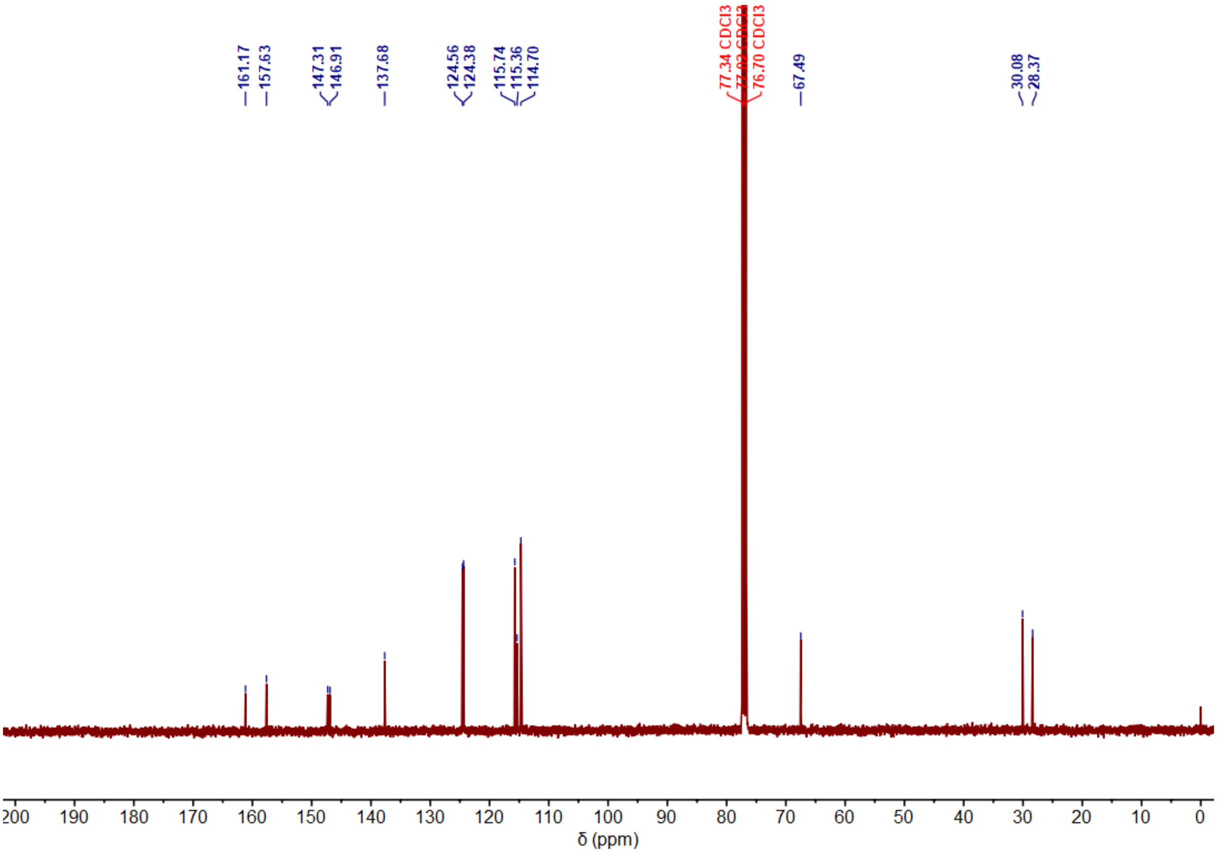

Figure S57**: ^{13}C NMR (100 MHz, $CDCl_3$) of **4-((4-(pentyloxy)phenyl)diazenyl)phenol (10)

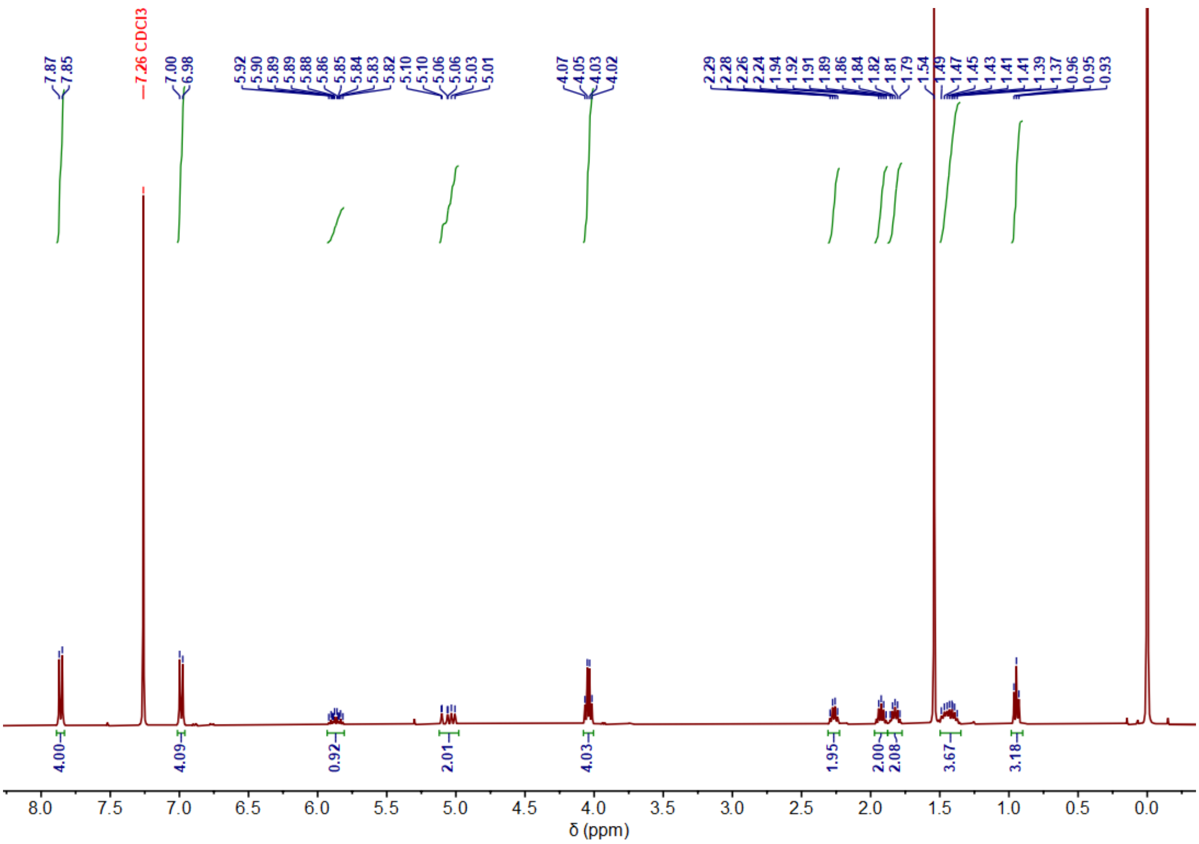

Figure S58: *^{1}H NMR (400 MHz, $CDCl_3$) of* ***1-(4-(pent-4-en-1-yloxy)phenyl)-2-(4-(pentyloxy)phenyl)diazene (11)***

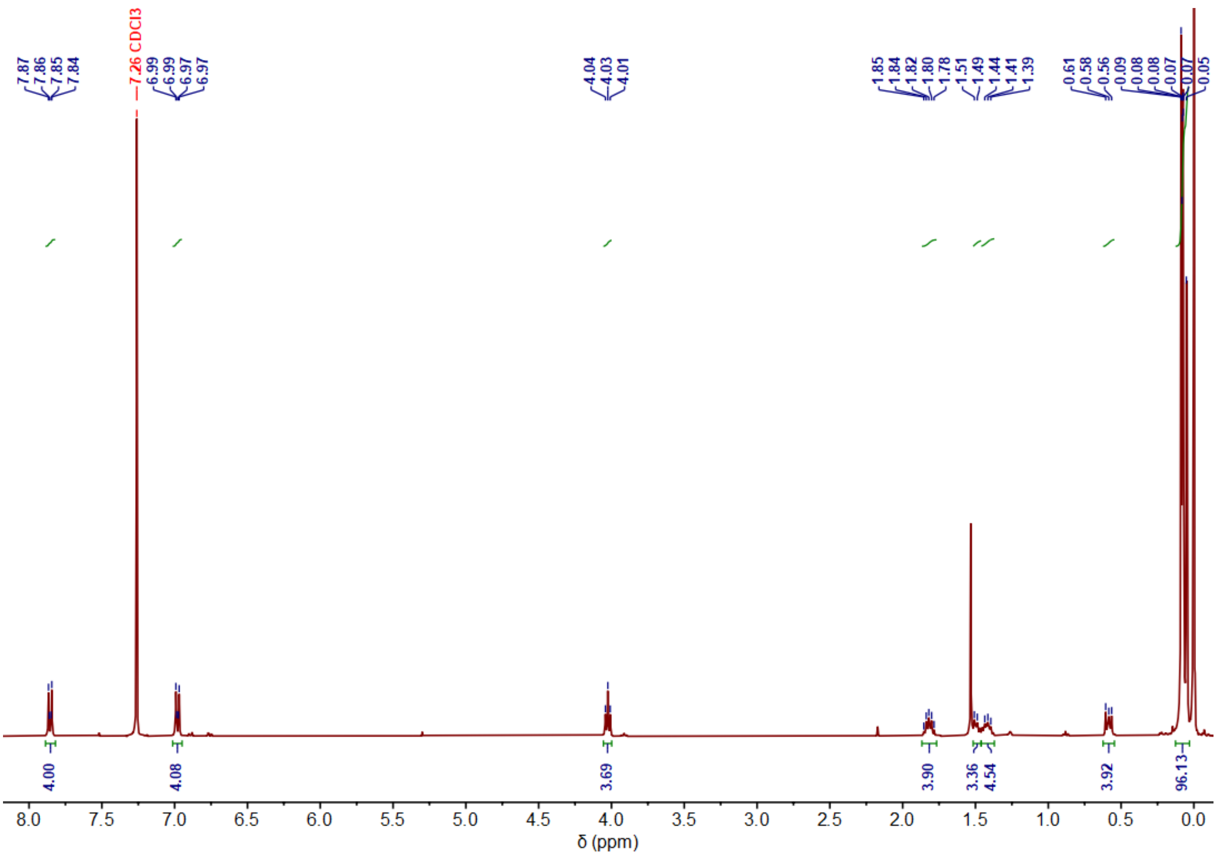

Figure S59: *^{1}H NMR (400 MHz, CDCl$_3$) of **Si$_7$-Azo-Si$_7$***

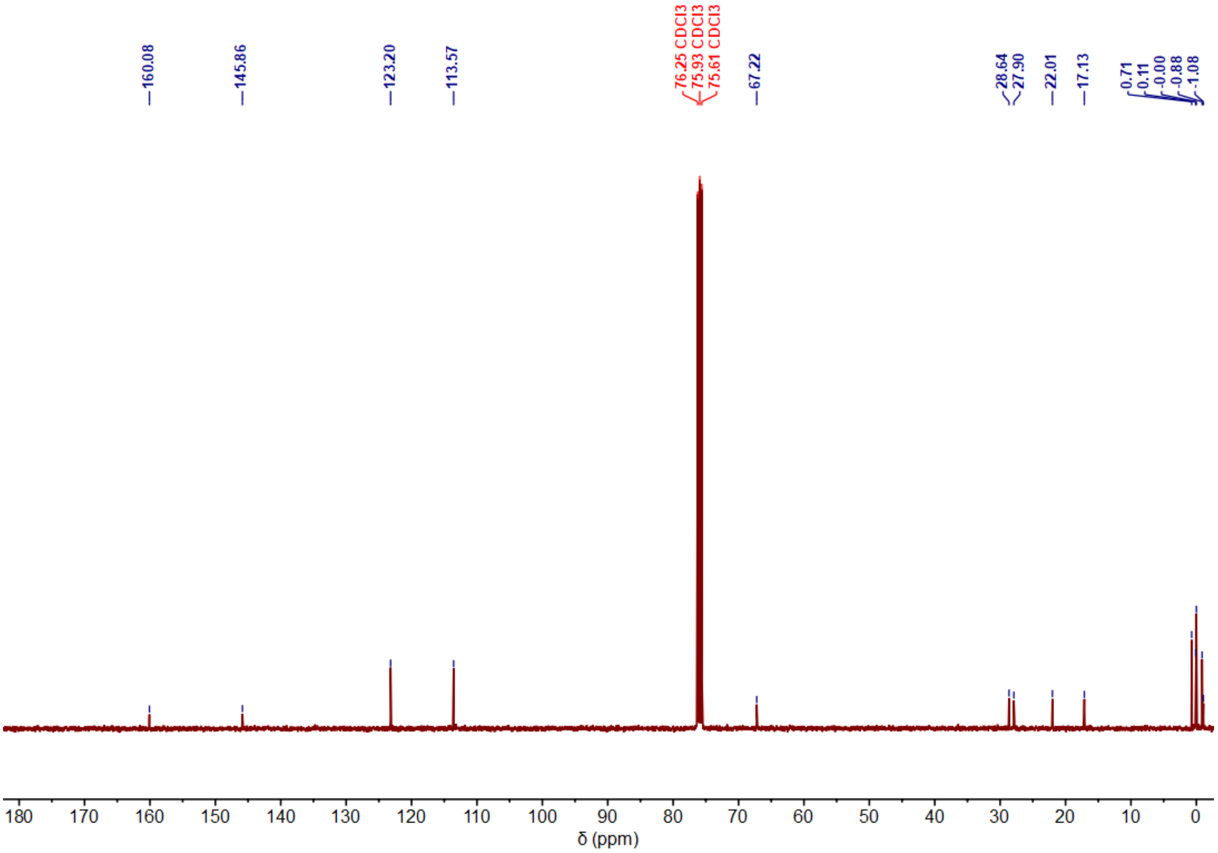

Figure S60: *^{13}C NMR (100 MHz, CDCl$_3$) of **Si$_7$-Azo-Si$_7$***

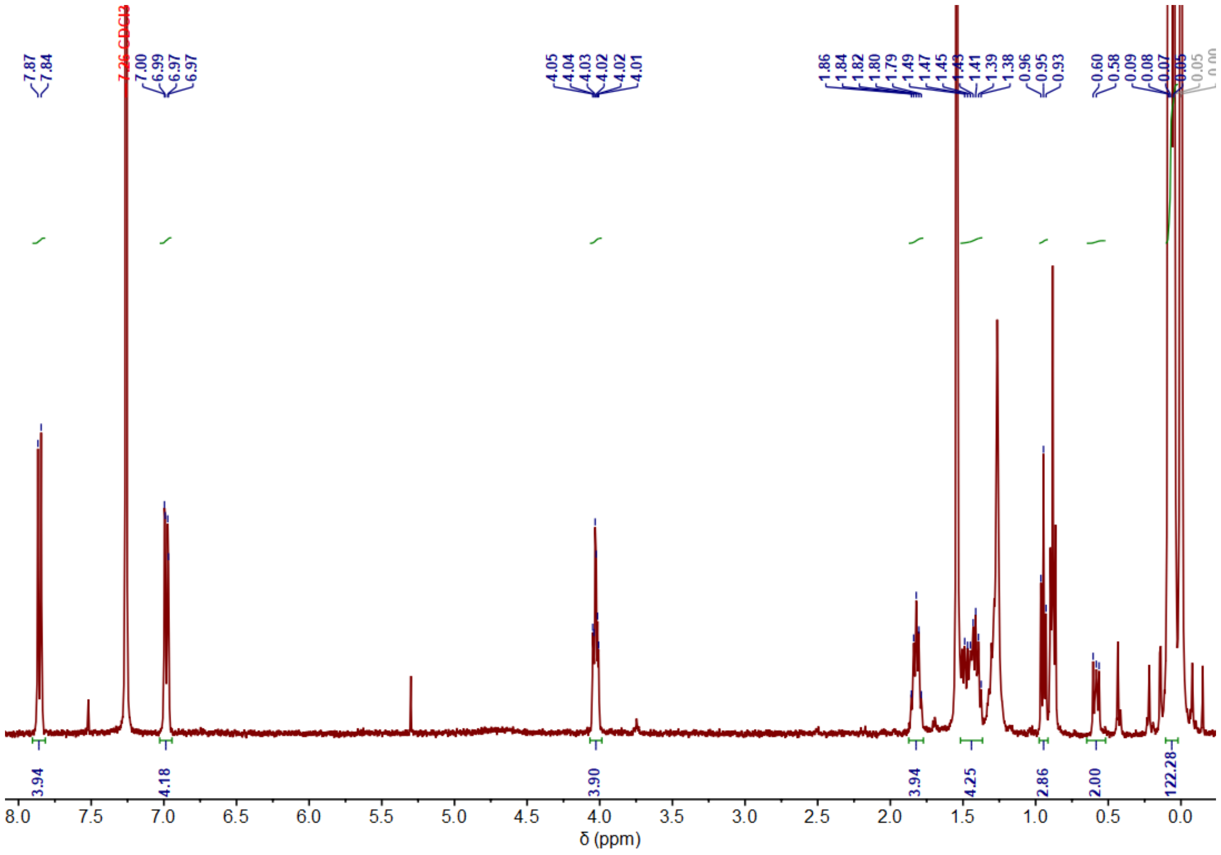

Figure S61: ^{1}H NMR (400 MHz, $CDCl_3$) of **Azo-Si$_{15}$**

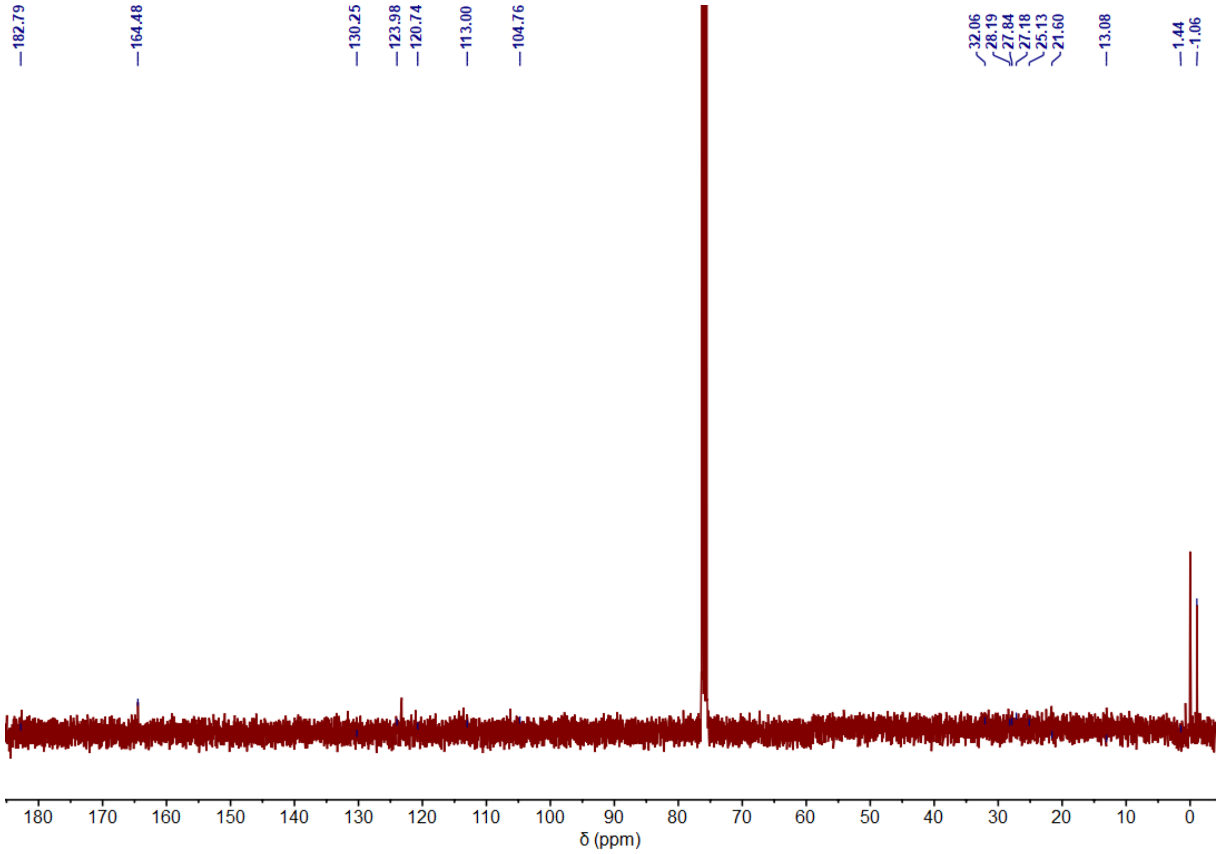

Figure S62: ^{13}C NMR (100 MHz, $CDCl_3$) of **Azo-Si$_{15}$**

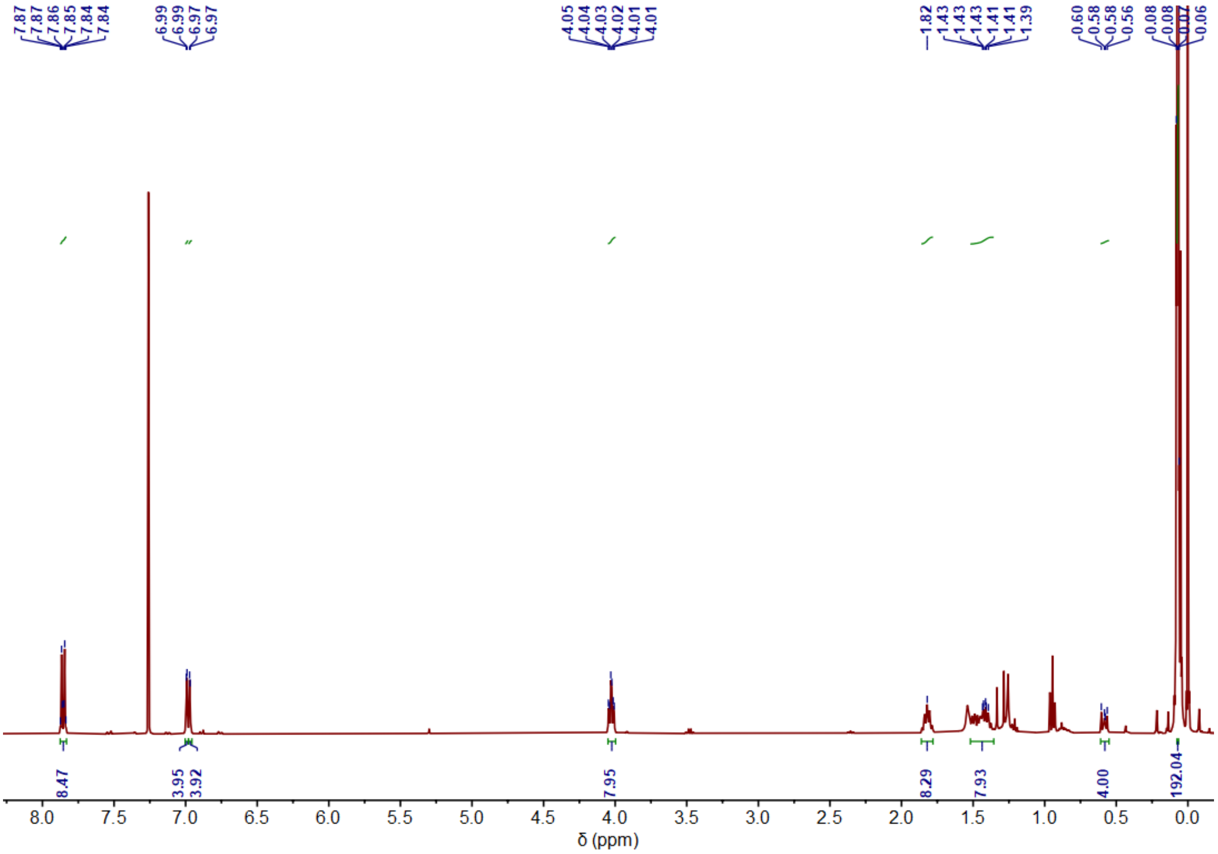

Figure S63**: ^{1}H NMR (400 MHz, $CDCl_3$) of **Azo-Si$_{32}$-Azo

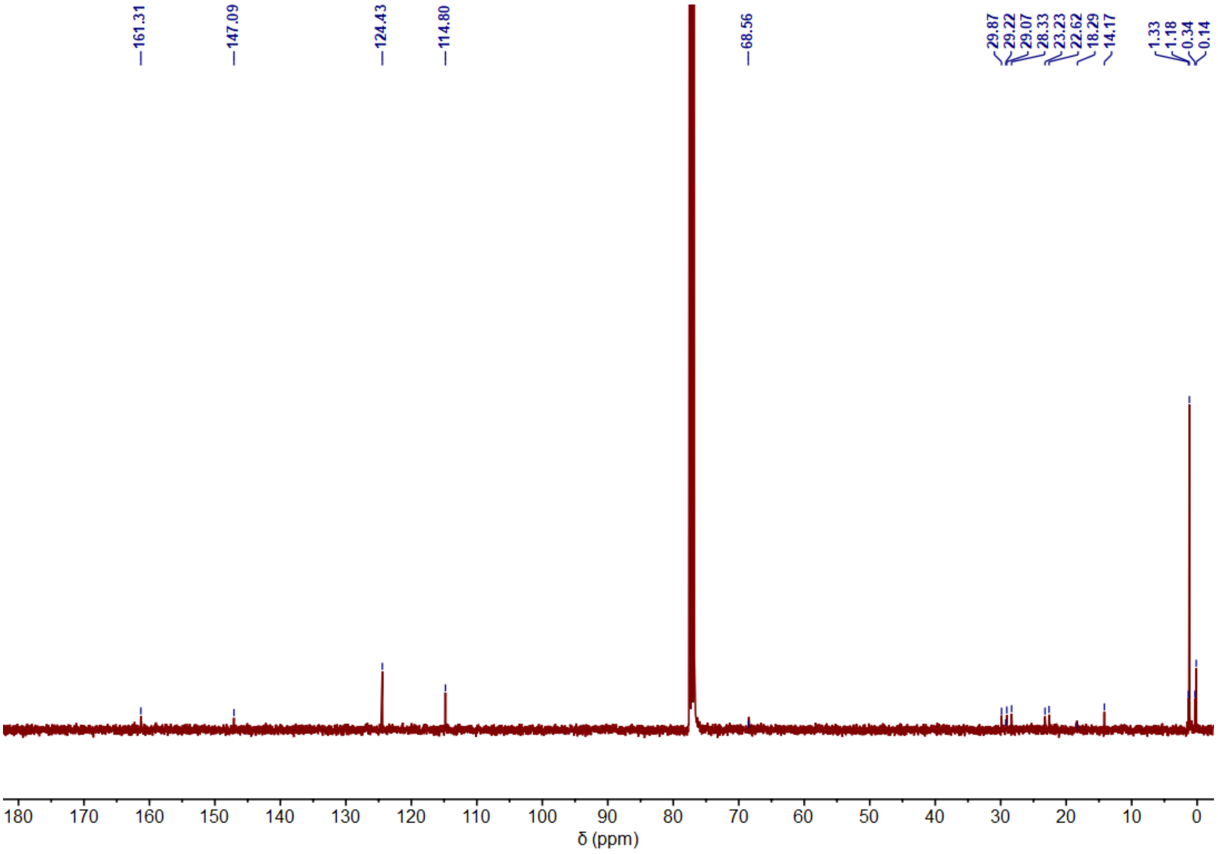

Figure S64**: ^{13}C NMR (100 MHz, $CDCl_3$) of **Azo-Si$_{32}$-Azo

4.4 OPV derivatives

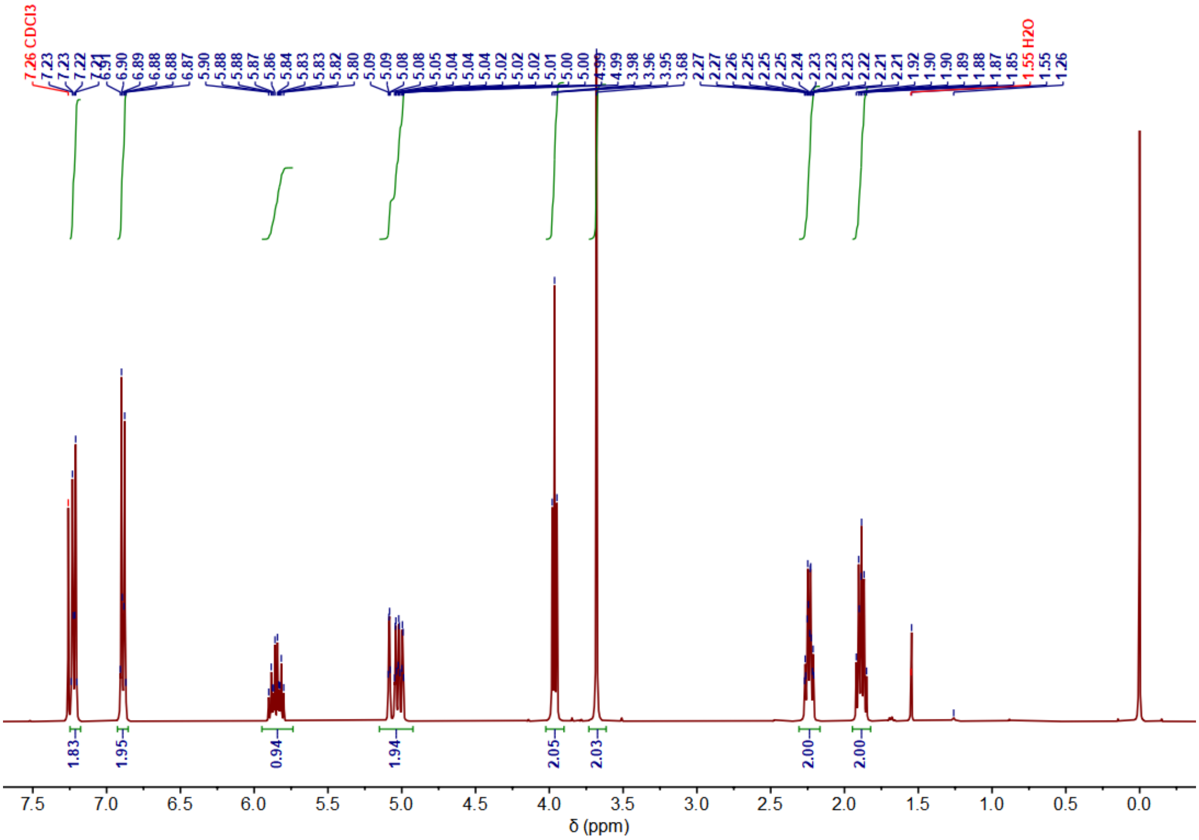

Figure S65: *¹H NMR (400 MHz, CDCl₃) of* ***2-(4-(pent-4-en-1-yloxy)phenyl)acetonitrile (12)***

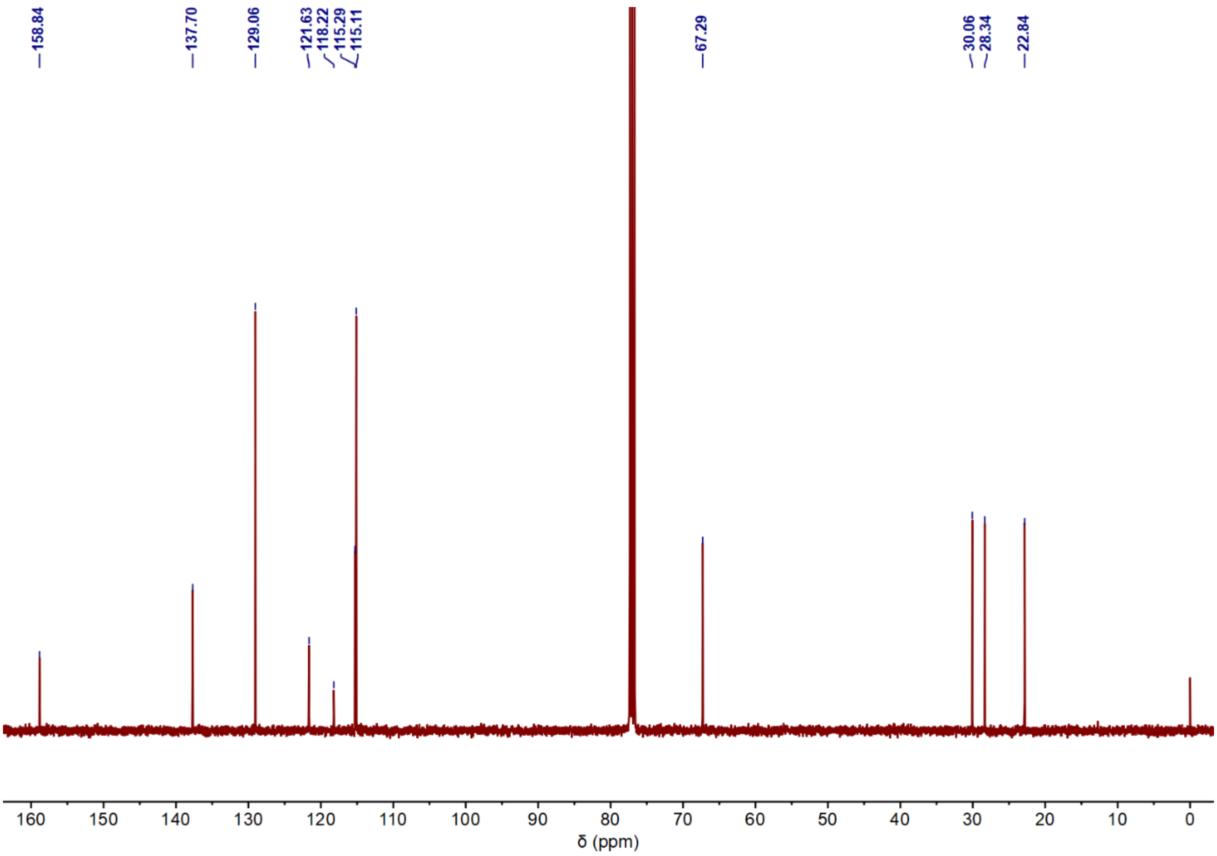

Figure S66: *¹³C NMR (100 MHz, CDCl₃) of* ***2-(4-(pent-4-en-1-yloxy)phenyl)acetonitrile (12)***

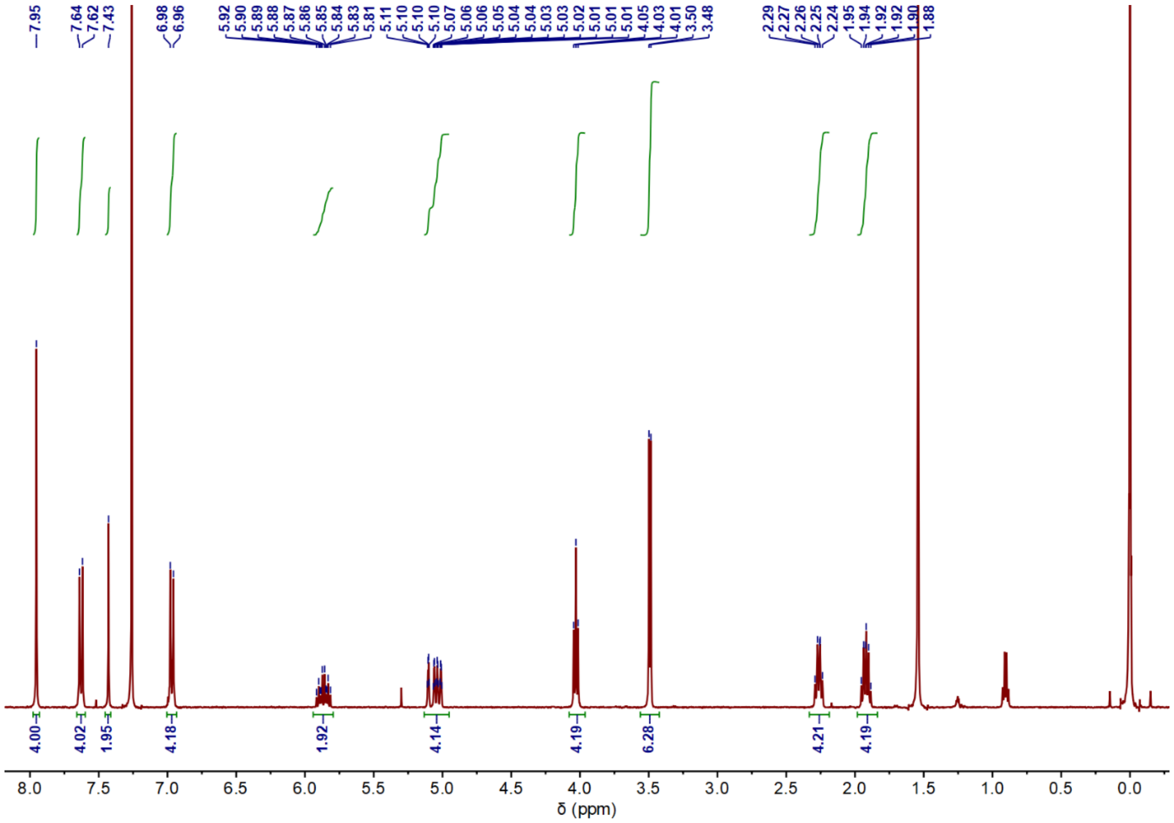

Figure S67: *^{1}H NMR (400 MHz, CDCl3) of* ***3,3'-(1,4-phenylene)bis(2-(4-(pent-4-en-1-yloxy)phenyl)acrylonitrile) (13)***

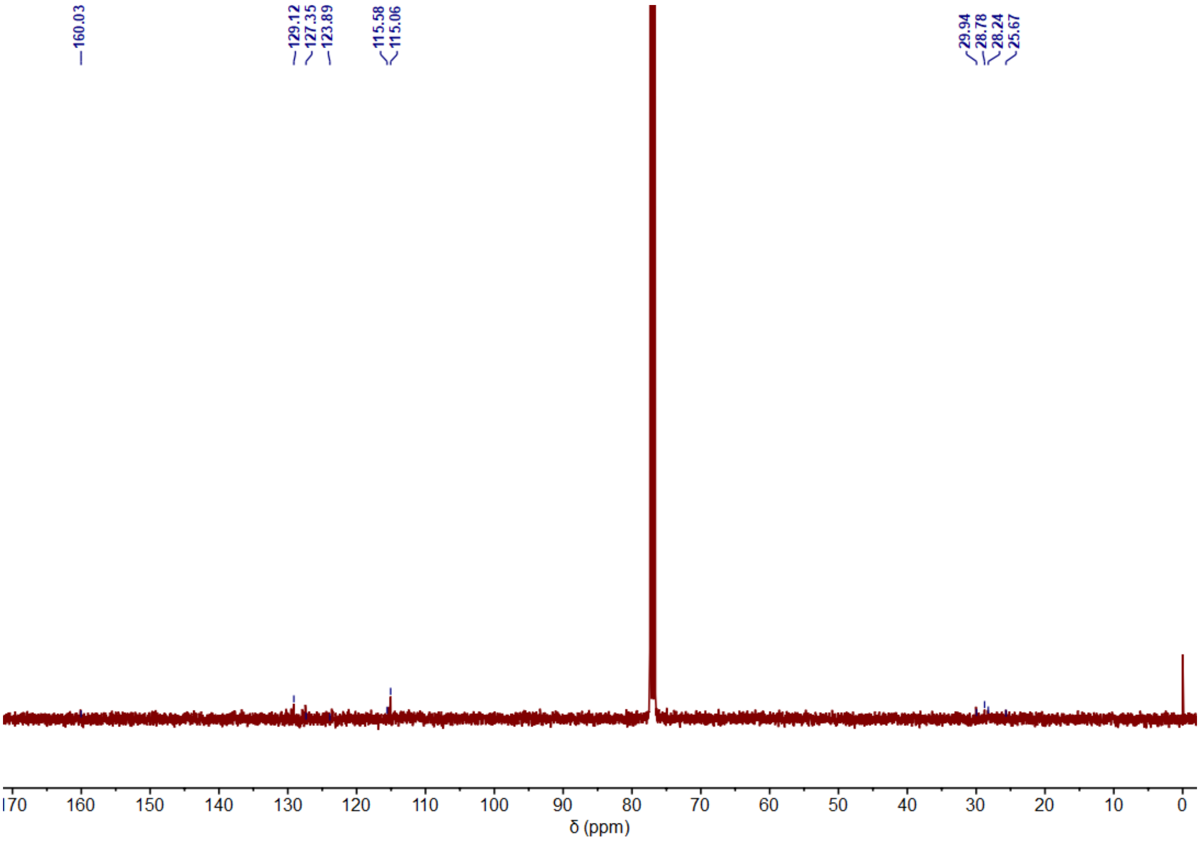

Figure S68*: ^{13}C NMR (100 MHz, CDCl3) of* ***3,3'-(1,4-phenylene)bis(2-(4-(pent-4-en-1-yloxy)phenyl)acrylonitrile) (13)***

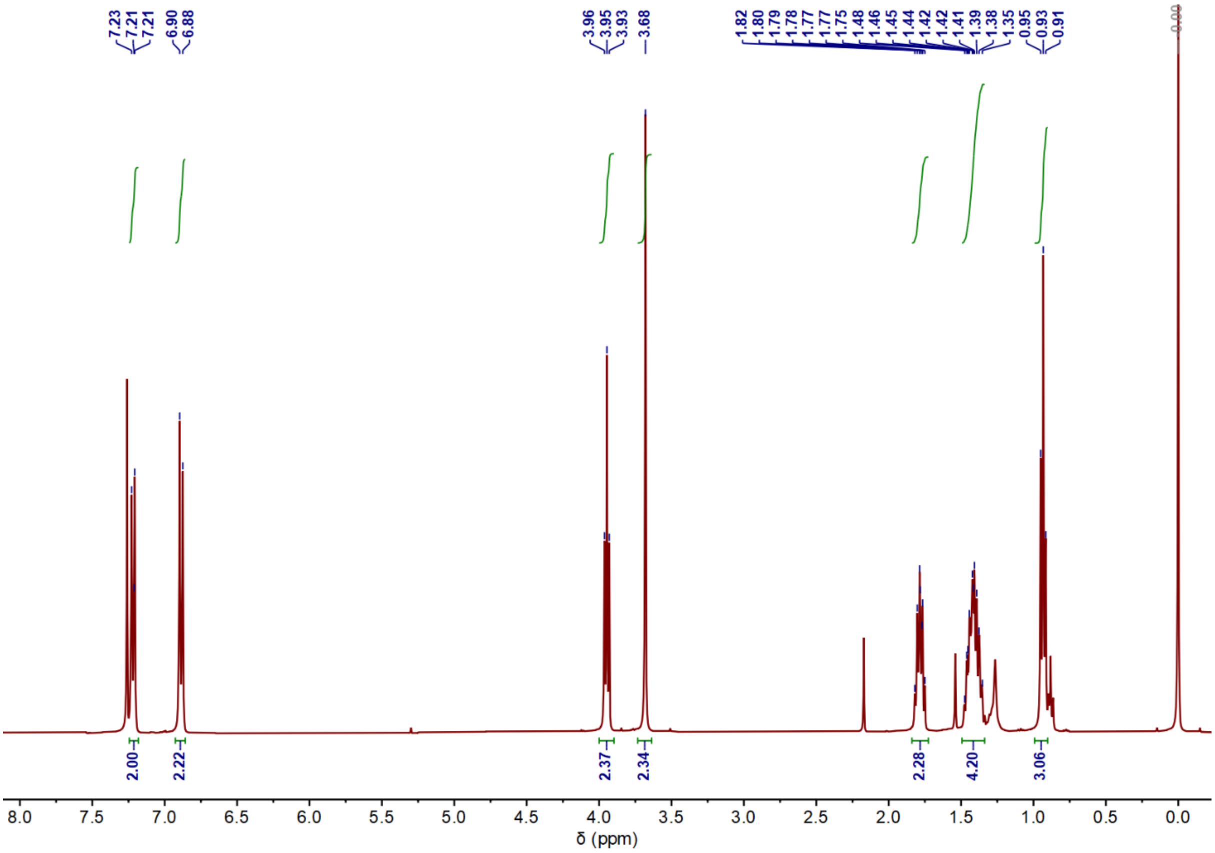

Figure S69: *^{1}H NMR (400 MHz, $CDCl_3$) of* ***2-(4-(pentyloxy)phenyl)acetonitrile (14)***

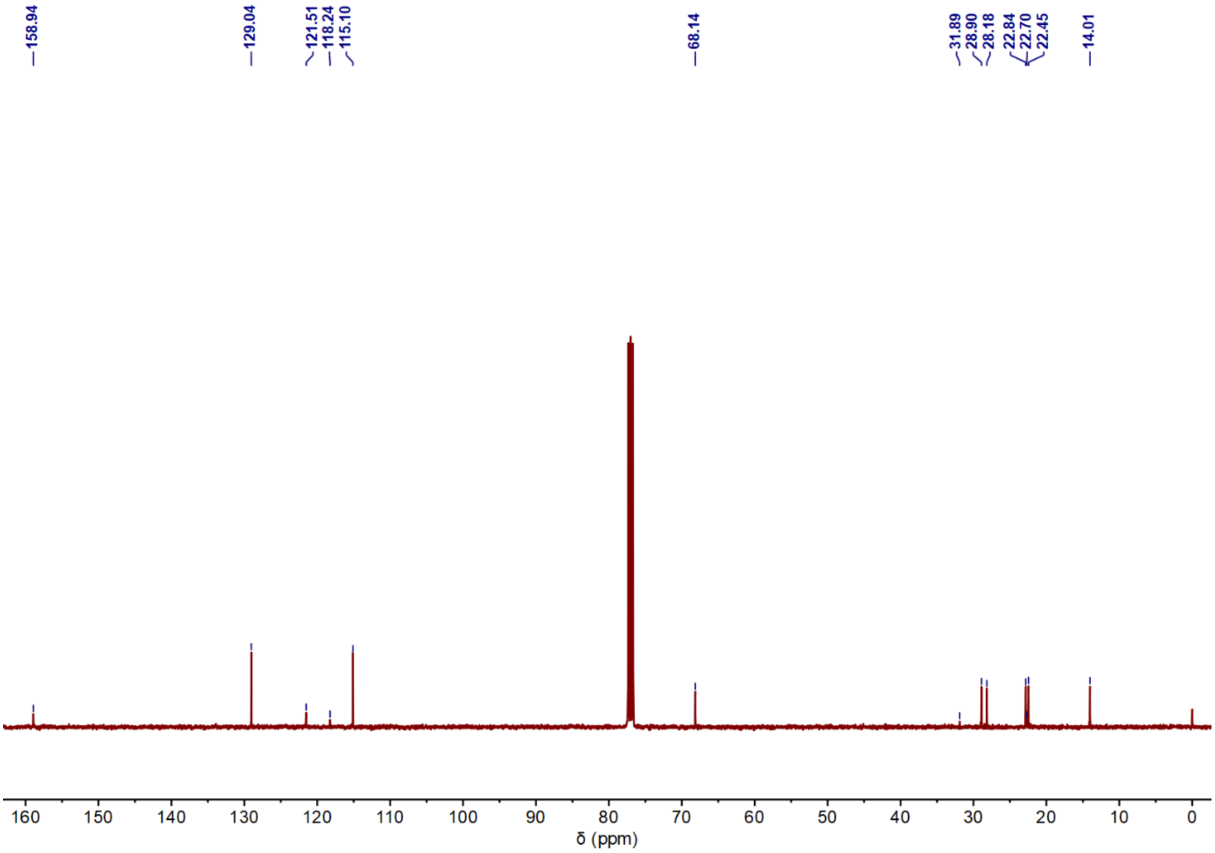

Figure S70: *^{13}C NMR (100 MHz, $CDCl_3$) of* ***2-(4-(pentyloxy)phenyl)acetonitrile (14)***

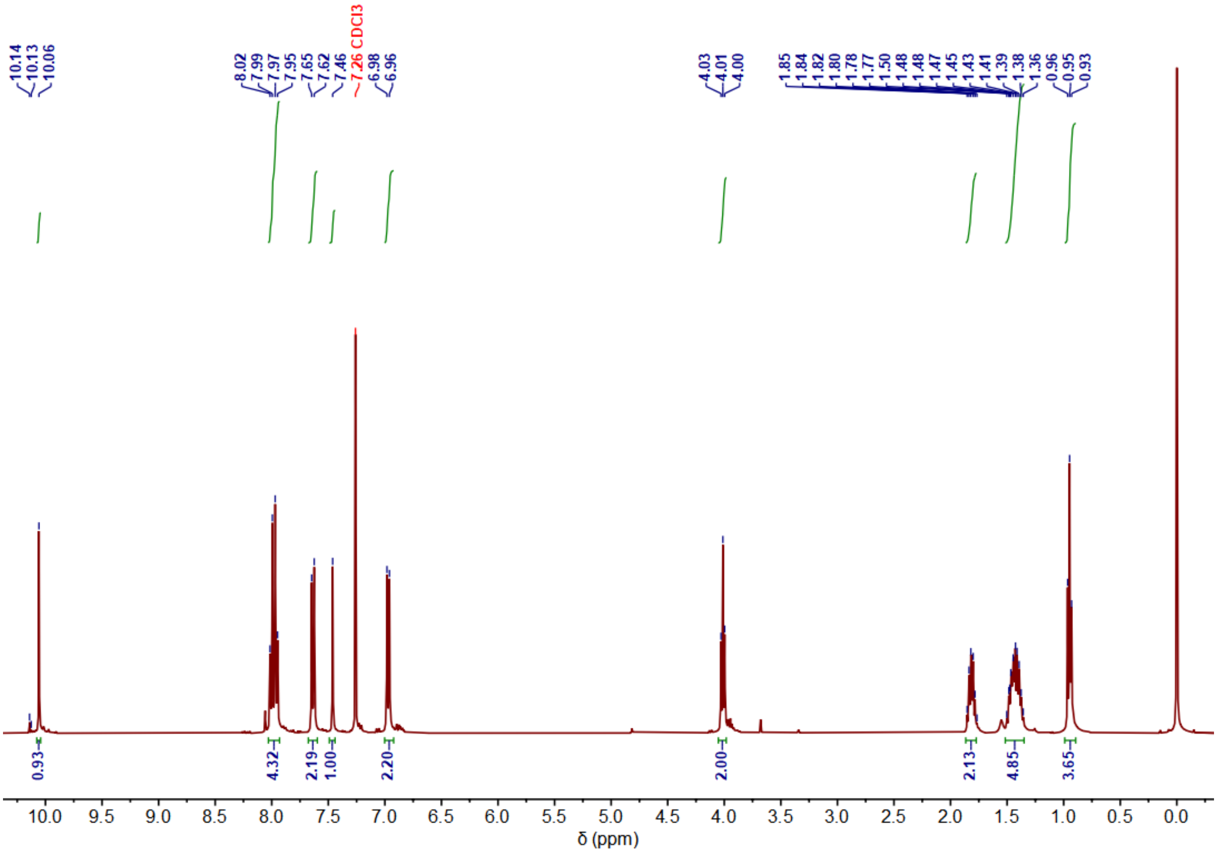

Figure S71**: ^{1}H NMR (400 MHz, $CDCl_3$) of **3-(4-formylphenyl)-2-(4-(pentyloxy)phenyl)acetonitrile (15)

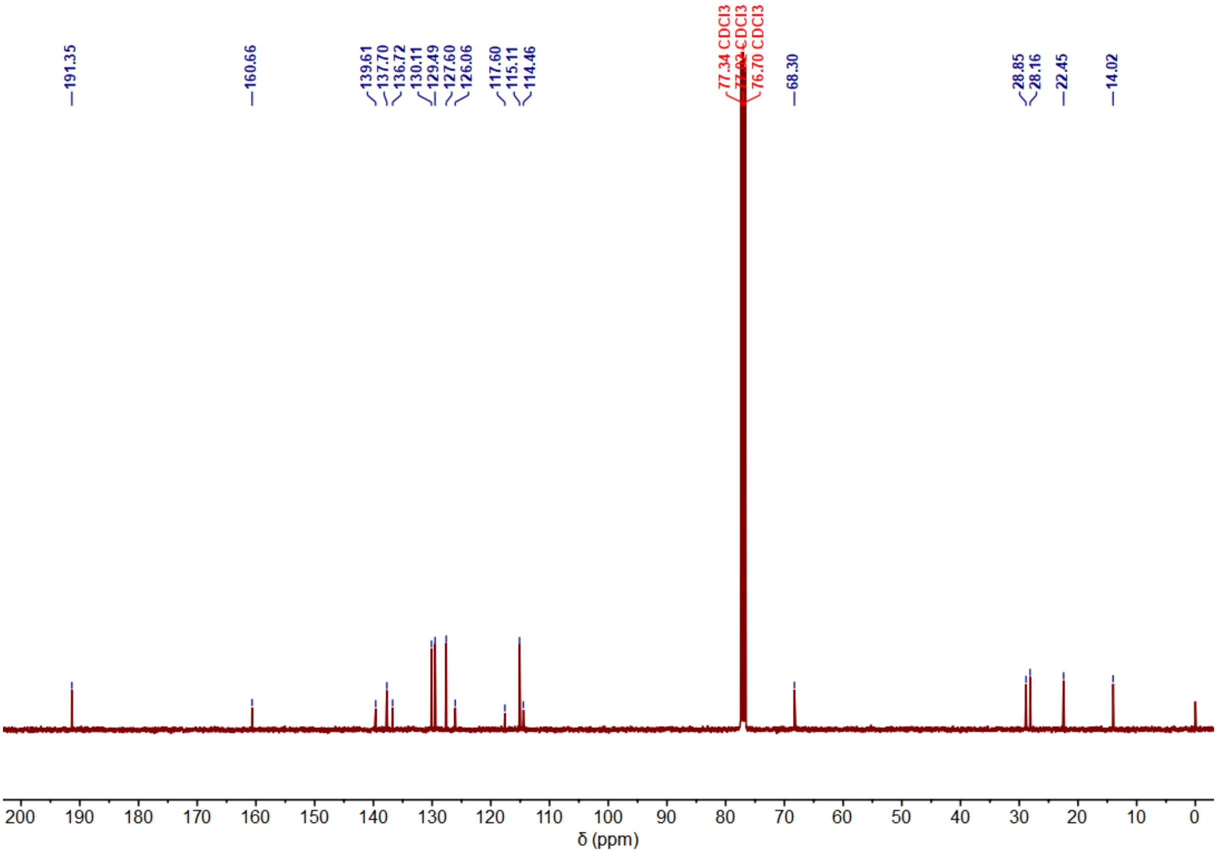

Figure S72**: ^{13}C NMR (100 MHz, $CDCl_3$) of **3-(4-formylphenyl)-2-(4-(pentyloxy)phenyl)acetonitrile (15)

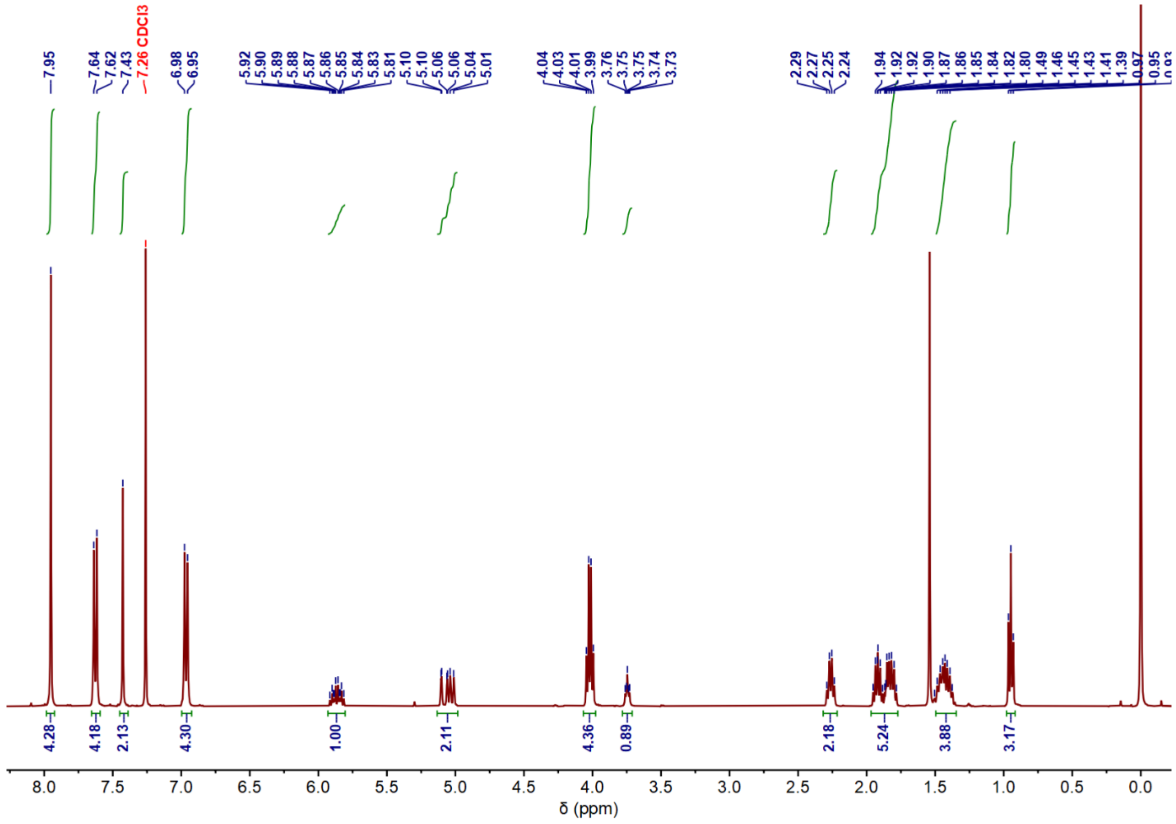

Figure S73: ^{1}H NMR (400 MHz, $CDCl_3$) of **3-(4-(2-cyano-2-(4-(pent-4-en-1-yloxy)phenyl)vinyl)phenyl)-2-(4-(pentyloxy)phenyl)acrylonitrile (16)**

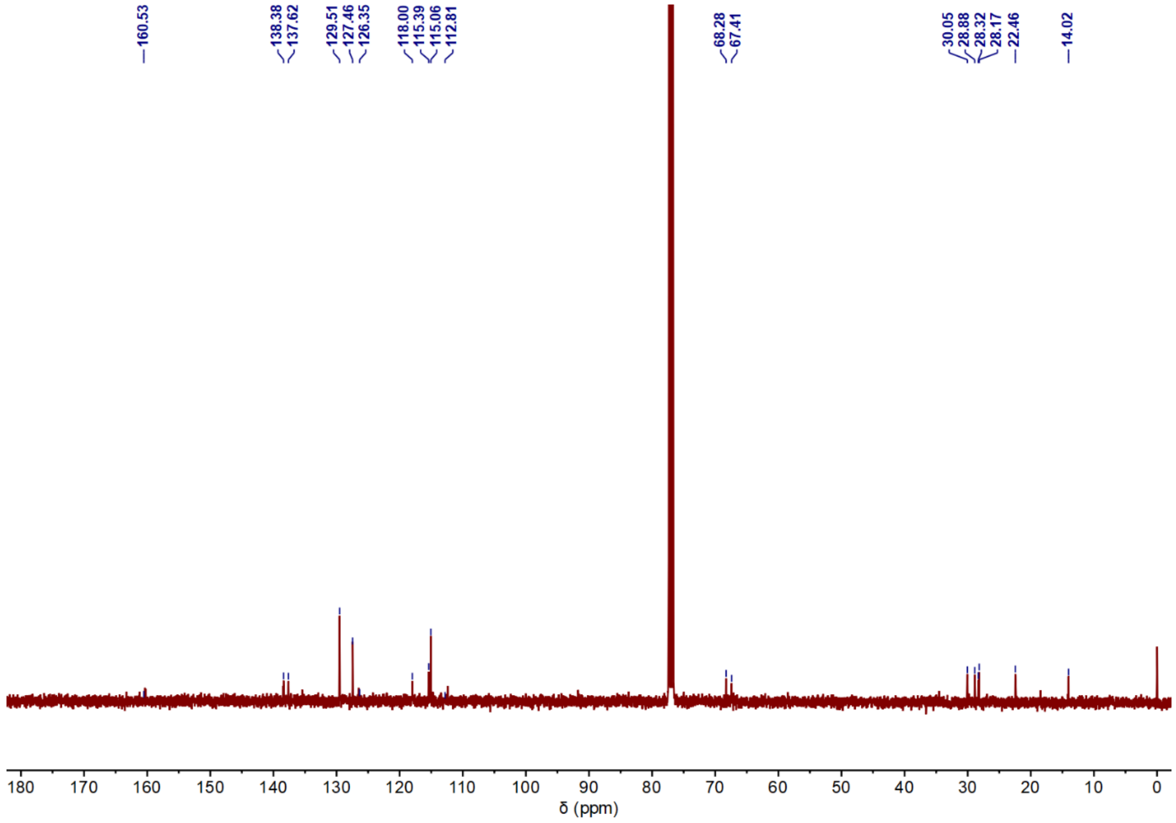

Figure S74: ^{13}C NMR (100 MHz, $CDCl_3$) of **3-(4-(2-cyano-2-(4-(pent-4-en-1-yloxy)phenyl)vinyl)phenyl)-2-(4-(pentyloxy)phenyl)acrylonitrile (16)**

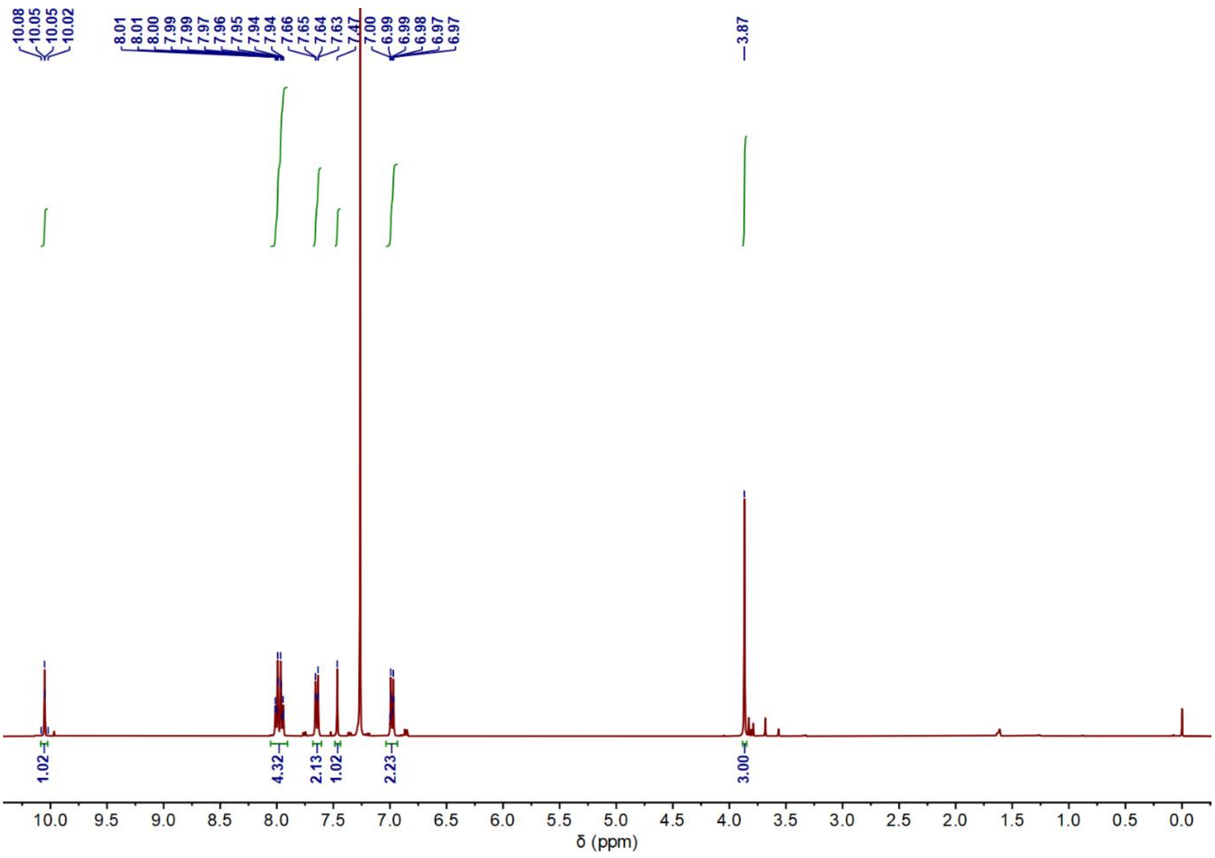

Figure S75: *^{1}H NMR (400 MHz, $CDCl_3$) of* ***3-(4-formylphenyl)-2-(4-methoxyphenyl)acetonitrile (17)***

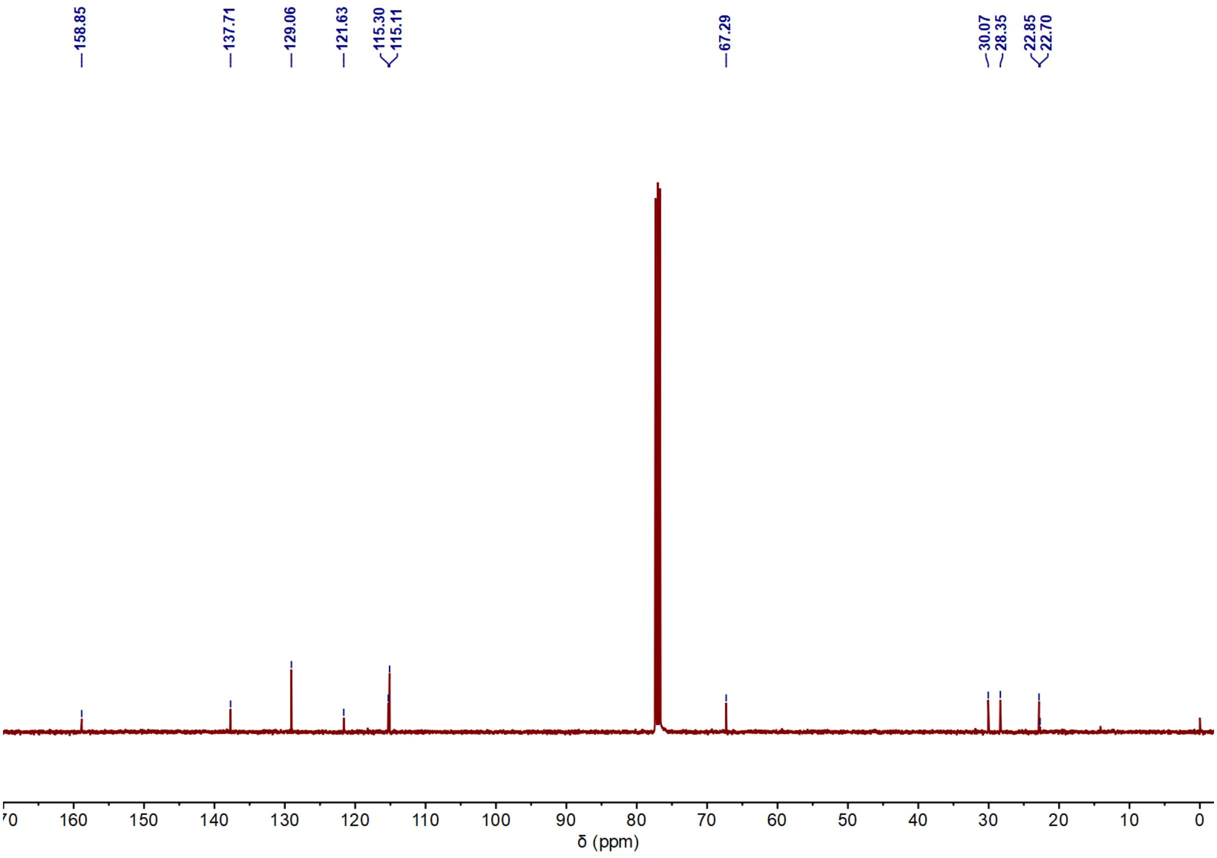

Figure S76: *^{13}C NMR (100 MHz, $CDCl_3$) of* ***3-(4-formylphenyl)-2-(4-methoxyphenyl)acetonitrile (17)***

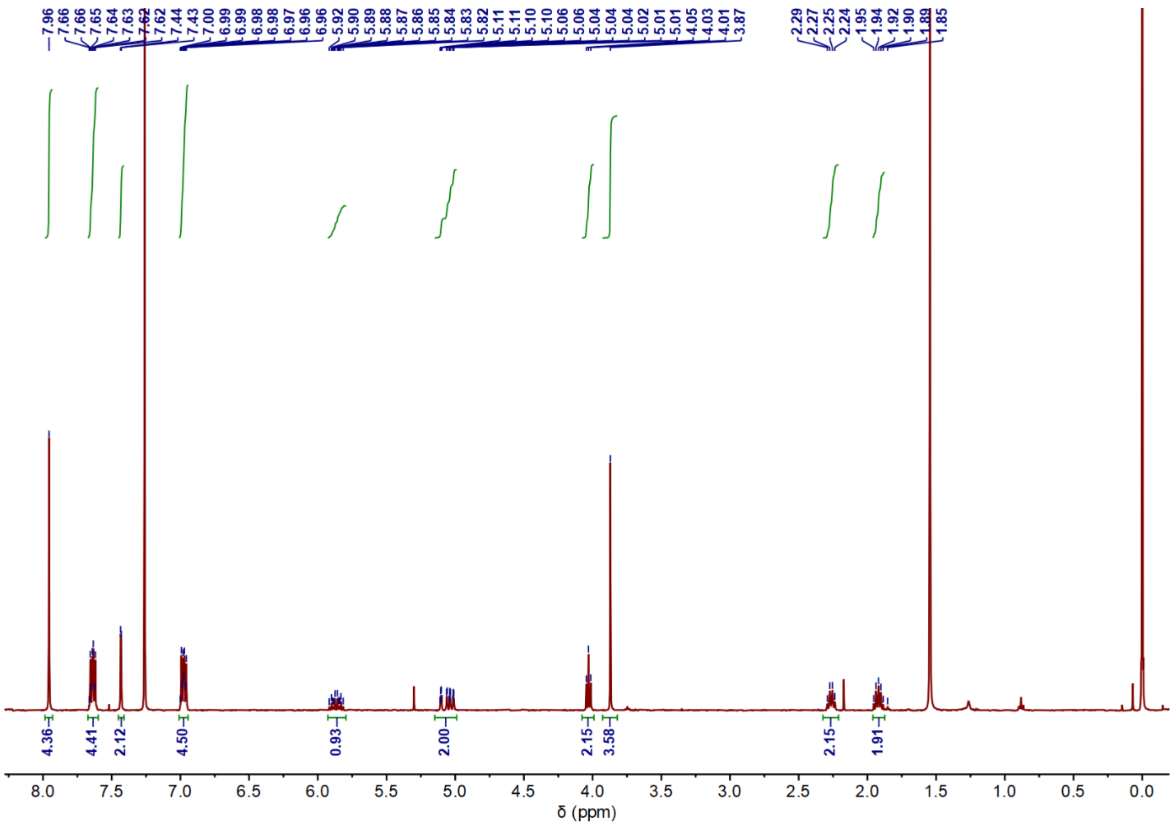

Figure S77: *^{1}H NMR (400 MHz, CDCl$_3$) of* ***3-(4-(2-cyano-2-(4-(pent-4-en-1-yloxy)phenyl)vinyl)phenyl)-2-(4-(methoxy)phenyl)acrylonitrile (18)***

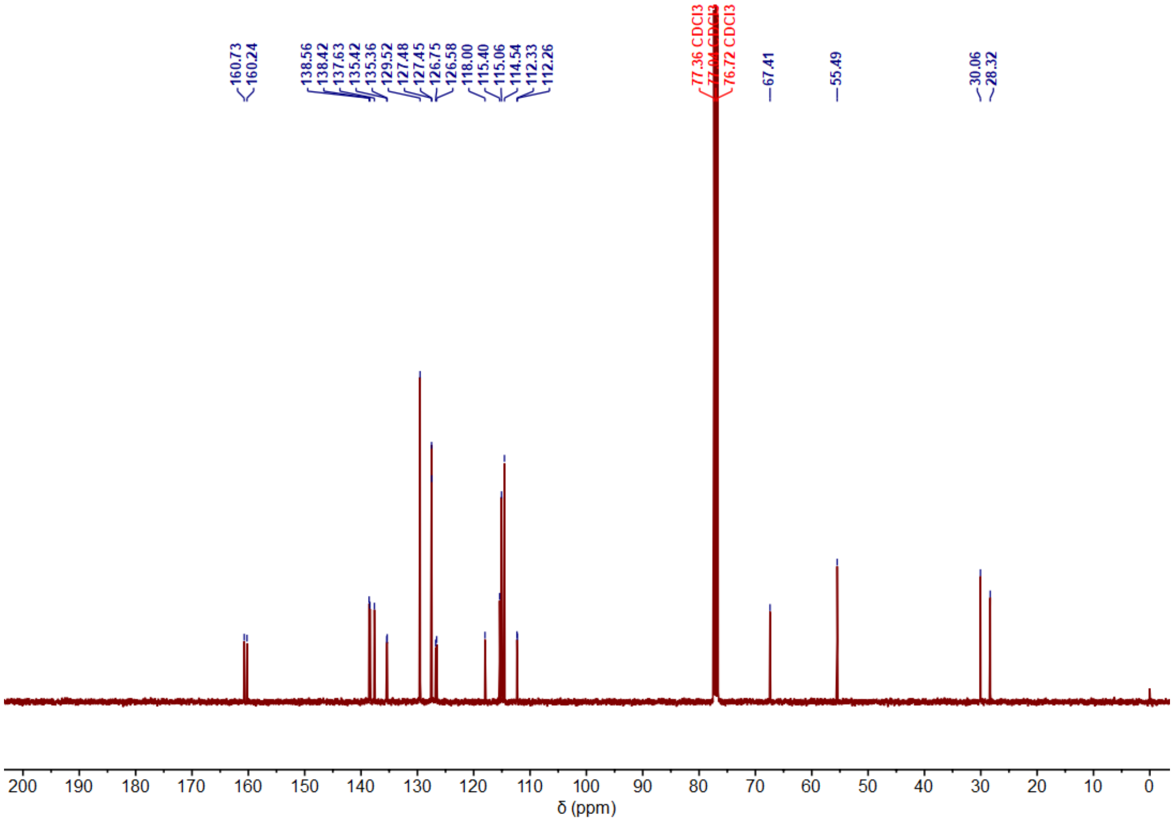

Figure S78: *^{13}C NMR (100 MHz, CDCl$_3$) of* ***3-(4-(2-cyano-2-(4-(pent-4-en-1-yloxy)phenyl)vinyl)phenyl)-2-(4-(methoxy)phenyl)acrylonitrile (18)***

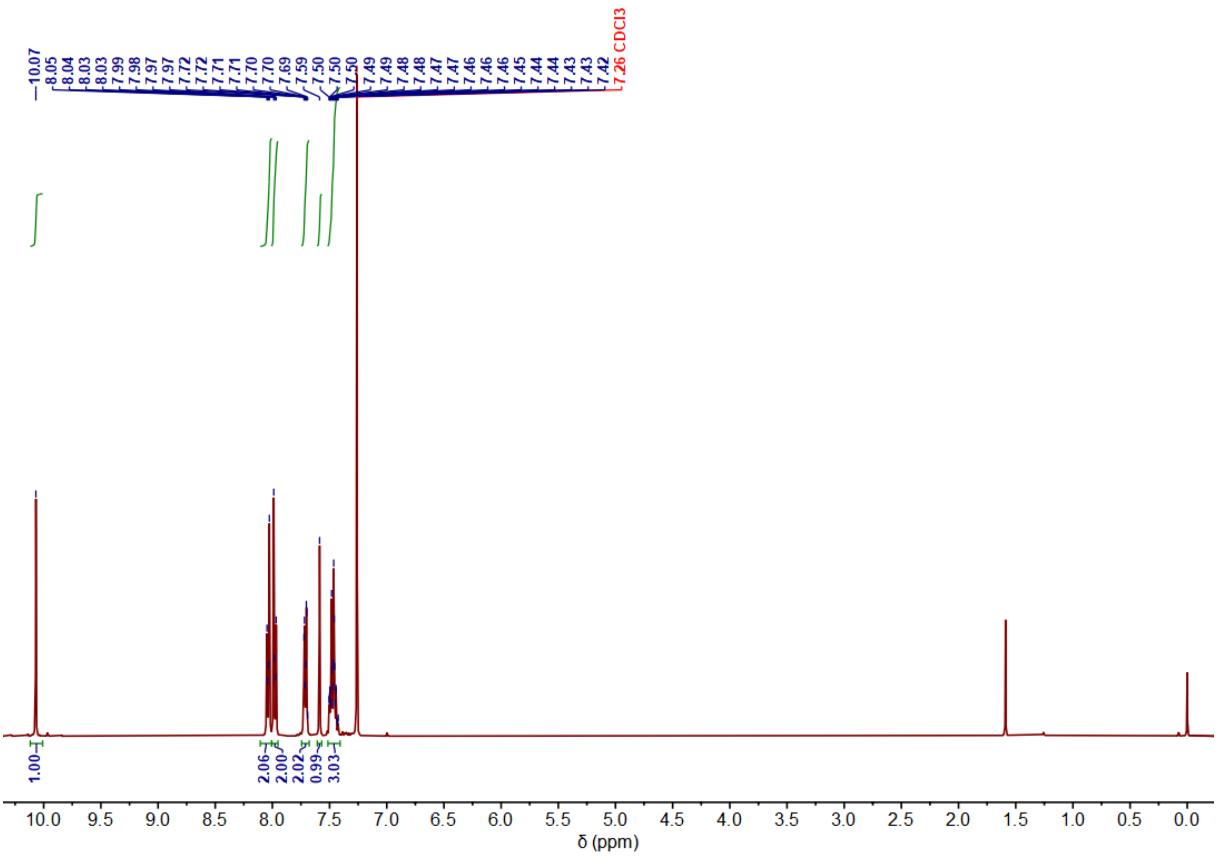

Figure S79: ^{1}H NMR (400 MHz, $CDCl_3$) of **3-(4-formylphenyl)-2-phenylacrylonitrile (19)**

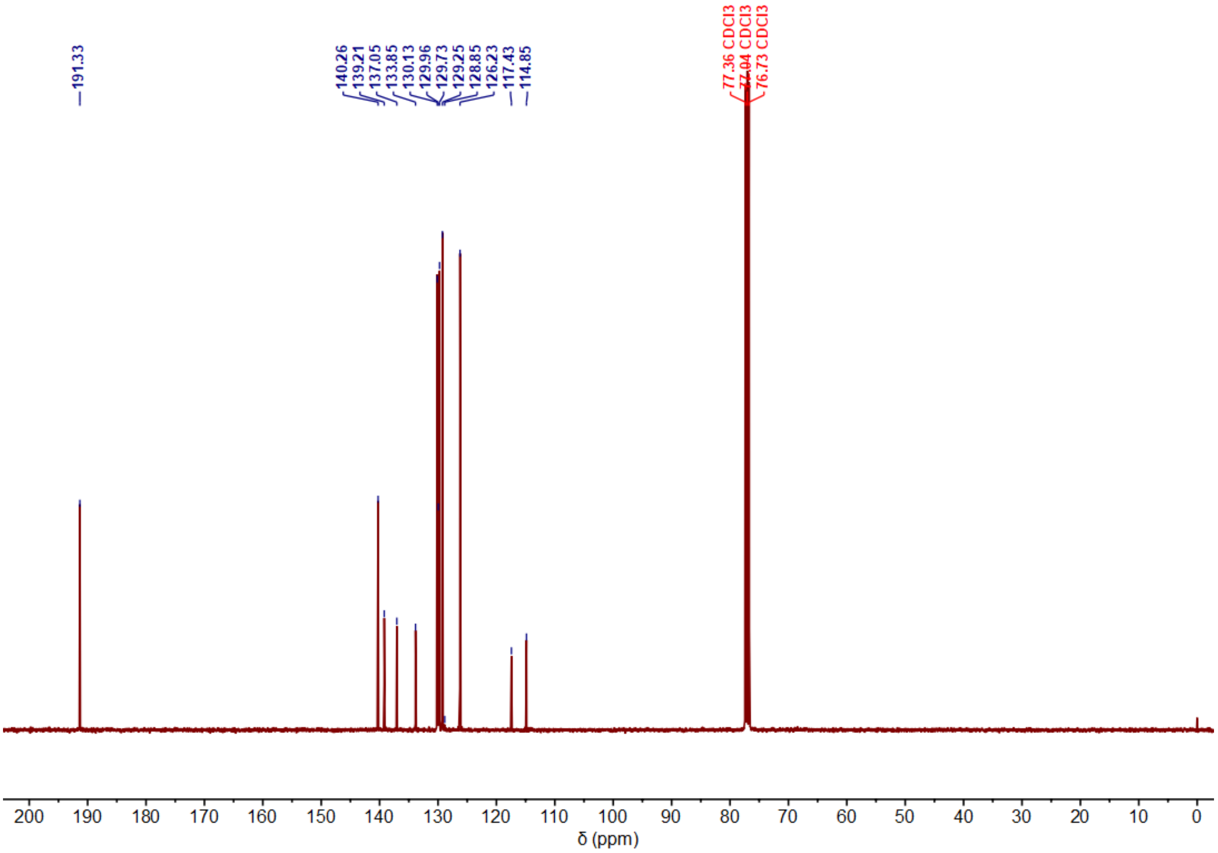

Figure S80: ^{13}C NMR (100 MHz, $CDCl_3$) of **3-(4-formylphenyl)-2-phenylacrylonitrile (19)**

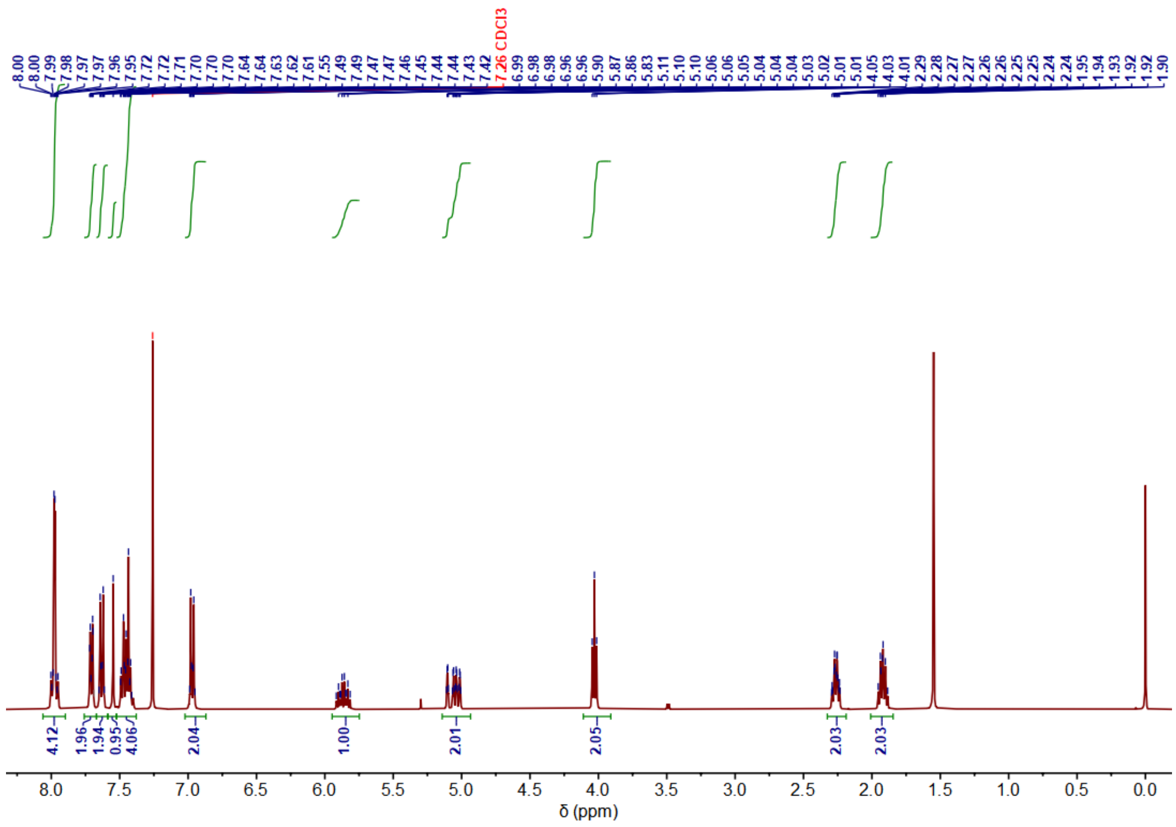

Figure S81**: ^{1}H NMR (400 MHz, $CDCl_3$) of **3-(4-(2-cyano-2-(4-(pent-4-en-1-yloxy)phenyl)vinyl)phenyl)-2-phenylacrylonitrile (20)

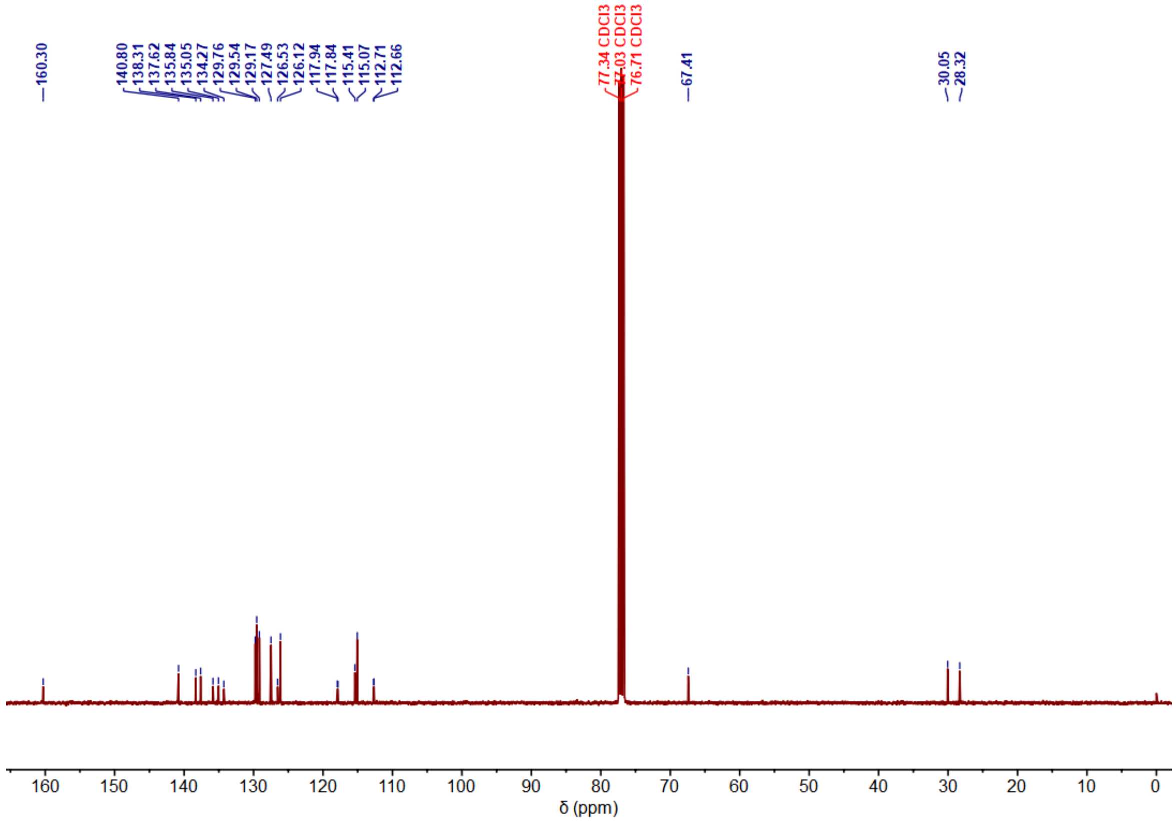

Figure S82**: ^{13}C NMR (100 MHz, $CDCl_3$) of **3-(4-(2-cyano-2-(4-(pent-4-en-1-yloxy)phenyl)vinyl)phenyl)-2-phenylacrylonitrile (20)

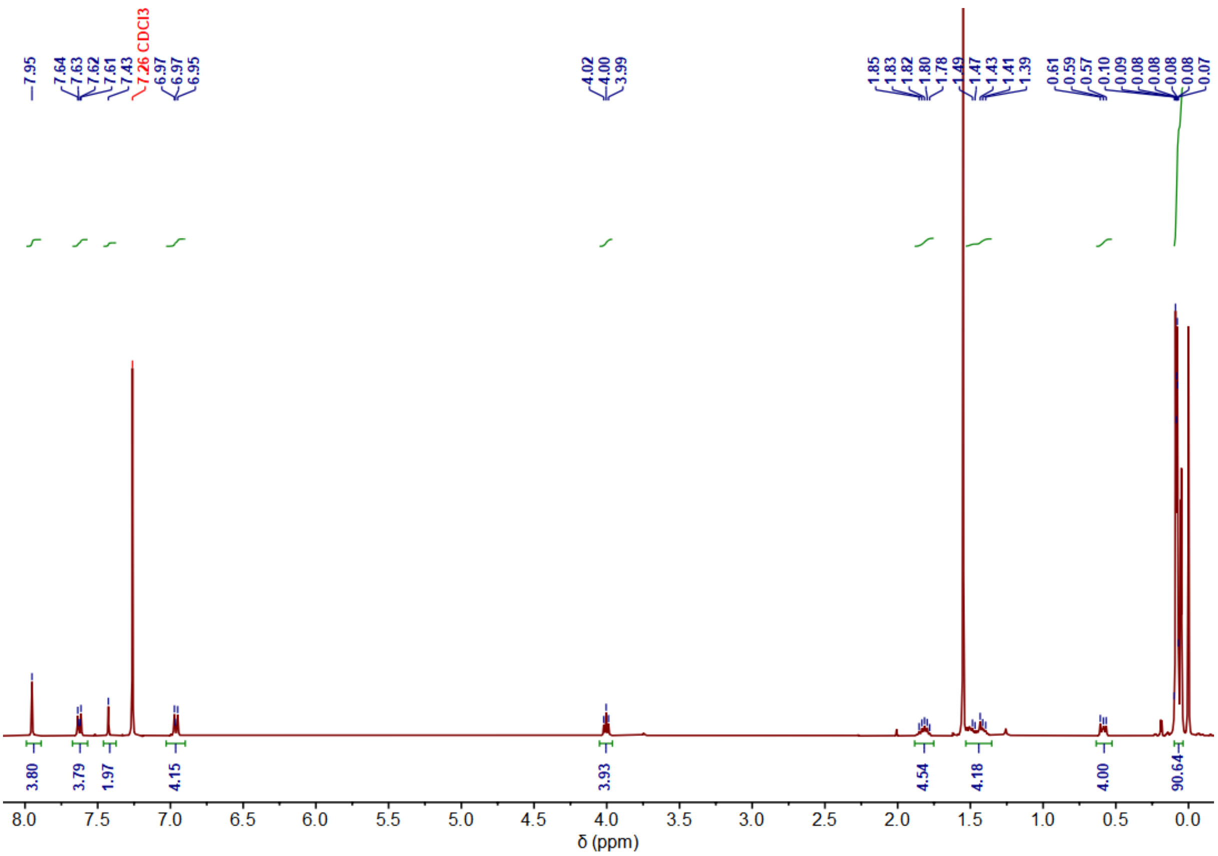

Figure S83: ^{1}H NMR (400 MHz, CDCl$_3$) of ***Si$_7$-OPV-Si$_7$***

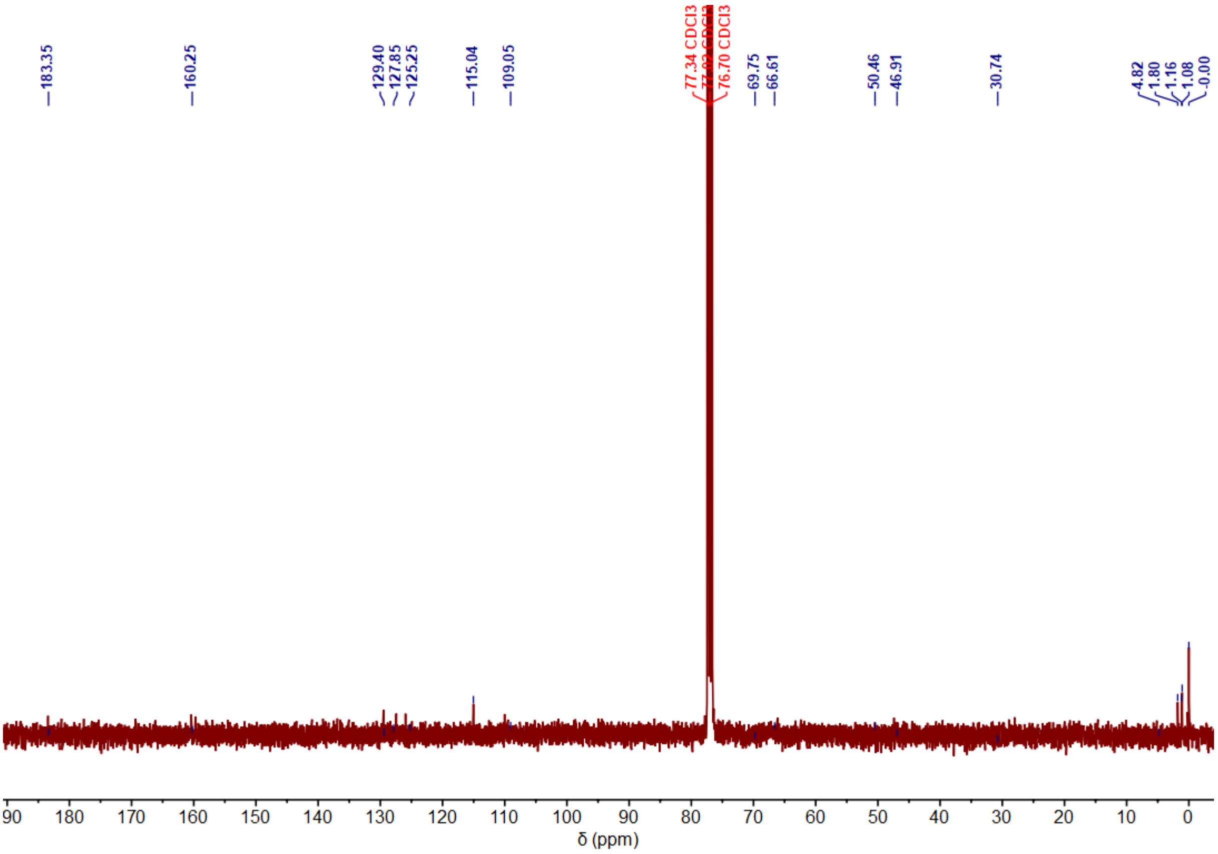

Figure S84: ^{13}C NMR (100 MHz, CDCl$_3$) of ***Si$_7$-OPV-Si$_7$***

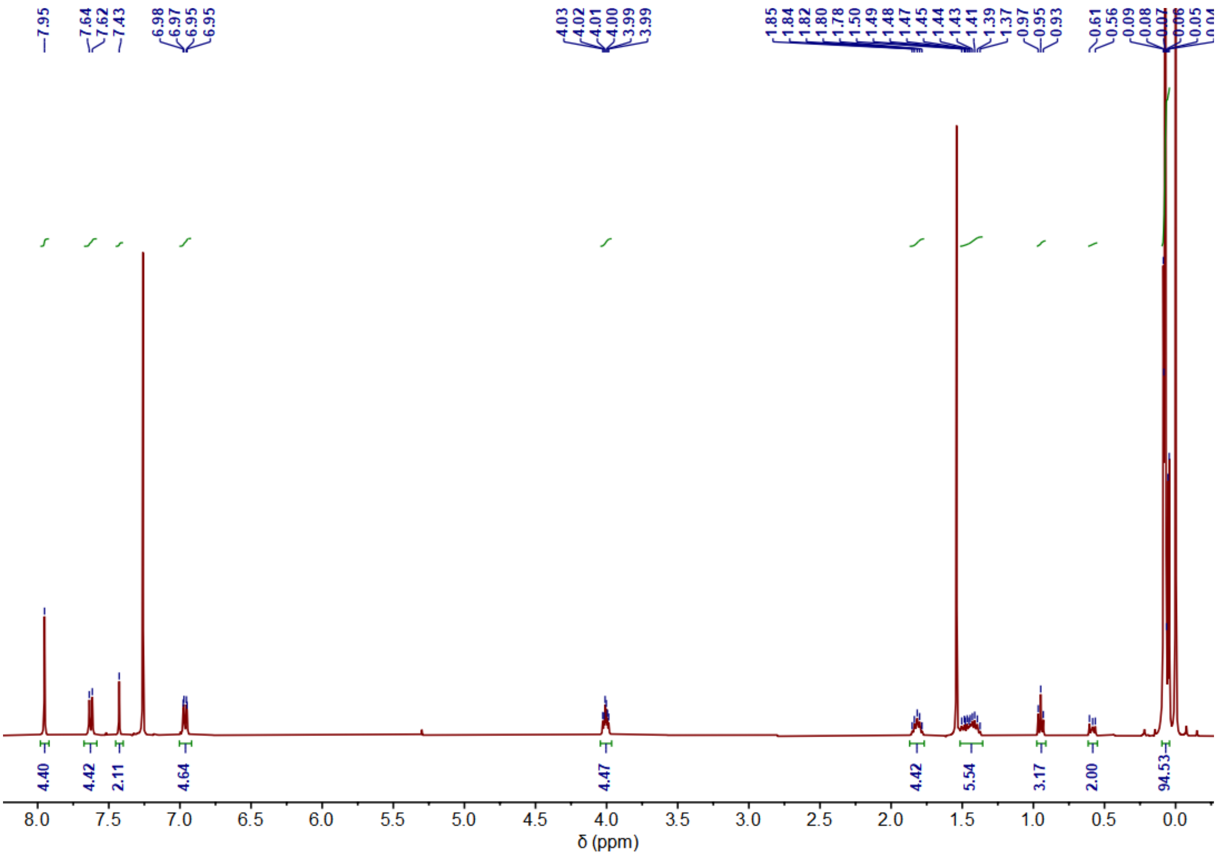

Figure S85**: ^{1}H NMR (400 MHz, $CDCl_3$) of **Pent-OPV-Si$_{15}$

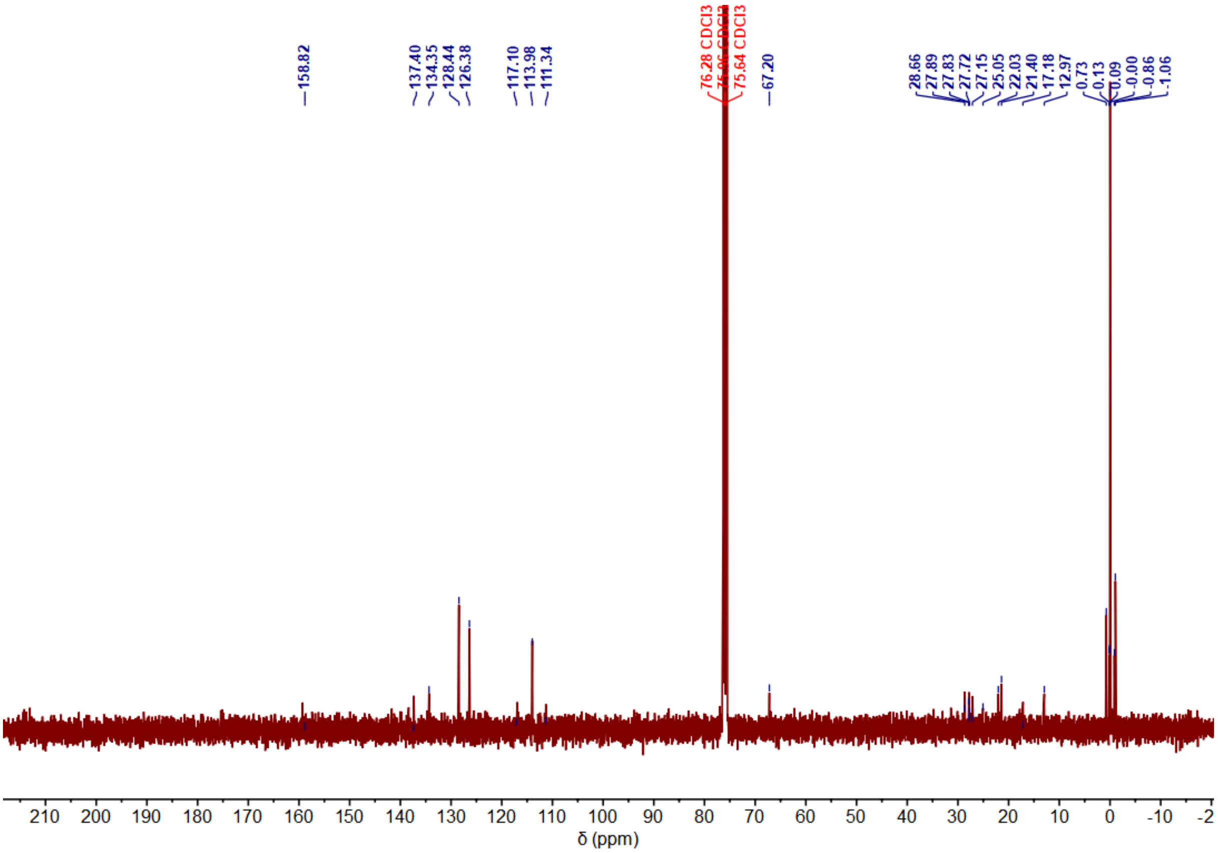

Figure S86**: ^{13}C NMR (100 MHz, $CDCl_3$) of **Pent-OPV-Si$_{15}$

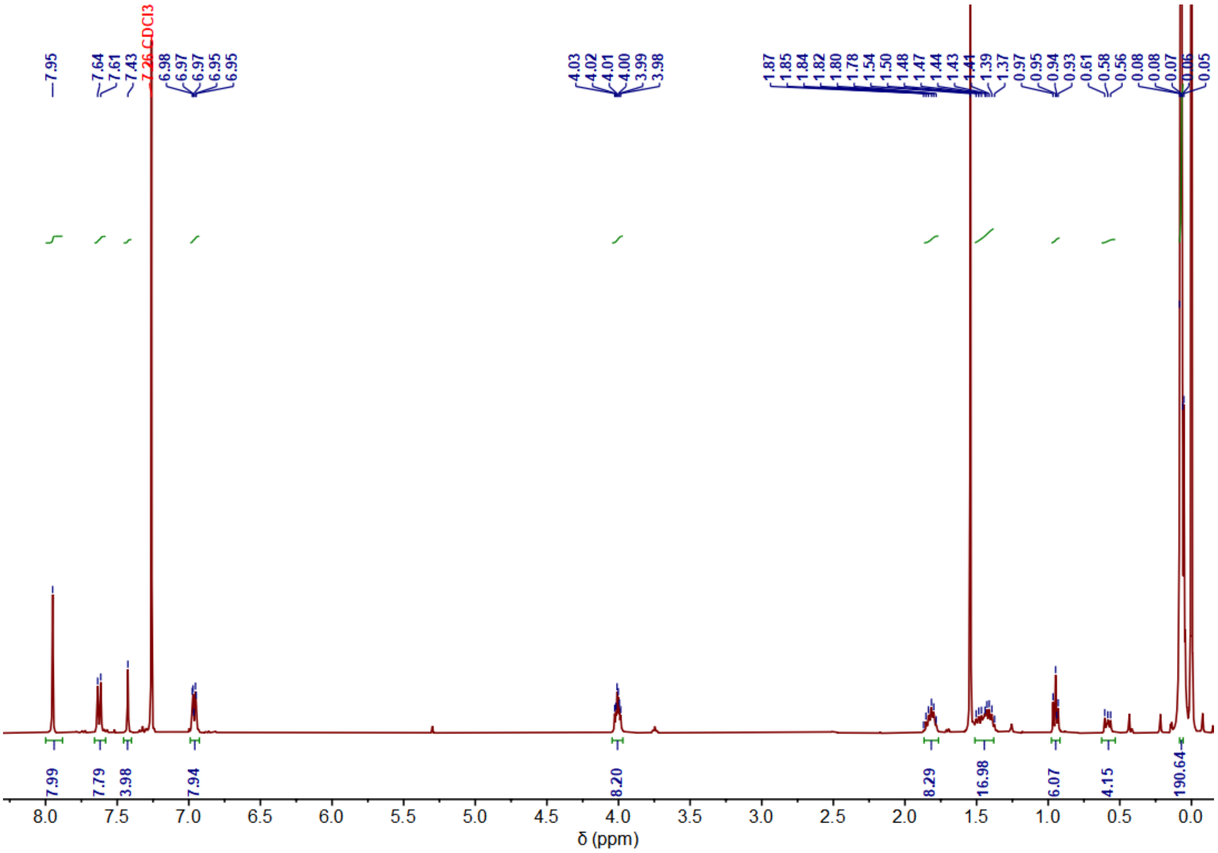

Figure S87: ^{1}H NMR (400 MHz, $CDCl_3$) of **Pent-OPV-Si$_{32}$-OPV-Pent**

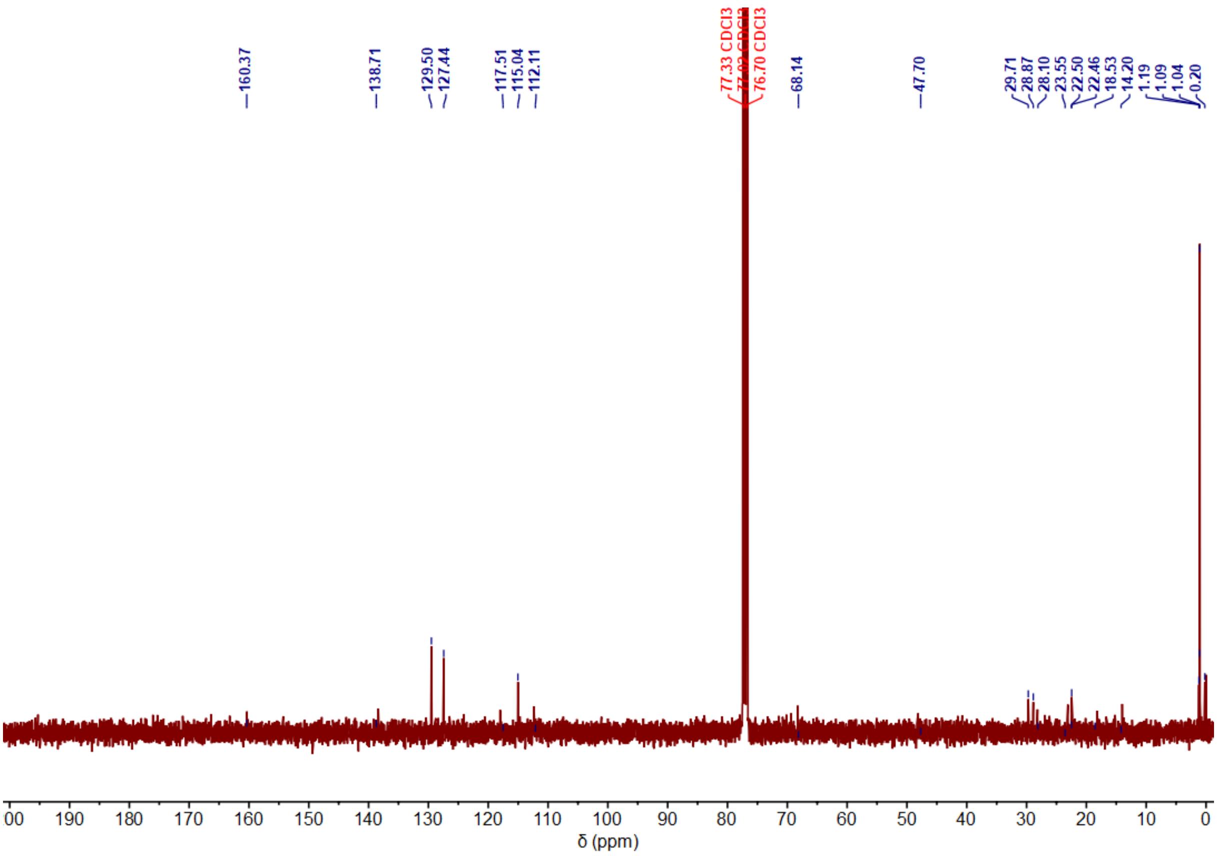

Figure S88: ^{13}C NMR (100 MHz, $CDCl_3$) of **Pent-OPV-Si$_{32}$-OPV-Pent**

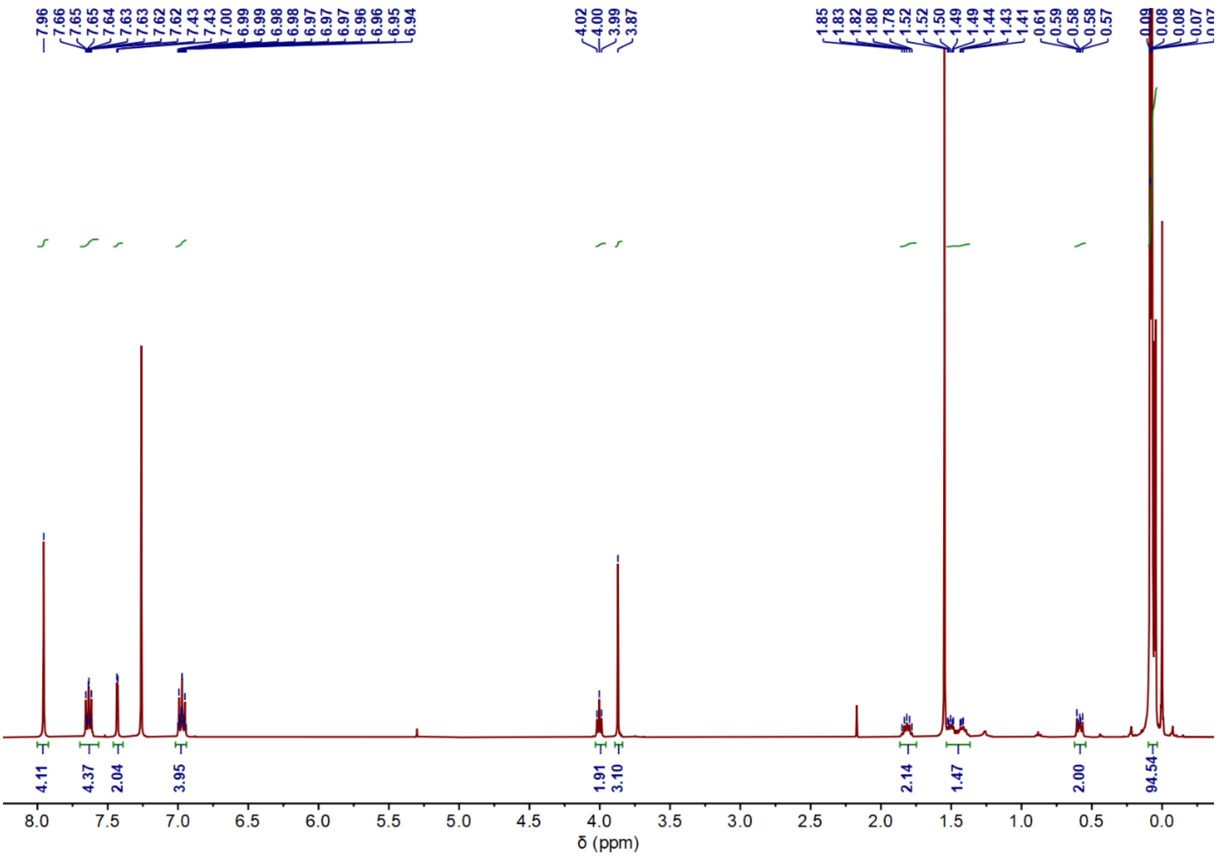

Figure S89**: ¹H NMR (400 MHz, $CDCl_3$) of **Me-OPV-Si$_{15}$

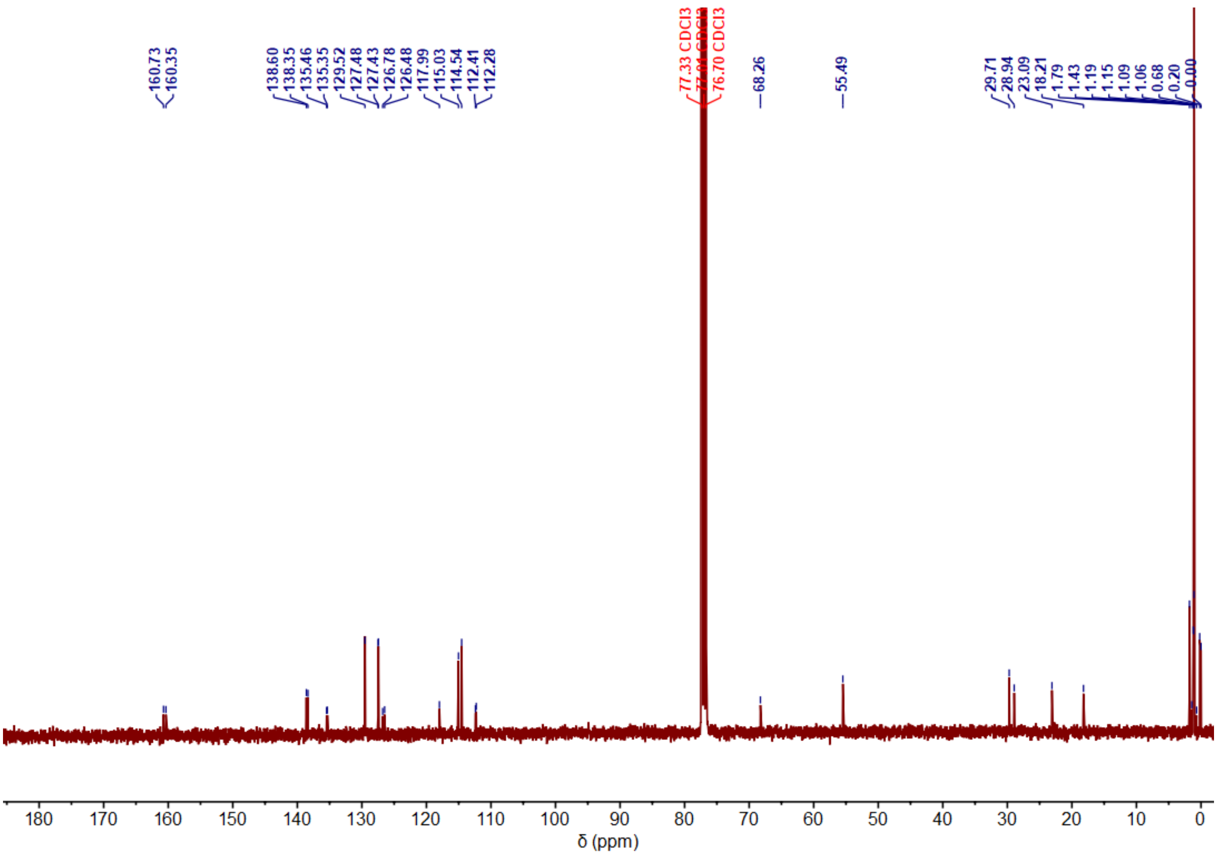

Figure S90**: ¹³C NMR (100 MHz, $CDCl_3$) of **Me-OPV-Si$_{15}$

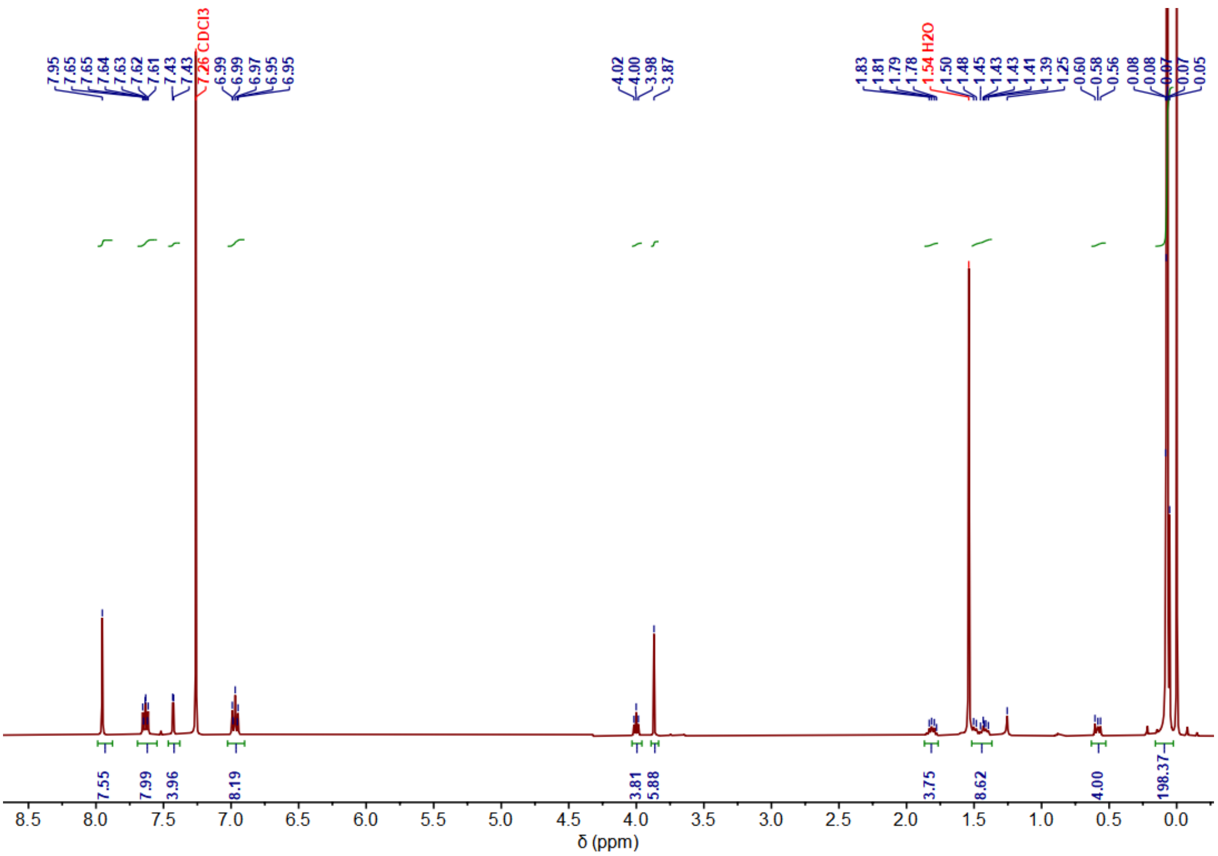

Figure S91: ^{1}H NMR (400 MHz, $CDCl_3$) of **Me-OPV-Si$_{32}$-OPV-Me**

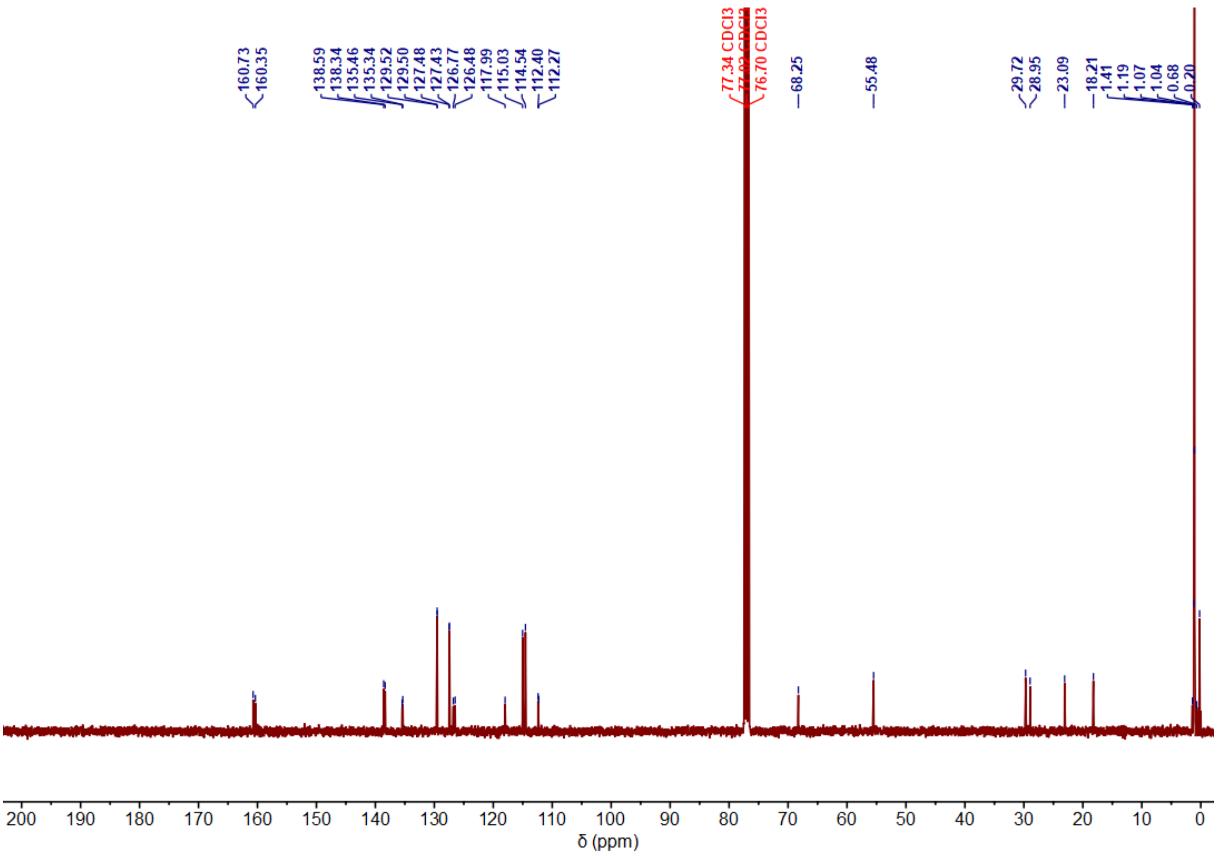

Figure S92: ^{13}C NMR (100 MHz, $CDCl_3$) of **Me-OPV-Si$_{32}$-OPV-Me**

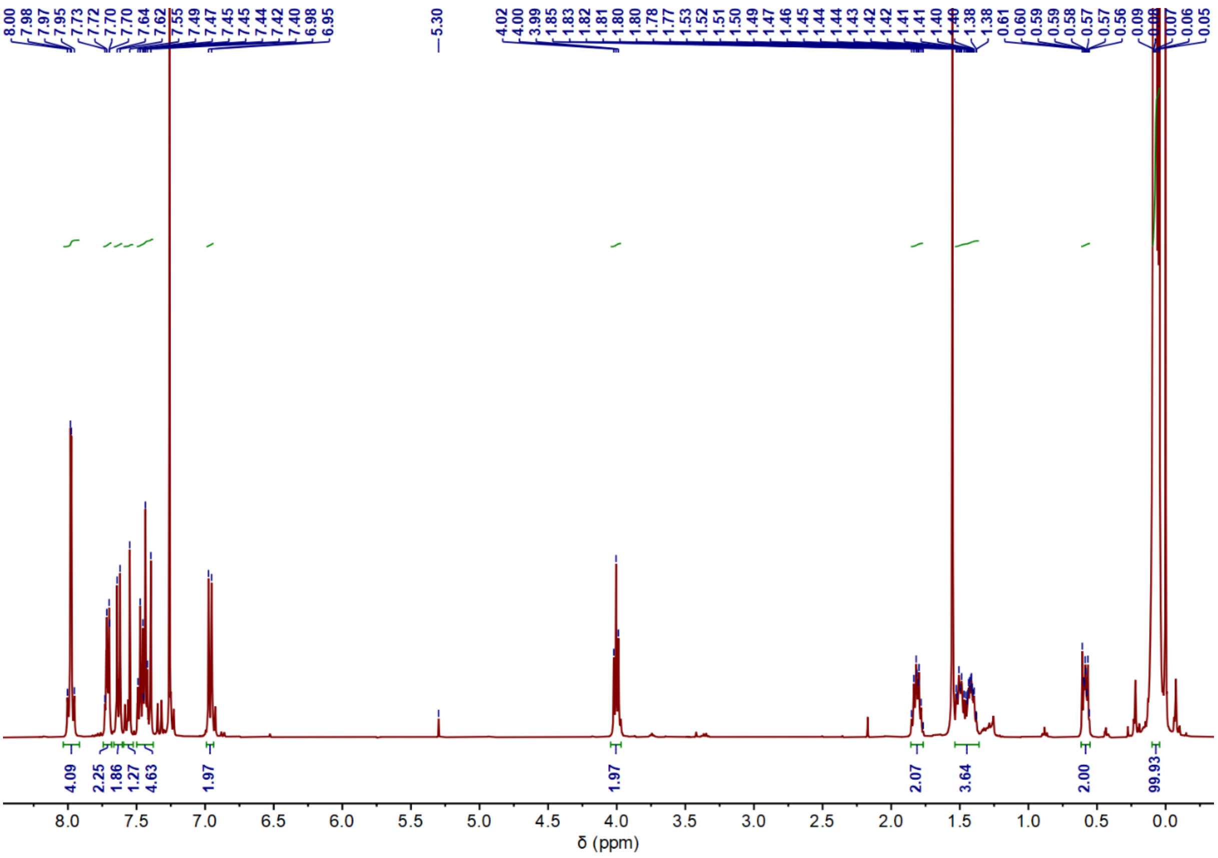

Figure S93**: ^{1}H NMR (400 MHz, $CDCl_3$) of **H-OPV-Si$_{15}$

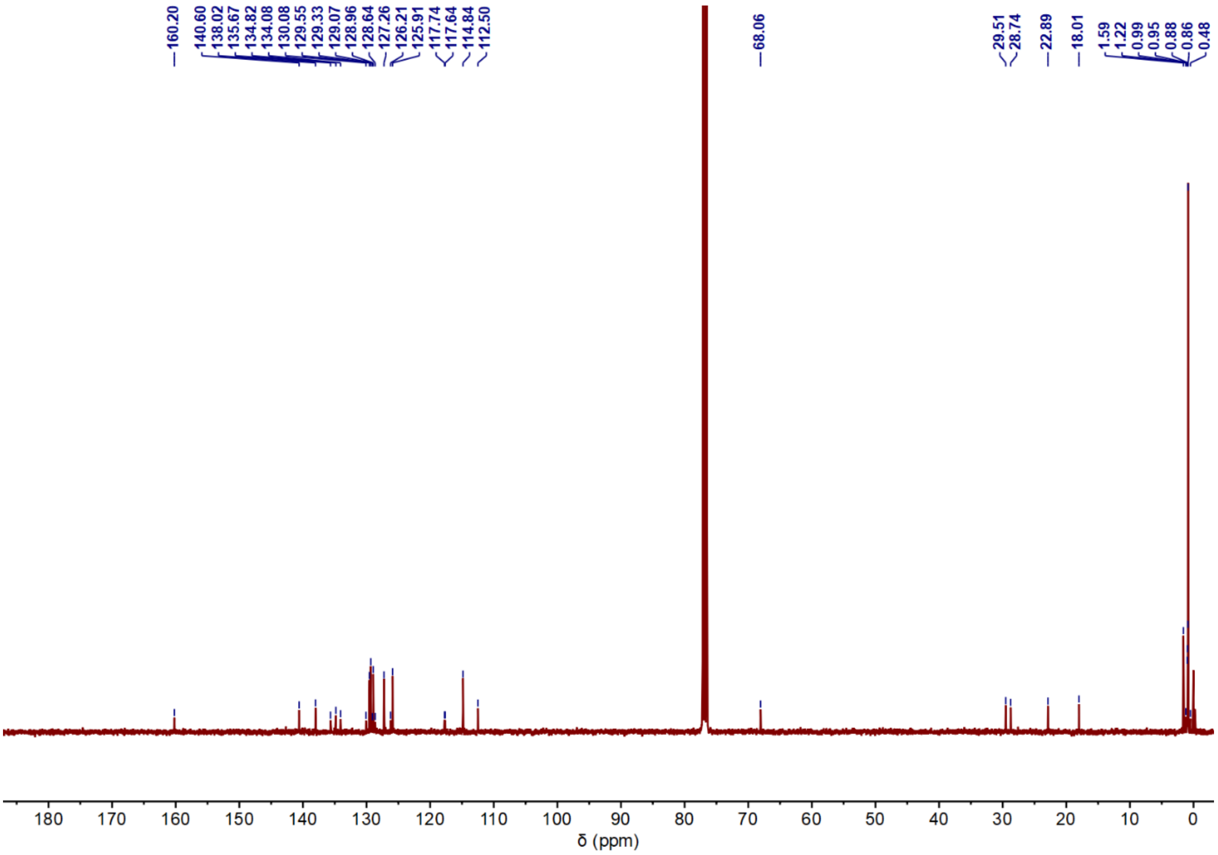

Figure S94**: ^{13}C NMR (100 MHz, $CDCl_3$) of **H-OPV-Si$_{15}$

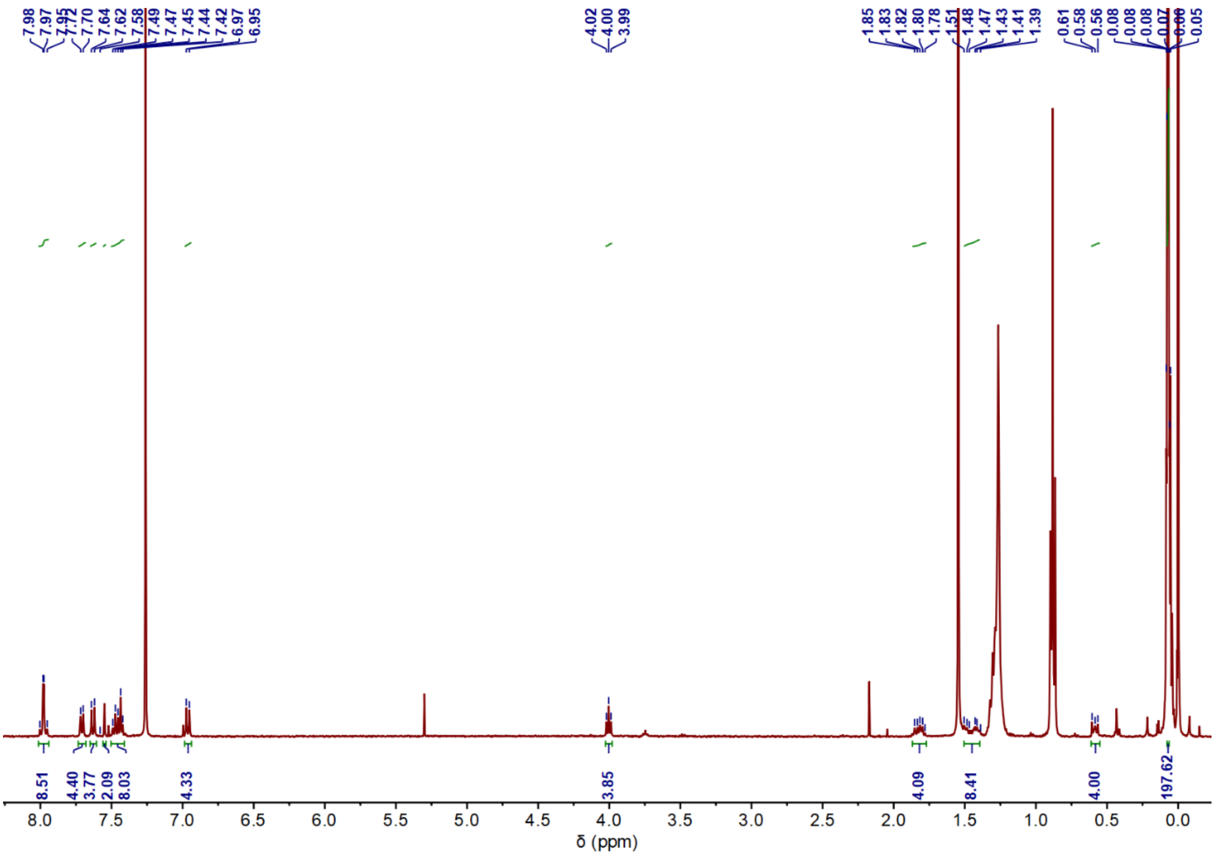

Figure S95: ^{1}H NMR (400 MHz, $CDCl_3$) of ***H-OPV-Si$_{32}$-OPV-H***

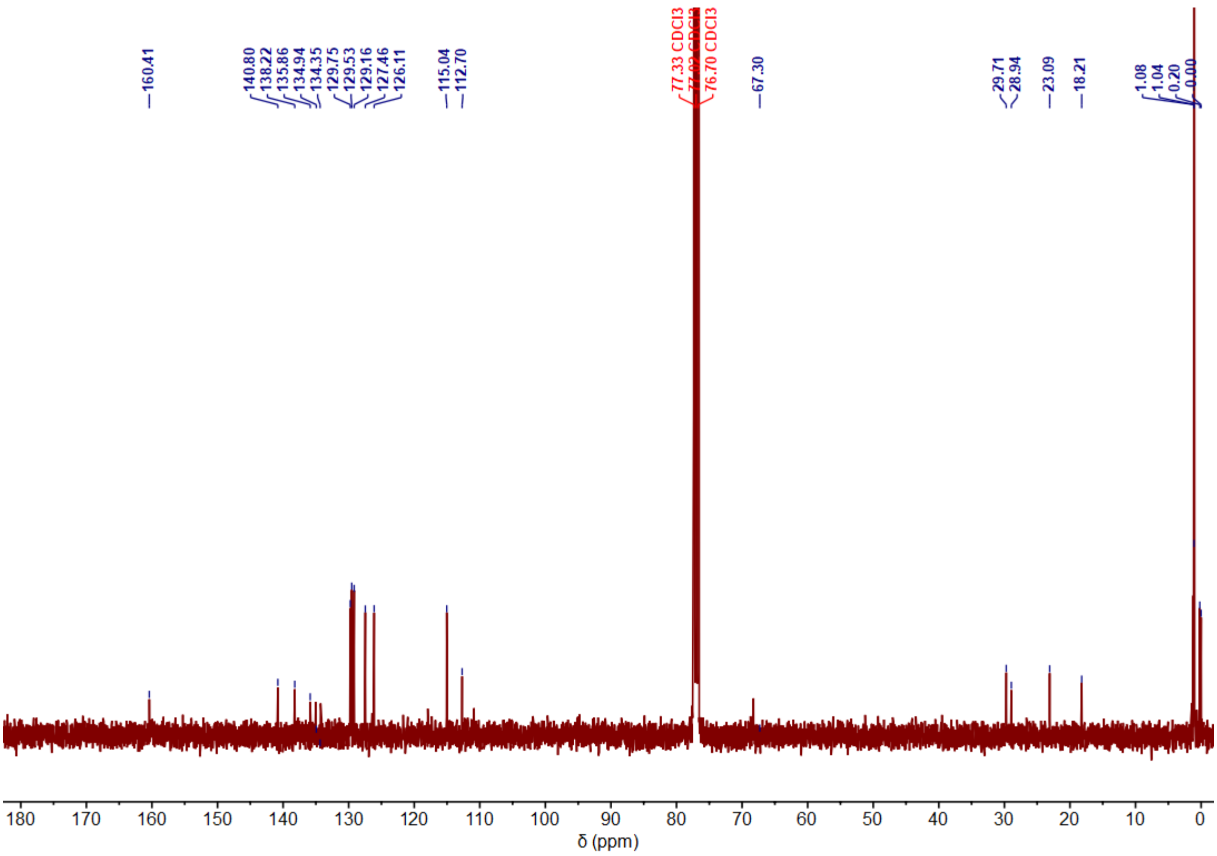

Figure S96: ^{13}C NMR (100 MHz, $CDCl_3$) of ***H-OPV-Si$_{32}$-OPV-H***

5. References

(1) Lamers, B. A. G.; Waal, B. F. M. de; Meijer, E. W. The Iterative Synthesis of Discrete Dimethylsiloxane Oligomers: A Practical Guide. *J. Polym. Sci.* **2021**, *59* (12), 1142–1150.

(2) Van Genabeek, B.; De Waal, B. F. M.; Gosens, M. M. J.; Pitet, L. M.; Palmans, A. R. A.; Meijer, E. W. Synthesis and Self-Assembly of Discrete Dimethylsiloxane-Lactic Acid Diblock Co-Oligomers: The Dononacontamer and Its Shorter Homologues. *J. Am. Chem. Soc.* **2016**, *138* (12), 4210–4218.

(3) Salzillo, T.; Della Valle, R. G.; Venuti, E.; Brillante, A.; Siegrist, T.; Masino, M.; Mezzadri, F.; Girlando, A. Two New Polymorphs of the Organic Semiconductor 9,10-Diphenylanthracene: Raman and X-Ray Analysis. *J. Phys. Chem. C* **2016**, *120* (3), 1831–1840.

(4) Ohta, A.; Hattori, K.; Kusumoto, Y.; Kawase, T.; Kobayashi, T.; Naito, H.; Kitamura, C. Effects of Alkoxy Substitution on the Optical Properties of 9,10-Anthraquinone and Anthracene: 2,3,6,7-Tetrapropoxy-Substituted vs. 2,6-Dipropoxy-Substituted Derivatives. *Chem. Lett.* **2012**, *41* (7), 674–676.

(5) Norikane, Y.; Uchida, E.; Tanaka, S.; Fujiwara, K.; Koyama, E.; Azumi, R.; Akiyama, H.; Kihara, H.; Yoshida, M. Photoinduced Crystal-to-Liquid Phase Transitions of Azobenzene Derivatives and Their Application in Photolithography Processes through a Solid-Liquid Patterning. *Org. Lett.* **2014**, *16* (19), 5012–5015.

(6) Varghese, S.; Yoon, S. J.; Casado, S.; Fischer, R. C.; Wannemacher, R.; Park, S. Y.; Gierschner, J. Orthogonal Resonator Modes and Low Lasing Threshold in Highly Emissive Distyrylbenzene-Based Molecular Crystals. *Adv. Opt. Mater.* **2014**, *2* (6), 542–548.

(7) Son, M. H. C. van; Berghuis, A. M.; Eisenreich, F.; Waal, B. de; Vantomme, G.; Rivas, J. G.; Meijer, E. W. Highly Ordered 2D-Assemblies of Phase-Segregated Block Molecules for Upconverted Linearly Polarized Emission. *Adv. Mater.* **2020**, *32* (48), 2004775.
